# Proteomic analysis of *Salmonella enterica *serovar Enteritidis following propionate adaptation

**DOI:** 10.1186/1471-2180-10-249

**Published:** 2010-09-28

**Authors:** Leona N Calhoun, Rohana Liyanage, Jackson O Lay, Young Min Kwon

**Affiliations:** 1 Cell and Molecular Biology Program, Department of Poultry Science, University of Arkansas, 1260 W. Maple Avenue, Fayetteville, AR 72701, USA; 2 State Wide Mass Spectrometry Facility, Chemistry and Biochemistry Department, University of Arkansas, 119 Chemistry Building, Fayetteville, AR 72701, USA

## Abstract

**Background:**

*Salmonella *Enteritidis is a highly prevalent and persistent foodborne pathogen and is therefore a leading cause of nontyphoidal gastrointestinal disease worldwide. A variety of stresses are endured throughout its infection cycle, including high concentrations of propionate (PA) within food processing systems and within the gut of infected hosts. Prolonged PA exposure experienced in such milieus may have a drastic effect on the proteome of *Salmonella *Enteritidis subjected to this stress.

**Results:**

In this study, we used 2 D gel electrophoresis to examine the proteomes of PA adapted and unadapted *S*. Enteritidis and have identified five proteins that are upregulated in PA adapted cultures using standard peptide mass fingerprinting by MALDI-TOF-MS and sequencing by MALDI LIFT-TOF/TOF tandem mass spectrometry. Of these five, two significant stress-related proteins (Dps and CpxR) were shown (via qRT-PCR analysis) to be upregulated at the transcriptional level as well. Unlike the wild type when adapted to PA (which demonstrates significant acid resistance), PA adapted *S*. Enteritidis ∆*dps *and *S*. Enteritidis ∆*cpxR *were at a clear disadvantage when challenged to a highly acidic environment. However, we found the acid resistance to be fully restorable after genetic complementation.

**Conclusions:**

This work reveals a significant difference in the proteomes of PA adapted and unadapted *S*. Enteritidis and affirms the contribution of Dps and CpxR in PA induced acid resistance.

## Background

*Salmonella enterica *Serovar Enteritidis (*S*. Enteritidis) is a facultative intracellular pathogen responsible for acute gastroenteritis and is currently the second most frequently isolated serovar in the United States - accounting for nearly 15% of total cases of human salmonellosis [1]. *S*. Enteritidis maintains its status as a leading cause of foodborne infections mainly due to its prevalence in poultry products and its environmental persistence despite the harsh conditions it encounters. The survival of this pathogen under intense conditions has been linked to its remarkable ability to quickly respond to environmental signals and adapt to its surroundings, as well as the induction of specific stress responses during environmental adaptation [2-6].

Throughout its infection cycle, *S*. Enteritidis encounters several distinctive environments including those rich in the short chain fatty acids (SCFAs) acetate, propionate (PA), and butyrate. PA is one of many SCFAs deemed acceptable for use in food preservation and is frequently employed to suppress bacterial growth in foods such as meat, salad dressing, and mayonnaise [7]. Also, the anaerobic environment of the mammalian ileum, cecum, and colon are rich in SCFAs and accumulate PA as a main byproduct of fermentative bacterial species [8,9]. Although the aforementioned SCFAs are all commonly encountered by *S*. Enteritidis during successful infection, a previous study indicates that PA may play a more important role than other SCFAs in the induction of subsequent stress responses [5]. Food processing systems and the mammalian gut are excellent sources for long term exposure to PA. These milieus also provide ample opportunity for PA-mediated induction of subsequent protective stress responses that may be necessary for survival in environments encountered later in the host infection process and/or environmental persistence.

We are unaware of any study to date that examines the proteomic changes of *S*. Enteritidis following prolonged exposure to environments rich in PA. Completed work has shown that short term exposure to PA (generally one hour) during the exponential growth phase at a neutral pH is correlated with significant changes in protein synthesis in *S*. Typhimurium, which ultimately affords protection during subsequent acid shock [5]. Furthermore, inhibition of protein synthesis during PA adaption ultimately resulted in a significant loss of acid resistance. With the exception of this knowledge, genetic and proteomic changes that occur during PA adaptation continue to be greatly uncharacterized. A comparative proteomic approach is likely to provide a comprehensive view of protein abundances as they vary between the unadapted and PA adapted condition. Furthermore, proteomic examination of PA adapted cells could quite possibly lead to the elucidation for putative virulence factors of this organism. In order to contribute to the current knowledge of molecular changes that occur in *S*. Enteritidis during PA adaptation, a global analysis of the cellular proteins in PA adapted and unadapted cultures was completed using two-dimensional gel electrophoresis and is described herein. We focused on a small subset of proteins that showed intense overexpression in PA adapted cultures and targeted them for in gel trypsin digestion followed by protein identification via peptide mass finger printing using MALDI TOF mass spectrometry [10,11]. Among proteins upregulated specifically in response to PA are those that function as transcriptional regulators (CpxR), as well as those that serve in a direct protective capacity under stressful conditions (Dps). Further examination of PA adapted cultures via quantitative real-time PCR revealed overexpression of *dps *and *cpxR *at the transcriptional level as well. Via deletion mutant and complementation studies, we were able to correlate the expression of these genes with the induction of an acid resistant phenotype in *S*. Enteritidis after long term PA adaptation.

## Methods

### Growth conditions and bacterial strains

The wild type strain *Salmonella *Enteritidis LK5 used in this study is a chicken isolate [12]. *E. coli *TOP10 was used for the initial propagation of pUC19 based plasmids. All bacteria were routinely propagated using Luria-Bertani (LB) media (The base level of sodium in this medium is 10 g/L or 171 mM). Growth media were supplemented with appropriate antibiotics when necessary at the following concentrations: kanamycin (Km, 50 μg/ml), ampicillin (Amp, 100 μg/ml). All plates and cultures were incubated at 37°C unless otherwise stated.

### PA adaptation of *S*. Enteritidis

*S*. Enteritidis LK5 was grown in 4 ml of LB broth overnight with vigorous agitation (225 rpm). Ten microliters from this overnight culture was subcultured into 2 ml of fresh LB broth containing 100 mM of propionate (pH 7.0; neutral pH of adapting media was achieved by the addition of 1 M NaOH). Two negative controls were utilized in initial acid challenge studies of wild type *S*. Enteritidis. For these control cultures, 10 μl of the overnight LK5 culture used to inoculate the PA adapted culture was also subcultured into 2 ml of either unsupplemented LB broth (pH 7.0) or LB broth containing 100 mM NaCl. Adapted and unadapted cultures were then grown statically (in order to mimic natural adaptation) for 16 hours exactly. It is important to note that the pH level of the growth medium containing PA was minimally affected after 16 hour adaptation. Prior to adaptation, the pH was 7.0. Post adaptation, the pH was 6.8. Therefore, the neutrality of the adaptation media remained intact throughout the experiment.

### Two-Dimensional (2D) Gel Electrophoresis

Following adaptation, the soluble protein extracts from both PA adapted and unadapted cultures were isolated using a Qproteome Bacterial Protein Prep Kit (Qiagen^©^) and subsequently used for two-dimensional gel electrophoresis. Immobiline™DryStrips (pH 3-10 NL, GE Healthcare) were used for isoelectric focusing on the IPGPhor system (Amersham Pharmacia) according to the manufacturer's instructions. Gels strips were loaded with 100 μg of protein sample, rehydrated for 16 hours in a rehydration solution (8 M urea, 2% CHAPS_3 _(w/v), trace amounts bromophenol blue, 0.5% IPG buffer (pH 3-10 NL), and 0.2% dithiothreitol (DTT)) and focused using the following conditions: 500 V, 30 minutes, current 0.25 mA; 1000 V, 30 minutes, current 0.5 mA; 5000 V, 1 hour 30 minutes, current 8.0 mA. Gel strips were equilibrated following isoelectric focusing using an SDS equilibration buffer (50 mM Tris-Cl pH 8.8, 6 M urea, 30% glycerol (w/v), 2% SDS (w/v), trace amounts bromophenol blue) once in the presence of 10 mg/mL DTT, and a second time (to reduce point streaking and other artifacts) in the presence of 25 mg/mL iodoacetamide. Following equilibration, proteins were separated according to their molecular weight on 12% SDS PAGE mini gels using a Hoefer SE 260 unit (Hoefer) at 100 V for the stacking period followed by a two hour run at 200 V. Gels were then fixed overnight in a solution of 40% ethanol and 10% acetic acid in ultrapure water, stained using the SilverQuest™silver staining kit (Invitrogen) per manufacturer's instructions and stored in 10% glycerol (v/v). Five replicate gels were prepared for both PA adapted and unadapted cultures from independently grown cultures. Prior to protein extraction, gel images were analyzed using Melanie 5.0 2 D gel electrophoresis analysis software (Swiss Institute of Bioinformatics, Geneva, Switzerland) to detect differences in protein abundance between PA adapted and unadapted gels. Spots were processed by total spot volume normalization. Also, background was subtracted from each spot intensity volume in order to obtain each spot volume percentage. This percentage value was used for comparison. A Student's *t*-test (*p *= 0.05) was performed to assess whether the means of the two groups of gels were statistically different from each other. Five gel spots corresponding to proteins with statistically significant overexpression (p < 0.05) in PA adapted gels, were carefully excised from PA adapted gels and placed in filter sterilized water for further analysis involving in gel trypsin digestion and protein identification by mass spectrometry.

### Mass Spectrometry analysis of gel spots

Excised gels spots were subjected to in-gel trypsin digestion using standard Bio Rad destaining and in gel trypsin digestion protocols for silver stained gels. After the in gel digestion, the digest was concentrated and desalted using Ziptip procedure (Millipore, Bedford, MA) as suggested by the manufacturer, and eluted with about 5 μl of 60% acetonitrile containing 0.1% formic acid. Two microliters of the eluted sample were then mixed with equal volume of saturated α-cyano-4-hydrocinnamic acid in 34% acetonitrile and spotted on a ground stainless steel MALDI target (Bruker MTP 384 ground steel) and followed by MALDI-TOF (MS) and MALDI LIFT-TOF/TOF [13] (MS/MS) measurements using Ultraflex II MALDI TOF/TOF (Bruker Daltonics GMBH, Bremen, Germany) in its positive ion mode. Mass spectrometer was calibrated externally by using Bruker peptide calibration standard II in the m/z range of 500 to 6000 by spotting the calibration standard immediately next to the sample spot to minimize the mass measurement error. Protein identification was performed using both peptide mass finger printing (PMF) data obtained from the MS mode and peptide sequencing data obtained from the MS/MS mode. MS and the MS/MS data derived as such were subjected to MASCOT data base search using house MASCOT Server. For PMF, the key parameters used to search the spectra against the database were: taxonomy, Bacteria (Eubacteria); fixed modification, carbamidomethyl(C), methionine oxidiation set as variable modification; mass values, monoisotopic; protein mass, unrestricted; peptide mass tolerance, 0.1 Da. For MS/MS search, the same key parameters were used except MS/MS fragment tolerance which was set at 0.5 Da. All proteins were reported as identified only if the MASCOT data base search [14] protein score was statistically significant using both MS and MS/MS search results. Protein score was calculated as -10*Log(P), where P is the probability that the observed match is a random event. Protein scores greater than 77 were considered to be significant (p < 0.05) [15].

### Quantitative Real Time PCR

Five proteins overexpressed in PA adapted 2 D gels were selected for further study to monitor changes at the mRNA level using quantitative real time PCR (qRT-PCR). Enzymatic lysis of cell wall material was performed by incubating freshly harvested cells in TE buffer containing 1 mg/mL lysozyme for five minutes at room temperature. Total RNA isolation of PA adapted cells and unadapted cells was achieved using Qiagen's RNeasy^® ^Mini Kit following the manufacturer's instructions. Contaminating genomic DNA was removed from the RNA sample using the RNase-Free DNase set (Qiagen). QuantiTect^® ^Reverse Transcription Kit (Qiagen) was then used to synthesize cDNA to be used for qRT-PCR. The cDNA template was subsequently diluted 10× in preparation for PCR. Primers utilized in this assay (listed in Table 1) were designed using gene specific sequences from *S*. Typhimurium LT2 obtained from *coli*BASE [16] and were designed to amplify ~100-base pair fragments from each gene. The gene sequences obtained from *coli*Base for the purpose of primer design are identical to those in *S*. Enteritidis (strain PT4). qRT-PCR was performed as a relative quantification run using the StepOne^™ ^Real-Time PCR System (Applied Biosystems) and SYBR green reagents for detection. Reaction mixtures were prepared as follows: 10 μL 2× SYBR Green PCR master mix (Applied Biosystems), 10 pmol/μL forward primer, 10 pmol/μL reverse primer, cDNA template (~10 ng), and autoclaved nuclease-free water up to 20 μL. Thermocycling conditions were as follows: 95°C for 10 min, followed by 40 cycles of 95°C for 15 sec, 60°C for one min. Runs for each target included a negative control without target cDNA as well as a reaction for the 16 s rRNA as a reference gene. qRT-PCR data was analyzed using StepOne™software (Applied Biosystems). The reproducibility of the qRT-PCR reaction was confirmed by running independent reactions from independently grown cultures. All runs were performed five times and the results for each gene were averaged.

**Table 1:** Primers used in this study

Primer name	Sequence (5'→3')	Amplification target
CpxR-F Long	CCAGCACTTCCTGGCTTAAATGTTCACGGG	*cpxR *in qRT-PCR
CpxR-R Long	GGCCAAACGCTGGAGCTGACCGGTACGG	*cpxR *in qRT-PCR
SodA-F Long	GGTCACGATCCACGTTATGCCAAACTGAGCG	*sodA *in qRT-PCR
SodA-R Long	GATCGGGAAGCCGGAAGCGCCGGAAATGG	*sodA *in qRT-PCR
rplF-F Long	GGTCAACCGGGTGAGAGAAACCTAAAGACAGG	*rplF *in qRT-PCR
rplF-R Long	GGCTTCACTAAGAAGCTGCAGCTGGTTGG	*rplF *in qRT-PCR
rplE-F Long	GGGAAGATGATCTGCTCACGGACACCCATGC	*rplE *in qRT-PCR
rplE-R Long	GCCTGATCACTATTGCTGTTCCTCGTATCCG	*rplE *in qRT-PCR
dps-F Long	CGGCCAGTTCTTTTAAGTGATCCTGCACG	*dps *in qRT-PCR
dps-R Long	GGCGTTAGGCACCACGCAAGTTATCAACAGC	*dps *in qRT-PCR
16 s rRNA-FP	GGTAGCCAACTGCTGCTGTCT	*16 S rRNA *in qRT-PCR
16 s rRNA-RP	CCGCAGCCGACATCCA	*16 S rRNA *in qRT-PCR
CpxR-P1	GTTGATGATGACCGAGAGCTGACTTCCCTGTGTGTA GGCTGGAGCTGCTT	Km^R ^gene from pKD4; specific for deletion of *cpxR*
CpxR-P2	AGCGGAAACCATCAGATAGCCGCGACCACGCATATG AATATCCTCCTTAG	Km^R ^gene from pKD4; specific for deletion of *cpxR*
CpxR-UpF	CTGCTGACGCTGATGTTCGG	region upstream of *cpxR*
CpXR-UpR	CAGGGAAGTCAGCTCTCGGTC	region upstream of *cpxR*
CpxR-DwnF	CGTGGTCGCGGCTATCTGATGG	region downstream of *cpxR*
CpxR-DwnR	GCGGATGATCGGCGTTATCCGC	region downstream of *cpxR*
dps-P1	TGCTTTATACCCGTAACGATGTATCAGAGAGCGTGT GTAGGCTGGAGCTGCTT	Km^R ^gene from pKD4; specific for deletion of *dps*
dps-P2	AGGTCGCGTGATGCGGCGGTAAAGATATCGGCCATA TGAATATCCTCCTTAG	Km^R ^gene from pKD4; specific for deletion of *dps*
dps-UpF	CTGACCAGCATCGTGACAATGAGC	region upstream of *dps*
dps-UpR	CGCTCTCTGATACATCGTTACGG	region upstream of *dps*
dps-DwnF	GCCGATATCTTTACCGCCGC	region downstream of *dps*
dps-DwnR	GGGCAAAACCAGTATGCCGCACC	region downstream of *dps*

### Deletion mutant construction

Knockout *S*. Enteritidis LK5 mutants harboring deletions in either *dps *or *cpxR *were made using an overlapping PCR extension protocol and the Red recombination system [17,18]. Primers used to create deletion cassette are listed in Table1. KOD DNA polymerase (EMD Chemicals Inc.) was utilized to ensure blunt-ended PCR products with no residual nucleotide overhang. For each gene deletion, P1 (forward) and P2 (reverse) primers were used to amplify the kanamycin resistance cassette from plasmid pKD4 [17]. These primers were made specific for the gene to be deleted by adding gene specific 30 bp flanking sequence to the 5' end of the of both P1 and P2 primers (30 bp from the outermost 5' end of the gene targeted for deletion was added to P1 while 30 bp from the outermost 3' end of said gene was added to P2). The resultant PCR product -the kanamycin resistance cassette with the extreme 5' and 3' ends of the gene that was to be deleted-was the first of three templates necessary for construction of the deletion cassette. The second and third templates for the overlapping PCR extension were PCR products of the immediate up and downstream regions (300-500 bp) of the targeted gene; amplified from *S*. Enteritidis LK5 genomic DNA using "up" and "down" primers specific for the target. A final PCR reaction was performed to create the deletion cassette (total length 2.2 - 2.3 kb). Template DNA for this reaction consisted of the aforementioned PCR products (the upstream region of the gene to be deleted, the kanamycin resistance cassette, and the downstream region of the gene to be deleted). Joining of the three templates during the final PCR reaction was made possible by the 30 bp extensions added to the 5' end of the P1 and P2 primers.

The deletion cassette was incorporated into the genome via the λ Red recombinase method previously described by Datsenko & Wanner, 2000. Deletion mutants were selected for on LB plates containing kanamycin. Deletion of the target genes was initially confirmed by colony PCR and ultimately by sequencing. pKD46 was cured from the resulting deletion mutants by overnight growth at 37°C. Finally, isogenic strains were constructed in a fresh background for each knock-out strain by P22 HT *int*- mediated transduction of the Δ*dps*::Kan and Δ*cpxR*::Kan mutations into wild type *S*. Enteritidis LK5.

### Acid resistance studies

For measuring acid resistance, 10 μl of a PA adapted culture for each strain (WT, *∆cpxR*, and *∆dps*) was transferred to 2 ml of LB broth (pH 3.0) acidified with 1 M HCl and incubated for 1 hour without shaking. One hundred microliter samples of the challenge culture were taken at time zero, and 1 hour post inoculation, serially diluted and plated for enumeration on LB plates. An unadapted *S*. Enteritidis strain (adapted in unsupplemented LB broth) served as a negative control and was tested for resistance to acid as well. The CFU/ml of each challenge culture was calculated and the percent survival of the PA adapted and control cultures were determined using the following formula

$$\%\text{ survival at time 1 hour}=\left(\text{CFU per ml at 1 hour}/{\text{CFU per ml at time}}_{\text{o}}\right)\times \text{1}00$$

All challenge assays were performed in triplicate and the presented results represent an average of each strain.

### Complementation of S. Enteritidis LK5 Δdps and S. Enteritidis LK5 ΔcpxR deletion mutants

Complementation studies were performed in order to confirm that the observed phenotype of the mutants was not due to a polar effect of the deletion. The coding region of *dps *and *cpxR *were both individually amplified from the genome of *S*. Enteritidis LK5, cloned into the *Xba*I site of pUC19 for expression from the *lacZ *promoter, and finally electroporated in to *E. coli *TOP10. To confirm genetic complementation, pUC19 plasmids were isolated from transformants and sequenced to verify presence of the cloned target gene. Each mutant, *S*. Enteritidis Δ*dps *and *S*. Enteritidis Δ*cpxR*, was then transformed with pUC19 carrying the respective gene. Plasmids were transformed into *Salmonella *by electroporation and selected for on LB plates containing ampicillin. The two complemented strains were then subjected to an acid resistance assay as previously described.

### Statistical methods

The data reported for acid resistance studies and complementation studies are the average values from three independent trials. Data reported for qRT-PCR runs were the average of five independent trials. All data was analyzed using the Student's *t*-test and *P *values <0.05 were considered to be significant.

## Results

Previously, SCFA adaptation of *Salmonella *was performed for a relatively short period (~1 hour) at a neutral pH prior to acid challenge [5]. However, exposure of *Salmonella *to PA is most likely to be long term (> 1 hour) in natural settings and infecting salmonellae are likely to have reached stationary phase during adaptation. Also, the fact that the typical pH range of the mammalian gut lies between 6 and 7 suggests that meaningful PA adaptation be performed at a neutral or near neutral pH since these environments serve as a major source of PA exposure [8]. We determined that it may be more informative to explore PA induced genetic and proteomic variances in *S*. Enteritidis within an environmental and/or growth condition which more closely mimics that of real world PA exposure. However, it was first necessary to correlate long term PA adaptation with the induction of protective responses similar to that observed with short term adaptation.

### PA-induced acid resistance

**Figure 1:**
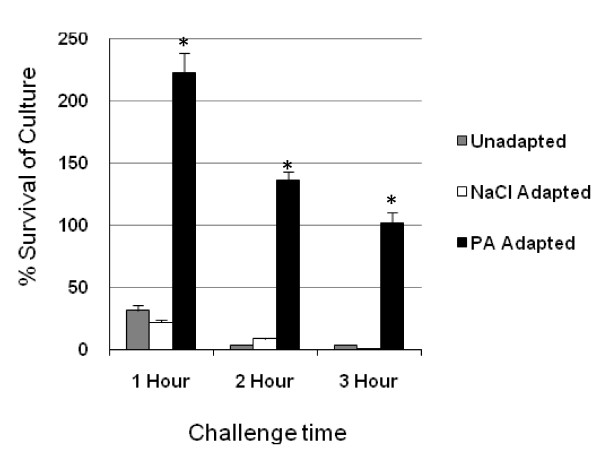
**Acid challenge of PA adapted and unadapted *S. Enteritidis***. Graph illustrates the percent survival of PA adapted, NaCl adapted, and unadapted *S*. Enteritidis LK5 cultures. All cultures were adapted for 16 hours and subsequently challenged over a three hour period to a highly acidic medium (pH 3.0). Acid resistance was determined by calculating the overall percent survival of each culture following acid exposure. Presented data is the average of three independent trials. Standard error is represented by error bars. Conditions that are significantly different from the unadapted condition with respect to acid resistance are indicated with an asterisk.

*S*. Enteritidis LK5 was adapted at a neutral pH in the presence of 100 mM PA for 16 hours and subsequently subjected to a highly acidic environment (pH 3.0). It should be mentioned that the difference in cell density between control and PA adapted cultures following overnight incubation was not statistically significant (*p *> 0.1). Unadapted *S*. Enteritidis cultures (grown in unsupplemented LB broth) and *S*. Enteritidis adapted using 100 mM NaCl were used as negative controls to determine the ability of the bacterium to survive acid stress without prior exposure to PA. LB containing NaCl was employed as a negative control because NaOH was utilized to adjust the pH of media containing PA. Therefore, the sodium ions present in both the control and experimental media were eliminated as an augmenting factor in the induction of stress resistance. PA adapted *S*. Enteritidis showed a much higher rate of survival during exposure to pH 3.0 than control bacteria over the three-hour period (Figure 1). Within the first hour of exposure to the highly acidic medium, the PA adapted culture (initial cell density 10^6 ^CFU/mL) more than doubled in numbers (223%). However, the number of viable adapted cells reduced thereafter and by three hours post-inoculation, cell numbers had reached their initial level (~100%). Lack of growth inhibition within PA adapted cultures in spite of acid shock is extremely suggestive of an induced acid resistant phenotype in response to PA exposure. Non-PA adapted bacterial populations (initial cell density 10^7 ^CFU/mL) showed no significant acid resistance during the three hour assay. Less than five percent remained viable after the third hour. The long term PA adaptation condition used in this study was able to induce intense acid resistance exceeding that following short term adaptation during exponential phase that has been previously reported [2,5]. Therefore, we deemed it most appropriate for subsequent 2 D gel experiments in which the proteomic changes of PA adapted *S*. Enteritidis were to be scrutinized.

### Two dimensional gel electrophoresis

**Figure 2:**
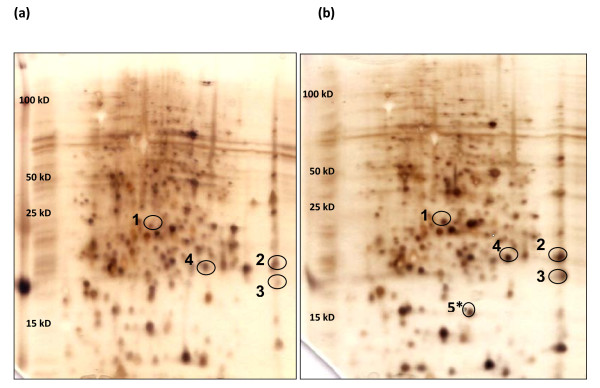
**2 D gel images of the soluble protein fractions from PA adapted and unadapted *S. Enteritidis cultures***. (a) Unadapted gel, (b) PA adapted gel. Proteins upregulated in PA gel selected for further examination are circled. Proteins restricted to PA adapted gels are designated with an asterisk (*) in gel (b). Labeled Proteins were identified as (1) CpxR, (2) RplE, (3) RplF, (4) SodA, (5) Dps.

The soluble proteins from PA adapted and unadapted cultures were visualized by 2 D gel electrophoresis (Figure 2). Because our objective was to identify proteins that were upregulated in response to PA, we concentrated on spots that were solely detected (after silver staining) on PA adapted gels or those that showed significant overexpression in PA adapted gels. In all, a combined total of 207 proteins were detected and their expressions on PA adapted and unadapted gels (or lack thereof) were evaluated. Although the analysis software determined that the differential expression of twenty-four spots was statistically significant (*p *< 0.05), we focused our attention on five spots (RplE, RplF, SodA, Dps and CpxR; Table 2) with pronounced overexpression in PA adapted gels and targeted them for identification. With respect to the overexpression of RplE and RplF in PA adapted gels, it should be noted that in general, the spot variances of basic proteins separated by 2 D gel electrophoresis have a low confidence level when a comprehensive analysis of total soluble proteins is intended. However, the results of 2 D gel experiments in this study were highly reproducible. Therefore, it is the opinion of the authors that these proteins were truly overexpressed following long-term PA exposure. The data obtained and the reproducibility of the presented gels support this notion.

**Table 2:** Proteins identified in PA adapted gels by PMF, MS/MS

Spot Number	Protein Name	Protein Description [Origin Species selected by MASCOT]	Fold Change	*p value*	Mascot Score	Peptides Matched	Molecular Weight (Da)
1	CpxR	DNA-Binding transcriptional regulator [*Shigella flexneri *5 str. 8401]	+5.01	0.02136	185	11	26274
2	RplE	50 S ribosomal subunit protein L5 [*Salmonella enterica *serovar Typhi str. CT18]	+5.84	0.03998	85	8	20362
3	RplF	50 S ribosomal subunit protein L6 [*Salmonella enterica *serovar Typhi str. CT18]	+6.09	0.04065	177	7	18905
4	SodA	Manganese superoxide dismutase [*Escherichia coli *O157:H7]	+7.51	0.01953	155	5	22886
5	Dps*	starvation/stationary phase DNA protection protein [*Salmonella enterica *serovar Typhi str. CT18]	-	-	482	12	18706

Table 2. Proteins in Table 2 are those with the highest and most statistically significant changes in protein expression following exposure to PA. Fold change is the level of change of each protein following PA adaptation. A Student's *t *test (performed by Melanie 5.0 gel analysis software) was used to determine the level of significance of expression values.

*As Dps was not detected by Melanie 5.0 in the unadapted control gels (for unknown reasons), no fold change or *p *value for this protein can be reported. This protein was selected for further study because of its prominence in PA adapted gels.

### Mass Spectrometry

Among the proteins identified were the 50 S rRNA-binding proteins RplE (an essential protein for cell viability in *E. coli*) and RplF (a protein associated with gentamycin and fusidic acid resistance) [19-21] (Additional Files 1 and 2, respectively). Manganese superoxide dismutase (SodA, Additional File 3)-also upregulated by PA- is an enzyme that catalyzes the dismutation of superoxide into oxygen and hydrogen peroxide and represents an important line of antioxidant defense in nearly all cells exposed to oxygen.

Of the identified proteins, CpxR and Dps (Additional File 4) are those commonly associated with stress resistance. CpxR is part of the two-component regulatory system CpxAR which controls gene expression in response to numerous external stimuli, including those responsible for alterations in the cell envelope [22-25]. The DNA-binding protein (Dps) has shown an ability to protect several pathogenic bacteria during acid stress, as well as when subjected to various oxidative stresses [26-30]. It is produced primarily throughout stationary phase and its expression is regulated by the stationary phase sigma factor RpoS (σ^38^), OxyR, and IHF [31]. Dps sequesters iron, thereby limiting Fenton-catalyzed oxyradical formation, and also physically protects DNA against environmental assaults by sequestering it into a highly stable biocrystal complex [32].

### Quantitative Real-time PCR

**Figure 3:**
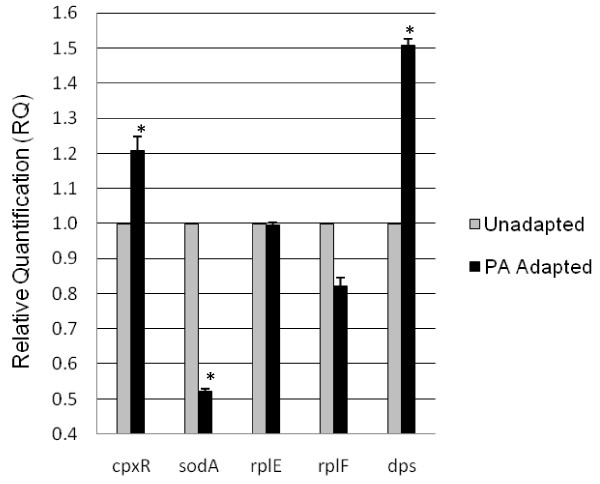
**qRT-PCR monitoring the expression of selected genes from PA adapted and unadapted cultures**. The level of expression of each target gene in the PA adapted culture was compared to the level of gene expression of the identical target in the unadapted culture. The expression of each gene in unadapted cultures was taken to be the basal level of expression for that particular gene to which the expression in PA adapted cultures was compared, therefore allowing quantification of the relative changes in gene expression of selected targets. The relative quantification (RQ) of each target gene was subsequently calculated from the qRT-PCR data using the comparative C_T _(ΔΔC_T_) method. All data obtained from qRT-PCR experiments were normalized using 16 s rRNA. Presented data is the average of five independent trials. Standard error is represented by error bars. Genes with expression that is significantly different from the unadapted condition are indicated with an asterisk.

Quantitative real-time PCR was performed to determine if the proteins upregulated in PA cultures (Dps, CpxR, SodA, RplE, and RplF) were overexpressed at the transcriptional level as well. A relative quantification experiment was performed; therefore, the level of expression of each target in the PA adapted culture was compared to the level of gene expression of the identical target gene in the unadapted culture. The expression of each gene in unadapted cultures was taken to be the basal level of expression for that particular gene (for the growth conditions used in this study) to which the expression in PA adapted cultures was compared. This method allowed the changes in gene expression of our selected targets to be carefully quantified. The relative quantification of each target gene was calculated from the data obtained using the comparative C_T _(ΔΔC_T_) method. Interestingly, qRT-PCR results did not fully coincided with all of the previously obtained proteomic results from 2 D electrophoresis (Figure 3). When compared to unadapted cultures, only two of the five targets overexpressed at the proteomic level (Dps and CpxR) showed increased expression at the transcriptional level (p < 0.05). *cpxR *showed a 20.8% increase in expression in PA adapted cultures, while *dps *from PA adapted cultures showed a 50.7% increase in expression over that from unadapted cultures. Expression of *rplE *and *rplF *in PA adapted cultures was only 82.1% and 99.5% respectively, of those from unadapted cultures. This difference in gene expression was not statistically significant (*p *> 0.05). Finally, *sodA *showed a significant decrease in expression after exposure to PA (*p *< 0.01). Its expression in PA adapted cultures was only 52.2% of that in unadapted cultures. Our combined results from 2D-gel and qRT-PCR experiments suggested that overexpression of *cpxR *and *dps *occurs at the transcriptional level, while *rplE*, *rplF*, and *sodA *were most likely upregulated post-translationally by an unidentified regulatory mechanism.

### Acid challenge and genetic complementation of *cpxR *and *dps *deletion mutants

**Figure 4:**
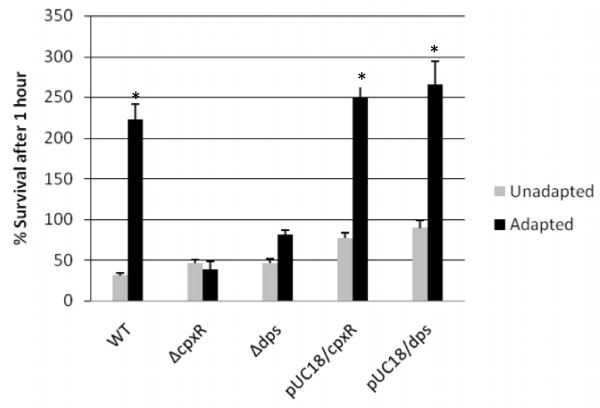
**Acid challenge of *S. Enteritidis cpxR and dps deletion mutants***. Wild type *S*. Enteritidis, *S*. Enteritidis Δ*cpxR*, *S*. Enteritidis *Δdps*, and both genetically complemented mutants were challenged to a highly acidic environment following PA adaptation. Unadapted and PA adapted cultures were challenged for one hour in LB broth (pH 3.0). Acid resistance was determined by calculating the overall percent survival of each culture following acid exposure. Presented data is the average of three independent trials. Standard error is represented by error bars. Acid resistant phenotypes that differ significantly from the unadapted condition are indicated with an asterisk.

To better understand PA-induced acid resistance, we assessed the significance of Dps and CpxR in the observed acid resistant phenotype of *S*. Enteritidis. These proteins were the focus of subsequent studies due to their common association with virulence in *Salmonella*. With our initial studies, we were able to show that long term PA adaptation of *S*. Enteritidis was tightly correlated with a remarkable increase in acid resistance over unadapted cultures. It was therefore reasoned that these stress-related proteins may be important for PA-induced acid resistance in *S*. Enteritidis as well. Unadapted and PA adapted cultures were prepared using the *cpxR *and *dps *mutant strains, subcultured in LB broth (pH 3.0). The percent survival for each PA adapted and unadapted culture is shown in Figure 4. After PA adaptation, wild type *S*. Enteritidis was able to withstand the highly acidic environment and even thrive after one hour. In fact, the percent survival for this culture was well above 220% at the study's endpoint. The unadapted wild type culture, however, demonstrated a poor ability to survive in this highly acidic medium, with only 31.4% of the culture remaining viable after one hour. Both deletion mutants experienced a dramatic loss in acid resistance induced by long term exposure to PA when compared to wild type *S*. Enteritidis. PA adaptation proved to be inconsequential in the *cpxR *mutant. In this case, the PA adapted *cpxR *mutant performed on the same level as the unadapted mutant with percent survivals of 38.3% and 46.14%, respectively, after one hour. The PA adapted *dps *mutant fared slightly better and outperformed the unadapted *dps *mutant by nearly 35%. However, the adapted *dps *mutant was still highly susceptible to acid with only 81% of the culture surviving after one hour. Although the *dps *deletion mutant was able to show a slightly elevated resistance to acid following PA adaptation, the induced acid resistance was highly diminished compared to that observed in wild type cultures following PA adaptation. Acid resistance was fully restored to both the *cpxR *and *dps *mutants following genetic complementation. When adapted in the presence of PA, the percent survival of these cultures surpassed the wild type by at least thirty percentage points; perhaps due to overexpression of the proteins from the high-copy number expression vector pUC19. However, unadapted complemented mutants still performed at a level much lower than that of PA adapted *S*. Enteritidis.

## Discussion

In *S*. Enteritidis, PA exposure has been correlated with the induction of a dramatic protective response to extreme acidic conditions and has also displayed the capacity to confer cross protection against other potentially bactericidal stresses. It has also been demonstrated that acid resistance following long term exposure to PA is actually greater than that induced after short term exposure and that this resistance is significantly enhanced with adaptation time [33]. PA has a pK-value of 4.88 and like other weak acids it can shuttle protons into the cell, thereby triggering the induction of an acid response. Consequently, it can only be expected that PA exposure would be associated with changes in gene expression and *de novo *protein synthesis, ultimately leading to profound differences in the transciptome and proteome of this pathogen. In this work, we closely examined the proteome of *S*. Enteritidis following long term exposure to PA and compared it to that of unadapted *S*. Enteritidis in order to monitor protein changes that may occur in direct response to PA. PA was able to induce the differential expression of over twenty proteins; the most statistically significant of which were identified as Dps, CpxR, RplE, RplF, and SodA. Excluding Dps, whose detection was solely restricted to PA adapted gels, all identified proteins were highly overexpressed in PA adapted gels. That is not to say that Dps was missing from unadapted cultures; in all likelihood, it was present. Dps is initially synthesized upon the cessation of growth and continues to accumulate even after several days of starvation [26]. A maximum concentration of Dps is reached after three days, after which time it becomes the most abundant protein in the cell [26,34]. However, at the time of protein harvest in this study (16 hours post inoculation), its overall abundance in unadapted cultures was extremely low (when compared to that within adapted cultures) and, in all probability, under the detection limit for silver staining.

PA exposure has been correlated with *de novo *protein synthesis [5]; therefore, the observed increase in abundance of ribosomal proteins in this study is not surprising. Specifically, this study establishes a direct link between PA exposure and the overexpression of ribosomal proteins. The 50 S ribosomal proteins RplE and RplF (both components of the *spc *operon) have not been studied in abundance in *Salmonella*. However, it is known that the synthesis of ribosomal proteins fluctuates in accordance to the cell's environment [35]. RplE was discovered to be crucial for cell viability in *E. coli *[20]. Knockout mutants lacking this gene were unable to compensate for the loss *in vitro *and its absence ultimately proved to be lethal. It is quite possible that RplE may play a similar role in *S*. Enteritidis; however, this hypothesis has yet to be tested in *Salmonella*. It is certain the abundance of these ribosomal proteins in PA adapted cultures serves a purpose; however, this and other hypotheses must be tested to gain insight into their role in PA adapted cultures before further speculation can be made. Of the five proteins overexpressed in PA adapted cultures, Dps and CpxR are those normally associated with virulence and pathogenesis in *Salmonella *and other enteropathogenic bacteria [28,36]. Interestingly, these are also the only two proteins over-expressed at the mRNA level as well. The fact that RplE, RplF, and SodA were either suppressed (*sodA *and *rplF*) or unaffected (*rplE*) at the transcriptional level, yet overexpressed at the translational level is not highly unusual. In fact, studies comparing mRNA and protein abundances has demonstrated that, in general, the amount of mRNA levels in a cell at a given instance shows no correlation with the amount of protein that is produced by the cell [37,38].

A potential mechanism for regulation of Dps in response to prolonged PA exposure may stem from the fact that this protein is translationally regulated by the RNA-binding protein Hfq during stationary phase [38] and that expression of Dps is reduced in an Hfq deletion mutant during this time. (Expression of RplF is also reduced in an Hfq mutant; however, this expression pattern is specific to growth in acidified minimal medium.) PA exposure may increase the expression of Hfq during stationary phase and ultimately result in increased translation of Dps. Additionally, an interesting aspect with regards to RplE expression during stationary phase and Hfq-dependent regulation can be pointed out. Although RplE was not specifically found to be translationally regulated by Hfq during stationary phase, the fact that Hfq putatively binds RplE [39] and that RplE is among those proteins overexpressed in response to PA during stationary phase suggests that Hfq may also positively regulate RplE expression in some fashion following PA exposure during stationary phase.

Dps, a DNA-binding protein normally associated with stationary phase or starved cells, was highly overexpressed in PA adapted cultures. The upregulation of this particular protein is of no surprise, as expression of Dps is known to be upregulated in response to other *in vivo *mimicking environments [40]. The extended adaptation time utilized in this study (16 hours) was well into stationary phase. However, Dps was undetectable in second dimension PAGE gels from unadapted cultures, which were well into stationary phase at the time of protein harvest as well. Although it is certain that unadapted cultures contain Dps (as confirmed by our qRT-PCR results), the combined results of our assays provide evidence that this protein was overexpressed in PA adapted cultures as a result of prolonged PA exposure, not because the cells' entry into a starved state, or stationary phase. Results of our acid challenge studies also suggest a major role of Dps in PA-induced acid resistance in *S*. Enteritidis. Unlike the wild type, *S*. Enteritidis ∆*dps *was highly susceptible to acid, even when subjected to prolonged PA adaptation prior to acid stress. A previous study has determined that Dps protects *E. coli *O157:H7 via direct interaction with DNA under acidic conditions [27]. It is highly probable that protection from acid shock is afforded to *S*. Enteritidis in a similar manner. The combined results of our genetic, proteomic, and acid stress studies confirm that CpxR is highly overexpressed in PA adapted cultures (when compared to the level of expression in unadapted cultures) and is required for induction of acid resistance in *S*. Enteritidis following long term PA adaptation. *cpxRA *is a two component regulatory system that controls the expression of several genes in response to environmental stimuli [22,24,25]. CpxA is a histidine kinase sensor, while CpxR serves as its cognate response regulator. This regulon, commonly associated with virulence in several gram-negative bacteria, was previously thought to be an essential part of the *Salmonella *starvation-stress response [41]. It is tempting to assume our specific results (overexpression of CpxR) were obtained because the extended period of adaptation sent the cells into a state of starvation and that exposure to PA only augmented the starved state by introducing a sublethal stress. However, carbon starvation does not generate the signals necessary for full induction of the *cpx *regulon [41]. When coupled with the fact that overexpression of CpxR was only observed in PA adapted cells, we are confident in inferring that CpxR was overexpressed as a result of PA exposure. Although we did not identify CpxA as an overexpressed protein in PA adapted cultures, it is a common belief that a sensor kinase and its coupled response regulator normally function in a concerted manner to control the expression of targeted genes [41]. It has also been suggested that the two components of this particular regulatory system do not always act in tandem specifically in response to acid stress. From the results obtained in this study, we cannot speculate on the overexpression of CpxA in PA adapted cultures-as CpxA is a membrane localized protein and this study focused on soluble proteins. It may be informative, however, to examine the expression profile of CpxA in PA adapted cultures in order to decipher if CpxR works in a concerted manner with CpxA to protect cells from acid stress following the onset of PA-induced acid resistance.

## Conclusion

It is apparent that long term PA adaptation of *S*. Enteritidis is associated with differential protein expression, with the synthesis of certain proteins being significantly upregulated. Of these proteins, Dps and CpxR are those commonly associated with virulence and we have not only demonstrated that they are inducible by PA, but also that they are crucial for PA-induced acid resistance in *S*. Enteritidis. These results clearly demonstrate that Dps and CpxR play an important role in PA-induced acid resistance. It is also apparent that overexpression of either Dps or CpxR alone in PA adapted cultures is not sufficient to confer increased acid resistance.

## Authors' contributions

LNC carried out the molecular and genetic studies, conducted the 2 D gel electrophoresis studies and drafted the manuscript. RL performed all mass spec studies and protein identifications and reviewed the manuscript. JOL contributed financially to the research and also participated in the manuscript review. YMK conceived the study, participated in its design and coordination and helped to draft the manuscript. All authors read and approved the final draft for submission.

## Supplementary Material

Additional file 1 **Protein Report C**. Mass spectrometry report for RplE Click here for file

Additional file 2 **Protein Report B**. Mass spectrometry report for RplF Click here for file

Additional file 3 **Protein Report A**. Mass spectrometry report for SodA Click here for file

Additional file 4 **Protein Report D**. Mass spectrometry report for CpxR and Dps Click here for file
