# Supplementary material for: Proteomic analysis of Salmonella enterica serovar Enteritidis following propionate adaptation
Source: BMC Microbiol. 2010 Sep 28;10:249. doi: 10.1186/1471-2180-10-249 (PMC2957393; doi:10.1186/1471-2180-10-249)
Supplement: Additional file 1 — Protein Report C. Mass spectrometry report for RplE [file 1471-2180-10-249-S3.PDF]

Mascot Search Results

User	:	
Email	:	rliyana@uark.edu
Search title	:	
MS data file	:	DATA.TXT
Database	:	NCBInr 20071202 (5678482 sequences; 1961803296 residues)
Taxonomy	:	Bacteria (Eubacteria) (2746213 sequences)
Timestamp	:	23 Dec 2008 at 04:37:09 GMT
Warning	:	A Peptide summary report will usually give a much clearer picture of MS/MS se
Top Score	:	85 for gi|16762855, 50S ribosomal protein L5 [Salmonella enterica subsp. ente

Probability Based Mowse Score

Protein score is -10*Log(P), where P is the probability that the observed match is a random event.
Protein scores greater than 77 are significant (p<0.05).
Protein scores are derived from ions scores as a non-probabilistic basis for ranking protein hits.

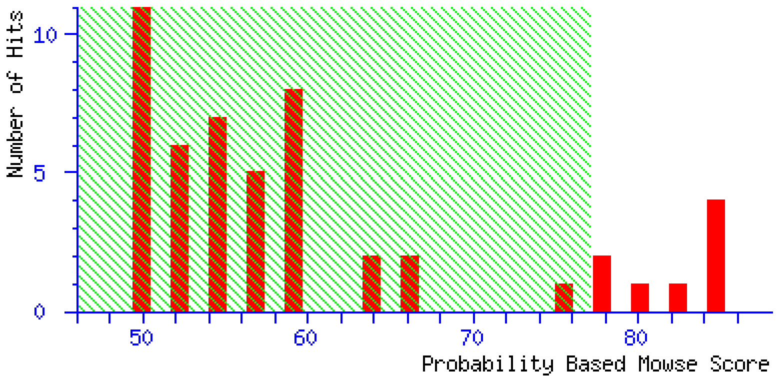

Protein Summary Report

Format As | Protein Summary (deprecated)

Help

Significance threshold p< 0.05 Max. number of hits 50

Standard scoring ◉ MudPIT scoring ○ Ions score or expect cut-off 0 Show sub-sets 0

Show pop-ups ◉ Suppress pop-ups ○ Sort unassigned Decreasing Score Require bold red ☐

Overview Table

Click on column header to jump to entry in results list.
Move mouse over any indicator to highlight identical peptides.
Click on an indicator to see details of individual match.
Use check boxes to select sub-set of queries for new search.

Mouse over: -Query-

-Accession- -Sequence-

Hit:	1	2	3	4	5	6	7	8	9	10	11	12	13	14	15	16	17	18	19	20	21	22	23	24	25	26	27	28	29	30	31	32	33	34	35
☑ 807.3826 (1+)																																			
☑ 827.4583 (1+)																																			
☑ 832.4761 (1+)																																			
☑ 856.5182 (1+)																										●							●		
☑ 870.5347 (1+)											●											●		●											●
☑ 882.5756 (1+)	●	●	●	●	●	●	●	●	●	●		●	●	●	●	●	●	●	●	●	●		●		●				●	●	●	●		●	
☑ 897.4091 (1+)																																			
☑ 973.5334 (1+)																																			
☑ 986.5831 (1+)																																			
☑ 993.5026 (1+)																																			
☑ 1004.5915 (1+)																																			
☑ 1016.5494 (1+)																																			
☑ 1026.5340 (1+)																																			

☑ 1029.5957 (1+)																																			
☑ 1033.5294 (1+)																																			
☑ 1037.5324 (1+)																																			
☑ 1053.5472 (1+)																																			
☑ 1055.6246 (1+)																																			
☑ 1057.5248 (1+)																																			
☑ 1060.5444 (1+)																																			
☑ 1066.5116 (1+)																																			
☑ 1074.5274 (1+)																																			
☑ 1090.5346 (1+)																																			
☑ 1092.4565 (1+)																																			
☑ 1107.5419 (1+)																																			
☑ 1109.4972 (1+)																																			
☑ 1111.5759 (1+)																																			
☑ 1122.5860 (1+)																																			
☑ 1126.5648 (1+)																																			
☑ 1132.6784 (1+)																																			
☑ 1140.5689 (1+)																																			
☑ 1150.6754 (1+)																																			
☑ 1152.6441 (1+)																																			
☑ 1157.5946 (1+)																																			
☑ 1169.6688 (1+)																																			
☑ 1184.6097 (1+)																																			
☑ 1193.6197 (1+)																																			
☑ 1198.6059 (1+)																																			
☑ 1201.6200 (1+)																																			
☑ 1212.5840 (1+)																																			

☑ 1228.6240 (1+)																																			
☑ 1230.5958 (1+)																																			
☑ 1234.6802 (1+)																																			
☑ 1262.6625 (1+)																																			
☑ 1263.7000 (1+)																																			
☑ 1265.6524 (1+)																																			
☑ 1267.6755 (1+)																																			
☑ 1277.7100 (1+)																																			
☑ 1290.6733 (1+)																																			
☑ 1307.6697 (1+)																																			
☑ 1308.6591 (1+)																																			
☑ 1314.7606 (1+)																																			
☑ 1320.5947 (1+)																																			
☑ 1323.6645 (1+)																																			
☑ 1329.6493 (1+)																																			
☑ 1338.6690 (1+)																																			
☑ 1341.6882 (1+)																																			
☑ 1357.7032 (1+)																																			
☑ 1365.6448 (1+)																																			
☑ 1373.6597 (1+)																																			
☑ 1379.7061 (1+)																																			
☑ 1383.6844 (1+)																																			
☑ 1390.6824 (1+)																																			
☑ 1407.7186 (1+)																																			
☑ 1424.7271 (1+)																																			
☑ 1427.7832 (1+)																																			
☑ 1434.7521 (1+)																																			

☑ 1438.6321 (1+)																																			
☑ 1458.7141 (1+)																																			
☑ 1475.7546 (1+)																																			
☑ 1487.7422 (1+)																																			
☑ 1493.7259 (1+)																																			
☑ 1509.7278 (1+)																																			
☑ 1541.7313 (1+)																																			
☑ 1600.8150 (1+)																																			
☑ 1657.7770 (1+)																																			
☑ 1699.8072 (1+)																																			
☑ 1707.7382 (1+)																																			
☑ 1708.6910 (1+)																																			
☑ 1753.8091 (1+)																																			
☑ 1838.8954 (1+)																																			
☑ 1860.8362 (1+)																																			
☑ 1879.9133 (1+)																																			
☑ 1940.8932 (1+)																																			
☑ 2022.8621 (1+)																																			
☑ 2087.9259 (1+)																																			
☑ 2225.0667 (1+)																																			
☑ 2239.0815 (1+)																																			
☑ 2285.9290 (1+)																																			
☑ 2383.9100 (1+)																																			
☑ 2398.9692 (1+)																																			
☑ 2705.1276 (1+)																																			
☑ 3312.3547 (1+)																																			

Select All | Select None | Search Selected

Index

	Accession	Mass	Score	Description
1.	gi|16762855	20362	85	50S ribosomal protein L5 [Salmonella enterica subsp. enterica
2.	gi|157148884	20332	85	hypothetical protein CKO_04722 [Citrobacter koseri ATCC BAA-89
3.	gi|15803835	20346	84	50S ribosomal protein L5 [Escherichia coli O157:H7 EDL933]
4.	gi|160867367	20376	84	hypothetical protein SARI_04201 [Salmonella enterica subsp. ar
5.	gi|24114586	20616	83	50S ribosomal protein L5 [Shigella flexneri 2a str. 301]
6.	gi|116667438	20144	80	Chain D, Structure Of The 50s Subunit Of A Pre-Translocational
7.	gi|124532473	21589	78	ribosomal protein L5 [Escherichia coli B]
8.	gi|33357905	20215	78	Chain D, Real Space Refined Coordinates Of The 50s Subunit Fit
9.	gi|156932247	20345	74	hypothetical protein ESA_00018 [Enterobacter sakazakii ATCC BA
10.	gi|86749427	20892	67	Ribosomal protein L5 [Rhodopseudomonas palustris HaA2]
11.	gi|31211267	19595	66	ShnG [Streptomyces hygroscopicus]
12.	gi|46129210	20355	63	COG0094: Ribosomal protein L5 [Haemophilus influenzae R2846]
13.	gi|15603268	20427	63	50S ribosomal protein L5 [Pasteurella multocida subsp. multoci
14.	gi|115525573	21078	60	ribosomal protein L5 [Rhodopseudomonas palustris BisA53]
15.	gi|152978153	20383	59	ribosomal protein L5 [Actinobacillus succinogenes 130Z]
16.	gi|33152942	20430	59	50S ribosomal protein L5 [Haemophilus ducreyi 35000HP]
17.	gi|52426091	20352	59	50S ribosomal protein L5 [Mannheimia succiniciproducens MBEL55
18.	gi|16120559	20308	59	50S ribosomal protein L5 [Yersinia pestis CO92]
19.	gi|77957319	20307	59	COG0094: Ribosomal protein L5 [Yersinia bercovieri ATCC 43970]
20.	gi|123444086	20331	59	50S ribosomal protein L5 [Yersinia enterocolitica subsp. enter
21.	gi|30995402	15932	58	hypothetical protein HI0790 [Haemophilus influenzae Rd KW20]
22.	gi|125716997	19863	58	50S ribosomal protein L5, putative [Streptococcus sanguinis SK
23.	gi|121591845	19566	58	ribosomal protein L5 [Vibrio cholerae 2740-80]
24.	gi|145620236	19918	57	ribosomal protein L5 [Geobacter bemidjiensis Bem]
25.	gi|15642579	20203	57	50S ribosomal protein L5 [Vibrio cholerae O1 biovar eltor str.
26.	gi|29653798	25907	57	adenylate kinase [Coxiella burnetii RSA 493]
27.	gi|154254005	68655	55	chaperone protein DnaK [Parvibaculum lavamentivorans DS-1]
28.	gi|127511486	7201	54	BFD domain protein (2Fe-2S)-binding domain protein [Shewanella
29.	gi|158424166	21110	54	ribosomal protein L5 [Azorhizobium caulinodans ORS 571]
30.	gi|113460221	20358	54	50S ribosomal protein L5 [Haemophilus somnus 129PT]
31.	gi|126209235	20399	54	50S ribosomal protein L5 [Actinobacillus pleuropneumoniae L20]

32.	gi|153094414	20328	54	ribosomal protein L5 [Mannheimia haemolytica PHL213]
33.	gi|117927621	50434	54	cell envelope-related transcriptional attenuator [Acidothermus
34.	gi|39936301	21139	52	50S ribosomal protein L5 [Rhodopseudomonas palustris CGA009]
35.	gi|23500984	121626	52	DNA polymerase III subunit alpha [Brucella suis 1330]
36.	gi|42522173	65055	52	histidine kinase [Bdellovibrio bacteriovorus HD100]
37.	gi|120401397	23486	52	transcriptional regulator, TetR family [Mycobacterium vanbaale
38.	gi|62289055	121631	52	DNA polymerase III subunit alpha [Brucella abortus biovar 1 st
39.	gi|120609238	23478	51	hypothetical protein Aave_0537 [Acidovorax avenae subsp. citru
40.	gi|116189194	9182	51	hypothetical protein VchoM_02001920 [Vibrio cholerae MO10]
41.	gi|23499837	45741	51	transglycosylase, putative [Brucella suis 1330]
42.	gi|62317029	46080	50	hypothetical transglycosylase [Brucella abortus biovar 1 str.
43.	gi|148558066	46110	50	putative transglycosylase [Brucella ovis ATCC 25840]
44.	gi|69247181	20177	50	Ribosomal protein L5 [Enterococcus faecium DO]
45.	gi|110635158	42059	50	beta-ketoadipyl CoA thiolase [Mesorhizobium sp. BNC1]
46.	gi|28493548	35163	50	FMN adenylyltransferase [Tropheryma whipplei str. Twist]
47.	gi|126358412	40266	50	putative iron-sulfur cluster binding protein [Pseudomonas puti
48.	gi|91977640	21075	50	ribosomal protein L5 [Rhodopseudomonas palustris BisB5]
49.	gi|17988368	46094	50	MEMBRANE-BOUND LYTIC MUREIN TRANSGLYCOSYLASE B [Brucella melit
50.	gi|85714293	17896	50	glutathione peroxidase [Nitrobacter sp. Nb-311A]

Results List

1. gi|16762855 **Mass:** 20362 **Score:** 85 **Expect:** 0.0093 **Queries matched:** 8

50S ribosomal protein L5 [Salmonella enterica subsp. enterica serovar Typhi str. CT18]

Observed	Mr(expt)	Mr(calc)	ppm	Start	End	Miss	Ions	Peptide
882.5756	881.5684	881.5698	-1.66	103 -	110	0	40	R.LITIAVPR.I
1037.5324	1036.5251	1036.5302	-4.86	116 -	125	1	---	R.GLSAKSFDGR.G
1184.6097	1183.6025	1183.5695	27.8	1 -	9	1	---	-.MAKLHDYYK.D + Oxidation
1267.6755	1266.6682	1266.6761	-6.20	168 -	178	0	---	R.ALLAAFDFPFR.K
1509.7278	1508.7205	1508.7399	-12.84	134 -	145	0	---	R.EQIIFPEIDYDK.V
1860.8362	1859.8289	1859.8546	-13.80	16 -	30	0	---	K.LMTEFNYNSVMQVPR.V + 2 Ox
1879.9133	1878.9060	1878.9363	-16.15	134 -	148	1	---	R.EQIIFPEIDYDKVDR.V
1940.8932	1939.8859	1939.9428	-29.33	162 -	178	1	---	K.SDEEGRALLAAFDFPFR.K

No match to: 807.3826, 827.4583, 832.4761, 856.5182, 870.5347, 897.4091, 973.5334, 986.5831, 993.5026, 1004.5915, 1016.5494, 1026.5340, 1029.5957, 1033.5294, 1053.5472,

1055.6246, 1057.5248, 1060.5444, 1066.5116, 1074.5274, 1090.5346, 1092.4565, 1107.5419, 1109.4972, 1111.5759, 1122.5860, 1126.5648, 1132.6784, 1140.5689, 1150.6754, 1152.6441, 1157.5946, 1169.6688, 1193.6197, 1198.6059, 1201.6200, 1212.5840, 1228.6240, 1230.5958, 1234.6802, 1262.6625, 1263.7000, 1265.6524, 1277.7100, 1290.6733, 1307.6697, 1308.6591, 1314.7606, 1320.5947, 1323.6645, 1329.6493, 1338.6690, 1341.6882, 1357.7032, 1365.6448, 1373.6597, 1379.7061, 1383.6844, 1390.6824, 1407.7186, 1424.7271, 1427.7832, 1434.7521, 1438.6321, 1458.7141, 1475.7546, 1487.7422, 1493.7259, 1541.7313, 1600.8150, 1657.7770, 1699.8072, 1707.7382, 1708.6910, 1753.8091, 1838.8954, 2022.8621, 2087.9259, 2225.0667, 2239.0815, 2285.9290, 2383.9100, 2398.9692, 2705.1276, 3312.3547

2. gi|157148884 **Mass:** 20332 **Score:** 85 **Expect:** 0.0093 **Queries matched:** 8

hypothetical protein CKO_04722 [Citrobacter koseri ATCC BAA-895]

Observed	Mr(expt)	Mr(calc)	ppm	Start	End	Miss	Ions	Peptide
882.5756	881.5684	881.5698	-1.66	103 -	110	0	40	R.LITIAVPR.I
1037.5324	1036.5251	1036.5302	-4.86	116 -	125	1	---	R.GLSAKSFDGR.G
1184.6097	1183.6025	1183.5695	27.8	1 -	9	1	---	-.MAKLHDYYK.D + Oxidation
1267.6755	1266.6682	1266.6761	-6.20	168 -	178	0	---	R.ALLAAFDFPFR.K
1509.7278	1508.7205	1508.7399	-12.84	134 -	145	0	---	R.EQIIFPEIDYDK.V
1860.8362	1859.8289	1859.8546	-13.80	16 -	30	0	---	K.LMTEFNYNSVMQVPR.V + 2 Ox
1879.9133	1878.9060	1878.9363	-16.15	134 -	148	1	---	R.EQIIFPEIDYDKVDR.V
1940.8932	1939.8859	1939.9428	-29.33	162 -	178	1	---	K.SDEEGRALLAAFDFPFR.K

No match to: 807.3826, 827.4583, 832.4761, 856.5182, 870.5347, 897.4091, 973.5334, 986.5831, 993.5026, 1004.5915, 1016.5494, 1026.5340, 1029.5957, 1033.5294, 1053.5472, 1055.6246, 1057.5248, 1060.5444, 1066.5116, 1074.5274, 1090.5346, 1092.4565, 1107.5419, 1109.4972, 1111.5759, 1122.5860, 1126.5648, 1132.6784, 1140.5689, 1150.6754, 1152.6441, 1157.5946, 1169.6688, 1193.6197, 1198.6059, 1201.6200, 1212.5840, 1228.6240, 1230.5958, 1234.6802, 1262.6625, 1263.7000, 1265.6524, 1277.7100, 1290.6733, 1307.6697, 1308.6591, 1314.7606, 1320.5947, 1323.6645, 1329.6493, 1338.6690, 1341.6882, 1357.7032, 1365.6448, 1373.6597, 1379.7061, 1383.6844, 1390.6824, 1407.7186, 1424.7271, 1427.7832, 1434.7521, 1438.6321, 1458.7141, 1475.7546, 1487.7422, 1493.7259, 1541.7313, 1600.8150, 1657.7770, 1699.8072, 1707.7382, 1708.6910, 1753.8091, 1838.8954, 2022.8621, 2087.9259, 2225.0667, 2239.0815, 2285.9290, 2383.9100, 2398.9692, 2705.1276, 3312.3547

3. gi|15803835 **Mass:** 20346 **Score:** 84 **Expect:** 0.011 **Queries matched:** 8

50S ribosomal protein L5 [Escherichia coli O157:H7 EDL933]

Observed	Mr(expt)	Mr(calc)	ppm	Start		End	Miss	Ions	Peptide
882.5756	881.5684	881.5698	-1.66	103	-	110	0	40	R.LITIAVPR.I
1037.5324	1036.5251	1036.5302	-4.86	116	-	125	1	---	R.GLSAKSFDGR.G
1184.6097	1183.6025	1183.5695	27.8	1	-	9	1	---	-.MAKLHDYYK.D + Oxidation
1267.6755	1266.6682	1266.6761	-6.20	168	-	178	0	---	R.ALLAAFDFPFR.K
1509.7278	1508.7205	1508.7399	-12.84	134	-	145	0	---	R.EQIIFPEIDYDK.V
1860.8362	1859.8289	1859.8546	-13.80	16	-	30	0	---	K.LMTEFNYNSVMQVPR.V + 2 Ox
1879.9133	1878.9060	1878.9363	-16.15	134	-	148	1	---	R.EQIIFPEIDYDKVDR.V
1940.8932	1939.8859	1939.9428	-29.33	162	-	178	1	---	K.SDEEGRALLAAFDFPFR.K

No match to: 807.3826, 827.4583, 832.4761, 856.5182, 870.5347, 897.4091, 973.5334, 986.5831, 993.5026, 1004.5915, 1016.5494, 1026.5340, 1029.5957, 1033.5294, 1053.5472, 1055.6246, 1057.5248, 1060.5444, 1066.5116, 1074.5274, 1090.5346, 1092.4565, 1107.5419, 1109.4972, 1111.5759, 1122.5860, 1126.5648, 1132.6784, 1140.5689, 1150.6754, 1152.6441, 1157.5946, 1169.6688, 1193.6197, 1198.6059, 1201.6200, 1212.5840, 1228.6240, 1230.5958, 1234.6802, 1262.6625, 1263.7000, 1265.6524, 1277.7100, 1290.6733, 1307.6697, 1308.6591, 1314.7606, 1320.5947, 1323.6645, 1329.6493, 1338.6690, 1341.6882, 1357.7032, 1365.6448, 1373.6597, 1379.7061, 1383.6844, 1390.6824, 1407.7186, 1424.7271, 1427.7832, 1434.7521, 1438.6321, 1458.7141, 1475.7546, 1487.7422, 1493.7259, 1541.7313, 1600.8150, 1657.7770, 1699.8072, 1707.7382, 1708.6910, 1753.8091, 1838.8954, 2022.8621, 2087.9259, 2225.0667, 2239.0815, 2285.9290, 2383.9100, 2398.9692, 2705.1276, 3312.3547

4. gi|160867367 **Mass:** 20376 **Score:** 84 **Expect:** 0.011 **Queries matched:** 8

hypothetical protein SARI_04201 [Salmonella enterica subsp. arizonae serovar 62:z4,z23:--

Observed	Mr(expt)	Mr(calc)	ppm	Start		End	Miss	Ions	Peptide
882.5756	881.5684	881.5698	-1.66	103	-	110	0	40	R.LITIAVPR.I
1037.5324	1036.5251	1036.5302	-4.86	116	-	125	1	---	R.GLSAKSFDGR.G
1184.6097	1183.6025	1183.5695	27.8	1	-	9	1	---	-.MAKLHDYYK.D + Oxidation
1267.6755	1266.6682	1266.6761	-6.20	168	-	178	0	---	R.ALLAAFDFPFR.K
1509.7278	1508.7205	1508.7399	-12.84	134	-	145	0	---	R.EQIIFPEIDYDK.V
1860.8362	1859.8289	1859.8546	-13.80	16	-	30	0	---	K.LMTEFNYNSVMQVPR.V + 2 Ox
1879.9133	1878.9060	1878.9363	-16.15	134	-	148	1	---	R.EQIIFPEIDYDKVDR.V
1940.8932	1939.8859	1939.9428	-29.33	162	-	178	1	---	K.SDEEGRALLAAFDFPFR.K

No match to: 807.3826, 827.4583, 832.4761, 856.5182, 870.5347, 897.4091, 973.5334, 986.5831, 993.5026, 1004.5915, 1016.5494, 1026.5340, 1029.5957, 1033.5294, 1053.5472, 1055.6246, 1057.5248, 1060.5444, 1066.5116, 1074.5274, 1090.5346, 1092.4565, 1107.5419, 1109.4972, 1111.5759, 1122.5860, 1126.5648, 1132.6784, 1140.5689, 1150.6754, 1152.6441, 1157.5946, 1169.6688, 1193.6197, 1198.6059, 1201.6200, 1212.5840, 1228.6240, 1230.5958, 1234.6802, 1262.6625, 1263.7000, 1265.6524, 1277.7100, 1290.6733, 1307.6697, 1308.6591, 1314.7606, 1320.5947, 1323.6645, 1329.6493, 1338.6690, 1341.6882, 1357.7032, 1365.6448, 1373.6597, 1379.7061, 1383.6844, 1390.6824, 1407.7186, 1424.7271, 1427.7832, 1434.7521, 1438.6321, 1458.7141, 1475.7546, 1487.7422, 1493.7259, 1541.7313, 1600.8150, 1657.7770, 1699.8072, 1707.7382, 1708.6910, 1753.8091, 1838.8954, 2022.8621, 2087.9259, 2225.0667, 2239.0815, 2285.9290, 2383.9100, 2398.9692, 2705.1276, 3312.3547

5. gi|24114586 **Mass:** 20616 **Score:** 83 **Expect:** 0.013 **Queries matched:** 8

50S ribosomal protein L5 [Shigella flexneri 2a str. 301]

Observed	Mr(expt)	Mr(calc)	ppm	Start	End	Miss	Ions	Peptide
882.5756	881.5684	881.5698	-1.66	103 -	110	0	40	R.LITIAVPR.I
1037.5324	1036.5251	1036.5302	-4.86	116 -	125	1	---	R.GLSAKSFDGR.G
1184.6097	1183.6025	1183.5695	27.8	1 -	9	1	---	-.MAKLHDYYK.D + Oxidation
1267.6755	1266.6682	1266.6761	-6.20	168 -	178	0	---	R.ALLAAFDFPFR.K
1509.7278	1508.7205	1508.7399	-12.84	134 -	145	0	---	R.EQIIFPEIDYDK.V
1860.8362	1859.8289	1859.8546	-13.80	16 -	30	0	---	K.LMTEFNYNSVMQVPR.V + 2 Ox
1879.9133	1878.9060	1878.9363	-16.15	134 -	148	1	---	R.EQIIFPEIDYDKVDR.V
1940.8932	1939.8859	1939.9428	-29.33	162 -	178	1	---	K.SDEEGRALLAAFDFPFR.K

No match to: 807.3826, 827.4583, 832.4761, 856.5182, 870.5347, 897.4091, 973.5334, 986.5831, 993.5026, 1004.5915, 1016.5494, 1026.5340, 1029.5957, 1033.5294, 1053.5472, 1055.6246, 1057.5248, 1060.5444, 1066.5116, 1074.5274, 1090.5346, 1092.4565, 1107.5419, 1109.4972, 1111.5759, 1122.5860, 1126.5648, 1132.6784, 1140.5689, 1150.6754, 1152.6441, 1157.5946, 1169.6688, 1193.6197, 1198.6059, 1201.6200, 1212.5840, 1228.6240, 1230.5958, 1234.6802, 1262.6625, 1263.7000, 1265.6524, 1277.7100, 1290.6733, 1307.6697, 1308.6591, 1314.7606, 1320.5947, 1323.6645, 1329.6493, 1338.6690, 1341.6882, 1357.7032, 1365.6448, 1373.6597, 1379.7061, 1383.6844, 1390.6824, 1407.7186, 1424.7271, 1427.7832, 1434.7521, 1438.6321, 1458.7141, 1475.7546, 1487.7422, 1493.7259, 1541.7313, 1600.8150, 1657.7770, 1699.8072, 1707.7382, 1708.6910, 1753.8091, 1838.8954, 2022.8621, 2087.9259, 2225.0667, 2239.0815, 2285.9290, 2383.9100, 2398.9692, 2705.1276, 3312.3547

6. gi|116667438 **Mass:** 20144 **Score:** 80 **Expect:** 0.027 **Queries matched:** 7
Chain D, Structure Of The 50s Subunit Of A Pre-Translocational E. Coli Ribosome Obtained

Observed	Mr(expt)	Mr(calc)	ppm	Start	End	Miss	Ions	Peptide
882.5756	881.5684	881.5698	-1.66	101 -	108	0	40	R.LITIAVPR.I
1037.5324	1036.5251	1036.5302	-4.86	114 -	123	1	---	R.GLSAKSFDGR.G
1267.6755	1266.6682	1266.6761	-6.20	166 -	176	0	---	R.ALLAAFDFPFR.K
1509.7278	1508.7205	1508.7399	-12.84	132 -	143	0	---	R.EQIIFPEIDYDK.V
1860.8362	1859.8289	1859.8546	-13.80	14 -	28	0	---	K.LMTEFNYNSVMQVPR.V + 2 Ox
1879.9133	1878.9060	1878.9363	-16.15	132 -	146	1	---	R.EQIIFPEIDYDKVDR.V
1940.8932	1939.8859	1939.9428	-29.33	160 -	176	1	---	K.SDEEGRALLAAFDFPFR.K

No match to: 807.3826, 827.4583, 832.4761, 856.5182, 870.5347, 897.4091, 973.5334, 986.5831, 993.5026, 1004.5915, 1016.5494, 1026.5340, 1029.5957, 1033.5294, 1053.5472, 1055.6246, 1057.5248, 1060.5444, 1066.5116, 1074.5274, 1090.5346, 1092.4565, 1107.5419, 1109.4972, 1111.5759, 1122.5860, 1126.5648, 1132.6784, 1140.5689, 1150.6754, 1152.6441, 1157.5946, 1169.6688, 1184.6097, 1193.6197, 1198.6059, 1201.6200, 1212.5840, 1228.6240, 1230.5958, 1234.6802, 1262.6625, 1263.7000, 1265.6524, 1277.7100, 1290.6733, 1307.6697, 1308.6591, 1314.7606, 1320.5947, 1323.6645, 1329.6493, 1338.6690, 1341.6882, 1357.7032, 1365.6448, 1373.6597, 1379.7061, 1383.6844, 1390.6824, 1407.7186, 1424.7271, 1427.7832, 1434.7521, 1438.6321, 1458.7141, 1475.7546, 1487.7422, 1493.7259, 1541.7313, 1600.8150, 1657.7770, 1699.8072, 1707.7382, 1708.6910, 1753.8091, 1838.8954, 2022.8621, 2087.9259, 2225.0667, 2239.0815, 2285.9290, 2383.9100, 2398.9692, 2705.1276, 3312.3547

7. gi|124532473 **Mass:** 21589 **Score:** 78 **Expect:** 0.039 **Queries matched:** 7
ribosomal protein L5 [Escherichia coli B]

Observed	Mr(expt)	Mr(calc)	ppm	Start	End	Miss	Ions	Peptide
882.5756	881.5684	881.5698	-1.66	113 -	120	0	40	R.LITIAVPR.I
1037.5324	1036.5251	1036.5302	-4.86	126 -	135	1	---	R.GLSAKSFDGR.G
1267.6755	1266.6682	1266.6761	-6.20	178 -	188	0	---	R.ALLAAFDFPFR.K
1509.7278	1508.7205	1508.7399	-12.84	144 -	155	0	---	R.EQIIFPEIDYDK.V
1860.8362	1859.8289	1859.8546	-13.80	26 -	40	0	---	K.LMTEFNYNSVMQVPR.V + 2 Ox
1879.9133	1878.9060	1878.9363	-16.15	144 -	158	1	---	R.EQIIFPEIDYDKVDR.V
1940.8932	1939.8859	1939.9428	-29.33	172 -	188	1	---	K.SDEEGRALLAAFDFPFR.K

No match to: 807.3826, 827.4583, 832.4761, 856.5182, 870.5347, 897.4091, 973.5334, 986.5831, 993.5026, 1004.5915, 1016.5494, 1026.5340, 1029.5957, 1033.5294, 1053.5472,

1055.6246, 1057.5248, 1060.5444, 1066.5116, 1074.5274, 1090.5346, 1092.4565, 1107.5419, 1109.4972, 1111.5759, 1122.5860, 1126.5648, 1132.6784, 1140.5689, 1150.6754, 1152.6441, 1157.5946, 1169.6688, 1184.6097, 1193.6197, 1198.6059, 1201.6200, 1212.5840, 1228.6240, 1230.5958, 1234.6802, 1262.6625, 1263.7000, 1265.6524, 1277.7100, 1290.6733, 1307.6697, 1308.6591, 1314.7606, 1320.5947, 1323.6645, 1329.6493, 1338.6690, 1341.6882, 1357.7032, 1365.6448, 1373.6597, 1379.7061, 1383.6844, 1390.6824, 1407.7186, 1424.7271, 1427.7832, 1434.7521, 1438.6321, 1458.7141, 1475.7546, 1487.7422, 1493.7259, 1541.7313, 1600.8150, 1657.7770, 1699.8072, 1707.7382, 1708.6910, 1753.8091, 1838.8954, 2022.8621, 2087.9259, 2225.0667, 2239.0815, 2285.9290, 2383.9100, 2398.9692, 2705.1276, 3312.3547

8. gi|33357905 **Mass:** 20215 **Score:** 78 **Expect:** 0.039 **Queries matched:** 7
Chain D, Real Space Refined Coordinates Of The 50s Subunit Fitted Into The Low Resolution

Observed	Mr(expt)	Mr(calc)	ppm	Start	End	Miss	Ions	Peptide
882.5756	881.5684	881.5698	-1.66	102 -	109	0	40	R.LITIAVPR.I
1037.5324	1036.5251	1036.5302	-4.86	115 -	124	1	---	R.GLSAKSFDGR.G
1267.6755	1266.6682	1266.6761	-6.20	167 -	177	0	---	R.ALLAAFDFPFR.K
1509.7278	1508.7205	1508.7399	-12.84	133 -	144	0	---	R.EQIIFPEIDYDK.V
1860.8362	1859.8289	1859.8546	-13.80	15 -	29	0	---	K.LMTEFNYNSVMQVPR.V + 2 Ox
1879.9133	1878.9060	1878.9363	-16.15	133 -	147	1	---	R.EQIIFPEIDYDKVDR.V
1940.8932	1939.8859	1939.9428	-29.33	161 -	177	1	---	K.SDEEGRALLAAFDFPFR.K

No match to: 807.3826, 827.4583, 832.4761, 856.5182, 870.5347, 897.4091, 973.5334, 986.5831, 993.5026, 1004.5915, 1016.5494, 1026.5340, 1029.5957, 1033.5294, 1053.5472, 1055.6246, 1057.5248, 1060.5444, 1066.5116, 1074.5274, 1090.5346, 1092.4565, 1107.5419, 1109.4972, 1111.5759, 1122.5860, 1126.5648, 1132.6784, 1140.5689, 1150.6754, 1152.6441, 1157.5946, 1169.6688, 1184.6097, 1193.6197, 1198.6059, 1201.6200, 1212.5840, 1228.6240, 1230.5958, 1234.6802, 1262.6625, 1263.7000, 1265.6524, 1277.7100, 1290.6733, 1307.6697, 1308.6591, 1314.7606, 1320.5947, 1323.6645, 1329.6493, 1338.6690, 1341.6882, 1357.7032, 1365.6448, 1373.6597, 1379.7061, 1383.6844, 1390.6824, 1407.7186, 1424.7271, 1427.7832, 1434.7521, 1438.6321, 1458.7141, 1475.7546, 1487.7422, 1493.7259, 1541.7313, 1600.8150, 1657.7770, 1699.8072, 1707.7382, 1708.6910, 1753.8091, 1838.8954, 2022.8621, 2087.9259, 2225.0667, 2239.0815, 2285.9290, 2383.9100, 2398.9692, 2705.1276, 3312.3547

9. gi|156932247 **Mass:** 20345 **Score:** 74 **Expect:** 0.097 **Queries matched:** 7
hypothetical protein ESA_00018 [Enterobacter sakazakii ATCC BAA-894]

Observed	Mr(expt)	Mr(calc)	ppm	Start	End	Miss	Ions	Peptide
882.5756	881.5684	881.5698	-1.66	103 -	110	0	40	R.LITIAVPR.I
1037.5324	1036.5251	1036.5302	-4.86	116 -	125	1	---	R.GLSAKSFDGR.G
1184.6097	1183.6025	1183.5695	27.8	1 -	9	1	---	-.MAKLHDYYK.D + Oxidation
1267.6755	1266.6682	1266.6761	-6.20	168 -	178	0	---	R.ALLAAFDFPFR.K
1509.7278	1508.7205	1508.7399	-12.84	134 -	145	0	---	R.EQIIFPEIDYDK.V
1879.9133	1878.9060	1878.9363	-16.15	134 -	148	1	---	R.EQIIFPEIDYDKVDR.V
1940.8932	1939.8859	1939.9428	-29.33	162 -	178	1	---	K.SDEEGRALLAAFDFPFR.K

No match to: 807.3826, 827.4583, 832.4761, 856.5182, 870.5347, 897.4091, 973.5334, 986.5831, 993.5026, 1004.5915, 1016.5494, 1026.5340, 1029.5957, 1033.5294, 1053.5472, 1055.6246, 1057.5248, 1060.5444, 1066.5116, 1074.5274, 1090.5346, 1092.4565, 1107.5419, 1109.4972, 1111.5759, 1122.5860, 1126.5648, 1132.6784, 1140.5689, 1150.6754, 1152.6441, 1157.5946, 1169.6688, 1193.6197, 1198.6059, 1201.6200, 1212.5840, 1228.6240, 1230.5958, 1234.6802, 1262.6625, 1263.7000, 1265.6524, 1277.7100, 1290.6733, 1307.6697, 1308.6591, 1314.7606, 1320.5947, 1323.6645, 1329.6493, 1338.6690, 1341.6882, 1357.7032, 1365.6448, 1373.6597, 1379.7061, 1383.6844, 1390.6824, 1407.7186, 1424.7271, 1427.7832, 1434.7521, 1438.6321, 1458.7141, 1475.7546, 1487.7422, 1493.7259, 1541.7313, 1600.8150, 1657.7770, 1699.8072, 1707.7382, 1708.6910, 1753.8091, 1838.8954, 1860.8362, 2022.8621, 2087.9259, 2225.0667, 2239.0815, 2285.9290, 2383.9100, 2398.9692, 2705.1276, 3312.3547

10. gi|86749427 **Mass:** 20892 **Score:** 67 **Expect:** 0.54 **Queries matched:** 8
Ribosomal protein L5 [Rhodopseudomonas palustris HaA2]

Observed	Mr(expt)	Mr(calc)	ppm	Start	End	Miss	Ions	Peptide
882.5756	881.5684	881.5698	-1.66	109 -	116	0	33	R.LITVALPR.V
1037.5324	1036.5251	1036.5011	23.1	1 -	9	0	---	-.MAETAYVPR.L
1053.5472	1052.5399	1052.4961	41.6	1 -	9	0	---	-.MAETAYVPR.L + Oxidation
1090.5346	1089.5273	1089.5567	-26.94	122 -	131	1	---	R.GLNPKSFDGR.G
1267.6755	1266.6682	1266.6179	39.7	26 -	36	0	---	K.FGYGNVMQVPR.L
1424.7271	1423.7198	1423.7612	-29.11	174 -	185	1	---	R.ALLTAFNFPFRQ.-
1487.7422	1486.7350	1486.7449	-6.70	56 -	70	0	---	K.AEQAAADMALIAGQK.A
1600.8150	1599.8078	1599.8403	-20.31	40 -	54	1	---	K.VVLNMGVGEAVNDRK.K

No match to: 807.3826, 827.4583, 832.4761, 856.5182, 870.5347, 897.4091, 973.5334, 986.5831, 993.5026, 1004.5915, 1016.5494, 1026.5340, 1029.5957, 1033.5294, 1055.6246, 1057.5248, 1060.5444, 1066.5116, 1074.5274, 1092.4565, 1107.5419, 1109.4972, 1111.5759,

1122.5860, 1126.5648, 1132.6784, 1140.5689, 1150.6754, 1152.6441, 1157.5946, 1169.6688, 1184.6097, 1193.6197, 1198.6059, 1201.6200, 1212.5840, 1228.6240, 1230.5958, 1234.6802, 1262.6625, 1263.7000, 1265.6524, 1277.7100, 1290.6733, 1307.6697, 1308.6591, 1314.7606, 1320.5947, 1323.6645, 1329.6493, 1338.6690, 1341.6882, 1357.7032, 1365.6448, 1373.6597, 1379.7061, 1383.6844, 1390.6824, 1407.7186, 1427.7832, 1434.7521, 1438.6321, 1458.7141, 1475.7546, 1493.7259, 1509.7278, 1541.7313, 1657.7770, 1699.8072, 1707.7382, 1708.6910, 1753.8091, 1838.8954, 1860.8362, 1879.9133, 1940.8932, 2022.8621, 2087.9259, 2225.0667, 2239.0815, 2285.9290, 2383.9100, 2398.9692, 2705.1276, 3312.3547

11. gi|31211267 **Mass:** 19595 **Score:** 66 **Expect:** 0.64 **Queries matched:** 6

ShnG [Streptomyces hygroscopicus]

Observed	Mr(expt)	Mr(calc)	ppm	Start	End	Miss	Ions	Peptide
870.5347	869.5274	869.5334	-6.94	158 -	165	0	33	R.VSLVSPIR.T
897.4091	896.4018	896.4352	-37.22	96 -	103	1	---	R.AGKSYTDR.G
1004.5915	1003.5842	1003.5736	10.6	12 -	20	1	---	K.MATAEKLLK.S
1053.5472	1052.5399	1052.5866	-44.37	2 -	11	1	---	M.PLPSPAKESK.M
1184.6097	1183.6025	1183.6271	-20.78	1 -	11	1	---	-.MPLPSPAKESK.M
2705.1276	2704.1203	2704.2097	-33.05	124 -	145	1	---	R.EALAMDPQQWAYWRMCTHPASR.R

No match to: 807.3826, 827.4583, 832.4761, 856.5182, 882.5756, 973.5334, 986.5831, 993.5026, 1016.5494, 1026.5340, 1029.5957, 1033.5294, 1037.5324, 1055.6246, 1057.5248, 1060.5444, 1066.5116, 1074.5274, 1090.5346, 1092.4565, 1107.5419, 1109.4972, 1111.5759, 1122.5860, 1126.5648, 1132.6784, 1140.5689, 1150.6754, 1152.6441, 1157.5946, 1169.6688, 1193.6197, 1198.6059, 1201.6200, 1212.5840, 1228.6240, 1230.5958, 1234.6802, 1262.6625, 1263.7000, 1265.6524, 1267.6755, 1277.7100, 1290.6733, 1307.6697, 1308.6591, 1314.7606, 1320.5947, 1323.6645, 1329.6493, 1338.6690, 1341.6882, 1357.7032, 1365.6448, 1373.6597, 1379.7061, 1383.6844, 1390.6824, 1407.7186, 1424.7271, 1427.7832, 1434.7521, 1438.6321, 1458.7141, 1475.7546, 1487.7422, 1493.7259, 1509.7278, 1541.7313, 1600.8150, 1657.7770, 1699.8072, 1707.7382, 1708.6910, 1753.8091, 1838.8954, 1860.8362, 1879.9133, 1940.8932, 2022.8621, 2087.9259, 2225.0667, 2239.0815, 2285.9290, 2383.9100, 2398.9692, 3312.3547

12. gi|46129210 **Mass:** 20355 **Score:** 63 **Expect:** 1.3 **Queries matched:** 6

COG0094: Ribosomal protein L5 [Haemophilus influenzae R2846]

Observed	Mr(expt)	Mr(calc)	ppm	Start	End	Miss	Ions	Peptide
832.4761	831.4688	831.4273	50.0	24 -	30	0	---	K.SVMQVPR.I + Oxidation (M

882.5756	881.5684	881.5698	-1.66	103 -	110	0	40	R.LITIAVPR.I
1037.5324	1036.5251	1036.5302	-4.86	116 -	125	1	---	R.GLSAKSFDGR.G
1212.5840	1211.5767	1211.5757	0.81	1 -	9	1	---	-.MAKLHDYYR.D + Oxidation
1509.7278	1508.7205	1508.7399	-12.84	134 -	145	0	---	R.EQIIFPEIDYDK.V
1879.9133	1878.9060	1878.9363	-16.15	134 -	148	1	---	R.EQIIFPEIDYDKVDR.V

No match to: 807.3826, 827.4583, 856.5182, 870.5347, 897.4091, 973.5334, 986.5831, 993.5026, 1004.5915, 1016.5494, 1026.5340, 1029.5957, 1033.5294, 1053.5472, 1055.6246, 1057.5248, 1060.5444, 1066.5116, 1074.5274, 1090.5346, 1092.4565, 1107.5419, 1109.4972, 1111.5759, 1122.5860, 1126.5648, 1132.6784, 1140.5689, 1150.6754, 1152.6441, 1157.5946, 1169.6688, 1184.6097, 1193.6197, 1198.6059, 1201.6200, 1228.6240, 1230.5958, 1234.6802, 1262.6625, 1263.7000, 1265.6524, 1267.6755, 1277.7100, 1290.6733, 1307.6697, 1308.6591, 1314.7606, 1320.5947, 1323.6645, 1329.6493, 1338.6690, 1341.6882, 1357.7032, 1365.6448, 1373.6597, 1379.7061, 1383.6844, 1390.6824, 1407.7186, 1424.7271, 1427.7832, 1434.7521, 1438.6321, 1458.7141, 1475.7546, 1487.7422, 1493.7259, 1541.7313, 1600.8150, 1657.7770, 1699.8072, 1707.7382, 1708.6910, 1753.8091, 1838.8954, 1860.8362, 1940.8932, 2022.8621, 2087.9259, 2225.0667, 2239.0815, 2285.9290, 2383.9100, 2398.9692, 2705.1276, 3312.3547

13. gi|15603268 **Mass:** 20427 **Score:** 63 **Expect:** 1.3 **Queries matched:** 6
50S ribosomal protein L5 [Pasteurella multocida subsp. multocida str. Pm70]

Observed	Mr(expt)	Mr(calc)	ppm	Start	End	Miss	Ions	Peptide
832.4761	831.4688	831.4273	50.0	24 -	30	0	---	K.SVMQVPR.I + Oxidation (M
882.5756	881.5684	881.5698	-1.66	103 -	110	0	40	R.LITIAVPR.I
1037.5324	1036.5251	1036.5302	-4.86	116 -	125	1	---	R.GLSAKSFDGR.G
1212.5840	1211.5767	1211.5757	0.81	1 -	9	1	---	-.MAKLHDYYR.D + Oxidation
1509.7278	1508.7205	1508.7399	-12.84	134 -	145	0	---	R.EQIIFPEIDYDK.V
1879.9133	1878.9060	1878.9363	-16.15	134 -	148	1	---	R.EQIIFPEIDYDKVDR.V

No match to: 807.3826, 827.4583, 856.5182, 870.5347, 897.4091, 973.5334, 986.5831, 993.5026, 1004.5915, 1016.5494, 1026.5340, 1029.5957, 1033.5294, 1053.5472, 1055.6246, 1057.5248, 1060.5444, 1066.5116, 1074.5274, 1090.5346, 1092.4565, 1107.5419, 1109.4972, 1111.5759, 1122.5860, 1126.5648, 1132.6784, 1140.5689, 1150.6754, 1152.6441, 1157.5946, 1169.6688, 1184.6097, 1193.6197, 1198.6059, 1201.6200, 1228.6240, 1230.5958, 1234.6802, 1262.6625, 1263.7000, 1265.6524, 1267.6755, 1277.7100, 1290.6733, 1307.6697, 1308.6591, 1314.7606, 1320.5947, 1323.6645, 1329.6493, 1338.6690, 1341.6882, 1357.7032, 1365.6448, 1373.6597, 1379.7061, 1383.6844, 1390.6824, 1407.7186, 1424.7271, 1427.7832, 1434.7521,

1438.6321, 1458.7141, 1475.7546, 1487.7422, 1493.7259, 1541.7313, 1600.8150, 1657.7770, 1699.8072, 1707.7382, 1708.6910, 1753.8091, 1838.8954, 1860.8362, 1940.8932, 2022.8621, 2087.9259, 2225.0667, 2239.0815, 2285.9290, 2383.9100, 2398.9692, 2705.1276, 3312.3547

14. gi|115525573 **Mass:** 21078 **Score:** 60 **Expect:** 2.9 **Queries matched:** 6
ribosomal protein L5 [Rhodopseudomonas palustris BisA53]

Observed	Mr(expt)	Mr(calc)	ppm	Start	End	Miss	Ions	Peptide
882.5756	881.5684	881.5698	-1.66	109 -	116	0	33	R.LITVALPR.V
1090.5346	1089.5273	1089.5567	-26.94	122 -	131	1	---	R.GLNPKSFDGR.G
1193.6197	1192.6124	1192.6200	-6.37	2 -	11	1	---	M.SETAYTPRLR.A
1424.7271	1423.7198	1423.7612	-29.11	174 -	185	1	---	R.ALLTAFNFPFRQ.-
1600.8150	1599.8078	1599.8403	-20.31	40 -	54	1	---	K.VVLNMGVGEAVNDRK.K
1753.8091	1752.8018	1752.8505	-27.79	22 -	36	0	---	K.LTEEFGYANVMQVPR.L

No match to: 807.3826, 827.4583, 832.4761, 856.5182, 870.5347, 897.4091, 973.5334, 986.5831, 993.5026, 1004.5915, 1016.5494, 1026.5340, 1029.5957, 1033.5294, 1037.5324, 1053.5472, 1055.6246, 1057.5248, 1060.5444, 1066.5116, 1074.5274, 1092.4565, 1107.5419, 1109.4972, 1111.5759, 1122.5860, 1126.5648, 1132.6784, 1140.5689, 1150.6754, 1152.6441, 1157.5946, 1169.6688, 1184.6097, 1198.6059, 1201.6200, 1212.5840, 1228.6240, 1230.5958, 1234.6802, 1262.6625, 1263.7000, 1265.6524, 1267.6755, 1277.7100, 1290.6733, 1307.6697, 1308.6591, 1314.7606, 1320.5947, 1323.6645, 1329.6493, 1338.6690, 1341.6882, 1357.7032, 1365.6448, 1373.6597, 1379.7061, 1383.6844, 1390.6824, 1407.7186, 1427.7832, 1434.7521, 1438.6321, 1458.7141, 1475.7546, 1487.7422, 1493.7259, 1509.7278, 1541.7313, 1657.7770, 1699.8072, 1707.7382, 1708.6910, 1838.8954, 1860.8362, 1879.9133, 1940.8932, 2022.8621, 2087.9259, 2225.0667, 2239.0815, 2285.9290, 2383.9100, 2398.9692, 2705.1276, 3312.3547

15. gi|152978153 **Mass:** 20383 **Score:** 59 **Expect:** 3.5 **Queries matched:** 5
ribosomal protein L5 [Actinobacillus succinogenes 130Z]

Observed	Mr(expt)	Mr(calc)	ppm	Start	End	Miss	Ions	Peptide
882.5756	881.5684	881.5698	-1.66	103 -	110	0	40	R.LITIAVPR.I
1037.5324	1036.5251	1036.5302	-4.86	116 -	125	1	---	R.GLSAKSFDGR.G
1212.5840	1211.5767	1211.5757	0.81	1 -	9	1	---	-.MAKLHDYYR.D + Oxidation
1509.7278	1508.7205	1508.7399	-12.84	134 -	145	0	---	R.EQIIFPEIDYDK.V
1879.9133	1878.9060	1878.9363	-16.15	134 -	148	1	---	R.EQIIFPEIDYDKVDR.V

No match to: 807.3826, 827.4583, 832.4761, 856.5182, 870.5347, 897.4091, 973.5334,

986.5831, 993.5026, 1004.5915, 1016.5494, 1026.5340, 1029.5957, 1033.5294, 1053.5472, 1055.6246, 1057.5248, 1060.5444, 1066.5116, 1074.5274, 1090.5346, 1092.4565, 1107.5419, 1109.4972, 1111.5759, 1122.5860, 1126.5648, 1132.6784, 1140.5689, 1150.6754, 1152.6441, 1157.5946, 1169.6688, 1184.6097, 1193.6197, 1198.6059, 1201.6200, 1228.6240, 1230.5958, 1234.6802, 1262.6625, 1263.7000, 1265.6524, 1267.6755, 1277.7100, 1290.6733, 1307.6697, 1308.6591, 1314.7606, 1320.5947, 1323.6645, 1329.6493, 1338.6690, 1341.6882, 1357.7032, 1365.6448, 1373.6597, 1379.7061, 1383.6844, 1390.6824, 1407.7186, 1424.7271, 1427.7832, 1434.7521, 1438.6321, 1458.7141, 1475.7546, 1487.7422, 1493.7259, 1541.7313, 1600.8150, 1657.7770, 1699.8072, 1707.7382, 1708.6910, 1753.8091, 1838.8954, 1860.8362, 1940.8932, 2022.8621, 2087.9259, 2225.0667, 2239.0815, 2285.9290, 2383.9100, 2398.9692, 2705.1276, 3312.3547

16. gi|33152942 **Mass:** 20430 **Score:** 59 **Expect:** 3.5 **Queries matched:** 5
50S ribosomal protein L5 [Haemophilus ducreyi 35000HP]

Observed	Mr(expt)	Mr(calc)	ppm	Start		End	Miss	Ions	Peptide
882.5756	881.5684	881.5698	-1.66	103	-	110	0	40	R.LITIAVPR.I
1037.5324	1036.5251	1036.5302	-4.86	116	-	125	1	---	R.GLSAKSFDGR.G
1212.5840	1211.5767	1211.5757	0.81	1	-	9	1	---	-.MAKLHDYYR.D + Oxidation
1509.7278	1508.7205	1508.7399	-12.84	134	-	145	0	---	R.EQIIFPEIDYDK.V
1879.9133	1878.9060	1878.9363	-16.15	134	-	148	1	---	R.EQIIFPEIDYDKVDR.V

No match to: 807.3826, 827.4583, 832.4761, 856.5182, 870.5347, 897.4091, 973.5334, 986.5831, 993.5026, 1004.5915, 1016.5494, 1026.5340, 1029.5957, 1033.5294, 1053.5472, 1055.6246, 1057.5248, 1060.5444, 1066.5116, 1074.5274, 1090.5346, 1092.4565, 1107.5419, 1109.4972, 1111.5759, 1122.5860, 1126.5648, 1132.6784, 1140.5689, 1150.6754, 1152.6441, 1157.5946, 1169.6688, 1184.6097, 1193.6197, 1198.6059, 1201.6200, 1228.6240, 1230.5958, 1234.6802, 1262.6625, 1263.7000, 1265.6524, 1267.6755, 1277.7100, 1290.6733, 1307.6697, 1308.6591, 1314.7606, 1320.5947, 1323.6645, 1329.6493, 1338.6690, 1341.6882, 1357.7032, 1365.6448, 1373.6597, 1379.7061, 1383.6844, 1390.6824, 1407.7186, 1424.7271, 1427.7832, 1434.7521, 1438.6321, 1458.7141, 1475.7546, 1487.7422, 1493.7259, 1541.7313, 1600.8150, 1657.7770, 1699.8072, 1707.7382, 1708.6910, 1753.8091, 1838.8954, 1860.8362, 1940.8932, 2022.8621, 2087.9259, 2225.0667, 2239.0815, 2285.9290, 2383.9100, 2398.9692, 2705.1276, 3312.3547

17. gi|52426091 **Mass:** 20352 **Score:** 59 **Expect:** 3.5 **Queries matched:** 5
50S ribosomal protein L5 [Mannheimia succiniciproducens MBEL55E]

Observed	Mr(expt)	Mr(calc)	ppm	Start		End	Miss	Ions	Peptide
882.5756	881.5684	881.5698	-1.66	103	-	110	0	40	R.LITIAVPR.I
1037.5324	1036.5251	1036.5302	-4.86	116	-	125	1	---	R.GLSAKSFDGR.G
1212.5840	1211.5767	1211.5757	0.81	1	-	9	1	---	-.MAKLHDYYR.D + Oxidation
1509.7278	1508.7205	1508.7399	-12.84	134	-	145	0	---	R.EQIIFPEIDYDK.V
1879.9133	1878.9060	1878.9363	-16.15	134	-	148	1	---	R.EQIIFPEIDYDKVDR.V

No match to: 807.3826, 827.4583, 832.4761, 856.5182, 870.5347, 897.4091, 973.5334, 986.5831, 993.5026, 1004.5915, 1016.5494, 1026.5340, 1029.5957, 1033.5294, 1053.5472, 1055.6246, 1057.5248, 1060.5444, 1066.5116, 1074.5274, 1090.5346, 1092.4565, 1107.5419, 1109.4972, 1111.5759, 1122.5860, 1126.5648, 1132.6784, 1140.5689, 1150.6754, 1152.6441, 1157.5946, 1169.6688, 1184.6097, 1193.6197, 1198.6059, 1201.6200, 1228.6240, 1230.5958, 1234.6802, 1262.6625, 1263.7000, 1265.6524, 1267.6755, 1277.7100, 1290.6733, 1307.6697, 1308.6591, 1314.7606, 1320.5947, 1323.6645, 1329.6493, 1338.6690, 1341.6882, 1357.7032, 1365.6448, 1373.6597, 1379.7061, 1383.6844, 1390.6824, 1407.7186, 1424.7271, 1427.7832, 1434.7521, 1438.6321, 1458.7141, 1475.7546, 1487.7422, 1493.7259, 1541.7313, 1600.8150, 1657.7770, 1699.8072, 1707.7382, 1708.6910, 1753.8091, 1838.8954, 1860.8362, 1940.8932, 2022.8621, 2087.9259, 2225.0667, 2239.0815, 2285.9290, 2383.9100, 2398.9692, 2705.1276, 3312.3547

18. gi|16120559 **Mass:** 20308 **Score:** 59 **Expect:** 3.6 **Queries matched:** 5
50S ribosomal protein L5 [Yersinia pestis CO92]

Observed	Mr(expt)	Mr(calc)	ppm	Start		End	Miss	Ions	Peptide
882.5756	881.5684	881.5698	-1.66	103	-	110	0	40	R.LITIAVPR.I
1037.5324	1036.5251	1036.5302	-4.86	116	-	125	1	---	R.GLSAKSFDGR.G
1184.6097	1183.6025	1183.5695	27.8	1	-	9	1	---	-.MAKLHDYYK.D + Oxidation
1509.7278	1508.7205	1508.7399	-12.84	134	-	145	0	---	R.EQIIFPEIDYDK.V
1879.9133	1878.9060	1878.9363	-16.15	134	-	148	1	---	R.EQIIFPEIDYDKVDR.V

No match to: 807.3826, 827.4583, 832.4761, 856.5182, 870.5347, 897.4091, 973.5334, 986.5831, 993.5026, 1004.5915, 1016.5494, 1026.5340, 1029.5957, 1033.5294, 1053.5472, 1055.6246, 1057.5248, 1060.5444, 1066.5116, 1074.5274, 1090.5346, 1092.4565, 1107.5419, 1109.4972, 1111.5759, 1122.5860, 1126.5648, 1132.6784, 1140.5689, 1150.6754, 1152.6441, 1157.5946, 1169.6688, 1193.6197, 1198.6059, 1201.6200, 1212.5840, 1228.6240, 1230.5958, 1234.6802, 1262.6625, 1263.7000, 1265.6524, 1267.6755, 1277.7100, 1290.6733, 1307.6697, 1308.6591, 1314.7606, 1320.5947, 1323.6645, 1329.6493, 1338.6690, 1341.6882, 1357.7032,

1365.6448, 1373.6597, 1379.7061, 1383.6844, 1390.6824, 1407.7186, 1424.7271, 1427.7832, 1434.7521, 1438.6321, 1458.7141, 1475.7546, 1487.7422, 1493.7259, 1541.7313, 1600.8150, 1657.7770, 1699.8072, 1707.7382, 1708.6910, 1753.8091, 1838.8954, 1860.8362, 1940.8932, 2022.8621, 2087.9259, 2225.0667, 2239.0815, 2285.9290, 2383.9100, 2398.9692, 2705.1276, 3312.3547

19. gi|77957319 **Mass:** 20307 **Score:** 59 **Expect:** 3.6 **Queries matched:** 5
COG0094: Ribosomal protein L5 [Yersinia bercovieri ATCC 43970]

Observed	Mr(expt)	Mr(calc)	ppm	Start	End	Miss	Ions	Peptide
882.5756	881.5684	881.5698	-1.66	103 -	110	0	40	R.LITIAVPR.I
1037.5324	1036.5251	1036.5302	-4.86	116 -	125	1	---	R.GLSAKSFDGR.G
1184.6097	1183.6025	1183.5695	27.8	1 -	9	1	---	-.MAKLHDYYK.D + Oxidation
1509.7278	1508.7205	1508.7399	-12.84	134 -	145	0	---	R.EQIIFPEIDYDK.V
1879.9133	1878.9060	1878.9363	-16.15	134 -	148	1	---	R.EQIIFPEIDYDKVDR.V

No match to: 807.3826, 827.4583, 832.4761, 856.5182, 870.5347, 897.4091, 973.5334, 986.5831, 993.5026, 1004.5915, 1016.5494, 1026.5340, 1029.5957, 1033.5294, 1053.5472, 1055.6246, 1057.5248, 1060.5444, 1066.5116, 1074.5274, 1090.5346, 1092.4565, 1107.5419, 1109.4972, 1111.5759, 1122.5860, 1126.5648, 1132.6784, 1140.5689, 1150.6754, 1152.6441, 1157.5946, 1169.6688, 1193.6197, 1198.6059, 1201.6200, 1212.5840, 1228.6240, 1230.5958, 1234.6802, 1262.6625, 1263.7000, 1265.6524, 1267.6755, 1277.7100, 1290.6733, 1307.6697, 1308.6591, 1314.7606, 1320.5947, 1323.6645, 1329.6493, 1338.6690, 1341.6882, 1357.7032, 1365.6448, 1373.6597, 1379.7061, 1383.6844, 1390.6824, 1407.7186, 1424.7271, 1427.7832, 1434.7521, 1438.6321, 1458.7141, 1475.7546, 1487.7422, 1493.7259, 1541.7313, 1600.8150, 1657.7770, 1699.8072, 1707.7382, 1708.6910, 1753.8091, 1838.8954, 1860.8362, 1940.8932, 2022.8621, 2087.9259, 2225.0667, 2239.0815, 2285.9290, 2383.9100, 2398.9692, 2705.1276, 3312.3547

20. gi|123444086 **Mass:** 20331 **Score:** 59 **Expect:** 3.6 **Queries matched:** 5
50S ribosomal protein L5 [Yersinia enterocolitica subsp. enterocolitica 8081]

Observed	Mr(expt)	Mr(calc)	ppm	Start	End	Miss	Ions	Peptide
882.5756	881.5684	881.5698	-1.66	103 -	110	0	40	R.LITIAVPR.I
1037.5324	1036.5251	1036.5302	-4.86	116 -	125	1	---	R.GLSAKSFDGR.G
1184.6097	1183.6025	1183.5695	27.8	1 -	9	1	---	-.MAKLHDYYK.D + Oxidation
1509.7278	1508.7205	1508.7399	-12.84	134 -	145	0	---	R.EQIIFPEIDYDK.V

1879.9133	1878.9060	1878.9363	-16.15	134 - 148		1	---	R.EQIIFPEIDYDKVDR.V

No match to: 807.3826, 827.4583, 832.4761, 856.5182, 870.5347, 897.4091, 973.5334, 986.5831, 993.5026, 1004.5915, 1016.5494, 1026.5340, 1029.5957, 1033.5294, 1053.5472, 1055.6246, 1057.5248, 1060.5444, 1066.5116, 1074.5274, 1090.5346, 1092.4565, 1107.5419, 1109.4972, 1111.5759, 1122.5860, 1126.5648, 1132.6784, 1140.5689, 1150.6754, 1152.6441, 1157.5946, 1169.6688, 1193.6197, 1198.6059, 1201.6200, 1212.5840, 1228.6240, 1230.5958, 1234.6802, 1262.6625, 1263.7000, 1265.6524, 1267.6755, 1277.7100, 1290.6733, 1307.6697, 1308.6591, 1314.7606, 1320.5947, 1323.6645, 1329.6493, 1338.6690, 1341.6882, 1357.7032, 1365.6448, 1373.6597, 1379.7061, 1383.6844, 1390.6824, 1407.7186, 1424.7271, 1427.7832, 1434.7521, 1438.6321, 1458.7141, 1475.7546, 1487.7422, 1493.7259, 1541.7313, 1600.8150, 1657.7770, 1699.8072, 1707.7382, 1708.6910, 1753.8091, 1838.8954, 1860.8362, 1940.8932, 2022.8621, 2087.9259, 2225.0667, 2239.0815, 2285.9290, 2383.9100, 2398.9692, 2705.1276, 3312.3547

21. gi|30995402 **Mass:** 15932 **Score:** 58 **Expect:** 4.3 **Queries matched:** 4

hypothetical protein HI0790 [Haemophilus influenzae Rd KW20]

Observed	Mr(expt)	Mr(calc)	ppm	Start	End	Miss	Ions	Peptide
882.5756	881.5684	881.5698	-1.66	66 -	73	0	40	R.LITIAVPR.I
1037.5324	1036.5251	1036.5302	-4.86	79 -	88	1	---	R.GLSAKSFDGR.G
1509.7278	1508.7205	1508.7399	-12.84	97 -	108	0	---	R.EQIIFPEIDYDK.V
1879.9133	1878.9060	1878.9363	-16.15	97 -	111	1	---	R.EQIIFPEIDYDKVDR.V

No match to: 807.3826, 827.4583, 832.4761, 856.5182, 870.5347, 897.4091, 973.5334, 986.5831, 993.5026, 1004.5915, 1016.5494, 1026.5340, 1029.5957, 1033.5294, 1053.5472, 1055.6246, 1057.5248, 1060.5444, 1066.5116, 1074.5274, 1090.5346, 1092.4565, 1107.5419, 1109.4972, 1111.5759, 1122.5860, 1126.5648, 1132.6784, 1140.5689, 1150.6754, 1152.6441, 1157.5946, 1169.6688, 1184.6097, 1193.6197, 1198.6059, 1201.6200, 1212.5840, 1228.6240, 1230.5958, 1234.6802, 1262.6625, 1263.7000, 1265.6524, 1267.6755, 1277.7100, 1290.6733, 1307.6697, 1308.6591, 1314.7606, 1320.5947, 1323.6645, 1329.6493, 1338.6690, 1341.6882, 1357.7032, 1365.6448, 1373.6597, 1379.7061, 1383.6844, 1390.6824, 1407.7186, 1424.7271, 1427.7832, 1434.7521, 1438.6321, 1458.7141, 1475.7546, 1487.7422, 1493.7259, 1541.7313, 1600.8150, 1657.7770, 1699.8072, 1707.7382, 1708.6910, 1753.8091, 1838.8954, 1860.8362, 1940.8932, 2022.8621, 2087.9259, 2225.0667, 2239.0815, 2285.9290, 2383.9100, 2398.9692, 2705.1276, 3312.3547

22. gi|125716997 **Mass:** 19863 **Score:** 58 **Expect:** 4.4 **Queries matched:** 4

50S ribosomal protein L5, putative [Streptococcus sanguinis SK36]

Observed	Mr(expt)	Mr(calc)	ppm	Start	End	Miss	Ions	Peptide
870.5347	869.5274	869.5334	-6.94	104 -	111	0	38	K.LVSVSLPR.V
1487.7422	1486.7350	1486.7814	-31.20	35 -	49	0	---	K.IVLNMGVGDAVSNAK.N
1600.8150	1599.8078	1599.7967	6.94	21 -	34	1	---	K.FNYSSVMAVPKVDK.I + Oxida
2225.0667	2224.0595	2224.1449	-38.44	12 -	31	1	---	K.EVVPSLTEKFNYSSVMAVPK.V

No match to: 807.3826, 827.4583, 832.4761, 856.5182, 882.5756, 897.4091, 973.5334, 986.5831, 993.5026, 1004.5915, 1016.5494, 1026.5340, 1029.5957, 1033.5294, 1037.5324, 1053.5472, 1055.6246, 1057.5248, 1060.5444, 1066.5116, 1074.5274, 1090.5346, 1092.4565, 1107.5419, 1109.4972, 1111.5759, 1122.5860, 1126.5648, 1132.6784, 1140.5689, 1150.6754, 1152.6441, 1157.5946, 1169.6688, 1184.6097, 1193.6197, 1198.6059, 1201.6200, 1212.5840, 1228.6240, 1230.5958, 1234.6802, 1262.6625, 1263.7000, 1265.6524, 1267.6755, 1277.7100, 1290.6733, 1307.6697, 1308.6591, 1314.7606, 1320.5947, 1323.6645, 1329.6493, 1338.6690, 1341.6882, 1357.7032, 1365.6448, 1373.6597, 1379.7061, 1383.6844, 1390.6824, 1407.7186, 1424.7271, 1427.7832, 1434.7521, 1438.6321, 1458.7141, 1475.7546, 1493.7259, 1509.7278, 1541.7313, 1657.7770, 1699.8072, 1707.7382, 1708.6910, 1753.8091, 1838.8954, 1860.8362, 1879.9133, 1940.8932, 2022.8621, 2087.9259, 2239.0815, 2285.9290, 2383.9100, 2398.9692, 2705.1276, 3312.3547

23. gi|121591845 **Mass:** 19566 **Score:** 58 **Expect:** 4.5 **Queries matched:** 7

ribosomal protein L5 [Vibrio cholerae 2740-80]

Observed	Mr(expt)	Mr(calc)	ppm	Start	End	Miss	Ions	Peptide
882.5756	881.5684	881.5698	-1.64	103 -	110	0	25	R.LISIALPR.V
1037.5324	1036.5251	1036.5341	-8.72	2 -	9	1	---	M.AKLHDYYK.S
1184.6097	1183.6025	1183.5695	27.8	1 -	9	1	---	-.MAKLHDYYK.S + Oxidation
1338.6690	1337.6618	1337.6186	32.2	93 -	102	1	---	R.GERMWDFLER.L
1458.7141	1457.7069	1457.6973	6.56	19 -	30	0	---	K.QFSYTSVMQVPR.I + Oxidati
1509.7278	1508.7205	1508.7399	-12.84	134 -	145	0	---	R.EQIIFPEIDYDK.V
1879.9133	1878.9060	1878.9363	-16.15	134 -	148	1	---	R.EQIIFPEIDYDKVDR.V

No match to: 807.3826, 827.4583, 832.4761, 856.5182, 870.5347, 897.4091, 973.5334, 986.5831, 993.5026, 1004.5915, 1016.5494, 1026.5340, 1029.5957, 1033.5294, 1053.5472, 1055.6246, 1057.5248, 1060.5444, 1066.5116, 1074.5274, 1090.5346, 1092.4565, 1107.5419, 1109.4972, 1111.5759, 1122.5860, 1126.5648, 1132.6784, 1140.5689, 1150.6754, 1152.6441, 1157.5946, 1169.6688, 1193.6197, 1198.6059, 1201.6200, 1212.5840, 1228.6240, 1230.5958,

1234.6802, 1262.6625, 1263.7000, 1265.6524, 1267.6755, 1277.7100, 1290.6733, 1307.6697, 1308.6591, 1314.7606, 1320.5947, 1323.6645, 1329.6493, 1341.6882, 1357.7032, 1365.6448, 1373.6597, 1379.7061, 1383.6844, 1390.6824, 1407.7186, 1424.7271, 1427.7832, 1434.7521, 1438.6321, 1475.7546, 1487.7422, 1493.7259, 1541.7313, 1600.8150, 1657.7770, 1699.8072, 1707.7382, 1708.6910, 1753.8091, 1838.8954, 1860.8362, 1940.8932, 2022.8621, 2087.9259, 2225.0667, 2239.0815, 2285.9290, 2383.9100, 2398.9692, 2705.1276, 3312.3547

24. gi|145620236 **Mass:** 19918 **Score:** 57 **Expect:** 5.1 **Queries matched:** 5
ribosomal protein L5 [Geobacter bemidjiensis Bem]

Observed	Mr(expt)	Mr(calc)	ppm	Start	End	Miss	Ions	Peptide
870.5347	869.5274	869.5334	-6.94	103 -	110	0	38	R.LVSVSLPR.V
1230.5958	1229.5886	1229.5750	11.0	94 -	102	1	---	R.EKMYEFLDR.L
1458.7141	1457.7069	1457.7595	-36.12	81 -	93	1	---	R.QGMPIGCAVTLRR.E
1509.7278	1508.7205	1508.7399	-12.84	134 -	145	0	---	K.EQLIFPEIDYDK.V
1600.8150	1599.8078	1599.8654	-36.03	34 -	48	0	---	K.IVVNMGLGEAIQNVK.I + Oxid

No match to: 807.3826, 827.4583, 832.4761, 856.5182, 882.5756, 897.4091, 973.5334, 986.5831, 993.5026, 1004.5915, 1016.5494, 1026.5340, 1029.5957, 1033.5294, 1037.5324, 1053.5472, 1055.6246, 1057.5248, 1060.5444, 1066.5116, 1074.5274, 1090.5346, 1092.4565, 1107.5419, 1109.4972, 1111.5759, 1122.5860, 1126.5648, 1132.6784, 1140.5689, 1150.6754, 1152.6441, 1157.5946, 1169.6688, 1184.6097, 1193.6197, 1198.6059, 1201.6200, 1212.5840, 1228.6240, 1234.6802, 1262.6625, 1263.7000, 1265.6524, 1267.6755, 1277.7100, 1290.6733, 1307.6697, 1308.6591, 1314.7606, 1320.5947, 1323.6645, 1329.6493, 1338.6690, 1341.6882, 1357.7032, 1365.6448, 1373.6597, 1379.7061, 1383.6844, 1390.6824, 1407.7186, 1424.7271, 1427.7832, 1434.7521, 1438.6321, 1475.7546, 1487.7422, 1493.7259, 1541.7313, 1657.7770, 1699.8072, 1707.7382, 1708.6910, 1753.8091, 1838.8954, 1860.8362, 1879.9133, 1940.8932, 2022.8621, 2087.9259, 2225.0667, 2239.0815, 2285.9290, 2383.9100, 2398.9692, 2705.1276, 3312.3547

25. gi|15642579 **Mass:** 20203 **Score:** 57 **Expect:** 5.2 **Queries matched:** 7
50S ribosomal protein L5 [Vibrio cholerae O1 biovar eltor str. N16961]

Observed	Mr(expt)	Mr(calc)	ppm	Start	End	Miss	Ions	Peptide
882.5756	881.5684	881.5698	-1.64	103 -	110	0	25	R.LISIALPR.V
1037.5324	1036.5251	1036.5341	-8.72	2 -	9	1	---	M.AKLHDYYK.S
1184.6097	1183.6025	1183.5695	27.8	1 -	9	1	---	-.MAKLHDYYK.S + Oxidation

1338.6690	1337.6618	1337.6186	32.2	93 -	102	1	---	R.GERMWDFLER.L
1458.7141	1457.7069	1457.6973	6.56	19 -	30	0	---	K.QFSYTSVMQVPR.I + Oxidati
1509.7278	1508.7205	1508.7399	-12.84	134 -	145	0	---	R.EQIIFPEIDYDK.V
1879.9133	1878.9060	1878.9363	-16.15	134 -	148	1	---	R.EQIIFPEIDYDKVDR.V

No match to: 807.3826, 827.4583, 832.4761, 856.5182, 870.5347, 897.4091, 973.5334, 986.5831, 993.5026, 1004.5915, 1016.5494, 1026.5340, 1029.5957, 1033.5294, 1053.5472, 1055.6246, 1057.5248, 1060.5444, 1066.5116, 1074.5274, 1090.5346, 1092.4565, 1107.5419, 1109.4972, 1111.5759, 1122.5860, 1126.5648, 1132.6784, 1140.5689, 1150.6754, 1152.6441, 1157.5946, 1169.6688, 1193.6197, 1198.6059, 1201.6200, 1212.5840, 1228.6240, 1230.5958, 1234.6802, 1262.6625, 1263.7000, 1265.6524, 1267.6755, 1277.7100, 1290.6733, 1307.6697, 1308.6591, 1314.7606, 1320.5947, 1323.6645, 1329.6493, 1341.6882, 1357.7032, 1365.6448, 1373.6597, 1379.7061, 1383.6844, 1390.6824, 1407.7186, 1424.7271, 1427.7832, 1434.7521, 1438.6321, 1475.7546, 1487.7422, 1493.7259, 1541.7313, 1600.8150, 1657.7770, 1699.8072, 1707.7382, 1708.6910, 1753.8091, 1838.8954, 1860.8362, 1940.8932, 2022.8621, 2087.9259, 2225.0667, 2239.0815, 2285.9290, 2383.9100, 2398.9692, 2705.1276, 3312.3547

26. gi|29653798 **Mass:** 25907 **Score:** 57 **Expect:** 5.5 **Queries matched:** 9

adenylate kinase [Coxiella burnetii RSA 493]

Observed	Mr(expt)	Mr(calc)	ppm	Start	End	Miss	Ions	Peptide
856.5182	855.5109	855.5066	5.05	45 -	52	0	9	K.TPLGLEVK.K
1037.5324	1036.5251	1036.5342	-8.75	82 -	90	0	---	K.GYLLDGFPR.T
1055.6246	1054.6174	1054.6386	-20.17	43 -	52	1	---	K.AKTPLGLEVK.K
1090.5346	1089.5273	1089.5051	20.5	159 -	167	1	---	R.ADDREETVR.H
1193.6197	1192.6124	1192.5758	30.7	201 -	211	0	---	R.ISGLGSMEEVR.E + Oxidatio
1265.6524	1264.6451	1264.6200	19.8	190 -	200	1	---	K.SGDPQAPKYFR.I
1277.7100	1276.7027	1276.6809	17.1	31 -	42	1	---	K.ISTGDMLRAAVK.A + Oxidati
1407.7186	1406.7114	1406.7531	-29.66	126 -	137	1	---	R.LIHPASGRTYHR.R
1699.8072	1698.7999	1698.8173	-10.21	148 -	162	1	---	K.DDVTGEPLIQRADDR.E

No match to: 807.3826, 827.4583, 832.4761, 870.5347, 882.5756, 897.4091, 973.5334, 986.5831, 993.5026, 1004.5915, 1016.5494, 1026.5340, 1029.5957, 1033.5294, 1053.5472, 1057.5248, 1060.5444, 1066.5116, 1074.5274, 1092.4565, 1107.5419, 1109.4972, 1111.5759, 1122.5860, 1126.5648, 1132.6784, 1140.5689, 1150.6754, 1152.6441, 1157.5946, 1169.6688, 1184.6097, 1198.6059, 1201.6200, 1212.5840, 1228.6240, 1230.5958, 1234.6802, 1262.6625, 1263.7000, 1267.6755, 1290.6733, 1307.6697, 1308.6591, 1314.7606, 1320.5947, 1323.6645,

1329.6493, 1338.6690, 1341.6882, 1357.7032, 1365.6448, 1373.6597, 1379.7061, 1383.6844, 1390.6824, 1424.7271, 1427.7832, 1434.7521, 1438.6321, 1458.7141, 1475.7546, 1487.7422, 1493.7259, 1509.7278, 1541.7313, 1600.8150, 1657.7770, 1707.7382, 1708.6910, 1753.8091, 1838.8954, 1860.8362, 1879.9133, 1940.8932, 2022.8621, 2087.9259, 2225.0667, 2239.0815, 2285.9290, 2383.9100, 2398.9692, 2705.1276, 3312.3547

27. gi|154254005 **Mass:** 68655 **Score:** 55 **Expect:** 8.9 **Queries matched:** 13
chaperone protein DnaK [Parvibaculum lavamentivorans DS-1]

Observed	Mr(expt)	Mr(calc)	ppm	Start	End	Miss	Ions	Peptide
870.5347	869.5274	869.5334	-6.92	160 -	167	0	---	K.IAGLEVLR.I
1037.5324	1036.5251	1036.5263	-1.16	87 -	95	0	---	K.GMVPYDIVK.A + Oxidation
1053.5472	1052.5399	1052.5655	-24.32	346 -	354	1	---	R.EAVKQFFGK.E
1060.5444	1059.5372	1059.5672	-28.40	487 -	495	1	---	K.ATNKEQTIR.I
1126.5648	1125.5575	1125.5190	34.2	125 -	134	0	---	K.ETAESYLGEK.V
1157.5946	1156.5873	1156.6088	-18.54	558 -	568	0	---	K.ADVEAALEALR.T
1277.7100	1276.7027	1276.6510	40.5	569 -	580	1	---	R.TALKEGSSEDIK.A
1338.6690	1337.6618	1337.6683	-4.88	581 -	592	1	---	K.AKMEALTQTSMK.M
1487.7422	1486.7350	1486.7627	-18.67	544 -	557	1	---	K.TLQENADKIGGDVK.A
1493.7259	1492.7186	1492.7522	-22.48	72 -	84	1	---	R.LIGRSYDDPTTQK.D
1838.8954	1837.8881	1837.8806	4.07	418 -	434	0	---	K.AQTFSTAEDNQSAVTIR.V
2225.0667	2224.0595	2224.0715	-5.44	4 -	25	0	---	K.VIGIDLGTTNSCVAVMEGSSAK.V
2239.0815	2238.0743	2238.0951	-9.30	35 -	55	1	---	R.TTPSMVAFTQDGERLVGQSAK.R

No match to: 807.3826, 827.4583, 832.4761, 856.5182, 882.5756, 897.4091, 973.5334, 986.5831, 993.5026, 1004.5915, 1016.5494, 1026.5340, 1029.5957, 1033.5294, 1055.6246, 1057.5248, 1066.5116, 1074.5274, 1090.5346, 1092.4565, 1107.5419, 1109.4972, 1111.5759, 1122.5860, 1132.6784, 1140.5689, 1150.6754, 1152.6441, 1169.6688, 1184.6097, 1193.6197, 1198.6059, 1201.6200, 1212.5840, 1228.6240, 1230.5958, 1234.6802, 1262.6625, 1263.7000, 1265.6524, 1267.6755, 1290.6733, 1307.6697, 1308.6591, 1314.7606, 1320.5947, 1323.6645, 1329.6493, 1341.6882, 1357.7032, 1365.6448, 1373.6597, 1379.7061, 1383.6844, 1390.6824, 1407.7186, 1424.7271, 1427.7832, 1434.7521, 1438.6321, 1458.7141, 1475.7546, 1509.7278, 1541.7313, 1600.8150, 1657.7770, 1699.8072, 1707.7382, 1708.6910, 1753.8091, 1860.8362, 1879.9133, 1940.8932, 2022.8621, 2087.9259, 2285.9290, 2383.9100, 2398.9692, 2705.1276, 3312.3547

28. gi|127511486 **Mass:** 7201 **Score:** 54 **Expect:** 10 **Queries matched:** 6
BFD domain protein (2Fe-2S)-binding domain protein [Shewanella loihica PV-4]

Observed	Mr(expt)	Mr(calc)	ppm	Start	End	Miss	Ions	Peptide
1267.6755	1266.6682	1266.6132	43.4	53 -	63	0	---	R.LDVTPNFYEVA.-
1307.6697	1306.6624	1306.6122	38.5	32 -	43	1	---	R.LGVGNQCGKCSK.M
1379.7061	1378.6989	1378.6373	44.6	2 -	12	0	---	M.YVCLCHAITDK.Q
1407.7186	1406.7114	1406.7010	7.39	41 -	52	1	---	K.CSKMATEIIQAR.L
1493.7259	1492.7186	1492.6715	31.5	16 -	29	0	---	K.EAVDQGDMSLADVK.R + Oxidat
1879.9133	1878.9060	1878.9154	-5.02	1 -	15	1	---	-.MYVCLCHAITDKQIK.E

No match to: 807.3826, 827.4583, 832.4761, 856.5182, 870.5347, 882.5756, 897.4091, 973.5334, 986.5831, 993.5026, 1004.5915, 1016.5494, 1026.5340, 1029.5957, 1033.5294, 1037.5324, 1053.5472, 1055.6246, 1057.5248, 1060.5444, 1066.5116, 1074.5274, 1090.5346, 1092.4565, 1107.5419, 1109.4972, 1111.5759, 1122.5860, 1126.5648, 1132.6784, 1140.5689, 1150.6754, 1152.6441, 1157.5946, 1169.6688, 1184.6097, 1193.6197, 1198.6059, 1201.6200, 1212.5840, 1228.6240, 1230.5958, 1234.6802, 1262.6625, 1263.7000, 1265.6524, 1277.7100, 1290.6733, 1308.6591, 1314.7606, 1320.5947, 1323.6645, 1329.6493, 1338.6690, 1341.6882, 1357.7032, 1365.6448, 1373.6597, 1383.6844, 1390.6824, 1424.7271, 1427.7832, 1434.7521, 1438.6321, 1458.7141, 1475.7546, 1487.7422, 1509.7278, 1541.7313, 1600.8150, 1657.7770, 1699.8072, 1707.7382, 1708.6910, 1753.8091, 1838.8954, 1860.8362, 1940.8932, 2022.8621, 2087.9259, 2225.0667, 2239.0815, 2285.9290, 2383.9100, 2398.9692, 2705.1276, 3312.3547

29. gi|158424166 **Mass:** 21110 **Score:** 54 **Expect:** 11 **Queries matched:** 6
ribosomal protein L5 [Azorhizobium caulinodans ORS 571]

Observed	Mr(expt)	Mr(calc)	ppm	Start	End	Miss	Ions	Peptide
882.5756	881.5684	881.5698	-1.66	109 -	116	0	33	R.LITVALPR.V
1026.5340	1025.5267	1025.5083	18.0	178 -	185	1	---	R.AFNFPFRQ.-
1090.5346	1089.5273	1089.5567	-26.94	122 -	131	1	---	R.GLNPKSFDGR.G
1111.5759	1110.5686	1110.6145	-41.33	85 -	94	1	---	K.LRENQPVGAK.V
1201.6200	1200.6127	1200.6251	-10.33	12 -	21	0	---	R.THYDSVVRPK.L
1308.6591	1307.6519	1307.6656	-10.48	1 -	11	1	---	-.MAEATYTPRLR.T

No match to: 807.3826, 827.4583, 832.4761, 856.5182, 870.5347, 897.4091, 973.5334, 986.5831, 993.5026, 1004.5915, 1016.5494, 1029.5957, 1033.5294, 1037.5324, 1053.5472, 1055.6246, 1057.5248, 1060.5444, 1066.5116, 1074.5274, 1092.4565, 1107.5419, 1109.4972, 1122.5860, 1126.5648, 1132.6784, 1140.5689, 1150.6754, 1152.6441, 1157.5946, 1169.6688,

1184.6097, 1193.6197, 1198.6059, 1212.5840, 1228.6240, 1230.5958, 1234.6802, 1262.6625, 1263.7000, 1265.6524, 1267.6755, 1277.7100, 1290.6733, 1307.6697, 1314.7606, 1320.5947, 1323.6645, 1329.6493, 1338.6690, 1341.6882, 1357.7032, 1365.6448, 1373.6597, 1379.7061, 1383.6844, 1390.6824, 1407.7186, 1424.7271, 1427.7832, 1434.7521, 1438.6321, 1458.7141, 1475.7546, 1487.7422, 1493.7259, 1509.7278, 1541.7313, 1600.8150, 1657.7770, 1699.8072, 1707.7382, 1708.6910, 1753.8091, 1838.8954, 1860.8362, 1879.9133, 1940.8932, 2022.8621, 2087.9259, 2225.0667, 2239.0815, 2285.9290, 2383.9100, 2398.9692, 2705.1276, 3312.3547

30. gi|113460221 **Mass:** 20358 **Score:** 54 **Expect:** 12 **Queries matched:** 4

50S ribosomal protein L5 [Haemophilus somnus 129PT]

Observed	Mr(expt)	Mr(calc)	ppm	Start		End	Miss	Ions	Peptide
882.5756	881.5684	881.5698	-1.66	103	-	110	0	40	R.LITIAVPR.I
1212.5840	1211.5767	1211.5757	0.81	1	-	9	1	---	-.MAKLHDYYR.D + Oxidation
1509.7278	1508.7205	1508.7399	-12.84	134	-	145	0	---	R.EQIIFPEIDYDK.V
1879.9133	1878.9060	1878.9363	-16.15	134	-	148	1	---	R.EQIIFPEIDYDKVDR.V

No match to: 807.3826, 827.4583, 832.4761, 856.5182, 870.5347, 897.4091, 973.5334, 986.5831, 993.5026, 1004.5915, 1016.5494, 1026.5340, 1029.5957, 1033.5294, 1037.5324, 1053.5472, 1055.6246, 1057.5248, 1060.5444, 1066.5116, 1074.5274, 1090.5346, 1092.4565, 1107.5419, 1109.4972, 1111.5759, 1122.5860, 1126.5648, 1132.6784, 1140.5689, 1150.6754, 1152.6441, 1157.5946, 1169.6688, 1184.6097, 1193.6197, 1198.6059, 1201.6200, 1228.6240, 1230.5958, 1234.6802, 1262.6625, 1263.7000, 1265.6524, 1267.6755, 1277.7100, 1290.6733, 1307.6697, 1308.6591, 1314.7606, 1320.5947, 1323.6645, 1329.6493, 1338.6690, 1341.6882, 1357.7032, 1365.6448, 1373.6597, 1379.7061, 1383.6844, 1390.6824, 1407.7186, 1424.7271, 1427.7832, 1434.7521, 1438.6321, 1458.7141, 1475.7546, 1487.7422, 1493.7259, 1541.7313, 1600.8150, 1657.7770, 1699.8072, 1707.7382, 1708.6910, 1753.8091, 1838.8954, 1860.8362, 1940.8932, 2022.8621, 2087.9259, 2225.0667, 2239.0815, 2285.9290, 2383.9100, 2398.9692, 2705.1276, 3312.3547

31. gi|126209235 **Mass:** 20399 **Score:** 54 **Expect:** 12 **Queries matched:** 4

50S ribosomal protein L5 [Actinobacillus pleuropneumoniae L20]

Observed	Mr(expt)	Mr(calc)	ppm	Start		End	Miss	Ions	Peptide
882.5756	881.5684	881.5698	-1.66	103	-	110	0	40	R.LITIAVPR.I
1212.5840	1211.5767	1211.5757	0.81	1	-	9	1	---	-.MAKLHDYYR.D + Oxidation
1509.7278	1508.7205	1508.7399	-12.84	134	-	145	0	---	R.EQIIFPEIDYDK.V

1879.9133	1878.9060	1878.9363	-16.15	134 - 148	1	---	R.EQIIFPEIDYDKVDR.V

No match to: 807.3826, 827.4583, 832.4761, 856.5182, 870.5347, 897.4091, 973.5334, 986.5831, 993.5026, 1004.5915, 1016.5494, 1026.5340, 1029.5957, 1033.5294, 1037.5324, 1053.5472, 1055.6246, 1057.5248, 1060.5444, 1066.5116, 1074.5274, 1090.5346, 1092.4565, 1107.5419, 1109.4972, 1111.5759, 1122.5860, 1126.5648, 1132.6784, 1140.5689, 1150.6754, 1152.6441, 1157.5946, 1169.6688, 1184.6097, 1193.6197, 1198.6059, 1201.6200, 1228.6240, 1230.5958, 1234.6802, 1262.6625, 1263.7000, 1265.6524, 1267.6755, 1277.7100, 1290.6733, 1307.6697, 1308.6591, 1314.7606, 1320.5947, 1323.6645, 1329.6493, 1338.6690, 1341.6882, 1357.7032, 1365.6448, 1373.6597, 1379.7061, 1383.6844, 1390.6824, 1407.7186, 1424.7271, 1427.7832, 1434.7521, 1438.6321, 1458.7141, 1475.7546, 1487.7422, 1493.7259, 1541.7313, 1600.8150, 1657.7770, 1699.8072, 1707.7382, 1708.6910, 1753.8091, 1838.8954, 1860.8362, 1940.8932, 2022.8621, 2087.9259, 2225.0667, 2239.0815, 2285.9290, 2383.9100, 2398.9692, 2705.1276, 3312.3547

32. gi|153094414 **Mass:** 20328 **Score:** 54 **Expect:** 12 **Queries matched:** 4

ribosomal protein L5 [Mannheimia haemolytica PHL213]

Observed	Mr(expt)	Mr(calc)	ppm	Start	End	Miss	Ions	Peptide
882.5756	881.5684	881.5698	-1.66	103 -	110	0	40	R.LITIAVPR.I
1212.5840	1211.5767	1211.5757	0.81	1 -	9	1	---	-.MAKLHDYYR.D + Oxidation
1509.7278	1508.7205	1508.7399	-12.84	134 -	145	0	---	R.EQIIFPEIDYDK.V
1879.9133	1878.9060	1878.9363	-16.15	134 -	148	1	---	R.EQIIFPEIDYDKVDR.V

No match to: 807.3826, 827.4583, 832.4761, 856.5182, 870.5347, 897.4091, 973.5334, 986.5831, 993.5026, 1004.5915, 1016.5494, 1026.5340, 1029.5957, 1033.5294, 1037.5324, 1053.5472, 1055.6246, 1057.5248, 1060.5444, 1066.5116, 1074.5274, 1090.5346, 1092.4565, 1107.5419, 1109.4972, 1111.5759, 1122.5860, 1126.5648, 1132.6784, 1140.5689, 1150.6754, 1152.6441, 1157.5946, 1169.6688, 1184.6097, 1193.6197, 1198.6059, 1201.6200, 1228.6240, 1230.5958, 1234.6802, 1262.6625, 1263.7000, 1265.6524, 1267.6755, 1277.7100, 1290.6733, 1307.6697, 1308.6591, 1314.7606, 1320.5947, 1323.6645, 1329.6493, 1338.6690, 1341.6882, 1357.7032, 1365.6448, 1373.6597, 1379.7061, 1383.6844, 1390.6824, 1407.7186, 1424.7271, 1427.7832, 1434.7521, 1438.6321, 1458.7141, 1475.7546, 1487.7422, 1493.7259, 1541.7313, 1600.8150, 1657.7770, 1699.8072, 1707.7382, 1708.6910, 1753.8091, 1838.8954, 1860.8362, 1940.8932, 2022.8621, 2087.9259, 2225.0667, 2239.0815, 2285.9290, 2383.9100, 2398.9692, 2705.1276, 3312.3547

33. gi|117927621 **Mass:** 50434 **Score:** 54 **Expect:** 12 **Queries matched:** 7

cell envelope-related transcriptional attenuator [Acidothermus cellulolyticus 11B]

Observed	Mr(expt)	Mr(calc)	ppm	Start	End	Miss	Ions	Peptide
856.5182	855.5109	855.5178	-8.08	121 -	128	0	30	R.ATLVSLPR.D
986.5831	985.5759	985.5305	46.0	97 -	105	0	---	K.GQTQVLGQR.S
1263.7000	1262.6927	1262.7207	-22.22	117 -	128	1	---	K.GHGRATLVSLPR.D
1357.7032	1356.6959	1356.7613	-48.16	257 -	269	1	---	R.KVESAGTLTNPLK.L
1379.7061	1378.6989	1378.6962	1.96	246 -	256	1	---	K.RQQQFLAAMMR.K
1383.6844	1382.6771	1382.6799	-2.00	247 -	257	1	---	R.QQQFLAAMMRK.V + 2 Oxidat
1541.7313	1540.7240	1540.6749	31.9	280 -	294	0	---	K.SVTVDAGMSTADMEK.L

No match to: 807.3826, 827.4583, 832.4761, 870.5347, 882.5756, 897.4091, 973.5334, 993.5026, 1004.5915, 1016.5494, 1026.5340, 1029.5957, 1033.5294, 1037.5324, 1053.5472, 1055.6246, 1057.5248, 1060.5444, 1066.5116, 1074.5274, 1090.5346, 1092.4565, 1107.5419, 1109.4972, 1111.5759, 1122.5860, 1126.5648, 1132.6784, 1140.5689, 1150.6754, 1152.6441, 1157.5946, 1169.6688, 1184.6097, 1193.6197, 1198.6059, 1201.6200, 1212.5840, 1228.6240, 1230.5958, 1234.6802, 1262.6625, 1265.6524, 1267.6755, 1277.7100, 1290.6733, 1307.6697, 1308.6591, 1314.7606, 1320.5947, 1323.6645, 1329.6493, 1338.6690, 1341.6882, 1365.6448, 1373.6597, 1390.6824, 1407.7186, 1424.7271, 1427.7832, 1434.7521, 1438.6321, 1458.7141, 1475.7546, 1487.7422, 1493.7259, 1509.7278, 1600.8150, 1657.7770, 1699.8072, 1707.7382, 1708.6910, 1753.8091, 1838.8954, 1860.8362, 1879.9133, 1940.8932, 2022.8621, 2087.9259, 2225.0667, 2239.0815, 2285.9290, 2383.9100, 2398.9692, 2705.1276, 3312.3547

34. gi|39936301 **Mass:** 21139 **Score:** 52 **Expect:** 15 **Queries matched:** 6

50S ribosomal protein L5 [Rhodopseudomonas palustris CGA009]

Observed	Mr(expt)	Mr(calc)	ppm	Start	End	Miss	Ions	Peptide
882.5756	881.5684	881.5698	-1.66	109 -	116	0	33	R.LITVALPR.V
1037.5324	1036.5251	1036.5011	23.1	1 -	9	0	---	-.MAETAYVPR.L
1053.5472	1052.5399	1052.4961	41.6	1 -	9	0	---	-.MAETAYVPR.L + Oxidation
1090.5346	1089.5273	1089.5567	-26.94	122 -	131	1	---	R.GLNPKSFDGR.G
1424.7271	1423.7198	1423.7612	-29.11	174 -	185	1	---	R.ALLTAFNFPFRQ.-
1600.8150	1599.8078	1599.8403	-20.31	40 -	54	1	---	K.VVLNMGVGEAVNDRK.K

No match to: 807.3826, 827.4583, 832.4761, 856.5182, 870.5347, 897.4091, 973.5334, 986.5831, 993.5026, 1004.5915, 1016.5494, 1026.5340, 1029.5957, 1033.5294, 1055.6246, 1057.5248, 1060.5444, 1066.5116, 1074.5274, 1092.4565, 1107.5419, 1109.4972, 1111.5759, 1122.5860, 1126.5648, 1132.6784, 1140.5689, 1150.6754, 1152.6441, 1157.5946, 1169.6688,

1184.6097, 1193.6197, 1198.6059, 1201.6200, 1212.5840, 1228.6240, 1230.5958, 1234.6802, 1262.6625, 1263.7000, 1265.6524, 1267.6755, 1277.7100, 1290.6733, 1307.6697, 1308.6591, 1314.7606, 1320.5947, 1323.6645, 1329.6493, 1338.6690, 1341.6882, 1357.7032, 1365.6448, 1373.6597, 1379.7061, 1383.6844, 1390.6824, 1407.7186, 1427.7832, 1434.7521, 1438.6321, 1458.7141, 1475.7546, 1487.7422, 1493.7259, 1509.7278, 1541.7313, 1657.7770, 1699.8072, 1707.7382, 1708.6910, 1753.8091, 1838.8954, 1860.8362, 1879.9133, 1940.8932, 2022.8621, 2087.9259, 2225.0667, 2239.0815, 2285.9290, 2383.9100, 2398.9692, 2705.1276, 3312.3547

35. gi|23500984 **Mass:** 121626 **Score:** 52 **Expect:** 17 **Queries matched:** 14
DNA polymerase III subunit alpha [Brucella suis 1330]

Observed	Mr(expt)	Mr(calc)	ppm	Start		End	Miss	Ions	Peptide
870.5347	869.5274	869.5334	-6.92	562	-	569	0	30	K.LTLASLPR.E
1057.5248	1056.5176	1056.5134	3.91	845	-	852	1	---	R.TIEDMHRR.L
1060.5444	1059.5372	1059.5495	-11.65	334	-	342	1	---	R.EKGILCQGR.G
1201.6200	1200.6127	1200.6462	-27.90	830	-	839	1	---	K.RDTELLIADR.Q
1234.6802	1233.6729	1233.7306	-46.73	1014	-	1023	1	---	R.LIHIEGRIQR.S
1267.6755	1266.6682	1266.6721	-3.03	819	-	829	1	---	R.LGFRQIDGFSK.R
1314.7606	1313.7533	1313.6979	42.2	886	-	896	0	---	R.LPNDETLPLFR.A
1341.6882	1340.6809	1340.6659	11.2	61	-	71	1	---	K.ESGFRLFIGCR.L
1357.7032	1356.6959	1356.7085	-9.23	1054	-	1065	0	---	R.LIAPSQMAHHPR.N
1373.6597	1372.6525	1372.7034	-37.09	1054	-	1065	0	---	R.LIAPSQMAHHPR.N + Oxidat
1434.7521	1433.7448	1433.7990	-37.83	405	-	417	1	---	R.DRAAIVATVISYR.S
1458.7141	1457.7069	1457.6391	46.5	709	-	720	1	---	K.MIDGMVNRGYDR.T + 2 Oxid
1487.7422	1486.7350	1486.6874	32.0	391	-	401	1	---	R.REEVMQYVYDR.Y
1753.8091	1752.8018	1752.8067	-2.82	378	-	391	1	---	K.EPPDIDVDFEHERR.E

No match to: 807.3826, 827.4583, 832.4761, 856.5182, 882.5756, 897.4091, 973.5334, 986.5831, 993.5026, 1004.5915, 1016.5494, 1026.5340, 1029.5957, 1033.5294, 1037.5324, 1053.5472, 1055.6246, 1066.5116, 1074.5274, 1090.5346, 1092.4565, 1107.5419, 1109.4972, 1111.5759, 1122.5860, 1126.5648, 1132.6784, 1140.5689, 1150.6754, 1152.6441, 1157.5946, 1169.6688, 1184.6097, 1193.6197, 1198.6059, 1212.5840, 1228.6240, 1230.5958, 1262.6625, 1263.7000, 1265.6524, 1277.7100, 1290.6733, 1307.6697, 1308.6591, 1320.5947, 1323.6645, 1329.6493, 1338.6690, 1365.6448, 1379.7061, 1383.6844, 1390.6824, 1407.7186, 1424.7271, 1427.7832, 1438.6321, 1475.7546, 1493.7259, 1509.7278, 1541.7313, 1600.8150, 1657.7770, 1699.8072, 1707.7382, 1708.6910, 1838.8954, 1860.8362, 1879.9133, 1940.8932, 2022.8621,

2087.9259, 2225.0667, 2239.0815, 2285.9290, 2383.9100, 2398.9692, 2705.1276, 3312.3547

36. gi|42522173 **Mass:** 65055 **Score:** 52 **Expect:** 19 **Queries matched:** 12
histidine kinase [Bdellovibrio bacteriovorus HD100]

Observed	Mr(expt)	Mr(calc)	ppm	Start	End	Miss	Ions	Peptide
1029.5957	1028.5884	1028.5978	-9.18	120 -	128	1	---	K.VLAQSLRDK.R
1066.5116	1065.5043	1065.5342	-28.05	364 -	372	0	---	K.SLADEQIYK.F
1107.5419	1106.5346	1106.5356	-0.94	220 -	229	0	---	R.QSDFAQNLGK.I
1132.6784	1131.6711	1131.6288	37.4	512 -	521	0	---	K.EAVFALLVDR.I
1152.6441	1151.6369	1151.6121	21.5	502 -	511	1	---	R.NSSAVFIRMK.E
1263.7000	1262.6927	1262.6367	44.3	219 -	229	1	---	R.RQSDFAQNLGK.I
1277.7100	1276.7027	1276.7027	0.05	542 -	553	1	---	K.GFKGISILADEK.V
1357.7032	1356.6959	1356.7612	-48.13	351 -	363	1	---	R.AIKNALVTEEAAK.S
1373.6597	1372.6525	1372.6011	37.4	563 -	573	0	---	K.MMALMMFDQQK.H
1407.7186	1406.7114	1406.7592	-33.97	510 -	521	1	---	R.MKEAVFALLVDR.I + Oxidati
1427.7832	1426.7759	1426.7919	-11.20	37 -	48	0	---	K.EILEDLDNLILK.L
1708.6910	1707.6837	1707.7498	-38.69	198 -	210	0	---	K.NFFQMMNQTVNFR.V + 2 Oxid

No match to: 807.3826, 827.4583, 832.4761, 856.5182, 870.5347, 882.5756, 897.4091, 973.5334, 986.5831, 993.5026, 1004.5915, 1016.5494, 1026.5340, 1033.5294, 1037.5324, 1053.5472, 1055.6246, 1057.5248, 1060.5444, 1074.5274, 1090.5346, 1092.4565, 1109.4972, 1111.5759, 1122.5860, 1126.5648, 1140.5689, 1150.6754, 1157.5946, 1169.6688, 1184.6097, 1193.6197, 1198.6059, 1201.6200, 1212.5840, 1228.6240, 1230.5958, 1234.6802, 1262.6625, 1265.6524, 1267.6755, 1290.6733, 1307.6697, 1308.6591, 1314.7606, 1320.5947, 1323.6645, 1329.6493, 1338.6690, 1341.6882, 1365.6448, 1379.7061, 1383.6844, 1390.6824, 1424.7271, 1434.7521, 1438.6321, 1458.7141, 1475.7546, 1487.7422, 1493.7259, 1509.7278, 1541.7313, 1600.8150, 1657.7770, 1699.8072, 1707.7382, 1753.8091, 1838.8954, 1860.8362, 1879.9133, 1940.8932, 2022.8621, 2087.9259, 2225.0667, 2239.0815, 2285.9290, 2383.9100, 2398.9692, 2705.1276, 3312.3547

37. gi|120401397 **Mass:** 23486 **Score:** 52 **Expect:** 19 **Queries matched:** 3
transcriptional regulator, TetR family [Mycobacterium vanbaalenii PYR-1]

Observed	Mr(expt)	Mr(calc)	ppm	Start	End	Miss	Ions	Peptide
856.5182	855.5109	855.4814	34.4	203 -	210	0	40	R.AVDVISPR.R

1263.7000	1262.6927	1262.7095	-13.32	44 -	54	1	---	R.ALGVTRQTVYR.Y
1600.8150	1599.8078	1599.8005	4.54	103 -	116	0	---	R.LAGDHQFENLLTSR.R

No match to: 807.3826, 827.4583, 832.4761, 870.5347, 882.5756, 897.4091, 973.5334, 986.5831, 993.5026, 1004.5915, 1016.5494, 1026.5340, 1029.5957, 1033.5294, 1037.5324, 1053.5472, 1055.6246, 1057.5248, 1060.5444, 1066.5116, 1074.5274, 1090.5346, 1092.4565, 1107.5419, 1109.4972, 1111.5759, 1122.5860, 1126.5648, 1132.6784, 1140.5689, 1150.6754, 1152.6441, 1157.5946, 1169.6688, 1184.6097, 1193.6197, 1198.6059, 1201.6200, 1212.5840, 1228.6240, 1230.5958, 1234.6802, 1262.6625, 1265.6524, 1267.6755, 1277.7100, 1290.6733, 1307.6697, 1308.6591, 1314.7606, 1320.5947, 1323.6645, 1329.6493, 1338.6690, 1341.6882, 1357.7032, 1365.6448, 1373.6597, 1379.7061, 1383.6844, 1390.6824, 1407.7186, 1424.7271, 1427.7832, 1434.7521, 1438.6321, 1458.7141, 1475.7546, 1487.7422, 1493.7259, 1509.7278, 1541.7313, 1657.7770, 1699.8072, 1707.7382, 1708.6910, 1753.8091, 1838.8954, 1860.8362, 1879.9133, 1940.8932, 2022.8621, 2087.9259, 2225.0667, 2239.0815, 2285.9290, 2383.9100, 2398.9692, 2705.1276, 3312.3547

38. gi|62289055 **Mass:** 121631 **Score:** 52 **Expect:** 19 **Queries matched:** 14
DNA polymerase III subunit alpha [Brucella abortus biovar 1 str. 9-941]

Observed	Mr(expt)	Mr(calc)	ppm	Start	End	Miss	Ions	Peptide
870.5347	869.5274	869.5334	-6.92	562 -	569	0	30	K.LTLASLPR.K
1057.5248	1056.5176	1056.5134	3.91	845 -	852	1	---	R.TIEDMHRR.L
1060.5444	1059.5372	1059.5495	-11.65	334 -	342	1	---	R.EKGILCQGR.G
1201.6200	1200.6127	1200.6462	-27.90	830 -	839	1	---	K.RDTELLIADR.Q
1234.6802	1233.6729	1233.7306	-46.73	1014 -	1023	1	---	R.LIHIEGRIQR.S
1267.6755	1266.6682	1266.6721	-3.03	819 -	829	1	---	R.LGFRQIDGFSK.R
1314.7606	1313.7533	1313.6979	42.2	886 -	896	0	---	R.LPNDETLPLFR.A
1341.6882	1340.6809	1340.6659	11.2	61 -	71	1	---	K.ESGFRLFIGCR.L
1357.7032	1356.6959	1356.7085	-9.23	1054 -	1065	0	---	R.LIAPSQMAHHPR.N
1373.6597	1372.6525	1372.7034	-37.09	1054 -	1065	0	---	R.LIAPSQMAHHPR.N + Oxidat
1434.7521	1433.7448	1433.7990	-37.83	405 -	417	1	---	R.DRAAIVATVISYR.S
1458.7141	1457.7069	1457.6391	46.5	709 -	720	1	---	K.MIDGMVNRGYDR.T + 2 Oxid
1487.7422	1486.7350	1486.6874	32.0	391 -	401	1	---	R.REEVMQYVYDR.Y
1753.8091	1752.8018	1752.8067	-2.82	378 -	391	1	---	K.EPPDIDVDFEHERR.E

No match to: 807.3826, 827.4583, 832.4761, 856.5182, 882.5756, 897.4091, 973.5334, 986.5831, 993.5026, 1004.5915, 1016.5494, 1026.5340, 1029.5957, 1033.5294, 1037.5324,

1053.5472, 1055.6246, 1066.5116, 1074.5274, 1090.5346, 1092.4565, 1107.5419, 1109.4972, 1111.5759, 1122.5860, 1126.5648, 1132.6784, 1140.5689, 1150.6754, 1152.6441, 1157.5946, 1169.6688, 1184.6097, 1193.6197, 1198.6059, 1212.5840, 1228.6240, 1230.5958, 1262.6625, 1263.7000, 1265.6524, 1277.7100, 1290.6733, 1307.6697, 1308.6591, 1320.5947, 1323.6645, 1329.6493, 1338.6690, 1365.6448, 1379.7061, 1383.6844, 1390.6824, 1407.7186, 1424.7271, 1427.7832, 1438.6321, 1475.7546, 1493.7259, 1509.7278, 1541.7313, 1600.8150, 1657.7770, 1699.8072, 1707.7382, 1708.6910, 1838.8954, 1860.8362, 1879.9133, 1940.8932, 2022.8621, 2087.9259, 2225.0667, 2239.0815, 2285.9290, 2383.9100, 2398.9692, 2705.1276, 3312.3547

39. gi|120609238 **Mass:** 23478 **Score:** 51 **Expect:** 20 **Queries matched:** 4

hypothetical protein Aave_0537 [Acidovorax avenae subsp. citrulli AAC00-1]

Observed	Mr(expt)	Mr(calc)	ppm	Start		End	Miss	Ions	Peptide
870.5347	869.5274	869.4971	34.9	65	-	72	0	34	R.VVESALPR.L
1152.6441	1151.6369	1151.6775	-35.29	202	-	211	1	---	R.VVTLRQPPSR.-
1373.6597	1372.6525	1372.7059	-38.91	36	-	46	1	---	K.REQSLQDQITR.Q
1940.8932	1939.8859	1939.9824	-49.72	47	-	64	1	---	R.QLQALNGAVDNADRSVNR.V

No match to: 807.3826, 827.4583, 832.4761, 856.5182, 882.5756, 897.4091, 973.5334, 986.5831, 993.5026, 1004.5915, 1016.5494, 1026.5340, 1029.5957, 1033.5294, 1037.5324, 1053.5472, 1055.6246, 1057.5248, 1060.5444, 1066.5116, 1074.5274, 1090.5346, 1092.4565, 1107.5419, 1109.4972, 1111.5759, 1122.5860, 1126.5648, 1132.6784, 1140.5689, 1150.6754, 1157.5946, 1169.6688, 1184.6097, 1193.6197, 1198.6059, 1201.6200, 1212.5840, 1228.6240, 1230.5958, 1234.6802, 1262.6625, 1263.7000, 1265.6524, 1267.6755, 1277.7100, 1290.6733, 1307.6697, 1308.6591, 1314.7606, 1320.5947, 1323.6645, 1329.6493, 1338.6690, 1341.6882, 1357.7032, 1365.6448, 1379.7061, 1383.6844, 1390.6824, 1407.7186, 1424.7271, 1427.7832, 1434.7521, 1438.6321, 1458.7141, 1475.7546, 1487.7422, 1493.7259, 1509.7278, 1541.7313, 1600.8150, 1657.7770, 1699.8072, 1707.7382, 1708.6910, 1753.8091, 1838.8954, 1860.8362, 1879.9133, 2022.8621, 2087.9259, 2225.0667, 2239.0815, 2285.9290, 2383.9100, 2398.9692, 2705.1276, 3312.3547

40. gi|116189194 **Mass:** 9182 **Score:** 51 **Expect:** 23 **Queries matched:** 6

hypothetical protein VchoM_02001920 [Vibrio cholerae MO10]

Observed	Mr(expt)	Mr(calc)	ppm	Start		End	Miss	Ions	Peptide
1053.5472	1052.5399	1052.5655	-24.30	68	-	76	1	---	K.NKALADFFK.V
1057.5248	1056.5176	1056.5022	14.5	1	-	9	0	---	-.MAANQELHK.A + Oxidation

1066.5116	1065.5043	1065.5342	-28.06	59 -	67	0	---	R.QLATEEFTK.N
1234.6802	1233.6729	1233.6870	-11.41	70 -	80	1	---	K.ALADFFKVPAR.K
1308.6591	1307.6519	1307.6721	-15.47	59 -	69	1	---	R.QLATEEFTKNK.A
1657.7770	1656.7697	1656.8439	-44.80	1 -	14	1	---	-.MAANQELHKAMLLR.E + 2 Oxi

No match to: 807.3826, 827.4583, 832.4761, 856.5182, 870.5347, 882.5756, 897.4091, 973.5334, 986.5831, 993.5026, 1004.5915, 1016.5494, 1026.5340, 1029.5957, 1033.5294, 1037.5324, 1055.6246, 1060.5444, 1074.5274, 1090.5346, 1092.4565, 1107.5419, 1109.4972, 1111.5759, 1122.5860, 1126.5648, 1132.6784, 1140.5689, 1150.6754, 1152.6441, 1157.5946, 1169.6688, 1184.6097, 1193.6197, 1198.6059, 1201.6200, 1212.5840, 1228.6240, 1230.5958, 1262.6625, 1263.7000, 1265.6524, 1267.6755, 1277.7100, 1290.6733, 1307.6697, 1314.7606, 1320.5947, 1323.6645, 1329.6493, 1338.6690, 1341.6882, 1357.7032, 1365.6448, 1373.6597, 1379.7061, 1383.6844, 1390.6824, 1407.7186, 1424.7271, 1427.7832, 1434.7521, 1438.6321, 1458.7141, 1475.7546, 1487.7422, 1493.7259, 1509.7278, 1541.7313, 1600.8150, 1699.8072, 1707.7382, 1708.6910, 1753.8091, 1838.8954, 1860.8362, 1879.9133, 1940.8932, 2022.8621, 2087.9259, 2225.0667, 2239.0815, 2285.9290, 2383.9100, 2398.9692, 2705.1276, 3312.3547

41. gi|23499837 **Mass:** 45741 **Score:** 51 **Expect:** 23 **Queries matched:** 11

transglycosylase, putative [Brucella suis 1330]

Observed	Mr(expt)	Mr(calc)	ppm	Start	End	Miss	Ions	Peptide
807.3826	806.3753	806.3705	5.95	146 -	151	1	---	K.RDDVMR.D + Oxidation (M)
973.5334	972.5262	972.4930	34.1	112 -	118	0	---	K.WGQWLQR.I
993.5026	992.4953	992.5331	-38.06	4 -	11	1	---	R.FPFTPEKK.L
1033.5294	1032.5221	1032.5352	-12.69	380 -	388	0	---	R.AAIEAFQQR.N
1090.5346	1089.5273	1089.5237	3.36	147 -	155	1	---	R.DDVMRDAIR.S
1265.6524	1264.6451	1264.6638	-14.80	1 -	10	1	---	-.MLRFPFTPEK.K
1267.6755	1266.6682	1266.6754	-5.67	170 -	180	1	---	K.YGRTQLIAAMK.I + Oxidatio
1307.6697	1306.6624	1306.6993	-28.21	156 -	167	1	---	R.SLATLAYADARR.A
1338.6690	1337.6618	1337.6476	10.6	281 -	292	1	---	R.ANGRAFPDPHEK.A
1379.7061	1378.6989	1378.6703	20.7	250 -	261	0	---	R.TWGYEVMLPAGR.K
1424.7271	1423.7198	1423.7208	-0.73	54 -	66	1	---	K.SGIAPSTFDRAFR.G

No match to: 827.4583, 832.4761, 856.5182, 870.5347, 882.5756, 897.4091, 986.5831, 1004.5915, 1016.5494, 1026.5340, 1029.5957, 1037.5324, 1053.5472, 1055.6246, 1057.5248, 1060.5444, 1066.5116, 1074.5274, 1092.4565, 1107.5419, 1109.4972, 1111.5759, 1122.5860, 1126.5648, 1132.6784, 1140.5689, 1150.6754, 1152.6441, 1157.5946, 1169.6688, 1184.6097,

1193.6197, 1198.6059, 1201.6200, 1212.5840, 1228.6240, 1230.5958, 1234.6802, 1262.6625, 1263.7000, 1277.7100, 1290.6733, 1308.6591, 1314.7606, 1320.5947, 1323.6645, 1329.6493, 1341.6882, 1357.7032, 1365.6448, 1373.6597, 1383.6844, 1390.6824, 1407.7186, 1427.7832, 1434.7521, 1438.6321, 1458.7141, 1475.7546, 1487.7422, 1493.7259, 1509.7278, 1541.7313, 1600.8150, 1657.7770, 1699.8072, 1707.7382, 1708.6910, 1753.8091, 1838.8954, 1860.8362, 1879.9133, 1940.8932, 2022.8621, 2087.9259, 2225.0667, 2239.0815, 2285.9290, 2383.9100, 2398.9692, 2705.1276, 3312.3547

42. gi|62317029 **Mass:** 46080 **Score:** 50 **Expect:** 24 **Queries matched:** 11

hypothetical transglycosylase [Brucella abortus biovar 1 str. 9-941]

Observed	Mr(expt)	Mr(calc)	ppm	Start	End	Miss	Ions	Peptide
807.3826	806.3753	806.3705	5.95	146	- 151	1	---	K.RDDVMR.D + Oxidation (M)
973.5334	972.5262	972.4930	34.1	112	- 118	0	---	K.WGQWLQR.I
993.5026	992.4953	992.5331	-38.06	4	- 11	1	---	R.FPFTPEKK.L
1033.5294	1032.5221	1032.5352	-12.69	384	- 392	0	---	R.AAIEAFQQR.N
1090.5346	1089.5273	1089.5237	3.36	147	- 155	1	---	R.DDVMRDAIR.S
1265.6524	1264.6451	1264.6638	-14.80	1	- 10	1	---	-.MLRFPFTPEK.K
1267.6755	1266.6682	1266.6754	-5.67	170	- 180	1	---	K.YGRTQLIAAMK.I + Oxidatio
1307.6697	1306.6624	1306.6993	-28.21	156	- 167	1	---	R.SLATLAYADARR.A
1338.6690	1337.6618	1337.6476	10.6	281	- 292	1	---	R.ANGRAFPDPHEK.A
1379.7061	1378.6989	1378.6703	20.7	250	- 261	0	---	R.TWGYEVMLPAGR.K
1424.7271	1423.7198	1423.7208	-0.73	54	- 66	1	---	K.SGIAPSTFDRAFR.G

No match to: 827.4583, 832.4761, 856.5182, 870.5347, 882.5756, 897.4091, 986.5831, 1004.5915, 1016.5494, 1026.5340, 1029.5957, 1037.5324, 1053.5472, 1055.6246, 1057.5248, 1060.5444, 1066.5116, 1074.5274, 1092.4565, 1107.5419, 1109.4972, 1111.5759, 1122.5860, 1126.5648, 1132.6784, 1140.5689, 1150.6754, 1152.6441, 1157.5946, 1169.6688, 1184.6097, 1193.6197, 1198.6059, 1201.6200, 1212.5840, 1228.6240, 1230.5958, 1234.6802, 1262.6625, 1263.7000, 1277.7100, 1290.6733, 1308.6591, 1314.7606, 1320.5947, 1323.6645, 1329.6493, 1341.6882, 1357.7032, 1365.6448, 1373.6597, 1383.6844, 1390.6824, 1407.7186, 1427.7832, 1434.7521, 1438.6321, 1458.7141, 1475.7546, 1487.7422, 1493.7259, 1509.7278, 1541.7313, 1600.8150, 1657.7770, 1699.8072, 1707.7382, 1708.6910, 1753.8091, 1838.8954, 1860.8362, 1879.9133, 1940.8932, 2022.8621, 2087.9259, 2225.0667, 2239.0815, 2285.9290, 2383.9100, 2398.9692, 2705.1276, 3312.3547

43. gi|148558066 **Mass:** 46110 **Score:** 50 **Expect:** 24 **Queries matched:** 11

putative transglycosylase [Brucella ovis ATCC 25840]

Observed	Mr(expt)	Mr(calc)	ppm	Start	End	Miss	Ions	Peptide
807.3826	806.3753	806.3705	5.95	146 -	151	1	---	K.RDDVMR.D + Oxidation (M)
973.5334	972.5262	972.4930	34.1	112 -	118	0	---	K.WGQWLQR.I
993.5026	992.4953	992.5331	-38.06	4 -	11	1	---	R.FPFTPEKK.L
1033.5294	1032.5221	1032.5352	-12.69	384 -	392	0	---	R.AAIEAFQQR.N
1090.5346	1089.5273	1089.5237	3.36	147 -	155	1	---	R.DDVMRDAIR.S
1265.6524	1264.6451	1264.6638	-14.80	1 -	10	1	---	-.MLRFPFTPEK.K
1267.6755	1266.6682	1266.6754	-5.67	170 -	180	1	---	K.YGRTQLIAAMK.I + Oxidatio
1307.6697	1306.6624	1306.6993	-28.21	156 -	167	1	---	R.SLATLAYADARR.A
1338.6690	1337.6618	1337.6476	10.6	281 -	292	1	---	R.ANGRAFPDPHEK.A
1379.7061	1378.6989	1378.6703	20.7	250 -	261	0	---	R.TWGYEVMLPAGR.K
1424.7271	1423.7198	1423.7208	-0.73	54 -	66	1	---	K.SGIAPSTFDRAFR.G

No match to: 827.4583, 832.4761, 856.5182, 870.5347, 882.5756, 897.4091, 986.5831, 1004.5915, 1016.5494, 1026.5340, 1029.5957, 1037.5324, 1053.5472, 1055.6246, 1057.5248, 1060.5444, 1066.5116, 1074.5274, 1092.4565, 1107.5419, 1109.4972, 1111.5759, 1122.5860, 1126.5648, 1132.6784, 1140.5689, 1150.6754, 1152.6441, 1157.5946, 1169.6688, 1184.6097, 1193.6197, 1198.6059, 1201.6200, 1212.5840, 1228.6240, 1230.5958, 1234.6802, 1262.6625, 1263.7000, 1277.7100, 1290.6733, 1308.6591, 1314.7606, 1320.5947, 1323.6645, 1329.6493, 1341.6882, 1357.7032, 1365.6448, 1373.6597, 1383.6844, 1390.6824, 1407.7186, 1427.7832, 1434.7521, 1438.6321, 1458.7141, 1475.7546, 1487.7422, 1493.7259, 1509.7278, 1541.7313, 1600.8150, 1657.7770, 1699.8072, 1707.7382, 1708.6910, 1753.8091, 1838.8954, 1860.8362, 1879.9133, 1940.8932, 2022.8621, 2087.9259, 2225.0667, 2239.0815, 2285.9290, 2383.9100, 2398.9692, 2705.1276, 3312.3547

44. gi|69247181 **Mass:** 20177 **Score:** 50 **Expect:** 25 **Queries matched:** 4

Ribosomal protein L5 [Enterococcus faecium DO]

Observed	Mr(expt)	Mr(calc)	ppm	Start	End	Miss	Ions	Peptide
870.5347	869.5274	869.5334	-6.94	103 -	110	0	38	K.LVSVSLPR.V
1033.5294	1032.5221	1032.5161	5.80	11 -	19	0	---	K.EVTPSLMEK.F
1487.7422	1486.7350	1486.7814	-31.20	34 -	48	0	---	K.IVINMGVGDAVSNAK.N
1657.7770	1656.7697	1656.8181	-29.23	20 -	33	1	---	K.FNYSSVMQTPKVEK.I

No match to: 807.3826, 827.4583, 832.4761, 856.5182, 882.5756, 897.4091, 973.5334, 986.5831, 993.5026, 1004.5915, 1016.5494, 1026.5340, 1029.5957, 1037.5324, 1053.5472, 1055.6246, 1057.5248, 1060.5444, 1066.5116, 1074.5274, 1090.5346, 1092.4565, 1107.5419, 1109.4972, 1111.5759, 1122.5860, 1126.5648, 1132.6784, 1140.5689, 1150.6754, 1152.6441, 1157.5946, 1169.6688, 1184.6097, 1193.6197, 1198.6059, 1201.6200, 1212.5840, 1228.6240, 1230.5958, 1234.6802, 1262.6625, 1263.7000, 1265.6524, 1267.6755, 1277.7100, 1290.6733, 1307.6697, 1308.6591, 1314.7606, 1320.5947, 1323.6645, 1329.6493, 1338.6690, 1341.6882, 1357.7032, 1365.6448, 1373.6597, 1379.7061, 1383.6844, 1390.6824, 1407.7186, 1424.7271, 1427.7832, 1434.7521, 1438.6321, 1458.7141, 1475.7546, 1493.7259, 1509.7278, 1541.7313, 1600.8150, 1699.8072, 1707.7382, 1708.6910, 1753.8091, 1838.8954, 1860.8362, 1879.9133, 1940.8932, 2022.8621, 2087.9259, 2225.0667, 2239.0815, 2285.9290, 2383.9100, 2398.9692, 2705.1276, 3312.3547

45. gi|110635158 **Mass:** 42059 **Score:** 50 **Expect:** 25 **Queries matched:** 10

beta-ketoadipyl CoA thiolase [Mesorhizobium sp. BNC1]

Observed	Mr(expt)	Mr(calc)	ppm	Start		End	Miss	Ions	Peptide
870.5347	869.5274	869.4858	47.8	215	-	222	0	---	K.ADPIIVDK.D
1026.5340	1025.5267	1025.5757	-47.75	26	-	35	0	---	R.ADDLAAIPLK.A
1057.5248	1056.5176	1056.5638	-43.71	294	-	304	0	---	R.IMGIGPAPASK.K + Oxidatio
1169.6688	1168.6615	1168.6638	-1.98	294	-	305	1	---	R.IMGIGPAPASKK.L
1365.6448	1364.6375	1364.6684	-22.65	223	-	234	0	---	K.DEHPRPGTTVEK.L
1373.6597	1372.6525	1372.6695	-12.40	188	-	200	1	---	R.SQDKAVAAQENGR.L
1379.7061	1378.6989	1378.6333	47.5	89	-	102	0	---	R.LCGSGMDAVATAAR.A
1390.6824	1389.6751	1389.6057	50.0	1	-	11	0	---	-.MAEAFICDYVR.T + Oxidatio
1438.6321	1437.6248	1437.6888	-44.55	137	-	148	0	---	R.NAEIYDTTIGWR.F
1707.7382	1706.7310	1706.7967	-38.53	106	-	122	0	---	K.AGEAELMIAGGVESMSR.A

No match to: 807.3826, 827.4583, 832.4761, 856.5182, 882.5756, 897.4091, 973.5334, 986.5831, 993.5026, 1004.5915, 1016.5494, 1029.5957, 1033.5294, 1037.5324, 1053.5472, 1055.6246, 1060.5444, 1066.5116, 1074.5274, 1090.5346, 1092.4565, 1107.5419, 1109.4972, 1111.5759, 1122.5860, 1126.5648, 1132.6784, 1140.5689, 1150.6754, 1152.6441, 1157.5946, 1184.6097, 1193.6197, 1198.6059, 1201.6200, 1212.5840, 1228.6240, 1230.5958, 1234.6802, 1262.6625, 1263.7000, 1265.6524, 1267.6755, 1277.7100, 1290.6733, 1307.6697, 1308.6591, 1314.7606, 1320.5947, 1323.6645, 1329.6493, 1338.6690, 1341.6882, 1357.7032, 1383.6844, 1407.7186, 1424.7271, 1427.7832, 1434.7521, 1458.7141, 1475.7546, 1487.7422, 1493.7259,

1509.7278, 1541.7313, 1600.8150, 1657.7770, 1699.8072, 1708.6910, 1753.8091, 1838.8954, 1860.8362, 1879.9133, 1940.8932, 2022.8621, 2087.9259, 2225.0667, 2239.0815, 2285.9290, 2383.9100, 2398.9692, 2705.1276, 3312.3547

46. gi|28493548 **Mass:** 35163 **Score:** 50 **Expect:** 27 **Queries matched:** 10
FMN adenylyltransferase [Tropheryma whipplei str. Twist]

Observed	Mr(expt)	Mr(calc)	ppm	Start	End	Miss	Ions	Peptide
1004.5915	1003.5842	1003.5662	17.9	159 -	167	0	---	K.TNISSTLIR.K
1016.5494	1015.5421	1015.5410	1.03	294 -	302	1	---	K.AAIRSDVER.C
1037.5324	1036.5251	1036.5203	4.66	23 -	31	0	---	K.FDGVHLGHR.R
1132.6784	1131.6711	1131.6611	8.83	159 -	168	1	---	K.TNISSTLIRK.F
1193.6197	1192.6124	1192.6214	-7.52	23 -	32	1	---	K.FDGVHLGHRR.L
1307.6697	1306.6624	1306.7173	-41.96	143 -	153	0	---	R.ELGFYLEVIPK.I
1308.6591	1307.6519	1307.6405	8.70	185 -	196	0	---	R.NHVCTGTVVHGK.K
1424.7271	1423.7198	1423.7130	4.79	113 -	125	0	---	K.LNMSSIVIGDGFR.F + Oxidat
1493.7259	1492.7186	1492.7708	-34.95	126 -	139	1	---	R.FGARGLGDAMLLEK.L + Oxida
1541.7313	1540.7240	1540.6902	22.0	63 -	75	0	---	K.DMSFAPLCSLEQK.L + Oxidat

No match to: 807.3826, 827.4583, 832.4761, 856.5182, 870.5347, 882.5756, 897.4091, 973.5334, 986.5831, 993.5026, 1026.5340, 1029.5957, 1033.5294, 1053.5472, 1055.6246, 1057.5248, 1060.5444, 1066.5116, 1074.5274, 1090.5346, 1092.4565, 1107.5419, 1109.4972, 1111.5759, 1122.5860, 1126.5648, 1140.5689, 1150.6754, 1152.6441, 1157.5946, 1169.6688, 1184.6097, 1198.6059, 1201.6200, 1212.5840, 1228.6240, 1230.5958, 1234.6802, 1262.6625, 1263.7000, 1265.6524, 1267.6755, 1277.7100, 1290.6733, 1314.7606, 1320.5947, 1323.6645, 1329.6493, 1338.6690, 1341.6882, 1357.7032, 1365.6448, 1373.6597, 1379.7061, 1383.6844, 1390.6824, 1407.7186, 1427.7832, 1434.7521, 1438.6321, 1458.7141, 1475.7546, 1487.7422, 1509.7278, 1600.8150, 1657.7770, 1699.8072, 1707.7382, 1708.6910, 1753.8091, 1838.8954, 1860.8362, 1879.9133, 1940.8932, 2022.8621, 2087.9259, 2225.0667, 2239.0815, 2285.9290, 2383.9100, 2398.9692, 2705.1276, 3312.3547

47. gi|126358412 **Mass:** 40266 **Score:** 50 **Expect:** 27 **Queries matched:** 9
putative iron-sulfur cluster binding protein [Pseudomonas putida GB-1]

Observed	Mr(expt)	Mr(calc)	ppm	Start	End	Miss	Ions	Peptide
1004.5915	1003.5842	1003.5563	27.8	118 -	125	1	---	K.RVQFLADR.I
1074.5274	1073.5201	1073.5465	-24.58	333 -	341	1	---	R.REDASELVR.E

1122.5860	1121.5787	1121.5618	15.1	105 - 113	1	---		R.YALGRDYHK.L
1169.6688	1168.6615	1168.6353	22.4	100 - 109	1	---		K.AYISRYALGR.D
1267.6755	1266.6682	1266.6642	3.19	221 - 230	0	---		R.CISYLTIELR.G
1475.7546	1474.7473	1474.8005	-36.02	63 - 75	1	---		K.RSHPDQLIPGTVR.V
1541.7313	1540.7240	1540.6650	38.3	81 - 93	0	---		R.MDYLPGDTQMAQR.L + Oxidat
2383.9100	2382.9028	2383.0079	-44.11	241 - 259	1	---		K.MGNRVFGCDDCQIVCPWNR.F
3312.3547	3311.3474	3311.5054	-47.71	263 - 289	1	---		K.HSQEQDFQPRHGLENAELAEMFLW

No match to: 807.3826, 827.4583, 832.4761, 856.5182, 870.5347, 882.5756, 897.4091, 973.5334, 986.5831, 993.5026, 1016.5494, 1026.5340, 1029.5957, 1033.5294, 1037.5324, 1053.5472, 1055.6246, 1057.5248, 1060.5444, 1066.5116, 1090.5346, 1092.4565, 1107.5419, 1109.4972, 1111.5759, 1126.5648, 1132.6784, 1140.5689, 1150.6754, 1152.6441, 1157.5946, 1184.6097, 1193.6197, 1198.6059, 1201.6200, 1212.5840, 1228.6240, 1230.5958, 1234.6802, 1262.6625, 1263.7000, 1265.6524, 1277.7100, 1290.6733, 1307.6697, 1308.6591, 1314.7606, 1320.5947, 1323.6645, 1329.6493, 1338.6690, 1341.6882, 1357.7032, 1365.6448, 1373.6597, 1379.7061, 1383.6844, 1390.6824, 1407.7186, 1424.7271, 1427.7832, 1434.7521, 1438.6321, 1458.7141, 1487.7422, 1493.7259, 1509.7278, 1600.8150, 1657.7770, 1699.8072, 1707.7382, 1708.6910, 1753.8091, 1838.8954, 1860.8362, 1879.9133, 1940.8932, 2022.8621, 2087.9259, 2225.0667, 2239.0815, 2285.9290, 2398.9692, 2705.1276

48. gi|91977640 **Mass:** 21075 **Score:** 50 **Expect:** 28 **Queries matched:** 6
ribosomal protein L5 [Rhodopseudomonas palustris BisB5]

Observed	Mr(expt)	Mr(calc)	ppm	Start	End	Miss	Ions	Peptide
882.5756	881.5684	881.5698	-1.66	109 -	116	0	33	R.LITVALPR.V
1037.5324	1036.5251	1036.5011	23.1	1 -	9	0	---	-.MAETAYVPR.L
1053.5472	1052.5399	1052.4961	41.6	1 -	9	0	---	-.MAETAYVPR.L + Oxidation
1090.5346	1089.5273	1089.5567	-26.94	122 -	131	1	---	R.GLNPKSFDGR.G
1267.6755	1266.6682	1266.6179	39.7	26 -	36	0	---	K.FGYGNVMQVPR.L
1424.7271	1423.7198	1423.7612	-29.11	174 -	185	1	---	R.ALLTAFNFPFRQ.-

No match to: 807.3826, 827.4583, 832.4761, 856.5182, 870.5347, 897.4091, 973.5334, 986.5831, 993.5026, 1004.5915, 1016.5494, 1026.5340, 1029.5957, 1033.5294, 1055.6246, 1057.5248, 1060.5444, 1066.5116, 1074.5274, 1092.4565, 1107.5419, 1109.4972, 1111.5759, 1122.5860, 1126.5648, 1132.6784, 1140.5689, 1150.6754, 1152.6441, 1157.5946, 1169.6688, 1184.6097, 1193.6197, 1198.6059, 1201.6200, 1212.5840, 1228.6240, 1230.5958, 1234.6802, 1262.6625, 1263.7000, 1265.6524, 1277.7100, 1290.6733, 1307.6697, 1308.6591, 1314.7606,

1320.5947, 1323.6645, 1329.6493, 1338.6690, 1341.6882, 1357.7032, 1365.6448, 1373.6597, 1379.7061, 1383.6844, 1390.6824, 1407.7186, 1427.7832, 1434.7521, 1438.6321, 1458.7141, 1475.7546, 1487.7422, 1493.7259, 1509.7278, 1541.7313, 1600.8150, 1657.7770, 1699.8072, 1707.7382, 1708.6910, 1753.8091, 1838.8954, 1860.8362, 1879.9133, 1940.8932, 2022.8621, 2087.9259, 2225.0667, 2239.0815, 2285.9290, 2383.9100, 2398.9692, 2705.1276, 3312.3547

49. gi|17988368 **Mass:** 46094 **Score:** 50 **Expect:** 28 **Queries matched:** 11
MEMBRANE-BOUND LYTIC MUREIN TRANSGLYCOSYLASE B [Brucella melitensis 16M]

Observed	Mr(expt)	Mr(calc)	ppm	Start	End	Miss	Ions	Peptide
807.3826	806.3753	806.3705	5.95	146 -	151	1	---	K.RDDVMR.D + Oxidation (M)
973.5334	972.5262	972.4930	34.1	112 -	118	0	---	K.WGQWLQR.I
993.5026	992.4953	992.5331	-38.06	4 -	11	1	---	R.FPFTPEKK.L
1033.5294	1032.5221	1032.5352	-12.69	384 -	392	0	---	R.AALEAFQQR.N
1090.5346	1089.5273	1089.5237	3.36	147 -	155	1	---	R.DDVMRDAIR.S
1265.6524	1264.6451	1264.6638	-14.80	1 -	10	1	---	-.MLRFPFTPEK.K
1267.6755	1266.6682	1266.6754	-5.67	170 -	180	1	---	K.YGRTQLIAAMK.I + Oxidatio
1307.6697	1306.6624	1306.6993	-28.21	156 -	167	1	---	R.SLATLAYADARR.A
1338.6690	1337.6618	1337.6476	10.6	281 -	292	1	---	R.ANGRAFPDPHEK.A
1379.7061	1378.6989	1378.6703	20.7	250 -	261	0	---	R.TWGYEVMLPAGR.K
1424.7271	1423.7198	1423.7208	-0.73	54 -	66	1	---	K.SGIAPSTFDRAFR.G

No match to: 827.4583, 832.4761, 856.5182, 870.5347, 882.5756, 897.4091, 986.5831, 1004.5915, 1016.5494, 1026.5340, 1029.5957, 1037.5324, 1053.5472, 1055.6246, 1057.5248, 1060.5444, 1066.5116, 1074.5274, 1092.4565, 1107.5419, 1109.4972, 1111.5759, 1122.5860, 1126.5648, 1132.6784, 1140.5689, 1150.6754, 1152.6441, 1157.5946, 1169.6688, 1184.6097, 1193.6197, 1198.6059, 1201.6200, 1212.5840, 1228.6240, 1230.5958, 1234.6802, 1262.6625, 1263.7000, 1277.7100, 1290.6733, 1308.6591, 1314.7606, 1320.5947, 1323.6645, 1329.6493, 1341.6882, 1357.7032, 1365.6448, 1373.6597, 1383.6844, 1390.6824, 1407.7186, 1427.7832, 1434.7521, 1438.6321, 1458.7141, 1475.7546, 1487.7422, 1493.7259, 1509.7278, 1541.7313, 1600.8150, 1657.7770, 1699.8072, 1707.7382, 1708.6910, 1753.8091, 1838.8954, 1860.8362, 1879.9133, 1940.8932, 2022.8621, 2087.9259, 2225.0667, 2239.0815, 2285.9290, 2383.9100, 2398.9692, 2705.1276, 3312.3547

50. gi|85714293 **Mass:** 17896 **Score:** 50 **Expect:** 28 **Queries matched:** 7
glutathione peroxidase [Nitrobacter sp. Nb-311A]

Observed	Mr(expt)	Mr(calc)	ppm	Start	End	Miss	Ions	Peptide
897.4091	896.4018	896.4352	-37.22	44 -	50	0	---	K.DLEDLHR.T
993.5026	992.4953	992.5403	-45.32	141 -	149	1	---	R.HAPTTNPKK.L
1004.5915	1003.5842	1003.5563	27.8	129 -	136	1	---	K.FLLDRQGR.V
1029.5957	1028.5884	1028.6117	-22.69	150 -	158	1	---	K.LTEKIEALL.-
1262.6625	1261.6553	1261.6085	37.1	9 -	20	0	---	R.ANTLAGESCALR.Q
1290.6733	1289.6660	1289.7204	-42.21	137 -	148	1	---	R.VVARHAPTTNPK.K
1879.9133	1878.9060	1878.9006	2.85	9 -	25	1	---	R.ANTLAGESCALRQFEGR.V

No match to: 807.3826, 827.4583, 832.4761, 856.5182, 870.5347, 882.5756, 973.5334, 986.5831, 1016.5494, 1026.5340, 1033.5294, 1037.5324, 1053.5472, 1055.6246, 1057.5248, 1060.5444, 1066.5116, 1074.5274, 1090.5346, 1092.4565, 1107.5419, 1109.4972, 1111.5759, 1122.5860, 1126.5648, 1132.6784, 1140.5689, 1150.6754, 1152.6441, 1157.5946, 1169.6688, 1184.6097, 1193.6197, 1198.6059, 1201.6200, 1212.5840, 1228.6240, 1230.5958, 1234.6802, 1263.7000, 1265.6524, 1267.6755, 1277.7100, 1307.6697, 1308.6591, 1314.7606, 1320.5947, 1323.6645, 1329.6493, 1338.6690, 1341.6882, 1357.7032, 1365.6448, 1373.6597, 1379.7061, 1383.6844, 1390.6824, 1407.7186, 1424.7271, 1427.7832, 1434.7521, 1438.6321, 1458.7141, 1475.7546, 1487.7422, 1493.7259, 1509.7278, 1541.7313, 1600.8150, 1657.7770, 1699.8072, 1707.7382, 1708.6910, 1753.8091, 1838.8954, 1860.8362, 1940.8932, 2022.8621, 2087.9259, 2225.0667, 2239.0815, 2285.9290, 2383.9100, 2398.9692, 2705.1276, 3312.3547

Search Parameters

Type of search : MS/MS Ion Search
Enzyme : Trypsin
Fixed modifications : Carbamidomethyl (C)
Variable modifications : Oxidation (M)
Mass values : Monoisotopic
Protein Mass : Unrestricted
Peptide Mass Tolerance : ± 50 ppm
Fragment Mass Tolerance: ± 0.5 Da
Max Missed Cleavages : 1
Instrument type : MALDI-TOF-TOF
Query1 (807.3826,1+) : <no title>
Query2 (827.4583,1+) : <no title>
Query3 (832.4761,1+) : <no title>

```
Query4 (856.5182,1+)   : <no title>
Query5 (870.5347,1+)   : <no title>
Query6 (882.5756,1+)   : <no title>
Query7 (897.4091,1+)   : <no title>
Query8 (973.5334,1+)   : <no title>
Query9 (986.5831,1+)   : <no title>
Query10 (993.5026,1+)  : <no title>
Query11 (1004.5915,1+) : <no title>
Query12 (1016.5494,1+) : <no title>
Query13 (1026.5340,1+) : <no title>
Query14 (1029.5957,1+) : <no title>
Query15 (1033.5294,1+) : <no title>
Query16 (1037.5324,1+) : <no title>
Query17 (1053.5472,1+) : <no title>
Query18 (1055.6246,1+) : <no title>
Query19 (1057.5248,1+) : <no title>
Query20 (1060.5444,1+) : <no title>
Query21 (1066.5116,1+) : <no title>
Query22 (1074.5274,1+) : <no title>
Query23 (1090.5346,1+) : <no title>
Query24 (1092.4565,1+) : <no title>
Query25 (1107.5419,1+) : <no title>
Query26 (1109.4972,1+) : <no title>
Query27 (1111.5759,1+) : <no title>
Query28 (1122.5860,1+) : <no title>
Query29 (1126.5648,1+) : <no title>
Query30 (1132.6784,1+) : <no title>
Query31 (1140.5689,1+) : <no title>
Query32 (1150.6754,1+) : <no title>
Query33 (1152.6441,1+) : <no title>
Query34 (1157.5946,1+) : <no title>
Query35 (1169.6688,1+) : <no title>
Query36 (1184.6097,1+) : <no title>
Query37 (1193.6197,1+) : <no title>
Query38 (1198.6059,1+) : <no title>
Query39 (1201.6200,1+) : <no title>
Query40 (1212.5840,1+) : <no title>
```

```
Query41 (1228.6240,1+) : <no title>
Query42 (1230.5958,1+) : <no title>
Query43 (1234.6802,1+) : <no title>
Query44 (1262.6625,1+) : <no title>
Query45 (1263.7000,1+) : <no title>
Query46 (1265.6524,1+) : <no title>
Query47 (1267.6755,1+) : <no title>
Query48 (1277.7100,1+) : <no title>
Query49 (1290.6733,1+) : <no title>
Query50 (1307.6697,1+) : <no title>
Query51 (1308.6591,1+) : <no title>
Query52 (1314.7606,1+) : <no title>
Query53 (1320.5947,1+) : <no title>
Query54 (1323.6645,1+) : <no title>
Query55 (1329.6493,1+) : <no title>
Query56 (1338.6690,1+) : <no title>
Query57 (1341.6882,1+) : <no title>
Query58 (1357.7032,1+) : <no title>
Query59 (1365.6448,1+) : <no title>
Query60 (1373.6597,1+) : <no title>
Query61 (1379.7061,1+) : <no title>
Query62 (1383.6844,1+) : <no title>
Query63 (1390.6824,1+) : <no title>
Query64 (1407.7186,1+) : <no title>
Query65 (1424.7271,1+) : <no title>
Query66 (1427.7832,1+) : <no title>
Query67 (1434.7521,1+) : <no title>
Query68 (1438.6321,1+) : <no title>
Query69 (1458.7141,1+) : <no title>
Query70 (1475.7546,1+) : <no title>
Query71 (1487.7422,1+) : <no title>
Query72 (1493.7259,1+) : <no title>
Query73 (1509.7278,1+) : <no title>
Query74 (1541.7313,1+) : <no title>
Query75 (1600.8150,1+) : <no title>
Query76 (1657.7770,1+) : <no title>
Query77 (1699.8072,1+) : <no title>
```

```
Query78 (1707.7382,1+) : <no title>
Query79 (1708.6910,1+) : <no title>
Query80 (1753.8091,1+) : <no title>
Query81 (1838.8954,1+) : <no title>
Query82 (1860.8362,1+) : <no title>
Query83 (1879.9133,1+) : <no title>
Query84 (1940.8932,1+) : <no title>
Query85 (2022.8621,1+) : <no title>
Query86 (2087.9259,1+) : <no title>
Query87 (2225.0667,1+) : <no title>
Query88 (2239.0815,1+) : <no title>
Query89 (2285.9290,1+) : <no title>
Query90 (2383.9100,1+) : <no title>
Query91 (2398.9692,1+) : <no title>
Query92 (2705.1276,1+) : <no title>
Query93 (3312.3547,1+) : <no title>
```

Mascot: http://www.matrixscience.com/
