# Supplementary material for: Proteomic analysis of Salmonella enterica serovar Enteritidis following propionate adaptation
Source: BMC Microbiol. 2010 Sep 28;10:249. doi: 10.1186/1471-2180-10-249 (PMC2957393; doi:10.1186/1471-2180-10-249)
Supplement: Additional file 2 — Protein Report B. Mass spectrometry report for RplF [file 1471-2180-10-249-S2.PDF]

Mascot Search Results

```
User            :
Email           : rliyana@uark.edu
Search title    :
MS data file    : DATA.TXT
Database        : NCBInr 20071202 (5678482 sequences; 1961803296 residues)
Taxonomy        : Bacteria (Eubacteria) (2746213 sequences)
Timestamp       : 23 Dec 2008 at 04:23:49 GMT
Warning         : A Peptide summary report will usually give a much clearer picture of MS/MS se
Top Score       : 177 for gi|16762858, 50S ribosomal protein L6 [Salmonella enterica subsp. ent
```

Probability Based Mowse Score

Protein score is -10*Log(P), where P is the probability that the observed match is a random event.
Protein scores greater than 77 are significant (p<0.05).
Protein scores are derived from ions scores as a non-probabilistic basis for ranking protein hits.

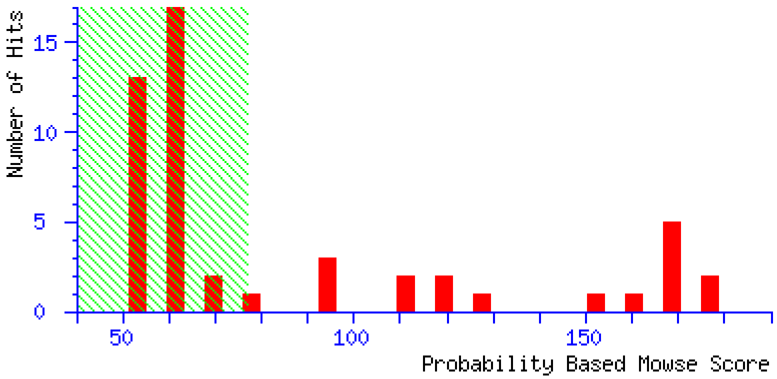

Protein Summary Report

Format As | Protein Summary (deprecated) | Help

Significance threshold p< 0.05 Max. number of hits 50

Standard scoring ◉ MudPIT scoring ○ Ions score or expect cut-off 0 Show sub-sets 0

Show pop-ups ◉ Suppress pop-ups ○ Sort unassigned Decreasing Score Require bold red ☐

Overview Table

Click on column header to jump to entry in results list.
Move mouse over any indicator to highlight identical peptides.
Click on an indicator to see details of individual match.
Use check boxes to select sub-set of queries for new search.

Mouse over: -Query-

-Accession- -Sequence-

Hit:	1	2	3	4	5	6	7	8	9	10	11	12	13	14	15	16	17	18	19	20	21	22	23	24	25	26	27	28	29	30	31	32	33	34	35
☑ 832.4830 (1+)																																			
☑ 864.4371 (1+)																																			
☑ 870.5446 (1+)																																			
☑ 897.4156 (1+)																																			
☑ 919.5370 (1+)																																			
☑ 973.5306 (1+)																																			
☑ 974.4789 (1+)																																			
☑ 986.5797 (1+)																																			
☑ 1004.5919 (1+)	●	●	●	●	●	●	●	●	●	●	●	●	●	●	●	●	●			●	●	●	●	●					●	●					
☑ 1037.5306 (1+)																																			
☑ 1060.5627 (1+)																																			
☑ 1132.6766 (1+)																																			
☑ 1143.5376 (1+)																																			

☑ 1150.6438 (1+)																																			
☑ 1152.6352 (1+)																																			
☑ 1160.5670 (1+)																																			
☑ 1169.6591 (1+)	●	●	●	●	●	●	●	●	●	●	●	●	●	●			●	●		●	●						●	●			●	●	●	●	●
☑ 1193.6003 (1+)																																			
☑ 1198.5902 (1+)																																			
☑ 1230.5778 (1+)																																			
☑ 1234.6676 (1+)																																			
☑ 1261.6761 (1+)																																			
☑ 1263.6787 (1+)																																			
☑ 1277.6967 (1+)																																			
☑ 1302.6902 (1+)																																			
☑ 1307.6557 (1+)																																			
☑ 1340.6607 (1+)																																			
☑ 1357.6919 (1+)																																			
☑ 1383.6631 (1+)																																			
☑ 1390.6668 (1+)																																			
☑ 1407.6965 (1+)																																			
☑ 1434.7636 (1+)																																			
☑ 1438.6220 (1+)																																			
☑ 1458.6980 (1+)																																			
☑ 1475.7261 (1+)																																			
☑ 1487.7204 (1+)																																			
☑ 1584.8657 (1+)																																			
☑ 1638.8097 (1+)																																			
☑ 1657.7630 (1+)																																			
☑ 1687.9216 (1+)																																			

☑ 1695.8492 (1+)	●	●	●	●	●	●	●	●	●						●	●																			
☑ 1699.7953 (1+)																																			
☑ 1707.7412 (1+)																																			
☑ 1838.8869 (1+)																																			
☑ 1851.8805 (1+)																																			
☑ 1888.8985 (1+)																																			
☑ 1904.8884 (1+)																																			
☑ 1940.8860 (1+)																																			
☑ 2225.0763 (1+)																																			
☑ 2239.0817 (1+)																																			
☑ 2248.1493 (1+)																																			
☑ 2252.0589 (1+)																																			
☑ 2286.0247 (1+)																																			
☑ 2399.0067 (1+)																																			
☑ 2510.1289 (1+)																																			
☑ 2705.2084 (1+)																																			

Select All Select None Search Selected

Index

	Accession	Mass	Score	Description
1.	gi|16762858	18905	177	50S ribosomal protein L6 [Salmonella enterica subsp. enterica s
2.	gi|152972212	18889	177	50S ribosomal protein L6 [Klebsiella pneumoniae subsp. pneumoni
3.	gi|116667439	17821	166	Chain E, Structure Of The 50s Subunit Of A Pre-Translocational
4.	gi|33357906	18818	165	Chain E, Real Space Refined Coordinates Of The 50s Subunit Fitt
5.	gi|15803832	18949	165	50S ribosomal protein L6 [Escherichia coli O157:H7 EDL933]
6.	gi|74313824	18977	165	50S ribosomal subunit protein L6 [Shigella sonnei Ss046]
7.	gi|160867370	18933	165	hypothetical protein SARI_04204 [Salmonella enterica subsp. ari
8.	gi|157148881	18933	164	hypothetical protein CKO_04719 [Citrobacter koseri ATCC BAA-895

9.	gi|16120562	18972	156	50S ribosomal protein L6 [Yersinia pestis CO92]
10.	gi|42984	19007	129	unnamed protein product [Escherichia coli]
11.	gi|77974974	18942	121	COG0097: Ribosomal protein L6P/L9E [Yersinia frederiksenii ATCC
12.	gi|123444083	18956	121	50S ribosomal protein L6 [Yersinia enterocolitica subsp. entero
13.	gi|77957316	18928	109	COG0097: Ribosomal protein L6P/L9E [Yersinia bercovieri ATCC 43
14.	gi|157372761	18927	109	ribosomal protein L6 [Serratia proteamaculans 568]
15.	gi|75214891	18958	97	COG0097: Ribosomal protein L6P/L9E [Escherichia coli E110019]
16.	gi|156932250	18933	97	hypothetical protein ESA_00021 [Enterobacter sakazakii ATCC BAA
17.	gi|121730159	18996	96	ribosomal protein L6 [Vibrio cholerae V52]
18.	gi|229631	18877	77	ribosomal protein L6
19.	gi|152979147	28967	73	Dihydrodipicolinate reductase [Actinobacillus succinogenes 130Z
20.	gi|78484655	18857	71	Ribosomal protein L6 [Thiomicrospira crunogena XCL-2]
21.	gi|114561351	18874	63	ribosomal protein L6 [Shewanella frigidimarina NCIMB 400]
22.	gi|32034702	18918	63	COG0097: Ribosomal protein L6P/L9E [Actinobacillus pleuropneumo
23.	gi|113460224	19091	62	50S ribosomal protein L6 [Haemophilus somnus 129PT]
24.	gi|32030990	19042	62	COG0097: Ribosomal protein L6P/L9E [Haemophilus somnus 2336]
25.	gi|54293560	55446	62	hypothetical protein lpl0612 [Legionella pneumophila str. Lens]
26.	gi|148360776	55461	62	UDP-N-acetylglucosamine 1- carboxyvinyltransferase [Legionella
27.	gi|54307556	18923	62	50S ribosomal protein L6 [Photobacterium profundum SS9]
28.	gi|90414971	18856	62	50S ribosomal protein L6 [Photobacterium profundum 3TCK]
29.	gi|52426088	19026	62	50S ribosomal protein L6 [Mannheimia succiniciproducens MBEL55E
30.	gi|146313370	18843	61	ribosomal protein L6 [Enterobacter sp. 638]
31.	gi|28897046	18881	59	50S ribosomal protein L6 [Vibrio parahaemolyticus RIMD 2210633]
32.	gi|91228963	18792	59	50S ribosomal protein L6 [Vibrio alginolyticus 12G01]
33.	gi|156973066	18867	59	ribosomal protein L6P/L9E [Vibrio harveyi ATCC BAA-1116]
34.	gi|154500500	6910	59	hypothetical protein BACCAP_04173 [Bacteroides capillosus ATCC
35.	gi|148982236	18859	59	50S ribosomal protein L6 [Vibrionales bacterium SWAT-3]
36.	gi|146312705	14384	59	formate C-acetyltransferase glycine radical [Enterobacter sp. 6
37.	gi|149192384	22354	58	50S ribosomal protein L6 [Vibrio shilonii AK1]
38.	gi|153806077	67846	57	hypothetical protein BACCAC_00328 [Bacteroides caccae ATCC 4318
39.	gi|94317657	6979	56	hypothetical protein [Salmonella enterica subsp. enterica serov
40.	gi|89074671	87370	55	hypothetical membrane carboxypeptidase [Photobacterium sp. SKA3
41.	gi|85060241	18971	54	50S ribosomal protein L6 [Sodalis glossinidius str. 'morsitans'
42.	gi|81176852	81296	54	Polyribonucleotide nucleotidyltransferase [Streptococcus suis 8
43.	gi|94312233	18896	54	ribosomal protein L6 [Ralstonia metallidurans CH34]
44.	gi|116625125	27271	54	hypothetical protein Acid_6062 [Solibacter usitatus Ellin6076]
45.	gi|89076309	18871	53	50S ribosomal protein L6 [Photobacterium sp. SKA34]

46.	gi|59710859	19010	53	50S ribosomal protein L6 [Vibrio fischeri ES114]
47.	gi|90581693	18952	53	50S ribosomal protein L6 [Vibrio angustum S14]
48.	gi|117621078	18568	53	50S ribosomal protein L6 [Aeromonas hydrophila subsp. hydrophil
49.	gi|145300920	18556	53	ribosomal protein L6 [Aeromonas salmonicida subsp. salmonicida
50.	gi|51894195	19030	53	50S ribosomal protein L6 [Symbiobacterium thermophilum IAM 1486

Results List

1. gi|16762858 **Mass:** 18905 **Score:** 177 **Expect:** 5.5e-012 **Queries matched:** 7

50S ribosomal protein L6 [Salmonella enterica subsp. enterica serovar Typhi str. CT18]

Observed	Mr(expt)	Mr(calc)	ppm	Start		End	Miss	Ions	Peptide
1004.5919	1003.5846	1003.5815	3.13	87	-	95	0	33	K.LQLVGVGYR.A
1132.6766	1131.6693	1131.6764	-6.26	86	-	95	1	---	K.KLQLVGVGYR.A
1150.6438	1149.6365	1149.6758	-34.14	7	-	18	0	---	K.APVVVPAGVDVK.I
1169.6591	1168.6518	1168.6564	-3.95	139	-	149	0	57	K.QVIGQVAADLR.A
1198.5902	1197.5829	1197.5891	-5.15	45	-	55	0	---	K.HADNALTFGPR.D
1438.6220	1437.6147	1437.6273	-8.77	56	-	69	0	---	R.DGYADGWAQAGTAR.A
1695.8492	1694.8419	1694.8913	-29.15	70	-	85	0	23	R.ALLNSMVIGVTEGFTK.K + Oxi

No match to: 832.4830, 864.4371, 870.5446, 897.4156, 919.5370, 973.5306, 974.4789, 986.5797, 1037.5306, 1060.5627, 1143.5376, 1152.6352, 1160.5670, 1193.6003, 1230.5778, 1234.6676, 1261.6761, 1263.6787, 1277.6967, 1302.6902, 1307.6557, 1340.6607, 1357.6919, 1383.6631, 1390.6668, 1407.6965, 1434.7636, 1458.6980, 1475.7261, 1487.7204, 1584.8657, 1638.8097, 1657.7630, 1687.9216, 1699.7953, 1707.7412, 1838.8869, 1851.8805, 1888.8985, 1904.8884, 1940.8860, 2225.0763, 2239.0817, 2248.1493, 2252.0589, 2286.0247, 2399.0067, 2510.1289, 2705.2084

2. gi|152972212 **Mass:** 18889 **Score:** 177 **Expect:** 5.5e-012 **Queries matched:** 7

50S ribosomal protein L6 [Klebsiella pneumoniae subsp. pneumoniae MGH 78578]

Observed	Mr(expt)	Mr(calc)	ppm	Start		End	Miss	Ions	Peptide
1004.5919	1003.5846	1003.5815	3.13	87	-	95	0	33	K.LQLVGVGYR.A
1132.6766	1131.6693	1131.6764	-6.26	86	-	95	1	---	K.KLQLVGVGYR.A
1150.6438	1149.6365	1149.6758	-34.14	7	-	18	0	---	K.APVVVPAGVDVK.I
1169.6591	1168.6518	1168.6564	-3.95	139	-	149	0	57	K.QVIGQVAADLR.A
1198.5902	1197.5829	1197.5891	-5.15	45	-	55	0	---	K.HADNALTFGPR.D

1438.6220	1437.6147	1437.6273	-8.77	56 -	69	0	---	R.DGYADGWAQAGTAR.A
1695.8492	1694.8419	1694.8913	-29.15	70 -	85	0	23	R.ALLNSMVIGVTEGFTK.K + Oxi

No match to: 832.4830, 864.4371, 870.5446, 897.4156, 919.5370, 973.5306, 974.4789, 986.5797, 1037.5306, 1060.5627, 1143.5376, 1152.6352, 1160.5670, 1193.6003, 1230.5778, 1234.6676, 1261.6761, 1263.6787, 1277.6967, 1302.6902, 1307.6557, 1340.6607, 1357.6919, 1383.6631, 1390.6668, 1407.6965, 1434.7636, 1458.6980, 1475.7261, 1487.7204, 1584.8657, 1638.8097, 1657.7630, 1687.9216, 1699.7953, 1707.7412, 1838.8869, 1851.8805, 1888.8985, 1904.8884, 1940.8860, 2225.0763, 2239.0817, 2248.1493, 2252.0589, 2286.0247, 2399.0067, 2510.1289, 2705.2084

3. gi|116667439 **Mass:** 17821 **Score:** 166 **Expect:** 6.9e-011 **Queries matched:** 6

Chain E, Structure Of The 50s Subunit Of A Pre-Translocational E. Coli Ribosome Obtained

Observed	Mr(expt)	Mr(calc)	ppm	Start	End	Miss	Ions	Peptide
1004.5919	1003.5846	1003.5815	3.13	82 -	90	0	33	K.LQLVGVGYR.A
1132.6766	1131.6693	1131.6764	-6.26	81 -	90	1	---	K.KLQLVGVGYR.A
1150.6438	1149.6365	1149.6758	-34.14	2 -	13	0	---	K.APVVVPAGVDVK.I
1169.6591	1168.6518	1168.6564	-3.95	134 -	144	0	57	K.QVIGQVAADLR.A
1438.6220	1437.6147	1437.6273	-8.77	51 -	64	0	---	R.DGYADGWAQAGTAR.A
1695.8492	1694.8419	1694.8913	-29.15	65 -	80	0	23	R.ALLNSMVIGVTEGFTK.K + Oxi

No match to: 832.4830, 864.4371, 870.5446, 897.4156, 919.5370, 973.5306, 974.4789, 986.5797, 1037.5306, 1060.5627, 1143.5376, 1152.6352, 1160.5670, 1193.6003, 1198.5902, 1230.5778, 1234.6676, 1261.6761, 1263.6787, 1277.6967, 1302.6902, 1307.6557, 1340.6607, 1357.6919, 1383.6631, 1390.6668, 1407.6965, 1434.7636, 1458.6980, 1475.7261, 1487.7204, 1584.8657, 1638.8097, 1657.7630, 1687.9216, 1699.7953, 1707.7412, 1838.8869, 1851.8805, 1888.8985, 1904.8884, 1940.8860, 2225.0763, 2239.0817, 2248.1493, 2252.0589, 2286.0247, 2399.0067, 2510.1289, 2705.2084

4. gi|33357906 **Mass:** 18818 **Score:** 165 **Expect:** 8.7e-011 **Queries matched:** 6

Chain E, Real Space Refined Coordinates Of The 50s Subunit Fitted Into The Low Resolution

Observed	Mr(expt)	Mr(calc)	ppm	Start	End	Miss	Ions	Peptide
1004.5919	1003.5846	1003.5815	3.13	86 -	94	0	33	K.LQLVGVGYR.A
1132.6766	1131.6693	1131.6764	-6.26	85 -	94	1	---	K.KLQLVGVGYR.A
1150.6438	1149.6365	1149.6758	-34.14	6 -	17	0	---	K.APVVVPAGVDVK.I
1169.6591	1168.6518	1168.6564	-3.95	138 -	148	0	57	K.QVIGQVAADLR.A

1438.6220	1437.6147	1437.6273	-8.77	55	-	68	0	---	R.DGYADGWAQAGTAR.A
1695.8492	1694.8419	1694.8913	-29.15	69	-	84	0	23	R.ALLNSMVIGVTEGFTK.K + Oxi

No match to: 832.4830, 864.4371, 870.5446, 897.4156, 919.5370, 973.5306, 974.4789, 986.5797, 1037.5306, 1060.5627, 1143.5376, 1152.6352, 1160.5670, 1193.6003, 1198.5902, 1230.5778, 1234.6676, 1261.6761, 1263.6787, 1277.6967, 1302.6902, 1307.6557, 1340.6607, 1357.6919, 1383.6631, 1390.6668, 1407.6965, 1434.7636, 1458.6980, 1475.7261, 1487.7204, 1584.8657, 1638.8097, 1657.7630, 1687.9216, 1699.7953, 1707.7412, 1838.8869, 1851.8805, 1888.8985, 1904.8884, 1940.8860, 2225.0763, 2239.0817, 2248.1493, 2252.0589, 2286.0247, 2399.0067, 2510.1289, 2705.2084

5. gi|15803832 **Mass:** 18949 **Score:** 165 **Expect:** 8.7e-011 **Queries matched:** 6
50S ribosomal protein L6 [Escherichia coli O157:H7 EDL933]

Observed	Mr(expt)	Mr(calc)	ppm	Start		End	Miss	Ions	Peptide
1004.5919	1003.5846	1003.5815	3.13	87	-	95	0	33	K.LQLVGVGYR.A
1132.6766	1131.6693	1131.6764	-6.26	86	-	95	1	---	K.KLQLVGVGYR.A
1150.6438	1149.6365	1149.6758	-34.14	7	-	18	0	---	K.APVVVPAGVDVK.I
1169.6591	1168.6518	1168.6564	-3.95	139	-	149	0	57	K.QVIGQVAADLR.A
1438.6220	1437.6147	1437.6273	-8.77	56	-	69	0	---	R.DGYADGWAQAGTAR.A
1695.8492	1694.8419	1694.8913	-29.15	70	-	85	0	23	R.ALLNSMVIGVTEGFTK.K + Oxi

No match to: 832.4830, 864.4371, 870.5446, 897.4156, 919.5370, 973.5306, 974.4789, 986.5797, 1037.5306, 1060.5627, 1143.5376, 1152.6352, 1160.5670, 1193.6003, 1198.5902, 1230.5778, 1234.6676, 1261.6761, 1263.6787, 1277.6967, 1302.6902, 1307.6557, 1340.6607, 1357.6919, 1383.6631, 1390.6668, 1407.6965, 1434.7636, 1458.6980, 1475.7261, 1487.7204, 1584.8657, 1638.8097, 1657.7630, 1687.9216, 1699.7953, 1707.7412, 1838.8869, 1851.8805, 1888.8985, 1904.8884, 1940.8860, 2225.0763, 2239.0817, 2248.1493, 2252.0589, 2286.0247, 2399.0067, 2510.1289, 2705.2084

6. gi|74313824 **Mass:** 18977 **Score:** 165 **Expect:** 8.7e-011 **Queries matched:** 6
50S ribosomal subunit protein L6 [Shigella sonnei Ss046]

Observed	Mr(expt)	Mr(calc)	ppm	Start		End	Miss	Ions	Peptide
1004.5919	1003.5846	1003.5815	3.13	87	-	95	0	33	K.LQLVGVGYR.A
1132.6766	1131.6693	1131.6764	-6.26	86	-	95	1	---	K.KLQLVGVGYR.A
1150.6438	1149.6365	1149.6758	-34.14	7	-	18	0	---	K.APVVVPAGVDVK.I
1169.6591	1168.6518	1168.6564	-3.95	139	-	149	0	57	K.QVIGQVAADLR.A

1438.6220	1437.6147	1437.6273	-8.77	56 -	69	0	---	R.DGYADGWAQAGTAR.A
1695.8492	1694.8419	1694.8913	-29.15	70 -	85	0	23	R.ALLNSMVIGVTEGFTK.K + Oxi

No match to: 832.4830, 864.4371, 870.5446, 897.4156, 919.5370, 973.5306, 974.4789, 986.5797, 1037.5306, 1060.5627, 1143.5376, 1152.6352, 1160.5670, 1193.6003, 1198.5902, 1230.5778, 1234.6676, 1261.6761, 1263.6787, 1277.6967, 1302.6902, 1307.6557, 1340.6607, 1357.6919, 1383.6631, 1390.6668, 1407.6965, 1434.7636, 1458.6980, 1475.7261, 1487.7204, 1584.8657, 1638.8097, 1657.7630, 1687.9216, 1699.7953, 1707.7412, 1838.8869, 1851.8805, 1888.8985, 1904.8884, 1940.8860, 2225.0763, 2239.0817, 2248.1493, 2252.0589, 2286.0247, 2399.0067, 2510.1289, 2705.2084

7. gi|160867370 **Mass:** 18933 **Score:** 165 **Expect:** 8.7e-011 **Queries matched:** 6

hypothetical protein SARI_04204 [Salmonella enterica subsp. arizonae serovar 62:z4,z23:--

Observed	Mr(expt)	Mr(calc)	ppm	Start	End	Miss	Ions	Peptide
1004.5919	1003.5846	1003.5815	3.13	87 -	95	0	33	K.LQLVGVGYR.A
1132.6766	1131.6693	1131.6764	-6.26	86 -	95	1	---	K.KLQLVGVGYR.A
1150.6438	1149.6365	1149.6758	-34.14	7 -	18	0	---	K.APVVVPAGVDVK.I
1169.6591	1168.6518	1168.6564	-3.95	139 -	149	0	57	K.QVIGQVAADLR.A
1438.6220	1437.6147	1437.6273	-8.77	56 -	69	0	---	R.DGYADGWAQAGTAR.A
1695.8492	1694.8419	1694.8913	-29.15	70 -	85	0	23	R.ALLNSMVIGVTEGFTK.K + Oxi

No match to: 832.4830, 864.4371, 870.5446, 897.4156, 919.5370, 973.5306, 974.4789, 986.5797, 1037.5306, 1060.5627, 1143.5376, 1152.6352, 1160.5670, 1193.6003, 1198.5902, 1230.5778, 1234.6676, 1261.6761, 1263.6787, 1277.6967, 1302.6902, 1307.6557, 1340.6607, 1357.6919, 1383.6631, 1390.6668, 1407.6965, 1434.7636, 1458.6980, 1475.7261, 1487.7204, 1584.8657, 1638.8097, 1657.7630, 1687.9216, 1699.7953, 1707.7412, 1838.8869, 1851.8805, 1888.8985, 1904.8884, 1940.8860, 2225.0763, 2239.0817, 2248.1493, 2252.0589, 2286.0247, 2399.0067, 2510.1289, 2705.2084

8. gi|157148881 **Mass:** 18933 **Score:** 164 **Expect:** 1.1e-010 **Queries matched:** 6

hypothetical protein CKO_04719 [Citrobacter koseri ATCC BAA-895]

Observed	Mr(expt)	Mr(calc)	ppm	Start	End	Miss	Ions	Peptide
1004.5919	1003.5846	1003.5815	3.13	87 -	95	0	33	K.LQLVGVGYR.A
1132.6766	1131.6693	1131.6764	-6.26	86 -	95	1	---	K.KLQLVGVGYR.A
1150.6438	1149.6365	1149.6758	-34.14	7 -	18	0	---	K.APVVVPAGVDVK.I
1169.6591	1168.6518	1168.6564	-3.95	139 -	149	0	57	K.QVIGQVAADLR.A

1198.5902	1197.5829	1197.5891	-5.15	45	-	55	0	---	K.HADNALTFGPR.D
1695.8492	1694.8419	1694.8913	-29.15	70	-	85	0	23	R.ALLNSMVIGVTEGFTK.K + Oxi

No match to: 832.4830, 864.4371, 870.5446, 897.4156, 919.5370, 973.5306, 974.4789, 986.5797, 1037.5306, 1060.5627, 1143.5376, 1152.6352, 1160.5670, 1193.6003, 1230.5778, 1234.6676, 1261.6761, 1263.6787, 1277.6967, 1302.6902, 1307.6557, 1340.6607, 1357.6919, 1383.6631, 1390.6668, 1407.6965, 1434.7636, 1438.6220, 1458.6980, 1475.7261, 1487.7204, 1584.8657, 1638.8097, 1657.7630, 1687.9216, 1699.7953, 1707.7412, 1838.8869, 1851.8805, 1888.8985, 1904.8884, 1940.8860, 2225.0763, 2239.0817, 2248.1493, 2252.0589, 2286.0247, 2399.0067, 2510.1289, 2705.2084

9. gi|16120562 **Mass:** 18972 **Score:** 156 **Expect:** 6.9e-010 **Queries matched:** 5

50S ribosomal protein L6 [Yersinia pestis CO92]

Observed	Mr(expt)	Mr(calc)	ppm	Start		End	Miss	Ions	Peptide
1004.5919	1003.5846	1003.5815	3.13	87	-	95	0	33	K.LQLVGVGYR.A
1132.6766	1131.6693	1131.6764	-6.26	86	-	95	1	---	K.KLQLVGVGYR.A
1169.6591	1168.6518	1168.6564	-3.95	139	-	149	0	57	K.QVIGQVAADLR.A
1695.8492	1694.8419	1694.8913	-29.15	70	-	85	0	23	R.ALLNSMVIGVTEGFTK.K + Oxi
2705.2084	2704.2011	2704.2841	-30.69	45	-	69	1	---	K.QEENTLTFAPREGAVDGWAQAGTT

No match to: 832.4830, 864.4371, 870.5446, 897.4156, 919.5370, 973.5306, 974.4789, 986.5797, 1037.5306, 1060.5627, 1143.5376, 1150.6438, 1152.6352, 1160.5670, 1193.6003, 1198.5902, 1230.5778, 1234.6676, 1261.6761, 1263.6787, 1277.6967, 1302.6902, 1307.6557, 1340.6607, 1357.6919, 1383.6631, 1390.6668, 1407.6965, 1434.7636, 1438.6220, 1458.6980, 1475.7261, 1487.7204, 1584.8657, 1638.8097, 1657.7630, 1687.9216, 1699.7953, 1707.7412, 1838.8869, 1851.8805, 1888.8985, 1904.8884, 1940.8860, 2225.0763, 2239.0817, 2248.1493, 2252.0589, 2286.0247, 2399.0067, 2510.1289

10. gi|42984 **Mass:** 19007 **Score:** 129 **Expect:** 3.5e-007 **Queries matched:** 5

unnamed protein product [Escherichia coli]

Observed	Mr(expt)	Mr(calc)	ppm	Start		End	Miss	Ions	Peptide
1004.5919	1003.5846	1003.5815	3.13	87	-	95	0	33	K.LQLVGVGYR.A
1132.6766	1131.6693	1131.6764	-6.26	86	-	95	1	---	K.KLQLVGVGYR.A
1150.6438	1149.6365	1149.6758	-34.14	7	-	18	0	---	K.APVVVPAGVDVK.I
1169.6591	1168.6518	1168.6564	-3.95	139	-	149	0	57	K.QVIGQVAADLR.A
1438.6220	1437.6147	1437.6273	-8.77	56	-	69	0	---	R.DGYADGWAQAGTAR.A

No match to: 832.4830, 864.4371, 870.5446, 897.4156, 919.5370, 973.5306, 974.4789, 986.5797, 1037.5306, 1060.5627, 1143.5376, 1152.6352, 1160.5670, 1193.6003, 1198.5902, 1230.5778, 1234.6676, 1261.6761, 1263.6787, 1277.6967, 1302.6902, 1307.6557, 1340.6607, 1357.6919, 1383.6631, 1390.6668, 1407.6965, 1434.7636, 1458.6980, 1475.7261, 1487.7204, 1584.8657, 1638.8097, 1657.7630, 1687.9216, 1695.8492, 1699.7953, 1707.7412, 1838.8869, 1851.8805, 1888.8985, 1904.8884, 1940.8860, 2225.0763, 2239.0817, 2248.1493, 2252.0589, 2286.0247, 2399.0067, 2510.1289, 2705.2084

11. gi|77974974 **Mass:** 18942 **Score:** 121 **Expect:** 2.2e-006 **Queries matched:** 4
COG0097: Ribosomal protein L6P/L9E [Yersinia frederiksenii ATCC 33641]

Observed	Mr(expt)	Mr(calc)	ppm	Start	End	Miss	Ions	Peptide
1004.5919	1003.5846	1003.5815	3.13	87 -	95	0	33	K.LQLVGVGYR.A
1132.6766	1131.6693	1131.6764	-6.26	86 -	95	1	---	K.KLQLVGVGYR.A
1169.6591	1168.6518	1168.6564	-3.95	139 -	149	0	57	K.QVIGQVAADLR.A
2705.2084	2704.2011	2704.2841	-30.69	45 -	69	1	---	K.QEENTLTFAPREGAVDGWAQAGTT

No match to: 832.4830, 864.4371, 870.5446, 897.4156, 919.5370, 973.5306, 974.4789, 986.5797, 1037.5306, 1060.5627, 1143.5376, 1150.6438, 1152.6352, 1160.5670, 1193.6003, 1198.5902, 1230.5778, 1234.6676, 1261.6761, 1263.6787, 1277.6967, 1302.6902, 1307.6557, 1340.6607, 1357.6919, 1383.6631, 1390.6668, 1407.6965, 1434.7636, 1438.6220, 1458.6980, 1475.7261, 1487.7204, 1584.8657, 1638.8097, 1657.7630, 1687.9216, 1695.8492, 1699.7953, 1707.7412, 1838.8869, 1851.8805, 1888.8985, 1904.8884, 1940.8860, 2225.0763, 2239.0817, 2248.1493, 2252.0589, 2286.0247, 2399.0067, 2510.1289

12. gi|123444083 **Mass:** 18956 **Score:** 121 **Expect:** 2.2e-006 **Queries matched:** 4
50S ribosomal protein L6 [Yersinia enterocolitica subsp. enterocolitica 8081]

Observed	Mr(expt)	Mr(calc)	ppm	Start	End	Miss	Ions	Peptide
1004.5919	1003.5846	1003.5815	3.13	87 -	95	0	33	K.LQLVGVGYR.A
1132.6766	1131.6693	1131.6764	-6.26	86 -	95	1	---	K.KLQLVGVGYR.A
1169.6591	1168.6518	1168.6564	-3.95	139 -	149	0	57	K.QVIGQVAADLR.A
2705.2084	2704.2011	2704.2841	-30.69	45 -	69	1	---	K.QEENTLTFAPREGAVDGWAQAGTT

No match to: 832.4830, 864.4371, 870.5446, 897.4156, 919.5370, 973.5306, 974.4789, 986.5797, 1037.5306, 1060.5627, 1143.5376, 1150.6438, 1152.6352, 1160.5670, 1193.6003, 1198.5902, 1230.5778, 1234.6676, 1261.6761, 1263.6787, 1277.6967, 1302.6902, 1307.6557, 1340.6607, 1357.6919, 1383.6631, 1390.6668, 1407.6965, 1434.7636, 1438.6220, 1458.6980,

1475.7261, 1487.7204, 1584.8657, 1638.8097, 1657.7630, 1687.9216, 1695.8492, 1699.7953, 1707.7412, 1838.8869, 1851.8805, 1888.8985, 1904.8884, 1940.8860, 2225.0763, 2239.0817, 2248.1493, 2252.0589, 2286.0247, 2399.0067, 2510.1289

13. gi|77957316 **Mass:** 18928 **Score:** 109 **Expect:** 3.5e-005 **Queries matched:** 3
COG0097: Ribosomal protein L6P/L9E [Yersinia bercovieri ATCC 43970]

Observed	Mr(expt)	Mr(calc)	ppm	Start	End	Miss	Ions	Peptide
1004.5919	1003.5846	1003.5815	3.13	87 -	95	0	33	K.LQLVGVGYR.A
1132.6766	1131.6693	1131.6764	-6.26	86 -	95	1	---	K.KLQLVGVGYR.A
1169.6591	1168.6518	1168.6564	-3.95	139 -	149	0	57	K.QVIGQVAADLR.A

No match to: 832.4830, 864.4371, 870.5446, 897.4156, 919.5370, 973.5306, 974.4789, 986.5797, 1037.5306, 1060.5627, 1143.5376, 1150.6438, 1152.6352, 1160.5670, 1193.6003, 1198.5902, 1230.5778, 1234.6676, 1261.6761, 1263.6787, 1277.6967, 1302.6902, 1307.6557, 1340.6607, 1357.6919, 1383.6631, 1390.6668, 1407.6965, 1434.7636, 1438.6220, 1458.6980, 1475.7261, 1487.7204, 1584.8657, 1638.8097, 1657.7630, 1687.9216, 1695.8492, 1699.7953, 1707.7412, 1838.8869, 1851.8805, 1888.8985, 1904.8884, 1940.8860, 2225.0763, 2239.0817, 2248.1493, 2252.0589, 2286.0247, 2399.0067, 2510.1289, 2705.2084

14. gi|157372761 **Mass:** 18927 **Score:** 109 **Expect:** 3.5e-005 **Queries matched:** 3
ribosomal protein L6 [Serratia proteamaculans 568]

Observed	Mr(expt)	Mr(calc)	ppm	Start	End	Miss	Ions	Peptide
1004.5919	1003.5846	1003.5815	3.13	87 -	95	0	33	K.LQLVGVGYR.A
1132.6766	1131.6693	1131.6764	-6.26	86 -	95	1	---	K.KLQLVGVGYR.A
1169.6591	1168.6518	1168.6564	-3.95	139 -	149	0	57	K.QVIGQVAADLR.A

No match to: 832.4830, 864.4371, 870.5446, 897.4156, 919.5370, 973.5306, 974.4789, 986.5797, 1037.5306, 1060.5627, 1143.5376, 1150.6438, 1152.6352, 1160.5670, 1193.6003, 1198.5902, 1230.5778, 1234.6676, 1261.6761, 1263.6787, 1277.6967, 1302.6902, 1307.6557, 1340.6607, 1357.6919, 1383.6631, 1390.6668, 1407.6965, 1434.7636, 1438.6220, 1458.6980, 1475.7261, 1487.7204, 1584.8657, 1638.8097, 1657.7630, 1687.9216, 1695.8492, 1699.7953, 1707.7412, 1838.8869, 1851.8805, 1888.8985, 1904.8884, 1940.8860, 2225.0763, 2239.0817, 2248.1493, 2252.0589, 2286.0247, 2399.0067, 2510.1289, 2705.2084

15. gi|75214891 **Mass:** 18958 **Score:** 97 **Expect:** 0.00055 **Queries matched:** 5
COG0097: Ribosomal protein L6P/L9E [Escherichia coli E110019]

Observed	Mr(expt)	Mr(calc)	ppm	Start	End	Miss	Ions	Peptide
1004.5919	1003.5846	1003.5815	3.13	87 -	95	0	33	K.LQLVGVGYR.A
1132.6766	1131.6693	1131.6764	-6.26	86 -	95	1	---	K.KLQLVGVGYR.A
1150.6438	1149.6365	1149.6758	-34.14	7 -	18	0	---	K.APVVVPAGVDVK.I
1438.6220	1437.6147	1437.6273	-8.77	56 -	69	0	---	R.DGYADGWAQAGTAR.A
1695.8492	1694.8419	1694.8913	-29.15	70 -	85	0	23	R.ALLNSMVIGVTEGFTK.K + Oxi

No match to: 832.4830, 864.4371, 870.5446, 897.4156, 919.5370, 973.5306, 974.4789, 986.5797, 1037.5306, 1060.5627, 1143.5376, 1152.6352, 1160.5670, 1169.6591, 1193.6003, 1198.5902, 1230.5778, 1234.6676, 1261.6761, 1263.6787, 1277.6967, 1302.6902, 1307.6557, 1340.6607, 1357.6919, 1383.6631, 1390.6668, 1407.6965, 1434.7636, 1458.6980, 1475.7261, 1487.7204, 1584.8657, 1638.8097, 1657.7630, 1687.9216, 1699.7953, 1707.7412, 1838.8869, 1851.8805, 1888.8985, 1904.8884, 1940.8860, 2225.0763, 2239.0817, 2248.1493, 2252.0589, 2286.0247, 2399.0067, 2510.1289, 2705.2084

16. gi|156932250 **Mass:** 18933 **Score:** 97 **Expect:** 0.00057 **Queries matched:** 5

hypothetical protein ESA_00021 [Enterobacter sakazakii ATCC BAA-894]

Observed	Mr(expt)	Mr(calc)	ppm	Start	End	Miss	Ions	Peptide
1004.5919	1003.5846	1003.5815	3.13	87 -	95	0	33	K.LQLVGVGYR.A
1132.6766	1131.6693	1131.6764	-6.26	86 -	95	1	---	K.KLQLVGVGYR.A
1198.5902	1197.5829	1197.5891	-5.15	45 -	55	0	---	K.HADNALTFGPR.D
1438.6220	1437.6147	1437.6273	-8.77	56 -	69	0	---	R.DGYADGWAQAGTAR.A
1695.8492	1694.8419	1694.8913	-29.15	70 -	85	0	23	R.ALLNSMVIGVTEGFTK.K + Oxi

No match to: 832.4830, 864.4371, 870.5446, 897.4156, 919.5370, 973.5306, 974.4789, 986.5797, 1037.5306, 1060.5627, 1143.5376, 1150.6438, 1152.6352, 1160.5670, 1169.6591, 1193.6003, 1230.5778, 1234.6676, 1261.6761, 1263.6787, 1277.6967, 1302.6902, 1307.6557, 1340.6607, 1357.6919, 1383.6631, 1390.6668, 1407.6965, 1434.7636, 1458.6980, 1475.7261, 1487.7204, 1584.8657, 1638.8097, 1657.7630, 1687.9216, 1699.7953, 1707.7412, 1838.8869, 1851.8805, 1888.8985, 1904.8884, 1940.8860, 2225.0763, 2239.0817, 2248.1493, 2252.0589, 2286.0247, 2399.0067, 2510.1289, 2705.2084

17. gi|121730159 **Mass:** 18996 **Score:** 96 **Expect:** 0.00072 **Queries matched:** 3

ribosomal protein L6 [Vibrio cholerae V52]

Observed	Mr(expt)	Mr(calc)	ppm	Start	End	Miss	Ions	Peptide
1004.5919	1003.5846	1003.6178	-33.12	87 -	95	1	31	K.LVLKGVGYR.A

1150.6438	1149.6365	1149.6758	-34.11	7 -	18	0	---	K.APVAIPAGVEVK.L
1169.6591	1168.6518	1168.6564	-3.95	139 -	149	0	48	K.QLVGQVAADIR.S

No match to: 832.4830, 864.4371, 870.5446, 897.4156, 919.5370, 973.5306, 974.4789, 986.5797, 1037.5306, 1060.5627, 1132.6766, 1143.5376, 1152.6352, 1160.5670, 1193.6003, 1198.5902, 1230.5778, 1234.6676, 1261.6761, 1263.6787, 1277.6967, 1302.6902, 1307.6557, 1340.6607, 1357.6919, 1383.6631, 1390.6668, 1407.6965, 1434.7636, 1438.6220, 1458.6980, 1475.7261, 1487.7204, 1584.8657, 1638.8097, 1657.7630, 1687.9216, 1695.8492, 1699.7953, 1707.7412, 1838.8869, 1851.8805, 1888.8985, 1904.8884, 1940.8860, 2225.0763, 2239.0817, 2248.1493, 2252.0589, 2286.0247, 2399.0067, 2510.1289, 2705.2084

18. gi|229631 **Mass:** 18877 **Score:** 77 **Expect:** 0.057 **Queries matched:** 3
ribosomal protein L6

Observed	Mr(expt)	Mr(calc)	ppm	Start	End	Miss	Ions	Peptide
1150.6438	1149.6365	1149.6758	-34.14	6 -	17	0	---	K.APVVVPAGVDVK.I
1169.6591	1168.6518	1168.6564	-3.95	138 -	148	0	57	K.QVIGQVAADLR.A
1438.6220	1437.6147	1437.6273	-8.77	55 -	68	0	---	R.DGYADGWAQAGTAR.A

No match to: 832.4830, 864.4371, 870.5446, 897.4156, 919.5370, 973.5306, 974.4789, 986.5797, 1004.5919, 1037.5306, 1060.5627, 1132.6766, 1143.5376, 1152.6352, 1160.5670, 1193.6003, 1198.5902, 1230.5778, 1234.6676, 1261.6761, 1263.6787, 1277.6967, 1302.6902, 1307.6557, 1340.6607, 1357.6919, 1383.6631, 1390.6668, 1407.6965, 1434.7636, 1458.6980, 1475.7261, 1487.7204, 1584.8657, 1638.8097, 1657.7630, 1687.9216, 1695.8492, 1699.7953, 1707.7412, 1838.8869, 1851.8805, 1888.8985, 1904.8884, 1940.8860, 2225.0763, 2239.0817, 2248.1493, 2252.0589, 2286.0247, 2399.0067, 2510.1289, 2705.2084

19. gi|152979147 **Mass:** 28967 **Score:** 73 **Expect:** 0.13 **Queries matched:** 9
Dihydrodipicolinate reductase [Actinobacillus succinogenes 130Z]

Observed	Mr(expt)	Mr(calc)	ppm	Start	End	Miss	Ions	Peptide
1037.5306	1036.5233	1036.5189	4.23	201 -	209	0	---	R.DEIGFSTIR.A
1193.6003	1192.5930	1192.6200	-22.67	200 -	209	1	---	K.RDEIGFSTIR.A
1302.6902	1301.6829	1301.7238	-31.40	1 -	13	1	---	-.MTLRIGVVGAGGR.M + Oxidat
1383.6631	1382.6559	1382.6725	-12.01	234 -	246	1	---	K.ASSRMTFANGAVR.A + Oxidat
1407.6965	1406.6893	1406.7042	-10.61	95 -	108	0	---	K.NVILGTTGFDDAGK.A
1699.7953	1698.7881	1698.8247	-21.55	161 -	177	0	---	K.VDAPSGTALSMGEHIAK.T + Ox
1707.7412	1706.7339	1706.8185	-49.61	256 -	270	0	---	K.QGLFDMTDVLDLNNL.-

1851.8805	1850.8732	1850.9084	-19.01	255 - 270	1	---	K.KQGLFDMTDVLDLNNL.- + Oxi
2239.0817	2238.0744	2238.0310	19.4	143 - 160	1	---	K.VMGDYCDIEIIEAHHRHK.V + O

No match to: 832.4830, 864.4371, 870.5446, 897.4156, 919.5370, 973.5306, 974.4789, 986.5797, 1004.5919, 1060.5627, 1132.6766, 1143.5376, 1150.6438, 1152.6352, 1160.5670, 1169.6591, 1198.5902, 1230.5778, 1234.6676, 1261.6761, 1263.6787, 1277.6967, 1307.6557, 1340.6607, 1357.6919, 1390.6668, 1434.7636, 1438.6220, 1458.6980, 1475.7261, 1487.7204, 1584.8657, 1638.8097, 1657.7630, 1687.9216, 1695.8492, 1838.8869, 1888.8985, 1904.8884, 1940.8860, 2225.0763, 2248.1493, 2252.0589, 2286.0247, 2399.0067, 2510.1289, 2705.2084

20. gi|78484655 **Mass:** 18857 **Score:** 71 **Expect:** 0.22 **Queries matched:** 3
Ribosomal protein L6 [Thiomicrospira crunogena XCL-2]

Observed	Mr(expt)	Mr(calc)	ppm	Start	End	Miss	Ions	Peptide
1004.5919	1003.5846	1003.5815	3.13	87 -	95	0	33	K.LQLVGVGYR.A
1132.6766	1131.6693	1131.6764	-6.26	86 -	95	1	---	K.KLQLVGVGYR.A
1169.6591	1168.6518	1168.6564	-3.95	139 -	149	0	19	K.QVVGQVAAEIR.G

No match to: 832.4830, 864.4371, 870.5446, 897.4156, 919.5370, 973.5306, 974.4789, 986.5797, 1037.5306, 1060.5627, 1143.5376, 1150.6438, 1152.6352, 1160.5670, 1193.6003, 1198.5902, 1230.5778, 1234.6676, 1261.6761, 1263.6787, 1277.6967, 1302.6902, 1307.6557, 1340.6607, 1357.6919, 1383.6631, 1390.6668, 1407.6965, 1434.7636, 1438.6220, 1458.6980, 1475.7261, 1487.7204, 1584.8657, 1638.8097, 1657.7630, 1687.9216, 1695.8492, 1699.7953, 1707.7412, 1838.8869, 1851.8805, 1888.8985, 1904.8884, 1940.8860, 2225.0763, 2239.0817, 2248.1493, 2252.0589, 2286.0247, 2399.0067, 2510.1289, 2705.2084

21. gi|114561351 **Mass:** 18874 **Score:** 63 **Expect:** 1.4 **Queries matched:** 2
ribosomal protein L6 [Shewanella frigidimarina NCIMB 400]

Observed	Mr(expt)	Mr(calc)	ppm	Start	End	Miss	Ions	Peptide
1004.5919	1003.5846	1003.6178	-33.12	87 -	95	1	33	K.LKLVGVGYR.A
1169.6591	1168.6518	1168.6564	-3.95	139 -	149	0	19	K.QVVGQVAAEIR.G

No match to: 832.4830, 864.4371, 870.5446, 897.4156, 919.5370, 973.5306, 974.4789, 986.5797, 1037.5306, 1060.5627, 1132.6766, 1143.5376, 1150.6438, 1152.6352, 1160.5670, 1193.6003, 1198.5902, 1230.5778, 1234.6676, 1261.6761, 1263.6787, 1277.6967, 1302.6902, 1307.6557, 1340.6607, 1357.6919, 1383.6631, 1390.6668, 1407.6965, 1434.7636, 1438.6220, 1458.6980, 1475.7261, 1487.7204, 1584.8657, 1638.8097, 1657.7630, 1687.9216, 1695.8492, 1699.7953, 1707.7412, 1838.8869, 1851.8805, 1888.8985, 1904.8884, 1940.8860, 2225.0763,

2239.0817, 2248.1493, 2252.0589, 2286.0247, 2399.0067, 2510.1289, 2705.2084

22. gi|32034702 **Mass:** 18918 **Score:** 63 **Expect:** 1.4 **Queries matched:** 4
COG0097: Ribosomal protein L6P/L9E [Actinobacillus pleuropneumoniae serovar 1 str. 4074]

Observed	Mr(expt)	Mr(calc)	ppm	Start		End	Miss	Ions	Peptide
1004.5919	1003.5846	1003.5815	3.13	87	-	95	0	33	K.LQLVGVGYR.A
1132.6766	1131.6693	1131.6764	-6.26	86	-	95	1	---	K.KLQLVGVGYR.A
1302.6902	1301.6829	1301.6324	38.8	56	-	69	0	---	R.TGVANADAQAGTAR.A
1584.8657	1583.8584	1583.8631	-2.95	135	-	149	1	---	K.SADKQLIGQVAADIR.A

No match to: 832.4830, 864.4371, 870.5446, 897.4156, 919.5370, 973.5306, 974.4789, 986.5797, 1037.5306, 1060.5627, 1143.5376, 1150.6438, 1152.6352, 1160.5670, 1169.6591, 1193.6003, 1198.5902, 1230.5778, 1234.6676, 1261.6761, 1263.6787, 1277.6967, 1307.6557, 1340.6607, 1357.6919, 1383.6631, 1390.6668, 1407.6965, 1434.7636, 1438.6220, 1458.6980, 1475.7261, 1487.7204, 1638.8097, 1657.7630, 1687.9216, 1695.8492, 1699.7953, 1707.7412, 1838.8869, 1851.8805, 1888.8985, 1904.8884, 1940.8860, 2225.0763, 2239.0817, 2248.1493, 2252.0589, 2286.0247, 2399.0067, 2510.1289, 2705.2084

23. gi|113460224 **Mass:** 19091 **Score:** 62 **Expect:** 1.7 **Queries matched:** 4
50S ribosomal protein L6 [Haemophilus somnus 129PT]

Observed	Mr(expt)	Mr(calc)	ppm	Start		End	Miss	Ions	Peptide
986.5797	985.5724	985.5808	-8.50	19	-	27	0	---	K.LDGQLLTVK.G
1004.5919	1003.5846	1003.5815	3.13	87	-	95	0	33	K.LQLVGVGYR.A
1132.6766	1131.6693	1131.6764	-6.26	86	-	95	1	---	K.KLQLVGVGYR.A
1584.8657	1583.8584	1583.8631	-2.95	135	-	149	1	---	K.SADKQLIGQVAADIR.A

No match to: 832.4830, 864.4371, 870.5446, 897.4156, 919.5370, 973.5306, 974.4789, 1037.5306, 1060.5627, 1143.5376, 1150.6438, 1152.6352, 1160.5670, 1169.6591, 1193.6003, 1198.5902, 1230.5778, 1234.6676, 1261.6761, 1263.6787, 1277.6967, 1302.6902, 1307.6557, 1340.6607, 1357.6919, 1383.6631, 1390.6668, 1407.6965, 1434.7636, 1438.6220, 1458.6980, 1475.7261, 1487.7204, 1638.8097, 1657.7630, 1687.9216, 1695.8492, 1699.7953, 1707.7412, 1838.8869, 1851.8805, 1888.8985, 1904.8884, 1940.8860, 2225.0763, 2239.0817, 2248.1493, 2252.0589, 2286.0247, 2399.0067, 2510.1289, 2705.2084

24. gi|32030990 **Mass:** 19042 **Score:** 62 **Expect:** 1.7 **Queries matched:** 4
COG0097: Ribosomal protein L6P/L9E [Haemophilus somnus 2336]

Observed	Mr(expt)	Mr(calc)	ppm	Start	End	Miss	Ions	Peptide
986.5797	985.5724	985.5808	-8.50	19 -	27	0	---	K.LDGQLLTVK.G
1004.5919	1003.5846	1003.5815	3.13	87 -	95	0	33	K.LQLVGVGYR.A
1132.6766	1131.6693	1131.6764	-6.26	86 -	95	1	---	K.KLQLVGVGYR.A
1584.8657	1583.8584	1583.8631	-2.95	135 -	149	1	---	K.SADKQLIGQVAADIR.A

No match to: 832.4830, 864.4371, 870.5446, 897.4156, 919.5370, 973.5306, 974.4789, 1037.5306, 1060.5627, 1143.5376, 1150.6438, 1152.6352, 1160.5670, 1169.6591, 1193.6003, 1198.5902, 1230.5778, 1234.6676, 1261.6761, 1263.6787, 1277.6967, 1302.6902, 1307.6557, 1340.6607, 1357.6919, 1383.6631, 1390.6668, 1407.6965, 1434.7636, 1438.6220, 1458.6980, 1475.7261, 1487.7204, 1638.8097, 1657.7630, 1687.9216, 1695.8492, 1699.7953, 1707.7412, 1838.8869, 1851.8805, 1888.8985, 1904.8884, 1940.8860, 2225.0763, 2239.0817, 2248.1493, 2252.0589, 2286.0247, 2399.0067, 2510.1289, 2705.2084

25. gi|54293560 **Mass:** 55446 **Score:** 62 **Expect:** 1.9 **Queries matched:** 10

hypothetical protein lpl0612 [Legionella pneumophila str. Lens]

Observed	Mr(expt)	Mr(calc)	ppm	Start	End	Miss	Ions	Peptide
832.4830	831.4758	831.4702	6.70	235 -	242	0	---	R.ITISGVDK.L
1004.5919	1003.5846	1003.5702	14.3	355 -	362	0	---	R.FQIVQELK.K
1060.5627	1059.5554	1059.5309	23.2	385 -	394	0	---	R.GSQVNATDLR.S
1132.6766	1131.6693	1131.6652	3.68	355 -	363	1	---	R.FQIVQELKK.M
1152.6352	1151.6280	1151.6550	-23.47	56 -	65	0	---	K.EPLQLTNLPK.I
1234.6676	1233.6604	1233.6717	-9.22	173 -	183	0	---	R.LQGNTINFSIK.T
1277.6967	1276.6894	1276.6371	41.0	456 -	466	1	---	K.EATSSETRIQR.A
1357.6919	1356.6846	1356.7249	-29.66	486 -	497	1	---	K.RVPTGEVTELEK.Q
1487.7204	1486.7131	1486.7813	-45.87	93 -	105	1	---	K.IGNREMDTLTIPK.E
1851.8805	1850.8732	1850.8940	-11.23	66 -	81	0	---	K.ILDVTNMIQCMEALGK.R + Oxi

No match to: 864.4371, 870.5446, 897.4156, 919.5370, 973.5306, 974.4789, 986.5797, 1037.5306, 1143.5376, 1150.6438, 1160.5670, 1169.6591, 1193.6003, 1198.5902, 1230.5778, 1261.6761, 1263.6787, 1302.6902, 1307.6557, 1340.6607, 1383.6631, 1390.6668, 1407.6965, 1434.7636, 1438.6220, 1458.6980, 1475.7261, 1584.8657, 1638.8097, 1657.7630, 1687.9216, 1695.8492, 1699.7953, 1707.7412, 1838.8869, 1888.8985, 1904.8884, 1940.8860, 2225.0763, 2239.0817, 2248.1493, 2252.0589, 2286.0247, 2399.0067, 2510.1289, 2705.2084

26. gi|148360776 **Mass:** 55461 **Score:** 62 **Expect:** 1.9 **Queries matched:** 10

UDP-N-acetylglucosamine 1- carboxyvinyltransferase [Legionella pneumophila str. Corby]

Observed	Mr(expt)	Mr(calc)	ppm	Start	End	Miss	Ions	Peptide
832.4830	831.4758	831.4702	6.70	235 -	242	0	---	R.ITISGVDK.L
1004.5919	1003.5846	1003.5702	14.3	355 -	362	0	---	R.FQIVQELK.K
1060.5627	1059.5554	1059.5309	23.2	385 -	394	0	---	R.GSQVNATDLR.S
1132.6766	1131.6693	1131.6652	3.68	355 -	363	1	---	R.FQIVQELKK.M
1152.6352	1151.6280	1151.6550	-23.47	56 -	65	0	---	K.EPLQLTNLPK.I
1234.6676	1233.6604	1233.6717	-9.22	173 -	183	0	---	R.LQGNTINFSIK.T
1277.6967	1276.6894	1276.6371	41.0	456 -	466	1	---	K.EATSSETRIQR.A
1357.6919	1356.6846	1356.7249	-29.66	486 -	497	1	---	K.RVPTGEVTELEK.Q
1487.7204	1486.7131	1486.7813	-45.87	93 -	105	1	---	K.IGNREMDTLTIPK.E
1851.8805	1850.8732	1850.8940	-11.23	66 -	81	0	---	K.ILDVTNMIQCMEALGK.R + Oxi

No match to: 864.4371, 870.5446, 897.4156, 919.5370, 973.5306, 974.4789, 986.5797, 1037.5306, 1143.5376, 1150.6438, 1160.5670, 1169.6591, 1193.6003, 1198.5902, 1230.5778, 1261.6761, 1263.6787, 1302.6902, 1307.6557, 1340.6607, 1383.6631, 1390.6668, 1407.6965, 1434.7636, 1438.6220, 1458.6980, 1475.7261, 1584.8657, 1638.8097, 1657.7630, 1687.9216, 1695.8492, 1699.7953, 1707.7412, 1838.8869, 1888.8985, 1904.8884, 1940.8860, 2225.0763, 2239.0817, 2248.1493, 2252.0589, 2286.0247, 2399.0067, 2510.1289, 2705.2084

27. gi|54307556 **Mass:** 18923 **Score:** 62 **Expect:** 1.9 **Queries matched:** 1

50S ribosomal protein L6 [Photobacterium profundum SS9]

Observed	Mr(expt)	Mr(calc)	ppm	Start	End	Miss	Ions	Peptide
1169.6591	1168.6518	1168.6564	-3.95	139 -	149	0	57	K.QVIGQVAADIR.A

No match to: 832.4830, 864.4371, 870.5446, 897.4156, 919.5370, 973.5306, 974.4789, 986.5797, 1004.5919, 1037.5306, 1060.5627, 1132.6766, 1143.5376, 1150.6438, 1152.6352, 1160.5670, 1193.6003, 1198.5902, 1230.5778, 1234.6676, 1261.6761, 1263.6787, 1277.6967, 1302.6902, 1307.6557, 1340.6607, 1357.6919, 1383.6631, 1390.6668, 1407.6965, 1434.7636, 1438.6220, 1458.6980, 1475.7261, 1487.7204, 1584.8657, 1638.8097, 1657.7630, 1687.9216, 1695.8492, 1699.7953, 1707.7412, 1838.8869, 1851.8805, 1888.8985, 1904.8884, 1940.8860, 2225.0763, 2239.0817, 2248.1493, 2252.0589, 2286.0247, 2399.0067, 2510.1289, 2705.2084

28. gi|90414971 **Mass:** 18856 **Score:** 62 **Expect:** 1.9 **Queries matched:** 1

50S ribosomal protein L6 [Photobacterium profundum 3TCK]

Observed	Mr(expt)	Mr(calc)	ppm	Start	End	Miss	Ions	Peptide

1169.6591	1168.6518	1168.6564	-3.95	139 - 149	0	57	K.QVIGQVAADIR.A

No match to: 832.4830, 864.4371, 870.5446, 897.4156, 919.5370, 973.5306, 974.4789, 986.5797, 1004.5919, 1037.5306, 1060.5627, 1132.6766, 1143.5376, 1150.6438, 1152.6352, 1160.5670, 1193.6003, 1198.5902, 1230.5778, 1234.6676, 1261.6761, 1263.6787, 1277.6967, 1302.6902, 1307.6557, 1340.6607, 1357.6919, 1383.6631, 1390.6668, 1407.6965, 1434.7636, 1438.6220, 1458.6980, 1475.7261, 1487.7204, 1584.8657, 1638.8097, 1657.7630, 1687.9216, 1695.8492, 1699.7953, 1707.7412, 1838.8869, 1851.8805, 1888.8985, 1904.8884, 1940.8860, 2225.0763, 2239.0817, 2248.1493, 2252.0589, 2286.0247, 2399.0067, 2510.1289, 2705.2084

29. gi|52426088 **Mass:** 19026 **Score:** 62 **Expect:** 1.9 **Queries matched:** 4
50S ribosomal protein L6 [Mannheimia succiniciproducens MBEL55E]

Observed	Mr(expt)	Mr(calc)	ppm	Start	End	Miss	Ions	Peptide
986.5797	985.5724	985.5808	-8.50	19 -	27	0	---	K.LDGQLLTVK.G
1004.5919	1003.5846	1003.5815	3.13	87 -	95	0	33	K.LQLVGVGYR.A
1132.6766	1131.6693	1131.6764	-6.26	86 -	95	1	---	K.KLQLVGVGYR.A
1152.6352	1151.6280	1151.6550	-23.50	7 -	18	0	---	K.APVSVPAGVEVK.L

No match to: 832.4830, 864.4371, 870.5446, 897.4156, 919.5370, 973.5306, 974.4789, 1037.5306, 1060.5627, 1143.5376, 1150.6438, 1160.5670, 1169.6591, 1193.6003, 1198.5902, 1230.5778, 1234.6676, 1261.6761, 1263.6787, 1277.6967, 1302.6902, 1307.6557, 1340.6607, 1357.6919, 1383.6631, 1390.6668, 1407.6965, 1434.7636, 1438.6220, 1458.6980, 1475.7261, 1487.7204, 1584.8657, 1638.8097, 1657.7630, 1687.9216, 1695.8492, 1699.7953, 1707.7412, 1838.8869, 1851.8805, 1888.8985, 1904.8884, 1940.8860, 2225.0763, 2239.0817, 2248.1493, 2252.0589, 2286.0247, 2399.0067, 2510.1289, 2705.2084

30. gi|146313370 **Mass:** 18843 **Score:** 61 **Expect:** 2 **Queries matched:** 4
ribosomal protein L6 [Enterobacter sp. 638]

Observed	Mr(expt)	Mr(calc)	ppm	Start	End	Miss	Ions	Peptide
986.5797	985.5724	985.5808	-8.50	19 -	27	0	---	K.IDGQVITIK.G
1004.5919	1003.5846	1003.5815	3.13	87 -	95	0	33	K.LQLVGVGYR.A
1132.6766	1131.6693	1131.6764	-6.26	86 -	95	1	---	K.KLQLVGVGYR.A
1198.5902	1197.5829	1197.5891	-5.15	45 -	55	0	---	K.HADNALTFGPR.D

No match to: 832.4830, 864.4371, 870.5446, 897.4156, 919.5370, 973.5306, 974.4789, 1037.5306, 1060.5627, 1143.5376, 1150.6438, 1152.6352, 1160.5670, 1169.6591, 1193.6003, 1230.5778, 1234.6676, 1261.6761, 1263.6787, 1277.6967, 1302.6902, 1307.6557, 1340.6607,

1357.6919, 1383.6631, 1390.6668, 1407.6965, 1434.7636, 1438.6220, 1458.6980, 1475.7261, 1487.7204, 1584.8657, 1638.8097, 1657.7630, 1687.9216, 1695.8492, 1699.7953, 1707.7412, 1838.8869, 1851.8805, 1888.8985, 1904.8884, 1940.8860, 2225.0763, 2239.0817, 2248.1493, 2252.0589, 2286.0247, 2399.0067, 2510.1289, 2705.2084

31. gi|28897046 **Mass:** 18881 **Score:** 59 **Expect:** 3.2 **Queries matched:** 2
50S ribosomal protein L6 [Vibrio parahaemolyticus RIMD 2210633]

Observed	Mr(expt)	Mr(calc)	ppm	Start	End	Miss	Ions	Peptide
1150.6438	1149.6365	1149.6758	-34.11	7 -	18	0	---	K.APVAIPAGVEVK.L
1169.6591	1168.6518	1168.6564	-3.95	139 -	149	0	48	K.QLVGQVAADIR.S

No match to: 832.4830, 864.4371, 870.5446, 897.4156, 919.5370, 973.5306, 974.4789, 986.5797, 1004.5919, 1037.5306, 1060.5627, 1132.6766, 1143.5376, 1152.6352, 1160.5670, 1193.6003, 1198.5902, 1230.5778, 1234.6676, 1261.6761, 1263.6787, 1277.6967, 1302.6902, 1307.6557, 1340.6607, 1357.6919, 1383.6631, 1390.6668, 1407.6965, 1434.7636, 1438.6220, 1458.6980, 1475.7261, 1487.7204, 1584.8657, 1638.8097, 1657.7630, 1687.9216, 1695.8492, 1699.7953, 1707.7412, 1838.8869, 1851.8805, 1888.8985, 1904.8884, 1940.8860, 2225.0763, 2239.0817, 2248.1493, 2252.0589, 2286.0247, 2399.0067, 2510.1289, 2705.2084

32. gi|91228963 **Mass:** 18792 **Score:** 59 **Expect:** 3.2 **Queries matched:** 2
50S ribosomal protein L6 [Vibrio alginolyticus 12G01]

Observed	Mr(expt)	Mr(calc)	ppm	Start	End	Miss	Ions	Peptide
1150.6438	1149.6365	1149.6758	-34.11	7 -	18	0	---	K.APVAIPAGVEVK.L
1169.6591	1168.6518	1168.6564	-3.95	139 -	149	0	48	K.QLVGQVAADIR.S

No match to: 832.4830, 864.4371, 870.5446, 897.4156, 919.5370, 973.5306, 974.4789, 986.5797, 1004.5919, 1037.5306, 1060.5627, 1132.6766, 1143.5376, 1152.6352, 1160.5670, 1193.6003, 1198.5902, 1230.5778, 1234.6676, 1261.6761, 1263.6787, 1277.6967, 1302.6902, 1307.6557, 1340.6607, 1357.6919, 1383.6631, 1390.6668, 1407.6965, 1434.7636, 1438.6220, 1458.6980, 1475.7261, 1487.7204, 1584.8657, 1638.8097, 1657.7630, 1687.9216, 1695.8492, 1699.7953, 1707.7412, 1838.8869, 1851.8805, 1888.8985, 1904.8884, 1940.8860, 2225.0763, 2239.0817, 2248.1493, 2252.0589, 2286.0247, 2399.0067, 2510.1289, 2705.2084

33. gi|156973066 **Mass:** 18867 **Score:** 59 **Expect:** 3.2 **Queries matched:** 2
ribosomal protein L6P/L9E [Vibrio harveyi ATCC BAA-1116]

Observed	Mr(expt)	Mr(calc)	ppm	Start	End	Miss	Ions	Peptide

1150.6438	1149.6365	1149.6758	-34.11	7	- 18	0	---	K.APVAIPAGVEVK.L
1169.6591	1168.6518	1168.6564	-3.95	139	- 149	0	48	K.QLVGQVAADIR.S

No match to: 832.4830, 864.4371, 870.5446, 897.4156, 919.5370, 973.5306, 974.4789, 986.5797, 1004.5919, 1037.5306, 1060.5627, 1132.6766, 1143.5376, 1152.6352, 1160.5670, 1193.6003, 1198.5902, 1230.5778, 1234.6676, 1261.6761, 1263.6787, 1277.6967, 1302.6902, 1307.6557, 1340.6607, 1357.6919, 1383.6631, 1390.6668, 1407.6965, 1434.7636, 1438.6220, 1458.6980, 1475.7261, 1487.7204, 1584.8657, 1638.8097, 1657.7630, 1687.9216, 1695.8492, 1699.7953, 1707.7412, 1838.8869, 1851.8805, 1888.8985, 1904.8884, 1940.8860, 2225.0763, 2239.0817, 2248.1493, 2252.0589, 2286.0247, 2399.0067, 2510.1289, 2705.2084

34. gi|154500500 **Mass:** 6910 **Score:** 59 **Expect:** 3.4 **Queries matched:** 3
hypothetical protein BACCAP_04173 [Bacteroides capillosus ATCC 29799]

Observed	Mr(expt)	Mr(calc)	ppm	Start	End	Miss	Ions	Peptide
832.4830	831.4758	831.4563	23.4	49	- 57	1	---	R.TGLGSGGKR.K
1169.6591	1168.6518	1168.6200	27.2	2	- 13	0	33	M.PSAAANIAADLR.F
1198.5902	1197.5829	1197.5700	10.8	14	- 23	0	---	R.FIETSISCNK.K

No match to: 864.4371, 870.5446, 897.4156, 919.5370, 973.5306, 974.4789, 986.5797, 1004.5919, 1037.5306, 1060.5627, 1132.6766, 1143.5376, 1150.6438, 1152.6352, 1160.5670, 1193.6003, 1230.5778, 1234.6676, 1261.6761, 1263.6787, 1277.6967, 1302.6902, 1307.6557, 1340.6607, 1357.6919, 1383.6631, 1390.6668, 1407.6965, 1434.7636, 1438.6220, 1458.6980, 1475.7261, 1487.7204, 1584.8657, 1638.8097, 1657.7630, 1687.9216, 1695.8492, 1699.7953, 1707.7412, 1838.8869, 1851.8805, 1888.8985, 1904.8884, 1940.8860, 2225.0763, 2239.0817, 2248.1493, 2252.0589, 2286.0247, 2399.0067, 2510.1289, 2705.2084

35. gi|148982236 **Mass:** 18859 **Score:** 59 **Expect:** 3.5 **Queries matched:** 2
50S ribosomal protein L6 [Vibrionales bacterium SWAT-3]

Observed	Mr(expt)	Mr(calc)	ppm	Start	End	Miss	Ions	Peptide
1150.6438	1149.6365	1149.6758	-34.11	7	- 18	0	---	K.APVAIPAGVEVK.L
1169.6591	1168.6518	1168.6564	-3.95	139	- 149	0	48	K.QLVGQVAADIR.S

No match to: 832.4830, 864.4371, 870.5446, 897.4156, 919.5370, 973.5306, 974.4789, 986.5797, 1004.5919, 1037.5306, 1060.5627, 1132.6766, 1143.5376, 1152.6352, 1160.5670, 1193.6003, 1198.5902, 1230.5778, 1234.6676, 1261.6761, 1263.6787, 1277.6967, 1302.6902, 1307.6557, 1340.6607, 1357.6919, 1383.6631, 1390.6668, 1407.6965, 1434.7636, 1438.6220, 1458.6980, 1475.7261, 1487.7204, 1584.8657, 1638.8097, 1657.7630, 1687.9216, 1695.8492,

1699.7953, 1707.7412, 1838.8869, 1851.8805, 1888.8985, 1904.8884, 1940.8860, 2225.0763, 2239.0817, 2248.1493, 2252.0589, 2286.0247, 2399.0067, 2510.1289, 2705.2084

36. gi|146312705 **Mass:** 14384 **Score:** 59 **Expect:** 3.5 **Queries matched:** 6
formate C-acetyltransferase glycine radical [Enterobacter sp. 638]

Observed	Mr(expt)	Mr(calc)	ppm	Start	End	Miss	Ions	Peptide
1004.5919	1003.5846	1003.5736	11.0	1 -	9	0	---	-.MITGIQITK.A
1060.5627	1059.5554	1059.5560	-0.54	80 -	88	1	---	R.RETLEDAVK.H
1193.6003	1192.5930	1192.6452	-43.76	117 -	127	1	---	R.DVIARTFTASL.-
1340.6607	1339.6534	1339.6806	-20.28	56 -	66	0	---	R.EIPMEIQPEVR.V
1434.7636	1433.7563	1433.7739	-12.27	67 -	79	0	---	R.VEGGQHLNVNVLR.R
2248.1493	2247.1420	2247.1535	-5.12	36 -	55	1	---	K.AGFAEDQIVPVNKLGEIEYR.E

No match to: 832.4830, 864.4371, 870.5446, 897.4156, 919.5370, 973.5306, 974.4789, 986.5797, 1037.5306, 1132.6766, 1143.5376, 1150.6438, 1152.6352, 1160.5670, 1169.6591, 1198.5902, 1230.5778, 1234.6676, 1261.6761, 1263.6787, 1277.6967, 1302.6902, 1307.6557, 1357.6919, 1383.6631, 1390.6668, 1407.6965, 1438.6220, 1458.6980, 1475.7261, 1487.7204, 1584.8657, 1638.8097, 1657.7630, 1687.9216, 1695.8492, 1699.7953, 1707.7412, 1838.8869, 1851.8805, 1888.8985, 1904.8884, 1940.8860, 2225.0763, 2239.0817, 2252.0589, 2286.0247, 2399.0067, 2510.1289, 2705.2084

37. gi|149192384 **Mass:** 22354 **Score:** 58 **Expect:** 3.9 **Queries matched:** 2
50S ribosomal protein L6 [Vibrio shilonii AK1]

Observed	Mr(expt)	Mr(calc)	ppm	Start	End	Miss	Ions	Peptide
1150.6438	1149.6365	1149.6758	-34.11	7 -	18	0	---	K.APVAIPAGVEVK.L
1169.6591	1168.6518	1168.6564	-3.95	139 -	149	0	48	K.QLVGQVAADIR.S

No match to: 832.4830, 864.4371, 870.5446, 897.4156, 919.5370, 973.5306, 974.4789, 986.5797, 1004.5919, 1037.5306, 1060.5627, 1132.6766, 1143.5376, 1152.6352, 1160.5670, 1193.6003, 1198.5902, 1230.5778, 1234.6676, 1261.6761, 1263.6787, 1277.6967, 1302.6902, 1307.6557, 1340.6607, 1357.6919, 1383.6631, 1390.6668, 1407.6965, 1434.7636, 1438.6220, 1458.6980, 1475.7261, 1487.7204, 1584.8657, 1638.8097, 1657.7630, 1687.9216, 1695.8492, 1699.7953, 1707.7412, 1838.8869, 1851.8805, 1888.8985, 1904.8884, 1940.8860, 2225.0763, 2239.0817, 2248.1493, 2252.0589, 2286.0247, 2399.0067, 2510.1289, 2705.2084

38. gi|153806077 **Mass:** 67846 **Score:** 57 **Expect:** 5.4 **Queries matched:** 10

hypothetical protein BACCAC_00328 [Bacteroides caccae ATCC 43185]

Observed	Mr(expt)	Mr(calc)	ppm	Start	End	Miss	Ions	Peptide
974.4789	973.4717	973.4658	6.07	2 -	8	0	---	M.DLFNYFR.R
1004.5919	1003.5846	1003.6066	-21.91	283 -	290	0	---	K.LVDYILLR.Q
1132.6766	1131.6693	1131.7016	-28.46	282 -	290	1	---	R.KLVDYILLR.Q
1143.5376	1142.5303	1142.5455	-13.27	590 -	599	0	---	K.NIPEEEAVDK.L
1234.6676	1233.6604	1233.6466	11.2	49 -	59	1	---	K.RIVDAGGEYVR.L
1261.6761	1260.6688	1260.6073	48.7	1 -	9	1	---	-.MDLFNYFRR.E
1475.7261	1474.7188	1474.7892	-47.75	319 -	332	0	---	R.NIGGEHVPVVIADR.M
1657.7630	1656.7557	1656.8366	-48.81	159 -	173	1	---	R.IGVNHGSLSDRIMSR.Y + Oxid
1695.8492	1694.8419	1694.9203	-46.22	267 -	282	1	---	R.VSLSEAPEAEIPVARK.L
2399.0067	2397.9994	2398.0853	-35.82	29 -	49	1	---	R.IQSMTNTSTQDTQACVDQAKR.I

No match to: 832.4830, 864.4371, 870.5446, 897.4156, 919.5370, 973.5306, 986.5797, 1037.5306, 1060.5627, 1150.6438, 1152.6352, 1160.5670, 1169.6591, 1193.6003, 1198.5902, 1230.5778, 1263.6787, 1277.6967, 1302.6902, 1307.6557, 1340.6607, 1357.6919, 1383.6631, 1390.6668, 1407.6965, 1434.7636, 1438.6220, 1458.6980, 1487.7204, 1584.8657, 1638.8097, 1687.9216, 1699.7953, 1707.7412, 1838.8869, 1851.8805, 1888.8985, 1904.8884, 1940.8860, 2225.0763, 2239.0817, 2248.1493, 2252.0589, 2286.0247, 2510.1289, 2705.2084

39. gi|94317657 **Mass:** 6979 **Score:** 56 **Expect:** 6.7 **Queries matched:** 5

hypothetical protein [Salmonella enterica subsp. enterica serovar Typhimurium]

Observed	Mr(expt)	Mr(calc)	ppm	Start	End	Miss	Ions	Peptide
1037.5306	1036.5233	1036.5665	-41.68	21 -	29	1	---	R.IYKATANTR.G
1277.6967	1276.6894	1276.6915	-1.58	45 -	55	0	---	R.DLVIEIYLDGK.G
1638.8097	1637.8024	1637.7654	22.6	7 -	20	1	---	R.QCAVPGTMVKYNDR.I
1851.8805	1850.8732	1850.9053	-17.31	1 -	16	1	---	-.TMEVIRQCAVPGTMVK.Y + 2 O
1888.8985	1887.8912	1887.9829	-48.59	45 -	61	1	---	R.DLVIEIYLDGKGEPLTN.-

No match to: 832.4830, 864.4371, 870.5446, 897.4156, 919.5370, 973.5306, 974.4789, 986.5797, 1004.5919, 1060.5627, 1132.6766, 1143.5376, 1150.6438, 1152.6352, 1160.5670, 1169.6591, 1193.6003, 1198.5902, 1230.5778, 1234.6676, 1261.6761, 1263.6787, 1302.6902, 1307.6557, 1340.6607, 1357.6919, 1383.6631, 1390.6668, 1407.6965, 1434.7636, 1438.6220, 1458.6980, 1475.7261, 1487.7204, 1584.8657, 1657.7630, 1687.9216, 1695.8492, 1699.7953, 1707.7412, 1838.8869, 1904.8884, 1940.8860, 2225.0763, 2239.0817, 2248.1493, 2252.0589,

2286.0247, 2399.0067, 2510.1289, 2705.2084

40. gi|89074671 **Mass:** 87370 **Score:** 55 **Expect:** 9.1 **Queries matched:** 11
hypothetical membrane carboxypeptidase [Photobacterium sp. SKA34]

Observed	Mr(expt)	Mr(calc)	ppm	Start		End	Miss	Ions	Peptide
973.5306	972.5233	972.5678	-45.72	415	-	422	1	---	K.KMIPLVEK.R + Oxidation (
974.4789	973.4717	973.4981	-27.18	636	-	644	0	---	K.FGTAHSLNK.L
1143.5376	1142.5303	1142.5680	-32.95	12	-	21	0	---	K.QDKPQPSSTR.K
1263.6787	1262.6715	1262.6580	10.6	173	-	182	1	---	R.IYLPEEKMPK.L + Oxidation
1277.6967	1276.6894	1276.6412	37.8	385	-	396	1	---	K.VGDAFEEGKGLR.L
1302.6902	1301.6829	1301.7377	-42.07	333	-	343	1	---	R.NVVLKIMLDNK.L + Oxidatio
1458.6980	1457.6907	1457.7303	-27.17	292	-	302	0	---	R.YYFDRPLSELR.S
1888.8985	1887.8912	1887.9836	-48.95	427	-	443	1	---	K.NLQTAMVIADRTTGEIR.A
2225.0763	2224.0690	2224.0735	-2.06	663	-	681	1	---	K.DSWYVGIDGREVVTVWMGR.D
2248.1493	2247.1420	2247.0967	20.2	444	-	464	1	---	R.AMIGGSNPNFPGYNRAINAQR.Q
2252.0589	2251.0516	2251.0804	-12.79	438	-	458	1	---	R.TTGEIRAMIGGSNPNFPGYNR.A

No match to: 832.4830, 864.4371, 870.5446, 897.4156, 919.5370, 986.5797, 1004.5919, 1037.5306, 1060.5627, 1132.6766, 1150.6438, 1152.6352, 1160.5670, 1169.6591, 1193.6003, 1198.5902, 1230.5778, 1234.6676, 1261.6761, 1307.6557, 1340.6607, 1357.6919, 1383.6631, 1390.6668, 1407.6965, 1434.7636, 1438.6220, 1475.7261, 1487.7204, 1584.8657, 1638.8097, 1657.7630, 1687.9216, 1695.8492, 1699.7953, 1707.7412, 1838.8869, 1851.8805, 1904.8884, 1940.8860, 2239.0817, 2286.0247, 2399.0067, 2510.1289, 2705.2084

41. gi|85060241 **Mass:** 18971 **Score:** 54 **Expect:** 11 **Queries matched:** 3
50S ribosomal protein L6 [Sodalis glossinidius str. 'morsitans']

Observed	Mr(expt)	Mr(calc)	ppm	Start		End	Miss	Ions	Peptide
1004.5919	1003.5846	1003.5815	3.13	87	-	95	0	33	K.LQLVGVGYR.A
1132.6766	1131.6693	1131.6764	-6.26	86	-	95	1	---	K.KLQLVGVGYR.A
2248.1493	2247.1420	2247.1032	17.3	36	-	55	0	---	R.TIHEAVDVQHADNQLSFAPR.E

No match to: 832.4830, 864.4371, 870.5446, 897.4156, 919.5370, 973.5306, 974.4789, 986.5797, 1037.5306, 1060.5627, 1143.5376, 1150.6438, 1152.6352, 1160.5670, 1169.6591, 1193.6003, 1198.5902, 1230.5778, 1234.6676, 1261.6761, 1263.6787, 1277.6967, 1302.6902, 1307.6557, 1340.6607, 1357.6919, 1383.6631, 1390.6668, 1407.6965, 1434.7636, 1438.6220,

1458.6980, 1475.7261, 1487.7204, 1584.8657, 1638.8097, 1657.7630, 1687.9216, 1695.8492, 1699.7953, 1707.7412, 1838.8869, 1851.8805, 1888.8985, 1904.8884, 1940.8860, 2225.0763, 2239.0817, 2252.0589, 2286.0247, 2399.0067, 2510.1289, 2705.2084

42. gi|81176852 **Mass:** 81296 **Score:** 54 **Expect:** 11 **Queries matched:** 12
Polyribonucleotide nucleotidyltransferase [Streptococcus suis 89/1591]

Observed	Mr(expt)	Mr(calc)	ppm	Start	End	Miss	Ions	Peptide
832.4830	831.4758	831.4450	37.0	606 -	612	1	---	R.DAIERTK.E
870.5446	869.5373	869.5334	4.46	613 -	620	0	---	K.EIIAGLVR.E
1152.6352	1151.6280	1151.5710	49.5	273 -	282	0	---	R.ETVIAAYEEK.Y
1263.6787	1262.6715	1262.6507	16.5	621 -	631	1	---	R.EAKVDEVFQAK.V
1340.6607	1339.6534	1339.7136	-44.95	2 -	13	1	---	M.SKQVFETVFAGK.K
1458.6980	1457.6907	1457.7072	-11.33	34 -	47	0	---	R.YGDSTVLTAAVMSK.K + Oxida
1475.7261	1474.7188	1474.6737	30.6	65 -	77	1	---	K.MYAAGKFPGGWMK.R + 2 Oxid
1487.7204	1486.7131	1486.7490	-24.12	1 -	13	1	---	-.MSKQVFETVFAGK.K + Oxidat
1888.8985	1887.8912	1887.8713	10.5	49 -	64	0	---	K.MATGDFFPLQVNYEEK.M
1904.8884	1903.8811	1903.8662	7.83	49 -	64	0	---	K.MATGDFFPLQVNYEEK.M + Oxi
2252.0589	2251.0516	2251.1089	-25.45	292 -	309	1	---	R.IMRDVHEILELMEHAEVR.R + 2
2510.1289	2509.1217	2509.1657	-17.57	49 -	70	1	---	K.MATGDFFPLQVNYEEKMYAAGK.F

No match to: 864.4371, 897.4156, 919.5370, 973.5306, 974.4789, 986.5797, 1004.5919, 1037.5306, 1060.5627, 1132.6766, 1143.5376, 1150.6438, 1160.5670, 1169.6591, 1193.6003, 1198.5902, 1230.5778, 1234.6676, 1261.6761, 1277.6967, 1302.6902, 1307.6557, 1357.6919, 1383.6631, 1390.6668, 1407.6965, 1434.7636, 1438.6220, 1584.8657, 1638.8097, 1657.7630, 1687.9216, 1695.8492, 1699.7953, 1707.7412, 1838.8869, 1851.8805, 1940.8860, 2225.0763, 2239.0817, 2248.1493, 2286.0247, 2399.0067, 2705.2084

43. gi|94312233 **Mass:** 18896 **Score:** 54 **Expect:** 11 **Queries matched:** 4
ribosomal protein L6 [Ralstonia metallidurans CH34]

Observed	Mr(expt)	Mr(calc)	ppm	Start	End	Miss	Ions	Peptide
1004.5919	1003.5846	1003.5815	3.13	87 -	95	0	33	K.LQLVGVGYR.A
1132.6766	1131.6693	1131.6764	-6.26	86 -	95	1	---	R.KLQLVGVGYR.A
1888.8985	1887.8912	1887.9658	-39.53	60 -	77	1	---	R.EANALQGTMRALAANMVK.G
1904.8884	1903.8811	1903.9608	-41.83	60 -	77	1	---	R.EANALQGTMRALAANMVK.G + O

No match to: 832.4830, 864.4371, 870.5446, 897.4156, 919.5370, 973.5306, 974.4789, 986.5797, 1037.5306, 1060.5627, 1143.5376, 1150.6438, 1152.6352, 1160.5670, 1169.6591, 1193.6003, 1198.5902, 1230.5778, 1234.6676, 1261.6761, 1263.6787, 1277.6967, 1302.6902, 1307.6557, 1340.6607, 1357.6919, 1383.6631, 1390.6668, 1407.6965, 1434.7636, 1438.6220, 1458.6980, 1475.7261, 1487.7204, 1584.8657, 1638.8097, 1657.7630, 1687.9216, 1695.8492, 1699.7953, 1707.7412, 1838.8869, 1851.8805, 1940.8860, 2225.0763, 2239.0817, 2248.1493, 2252.0589, 2286.0247, 2399.0067, 2510.1289, 2705.2084

44. gi|116625125 **Mass:** 27271 **Score:** 54 **Expect:** 12 **Queries matched:** 7
hypothetical protein Acid_6062 [Solibacter usitatus Ellin6076]

Observed	Mr(expt)	Mr(calc)	ppm	Start	End	Miss	Ions	Peptide
832.4830	831.4758	831.4385	44.8	79 -	84	1	---	R.REVCIR.H
1169.6591	1168.6518	1168.7040	-44.70	155 -	164	1	8	R.RGVTLVQLQR.A
1407.6965	1406.6893	1406.7122	-16.34	127 -	138	1	---	R.VRQMLSGTACIR.I + Oxidati
1687.9216	1686.9143	1686.8763	22.5	51 -	64	0	---	R.MVWEAIDLANLLQR.D + Oxida
1695.8492	1694.8419	1694.7900	30.6	111 -	125	0	---	K.SPVEEFGVDQNGYVR.R
1707.7412	1706.7339	1706.7894	-32.50	65 -	78	1	---	R.DRQSVTDSLEELCR.R
1851.8805	1850.8732	1850.8911	-9.67	111 -	126	1	---	K.SPVEEFGVDQNGYVRR.V

No match to: 864.4371, 870.5446, 897.4156, 919.5370, 973.5306, 974.4789, 986.5797, 1004.5919, 1037.5306, 1060.5627, 1132.6766, 1143.5376, 1150.6438, 1152.6352, 1160.5670, 1193.6003, 1198.5902, 1230.5778, 1234.6676, 1261.6761, 1263.6787, 1277.6967, 1302.6902, 1307.6557, 1340.6607, 1357.6919, 1383.6631, 1390.6668, 1434.7636, 1438.6220, 1458.6980, 1475.7261, 1487.7204, 1584.8657, 1638.8097, 1657.7630, 1699.7953, 1838.8869, 1888.8985, 1904.8884, 1940.8860, 2225.0763, 2239.0817, 2248.1493, 2252.0589, 2286.0247, 2399.0067, 2510.1289, 2705.2084

45. gi|89076309 **Mass:** 18871 **Score:** 53 **Expect:** 13 **Queries matched:** 1
50S ribosomal protein L6 [Photobacterium sp. SKA34]

Observed	Mr(expt)	Mr(calc)	ppm	Start	End	Miss	Ions	Peptide
1169.6591	1168.6518	1168.6564	-3.95	139 -	149	0	48	K.QLVGQVAADIR.A

No match to: 832.4830, 864.4371, 870.5446, 897.4156, 919.5370, 973.5306, 974.4789, 986.5797, 1004.5919, 1037.5306, 1060.5627, 1132.6766, 1143.5376, 1150.6438, 1152.6352, 1160.5670, 1193.6003, 1198.5902, 1230.5778, 1234.6676, 1261.6761, 1263.6787, 1277.6967, 1302.6902, 1307.6557, 1340.6607, 1357.6919, 1383.6631, 1390.6668, 1407.6965, 1434.7636,

1438.6220, 1458.6980, 1475.7261, 1487.7204, 1584.8657, 1638.8097, 1657.7630, 1687.9216, 1695.8492, 1699.7953, 1707.7412, 1838.8869, 1851.8805, 1888.8985, 1904.8884, 1940.8860, 2225.0763, 2239.0817, 2248.1493, 2252.0589, 2286.0247, 2399.0067, 2510.1289, 2705.2084

46. gi|59710859 **Mass:** 19010 **Score:** 53 **Expect:** 13 **Queries matched:** 1
50S ribosomal protein L6 [Vibrio fischeri ES114]

Observed	Mr(expt)	Mr(calc)	ppm	Start	End	Miss	Ions	Peptide
1169.6591	1168.6518	1168.6564	-3.95	139 -	149	0	48	K.QLVGQVAADLR.S

No match to: 832.4830, 864.4371, 870.5446, 897.4156, 919.5370, 973.5306, 974.4789, 986.5797, 1004.5919, 1037.5306, 1060.5627, 1132.6766, 1143.5376, 1150.6438, 1152.6352, 1160.5670, 1193.6003, 1198.5902, 1230.5778, 1234.6676, 1261.6761, 1263.6787, 1277.6967, 1302.6902, 1307.6557, 1340.6607, 1357.6919, 1383.6631, 1390.6668, 1407.6965, 1434.7636, 1438.6220, 1458.6980, 1475.7261, 1487.7204, 1584.8657, 1638.8097, 1657.7630, 1687.9216, 1695.8492, 1699.7953, 1707.7412, 1838.8869, 1851.8805, 1888.8985, 1904.8884, 1940.8860, 2225.0763, 2239.0817, 2248.1493, 2252.0589, 2286.0247, 2399.0067, 2510.1289, 2705.2084

47. gi|90581693 **Mass:** 18952 **Score:** 53 **Expect:** 13 **Queries matched:** 1
50S ribosomal protein L6 [Vibrio angustum S14]

Observed	Mr(expt)	Mr(calc)	ppm	Start	End	Miss	Ions	Peptide
1169.6591	1168.6518	1168.6564	-3.95	139 -	149	0	48	K.QLVGQVAADIR.A

No match to: 832.4830, 864.4371, 870.5446, 897.4156, 919.5370, 973.5306, 974.4789, 986.5797, 1004.5919, 1037.5306, 1060.5627, 1132.6766, 1143.5376, 1150.6438, 1152.6352, 1160.5670, 1193.6003, 1198.5902, 1230.5778, 1234.6676, 1261.6761, 1263.6787, 1277.6967, 1302.6902, 1307.6557, 1340.6607, 1357.6919, 1383.6631, 1390.6668, 1407.6965, 1434.7636, 1438.6220, 1458.6980, 1475.7261, 1487.7204, 1584.8657, 1638.8097, 1657.7630, 1687.9216, 1695.8492, 1699.7953, 1707.7412, 1838.8869, 1851.8805, 1888.8985, 1904.8884, 1940.8860, 2225.0763, 2239.0817, 2248.1493, 2252.0589, 2286.0247, 2399.0067, 2510.1289, 2705.2084

48. gi|117621078 **Mass:** 18568 **Score:** 53 **Expect:** 13 **Queries matched:** 1
50S ribosomal protein L6 [Aeromonas hydrophila subsp. hydrophila ATCC 7966]

Observed	Mr(expt)	Mr(calc)	ppm	Start	End	Miss	Ions	Peptide
1169.6591	1168.6518	1168.6564	-3.95	139 -	149	0	48	K.QLVGQVAADIR.A

No match to: 832.4830, 864.4371, 870.5446, 897.4156, 919.5370, 973.5306, 974.4789, 986.5797, 1004.5919, 1037.5306, 1060.5627, 1132.6766, 1143.5376, 1150.6438, 1152.6352,

1160.5670, 1193.6003, 1198.5902, 1230.5778, 1234.6676, 1261.6761, 1263.6787, 1277.6967, 1302.6902, 1307.6557, 1340.6607, 1357.6919, 1383.6631, 1390.6668, 1407.6965, 1434.7636, 1438.6220, 1458.6980, 1475.7261, 1487.7204, 1584.8657, 1638.8097, 1657.7630, 1687.9216, 1695.8492, 1699.7953, 1707.7412, 1838.8869, 1851.8805, 1888.8985, 1904.8884, 1940.8860, 2225.0763, 2239.0817, 2248.1493, 2252.0589, 2286.0247, 2399.0067, 2510.1289, 2705.2084

49. gi|145300920 **Mass:** 18556 **Score:** 53 **Expect:** 13 **Queries matched:** 1

ribosomal protein L6 [Aeromonas salmonicida subsp. salmonicida A449]

Observed	Mr(expt)	Mr(calc)	ppm	Start	End	Miss	Ions	Peptide
1169.6591	1168.6518	1168.6564	-3.95	139 -	149	0	48	K.QLVGQVAADIR.S

No match to: 832.4830, 864.4371, 870.5446, 897.4156, 919.5370, 973.5306, 974.4789, 986.5797, 1004.5919, 1037.5306, 1060.5627, 1132.6766, 1143.5376, 1150.6438, 1152.6352, 1160.5670, 1193.6003, 1198.5902, 1230.5778, 1234.6676, 1261.6761, 1263.6787, 1277.6967, 1302.6902, 1307.6557, 1340.6607, 1357.6919, 1383.6631, 1390.6668, 1407.6965, 1434.7636, 1438.6220, 1458.6980, 1475.7261, 1487.7204, 1584.8657, 1638.8097, 1657.7630, 1687.9216, 1695.8492, 1699.7953, 1707.7412, 1838.8869, 1851.8805, 1888.8985, 1904.8884, 1940.8860, 2225.0763, 2239.0817, 2248.1493, 2252.0589, 2286.0247, 2399.0067, 2510.1289, 2705.2084

50. gi|51894195 **Mass:** 19030 **Score:** 53 **Expect:** 13 **Queries matched:** 1

50S ribosomal protein L6 [Symbiobacterium thermophilum IAM 14863]

Observed	Mr(expt)	Mr(calc)	ppm	Start	End	Miss	Ions	Peptide
1169.6591	1168.6518	1168.6564	-3.95	139 -	149	0	48	K.QLVGQVAADIR.A

No match to: 832.4830, 864.4371, 870.5446, 897.4156, 919.5370, 973.5306, 974.4789, 986.5797, 1004.5919, 1037.5306, 1060.5627, 1132.6766, 1143.5376, 1150.6438, 1152.6352, 1160.5670, 1193.6003, 1198.5902, 1230.5778, 1234.6676, 1261.6761, 1263.6787, 1277.6967, 1302.6902, 1307.6557, 1340.6607, 1357.6919, 1383.6631, 1390.6668, 1407.6965, 1434.7636, 1438.6220, 1458.6980, 1475.7261, 1487.7204, 1584.8657, 1638.8097, 1657.7630, 1687.9216, 1695.8492, 1699.7953, 1707.7412, 1838.8869, 1851.8805, 1888.8985, 1904.8884, 1940.8860, 2225.0763, 2239.0817, 2248.1493, 2252.0589, 2286.0247, 2399.0067, 2510.1289, 2705.2084

Search Parameters

Type of search : **MS/MS Ion Search**
Enzyme : **Trypsin**

```
Fixed modifications    : Carbamidomethyl (C)
Variable modifications : Oxidation (M)
Mass values            : Monoisotopic
Protein Mass           : Unrestricted
Peptide Mass Tolerance : ± 50 ppm
Fragment Mass Tolerance: ± 0.5 Da
Max Missed Cleavages   : 1
Instrument type        : MALDI-TOF-TOF
Query1 (832.4830,1+)   : <no title>
Query2 (864.4371,1+)   : <no title>
Query3 (870.5446,1+)   : <no title>
Query4 (897.4156,1+)   : <no title>
Query5 (919.5370,1+)   : <no title>
Query6 (973.5306,1+)   : <no title>
Query7 (974.4789,1+)   : <no title>
Query8 (986.5797,1+)   : <no title>
Query9 (1004.5919,1+)  : <no title>
Query10 (1037.5306,1+) : <no title>
Query11 (1060.5627,1+) : <no title>
Query12 (1132.6766,1+) : <no title>
Query13 (1143.5376,1+) : <no title>
Query14 (1150.6438,1+) : <no title>
Query15 (1152.6352,1+) : <no title>
Query16 (1160.5670,1+) : <no title>
Query17 (1169.6591,1+) : <no title>
Query18 (1193.6003,1+) : <no title>
Query19 (1198.5902,1+) : <no title>
Query20 (1230.5778,1+) : <no title>
Query21 (1234.6676,1+) : <no title>
Query22 (1261.6761,1+) : <no title>
Query23 (1263.6787,1+) : <no title>
Query24 (1277.6967,1+) : <no title>
Query25 (1302.6902,1+) : <no title>
Query26 (1307.6557,1+) : <no title>
Query27 (1340.6607,1+) : <no title>
Query28 (1357.6919,1+) : <no title>
Query29 (1383.6631,1+) : <no title>
```

```
Query30 (1390.6668,1+) : <no title>
Query31 (1407.6965,1+) : <no title>
Query32 (1434.7636,1+) : <no title>
Query33 (1438.6220,1+) : <no title>
Query34 (1458.6980,1+) : <no title>
Query35 (1475.7261,1+) : <no title>
Query36 (1487.7204,1+) : <no title>
Query37 (1584.8657,1+) : <no title>
Query38 (1638.8097,1+) : <no title>
Query39 (1657.7630,1+) : <no title>
Query40 (1687.9216,1+) : <no title>
Query41 (1695.8492,1+) : <no title>
Query42 (1699.7953,1+) : <no title>
Query43 (1707.7412,1+) : <no title>
Query44 (1838.8869,1+) : <no title>
Query45 (1851.8805,1+) : <no title>
Query46 (1888.8985,1+) : <no title>
Query47 (1904.8884,1+) : <no title>
Query48 (1940.8860,1+) : <no title>
Query49 (2225.0763,1+) : <no title>
Query50 (2239.0817,1+) : <no title>
Query51 (2248.1493,1+) : <no title>
Query52 (2252.0589,1+) : <no title>
Query53 (2286.0247,1+) : <no title>
Query54 (2399.0067,1+) : <no title>
Query55 (2510.1289,1+) : <no title>
Query56 (2705.2084,1+) : <no title>
```

Mascot: http://www.matrixscience.com/
