# Supplementary material for: Proteomic analysis of Salmonella enterica serovar Enteritidis following propionate adaptation
Source: BMC Microbiol. 2010 Sep 28;10:249. doi: 10.1186/1471-2180-10-249 (PMC2957393; doi:10.1186/1471-2180-10-249)
Supplement: Additional file 3 — Protein Report A. Mass spectrometry report for SodA [file 1471-2180-10-249-S1.PDF]

Mascot Search Results

User	:	rliyana
Email	:	rliyana@uark.edu
Search title	:	
MS data file	:	DATA.TXT
Database	:	NCBInr 20071202 (5678482 sequences; 1961803296 residues)
Taxonomy	:	Bacteria (Eubacteria) (2746213 sequences)
Timestamp	:	21 Dec 2008 at 12:09:31 GMT
Warning	:	A Peptide summary report will usually give a much clearer picture of MS/MS se
Top Score	:	155 for gi|13399618, Chain A, Crystal Structure Analysis Of The H30a Mutant O

Probability Based Mowse Score

Protein score is -10*Log(P), where P is the probability that the observed match is a random event.
Protein scores greater than 77 are significant (p<0.05).
Protein scores are derived from ions scores as a non-probabilistic basis for ranking protein hits.

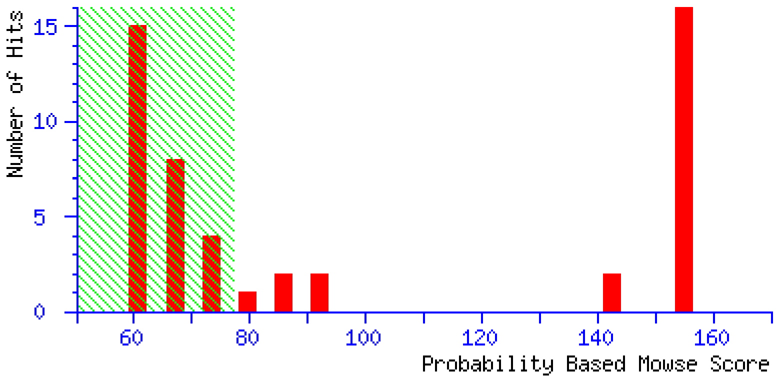

Protein Summary Report

Format As | Protein Summary (deprecated) Help

Significance threshold p< 0.05 Max. number of hits 50

Standard scoring ◉ MudPIT scoring ○ Ions score or expect cut-off 0 Show sub-sets 0

Show pop-ups ◉ Suppress pop-ups ○ Sort unassigned Decreasing Score Require bold red ☐

Overview Table

Click on column header to jump to entry in results list.
Move mouse over any indicator to highlight identical peptides.
Click on an indicator to see details of individual match.
Use check boxes to select sub-set of queries for new search.

Mouse over: -Query-

-Accession- -Sequence-

Hit:	1	2	3	4	5	6	7	8	9	10	11	12	13	14	15	16	17	18	19	20	21	22	23	24	25	26	27	28	29	30	31	32	33	34	35
☑ 807.3999 (1+)																																			
☑ 832.4835 (1+)																																			
☑ 856.5285 (1+)																																			
☑ 870.5457 (1+)																																			
☑ 897.4207 (1+)																																			
☑ 943.5581 (1+)																																			
☑ 973.5402 (1+)																																			
☑ 1028.4847 (1+)	●	●	●	●	●	●	●	●	●	●	●	●	●	●	●	●	●	●	●				●												
☑ 1037.5513 (1+)																																			
☑ 1051.6991 (1+)																																			
☑ 1052.6447 (1+)																																			
☑ 1060.5854 (1+)	●	●	●	●	●	●	●	●	●	●	●	●	●	●	●	●					●	●	●		●										
☑ 1064.6072 (1+)																																			

☑ 1066.5148 (1+)																																			
☑ 1074.5430 (1+)																																			
☑ 1090.5585 (1+)																																			
☑ 1107.5630 (1+)																																			
☑ 1109.5211 (1+)																																			
☑ 1111.5941 (1+)																																			
☑ 1140.5769 (1+)																																			
☑ 1150.6550 (1+)																																			
☑ 1157.6067 (1+)																																			
☑ 1184.6118 (1+)																																			
☑ 1193.6299 (1+)																															●			
☑ 1201.6527 (1+)																																			
☑ 1234.6902 (1+)																																			
☑ 1262.6852 (1+)																																			
☑ 1265.6542 (1+)																																			
☑ 1277.7111 (1+)																																			
☑ 1307.6798 (1+)																																			
☑ 1308.6631 (1+)																																			
☑ 1314.7742 (1+)																			●																
☑ 1320.5936 (1+)																															●			
☑ 1323.6793 (1+)																																			
☑ 1329.6530 (1+)																																			
☑ 1338.6701 (1+)																																			
☑ 1340.6933 (1+)																																			
☑ 1357.7238 (1+)																																			
☑ 1365.6565 (1+)																																			
☑ 1373.6674 (1+)																																			

☑ 1383.6947 (1+)																																			
☑ 1390.6914 (1+)																																			
☑ 1393.7380 (1+)																																			
☑ 1427.8365 (1+)																																			
☑ 1434.7666 (1+)																																			
☑ 1458.7253 (1+)																																			
☑ 1475.7605 (1+)																																			
☑ 1487.7463 (1+)																																			
☑ 1493.7394 (1+)																				●											●				
☑ 1566.7300 (1+)																																			
☑ 1586.7234 (1+)																																			
☑ 1618.7046 (1+)																																			
☑ 1632.7336 (1+)																			●																
☑ 1657.7836 (1+)																												●			●			●	
☑ 1688.7715 (1+)																																			
☑ 1699.8070 (1+)																																			
☑ 1706.7773 (1+)	●	●	●	●	●	●	●	●	●	●	●	●	●	●	●	●	●	●	●		●	●		●			●	●							
☑ 1708.7559 (1+)																																			
☑ 1722.7759 (1+)																																			
☑ 1738.7724 (1+)																																			
☑ 1754.7672 (1+)																												●				●			
☑ 1770.7611 (1+)																												●							
☑ 1817.9139 (1+)																																			
☑ 1838.8982 (1+)																																			
☑ 1851.8839 (1+)																																			
☑ 1940.8981 (1+)																																			
☑ 2225.0635 (1+)																																			

☑ 2286.0353 (1+)																																									
☑ 2383.9128 (1+)																																									
☑ 2398.9758 (1+)																																									
☑ 2705.1300 (1+)																																									
☑ 2717.0471 (1+)																																									
☑ 3312.3408 (1+)																																									

Select All | Select None | Search Selected

Index

	Accession	Mass	Score	Description
1.	gi|13399618	22886	155	Chain A, Crystal Structure Analysis Of The H30a Mutant Of Mang
2.	gi|13399622	22936	155	Chain A, Crystal Structure Of The E. Coli Manganese Superoxide
3.	gi|14719520	22961	155	Chain A, Crystal Structure Analysis Of The E. Coli Manganese S
4.	gi|3212626	22952	155	Chain A, Manganese Superoxide Dismutase From Escherichia Coli
5.	gi|14719528	22937	155	Chain A, Crystal Structure Analysis Of The E. Coli Manganese S
6.	gi|14719524	22936	155	Chain A, Crystal Structure Analysis Of The E. Coli Manganese S
7.	gi|15804498	23065	155	superoxide dismutase, manganese [Escherichia coli O157:H7 EDL9
8.	gi|75240302	23107	155	COG0605: Superoxide dismutase [Escherichia coli F11]
9.	gi|16767321	23065	155	superoxide dismutase [Salmonella typhimurium LT2]
10.	gi|16762379	23093	155	manganese superoxide dismutase [Salmonella enterica subsp. ent
11.	gi|62182516	23035	155	superoxide dismutase, manganese [Salmonella enterica subsp. en
12.	gi|82778924	23079	155	superoxide dismutase, manganese [Shigella dysenteriae Sd197]
13.	gi|49176442	23083	155	superoxide dismutase, Mn [Escherichia coli K12]
14.	gi|26250673	23352	155	Superoxide dismutase [Mn] [Escherichia coli CFT073]
15.	gi|38704210	23310	155	SodA [Escherichia coli O157:H7 str. Sakai]
16.	gi|91213448	23295	155	Mn superoxide dismutase [Escherichia coli UTI89]
17.	gi|808038	22952	144	Mn-superoxide dismutase
18.	gi|157147301	23608	143	hypothetical protein CKO_03093 [Citrobacter koseri ATCC BAA-89
19.	gi|150003719	153958	92	two-component system sensor histidine kinase/response regulato
20.	gi|57867258	36500	91	catabolite control protein A [Staphylococcus epidermidis RP62A
21.	gi|156935949	23785	86	hypothetical protein ESA_03843 [Enterobacter sakazakii ATCC BA

22.	gi|160866780	23685	85	hypothetical protein SARI_03588 [Salmonella enterica subsp. ar
23.	gi|110833822	35956	79	hypothetical protein ABO_0961 [Alcanivorax borkumensis SK2]
24.	gi|116006777	82342	76	TraI [Bordetella pertussis]
25.	gi|148240893	44158	75	Putative cAMP-induced filamentation protein [Bradyrhizobium sp
26.	gi|58039077	37927	75	D-alanyl-D-alanine carboxypeptidase [Gluconobacter oxydans 621
27.	gi|109649126	93680	72	ATP dependent DNA ligase [Desulfitobacterium hafniense DCB-2]
28.	gi|89076064	84568	68	hypothetical protein SKA34_16590 [Photobacterium sp. SKA34]
29.	gi|91778672	48306	66	Electron-transferring-flavoprotein dehydrogenase [Burkholderia
30.	gi|146282034	37412	66	TIM-barrel protein, yjbN family [Pseudomonas stutzeri A1501]
31.	gi|75675707	76662	65	DEAD/DEAH box helicase [Nitrobacter winogradskyi Nb-255]
32.	gi|149279903	89251	65	TonB-linked outer membrane receptor [Pedobacter sp. BAL39]
33.	gi|124010167	17818	64	hypothetical protein M23134_01768 [Microscilla marina ATCC 231
34.	gi|30263944	104494	64	prophage LambdaBa02, tape measure protein, putative [Bacillus
35.	gi|148244760	13648	64	hypothetical protein COSY_0617 [Candidatus Vesicomyosocius oku
36.	gi|146279657	56213	64	hypothetical protein Rsph17025_3642 [Rhodobacter sphaeroides A
37.	gi|15602483	99875	63	PepN [Pasteurella multocida subsp. multocida str. Pm70]
38.	gi|82749916	67077	63	RGD-containing lipoprotein [Staphylococcus aureus RF122]
39.	gi|89893362	93668	63	hypothetical protein DSY0616 [Desulfitobacterium hafniense Y51
40.	gi|109648252	24507	62	conserved hypothetical protein [Desulfitobacterium hafniense D
41.	gi|49482442	67044	62	RGD-containing lipoprotein [Staphylococcus aureus subsp. aureu
42.	gi|153167459	70979	62	hypothetical protein LMHG_01079 [Listeria monocytogenes FSL N1
43.	gi|150015592	16889	62	transcriptional regulator, MarR family [Clostridium beijerinck
44.	gi|154249526	42276	61	hypothetical protein Fnod_0841 [Fervidobacterium nodosum Rt17-
45.	gi|21672982	92806	61	DNA gyrase subunit A [Chlorobium tepidum TLS]
46.	gi|121527739	35386	61	putative integrase/recombinase protein [Ralstonia pickettii 12
47.	gi|26554375	87671	61	phenylalanyl-tRNA synthetase beta subunit [Mycoplasma penetran
48.	gi|138895630	9671	61	Molybdopterin biosynthesis MoeB-like protein [Geobacillus ther
49.	gi|118045195	52397	61	histidyl-tRNA synthetase [Chloroflexus aggregans DSM 9485]
50.	gi|88797880	40810	61	hypothetical protein MED297_12030 [Reinekea sp. MED297]

Results List

1. gi|13399618 **Mass:** 22886 **Score:** 155 **Expect:** 8.7e-010 **Queries matched:** 5

Chain A, Crystal Structure Analysis Of The H30a Mutant Of Manganese Superoxide Dismutase

Observed	Mr(expt)	Mr(calc)	ppm	Start	End	Miss	Ions	Peptide
1028.4847	1027.4774	1027.4611	15.9	105 -	113	0	52	R.DFGSVDNFK.A

1060.5854	1059.5781	1059.5924	-13.51	90 - 99	1	3	K.KGTTLQGDLK.A
1140.5769	1139.5697	1139.5393	26.6	21 - 29	0	---	K.QTMEIHHTK.A + Oxidation
1632.7336	1631.7263	1631.7468	-12.52	105 - 118	1	---	R.DFGSVDNFKAEFEK.A
1706.7773	1705.7700	1705.7849	-8.69	187 - 200	0	70	K.EFWNVVNWDEAAAR.F

No match to: 807.3999, 832.4835, 856.5285, 870.5457, 897.4207, 943.5581, 973.5402, 1037.5513, 1051.6991, 1052.6447, 1064.6072, 1066.5148, 1074.5430, 1090.5585, 1107.5630, 1109.5211, 1111.5941, 1150.6550, 1157.6067, 1184.6118, 1193.6299, 1201.6527, 1234.6902, 1262.6852, 1265.6542, 1277.7111, 1307.6798, 1308.6631, 1314.7742, 1320.5936, 1323.6793, 1329.6530, 1338.6701, 1340.6933, 1357.7238, 1365.6565, 1373.6674, 1383.6947, 1390.6914, 1393.7380, 1427.8365, 1434.7666, 1458.7253, 1475.7605, 1487.7463, 1493.7394, 1566.7300, 1586.7234, 1618.7046, 1657.7836, 1688.7715, 1699.8070, 1708.7559, 1722.7759, 1738.7724, 1754.7672, 1770.7611, 1817.9139, 1838.8982, 1851.8839, 1940.8981, 2225.0635, 2286.0353, 2383.9128, 2398.9758, 2705.1300, 2717.0471, 3312.3408

2. gi|13399622 **Mass:** 22936 **Score:** 155 **Expect:** 8.7e-010 **Queries matched:** 5

Chain A, Crystal Structure Of The E. Coli Manganese Superoxide Dismutase Mutant Y174f At

Observed	Mr(expt)	Mr(calc)	ppm	Start - End	Miss	Ions	Peptide
1028.4847	1027.4774	1027.4611	15.9	105 - 113	0	52	R.DFGSVDNFK.A
1060.5854	1059.5781	1059.5924	-13.51	90 - 99	1	3	K.KGTTLQGDLK.A
1140.5769	1139.5697	1139.5393	26.6	21 - 29	0	---	K.QTMEIHHTK.H + Oxidation
1632.7336	1631.7263	1631.7468	-12.52	105 - 118	1	---	R.DFGSVDNFKAEFEK.A
1706.7773	1705.7700	1705.7849	-8.69	187 - 200	0	70	K.EFWNVVNWDEAAAR.F

No match to: 807.3999, 832.4835, 856.5285, 870.5457, 897.4207, 943.5581, 973.5402, 1037.5513, 1051.6991, 1052.6447, 1064.6072, 1066.5148, 1074.5430, 1090.5585, 1107.5630, 1109.5211, 1111.5941, 1150.6550, 1157.6067, 1184.6118, 1193.6299, 1201.6527, 1234.6902, 1262.6852, 1265.6542, 1277.7111, 1307.6798, 1308.6631, 1314.7742, 1320.5936, 1323.6793, 1329.6530, 1338.6701, 1340.6933, 1357.7238, 1365.6565, 1373.6674, 1383.6947, 1390.6914, 1393.7380, 1427.8365, 1434.7666, 1458.7253, 1475.7605, 1487.7463, 1493.7394, 1566.7300, 1586.7234, 1618.7046, 1657.7836, 1688.7715, 1699.8070, 1708.7559, 1722.7759, 1738.7724, 1754.7672, 1770.7611, 1817.9139, 1838.8982, 1851.8839, 1940.8981, 2225.0635, 2286.0353, 2383.9128, 2398.9758, 2705.1300, 2717.0471, 3312.3408

3. gi|14719520 **Mass:** 22961 **Score:** 155 **Expect:** 8.7e-010 **Queries matched:** 5

Chain A, Crystal Structure Analysis Of The E. Coli Manganese Superoxide Dismutase Q146h M

Observed	Mr(expt)	Mr(calc)	ppm	Start	End	Miss	Ions	Peptide
1028.4847	1027.4774	1027.4611	15.9	105 -	113	0	52	R.DFGSVDNFK.A
1060.5854	1059.5781	1059.5924	-13.51	90 -	99	1	3	K.KGTTLQGDLK.A
1140.5769	1139.5697	1139.5393	26.6	21 -	29	0	---	K.QTMEIHHTK.H + Oxidation
1632.7336	1631.7263	1631.7468	-12.52	105 -	118	1	---	R.DFGSVDNFKAEFEK.A
1706.7773	1705.7700	1705.7849	-8.69	187 -	200	0	70	K.EFWNVVNWDEAAAR.F

No match to: 807.3999, 832.4835, 856.5285, 870.5457, 897.4207, 943.5581, 973.5402, 1037.5513, 1051.6991, 1052.6447, 1064.6072, 1066.5148, 1074.5430, 1090.5585, 1107.5630, 1109.5211, 1111.5941, 1150.6550, 1157.6067, 1184.6118, 1193.6299, 1201.6527, 1234.6902, 1262.6852, 1265.6542, 1277.7111, 1307.6798, 1308.6631, 1314.7742, 1320.5936, 1323.6793, 1329.6530, 1338.6701, 1340.6933, 1357.7238, 1365.6565, 1373.6674, 1383.6947, 1390.6914, 1393.7380, 1427.8365, 1434.7666, 1458.7253, 1475.7605, 1487.7463, 1493.7394, 1566.7300, 1586.7234, 1618.7046, 1657.7836, 1688.7715, 1699.8070, 1708.7559, 1722.7759, 1738.7724, 1754.7672, 1770.7611, 1817.9139, 1838.8982, 1851.8839, 1940.8981, 2225.0635, 2286.0353, 2383.9128, 2398.9758, 2705.1300, 2717.0471, 3312.3408

4. gi|3212626 **Mass:** 22952 **Score:** 155 **Expect:** 8.7e-010 **Queries matched:** 5

Chain A, Manganese Superoxide Dismutase From Escherichia Coli

Observed	Mr(expt)	Mr(calc)	ppm	Start	End	Miss	Ions	Peptide
1028.4847	1027.4774	1027.4611	15.9	105 -	113	0	52	R.DFGSVDNFK.A
1060.5854	1059.5781	1059.5924	-13.51	90 -	99	1	3	K.KGTTLQGDLK.A
1140.5769	1139.5697	1139.5393	26.6	21 -	29	0	---	K.QTMEIHHTK.H + Oxidation
1632.7336	1631.7263	1631.7468	-12.52	105 -	118	1	---	R.DFGSVDNFKAEFEK.A
1706.7773	1705.7700	1705.7849	-8.69	187 -	200	0	70	K.EFWNVVNWDEAAAR.F

No match to: 807.3999, 832.4835, 856.5285, 870.5457, 897.4207, 943.5581, 973.5402, 1037.5513, 1051.6991, 1052.6447, 1064.6072, 1066.5148, 1074.5430, 1090.5585, 1107.5630, 1109.5211, 1111.5941, 1150.6550, 1157.6067, 1184.6118, 1193.6299, 1201.6527, 1234.6902, 1262.6852, 1265.6542, 1277.7111, 1307.6798, 1308.6631, 1314.7742, 1320.5936, 1323.6793, 1329.6530, 1338.6701, 1340.6933, 1357.7238, 1365.6565, 1373.6674, 1383.6947, 1390.6914, 1393.7380, 1427.8365, 1434.7666, 1458.7253, 1475.7605, 1487.7463, 1493.7394, 1566.7300, 1586.7234, 1618.7046, 1657.7836, 1688.7715, 1699.8070, 1708.7559, 1722.7759, 1738.7724, 1754.7672, 1770.7611, 1817.9139, 1838.8982, 1851.8839, 1940.8981, 2225.0635, 2286.0353, 2383.9128, 2398.9758, 2705.1300, 2717.0471, 3312.3408

5. gi|14719528 **Mass:** 22937 **Score:** 155 **Expect:** 8.7e-010 **Queries matched:** 5
Chain A, Crystal Structure Analysis Of The E. Coli Manganese Superoxide Dismutase Q146l M

Observed	Mr(expt)	Mr(calc)	ppm	Start	End	Miss	Ions	Peptide
1028.4847	1027.4774	1027.4611	15.9	105 -	113	0	52	R.DFGSVDNFK.A
1060.5854	1059.5781	1059.5924	-13.51	90 -	99	1	3	K.KGTTLQGDLK.A
1140.5769	1139.5697	1139.5393	26.6	21 -	29	0	---	K.QTMEIHHTK.H + Oxidation
1632.7336	1631.7263	1631.7468	-12.52	105 -	118	1	---	R.DFGSVDNFKAEFEK.A
1706.7773	1705.7700	1705.7849	-8.69	187 -	200	0	70	K.EFWNVVNWDEAAAR.F

No match to: 807.3999, 832.4835, 856.5285, 870.5457, 897.4207, 943.5581, 973.5402, 1037.5513, 1051.6991, 1052.6447, 1064.6072, 1066.5148, 1074.5430, 1090.5585, 1107.5630, 1109.5211, 1111.5941, 1150.6550, 1157.6067, 1184.6118, 1193.6299, 1201.6527, 1234.6902, 1262.6852, 1265.6542, 1277.7111, 1307.6798, 1308.6631, 1314.7742, 1320.5936, 1323.6793, 1329.6530, 1338.6701, 1340.6933, 1357.7238, 1365.6565, 1373.6674, 1383.6947, 1390.6914, 1393.7380, 1427.8365, 1434.7666, 1458.7253, 1475.7605, 1487.7463, 1493.7394, 1566.7300, 1586.7234, 1618.7046, 1657.7836, 1688.7715, 1699.8070, 1708.7559, 1722.7759, 1738.7724, 1754.7672, 1770.7611, 1817.9139, 1838.8982, 1851.8839, 1940.8981, 2225.0635, 2286.0353, 2383.9128, 2398.9758, 2705.1300, 2717.0471, 3312.3408

6. gi|14719524 **Mass:** 22936 **Score:** 155 **Expect:** 8.7e-010 **Queries matched:** 5
Chain A, Crystal Structure Analysis Of The E. Coli Manganese Superoxide Dismutase Y34f Mu

Observed	Mr(expt)	Mr(calc)	ppm	Start	End	Miss	Ions	Peptide
1028.4847	1027.4774	1027.4611	15.9	105 -	113	0	52	R.DFGSVDNFK.A
1060.5854	1059.5781	1059.5924	-13.51	90 -	99	1	3	K.KGTTLQGDLK.A
1140.5769	1139.5697	1139.5393	26.6	21 -	29	0	---	K.QTMEIHHTK.H + Oxidation
1632.7336	1631.7263	1631.7468	-12.52	105 -	118	1	---	R.DFGSVDNFKAEFEK.A
1706.7773	1705.7700	1705.7849	-8.69	187 -	200	0	70	K.EFWNVVNWDEAAAR.F

No match to: 807.3999, 832.4835, 856.5285, 870.5457, 897.4207, 943.5581, 973.5402, 1037.5513, 1051.6991, 1052.6447, 1064.6072, 1066.5148, 1074.5430, 1090.5585, 1107.5630, 1109.5211, 1111.5941, 1150.6550, 1157.6067, 1184.6118, 1193.6299, 1201.6527, 1234.6902, 1262.6852, 1265.6542, 1277.7111, 1307.6798, 1308.6631, 1314.7742, 1320.5936, 1323.6793, 1329.6530, 1338.6701, 1340.6933, 1357.7238, 1365.6565, 1373.6674, 1383.6947, 1390.6914, 1393.7380, 1427.8365, 1434.7666, 1458.7253, 1475.7605, 1487.7463, 1493.7394, 1566.7300, 1586.7234, 1618.7046, 1657.7836, 1688.7715, 1699.8070, 1708.7559, 1722.7759, 1738.7724, 1754.7672, 1770.7611, 1817.9139, 1838.8982, 1851.8839, 1940.8981, 2225.0635, 2286.0353,

2383.9128, 2398.9758, 2705.1300, 2717.0471, 3312.3408

7. gi|15804498 **Mass:** 23065 **Score:** 155 **Expect:** 8.7e-010 **Queries matched:** 5
superoxide dismutase, manganese [Escherichia coli O157:H7 EDL933]

Observed	Mr(expt)	Mr(calc)	ppm	Start	End	Miss	Ions	Peptide
1028.4847	1027.4774	1027.4611	15.9	106 -	114	0	52	R.DFGSVDNFK.A
1060.5854	1059.5781	1059.5924	-13.51	91 -	100	1	3	K.KGTTLQGDLK.A
1140.5769	1139.5697	1139.5393	26.6	22 -	30	0	---	K.QTMEIHHTK.H + Oxidation
1632.7336	1631.7263	1631.7468	-12.52	106 -	119	1	---	R.DFGSVDNFKAEFEK.A
1706.7773	1705.7700	1705.7849	-8.69	188 -	201	0	70	K.EFWNVVNWDEAAAR.F

No match to: 807.3999, 832.4835, 856.5285, 870.5457, 897.4207, 943.5581, 973.5402, 1037.5513, 1051.6991, 1052.6447, 1064.6072, 1066.5148, 1074.5430, 1090.5585, 1107.5630, 1109.5211, 1111.5941, 1150.6550, 1157.6067, 1184.6118, 1193.6299, 1201.6527, 1234.6902, 1262.6852, 1265.6542, 1277.7111, 1307.6798, 1308.6631, 1314.7742, 1320.5936, 1323.6793, 1329.6530, 1338.6701, 1340.6933, 1357.7238, 1365.6565, 1373.6674, 1383.6947, 1390.6914, 1393.7380, 1427.8365, 1434.7666, 1458.7253, 1475.7605, 1487.7463, 1493.7394, 1566.7300, 1586.7234, 1618.7046, 1657.7836, 1688.7715, 1699.8070, 1708.7559, 1722.7759, 1738.7724, 1754.7672, 1770.7611, 1817.9139, 1838.8982, 1851.8839, 1940.8981, 2225.0635, 2286.0353, 2383.9128, 2398.9758, 2705.1300, 2717.0471, 3312.3408

8. gi|75240302 **Mass:** 23107 **Score:** 155 **Expect:** 8.7e-010 **Queries matched:** 5
COG0605: Superoxide dismutase [Escherichia coli F11]

Observed	Mr(expt)	Mr(calc)	ppm	Start	End	Miss	Ions	Peptide
1028.4847	1027.4774	1027.4611	15.9	106 -	114	0	52	R.DFGSVDNFK.A
1060.5854	1059.5781	1059.5924	-13.51	91 -	100	1	3	K.KGTTLQGDLK.A
1140.5769	1139.5697	1139.5393	26.6	22 -	30	0	---	K.QTMEIHHTK.H + Oxidation
1632.7336	1631.7263	1631.7468	-12.52	106 -	119	1	---	R.DFGSVDNFKAEFEK.A
1706.7773	1705.7700	1705.7849	-8.69	188 -	201	0	70	K.EFWNVVNWDEAAAR.F

No match to: 807.3999, 832.4835, 856.5285, 870.5457, 897.4207, 943.5581, 973.5402, 1037.5513, 1051.6991, 1052.6447, 1064.6072, 1066.5148, 1074.5430, 1090.5585, 1107.5630, 1109.5211, 1111.5941, 1150.6550, 1157.6067, 1184.6118, 1193.6299, 1201.6527, 1234.6902, 1262.6852, 1265.6542, 1277.7111, 1307.6798, 1308.6631, 1314.7742, 1320.5936, 1323.6793, 1329.6530, 1338.6701, 1340.6933, 1357.7238, 1365.6565, 1373.6674, 1383.6947, 1390.6914, 1393.7380, 1427.8365, 1434.7666, 1458.7253, 1475.7605, 1487.7463, 1493.7394, 1566.7300,

1586.7234, 1618.7046, 1657.7836, 1688.7715, 1699.8070, 1708.7559, 1722.7759, 1738.7724, 1754.7672, 1770.7611, 1817.9139, 1838.8982, 1851.8839, 1940.8981, 2225.0635, 2286.0353, 2383.9128, 2398.9758, 2705.1300, 2717.0471, 3312.3408

9. gi|16767321 **Mass:** 23065 **Score:** 155 **Expect:** 8.7e-010 **Queries matched:** 5
superoxide dismutase [Salmonella typhimurium LT2]

Observed	Mr(expt)	Mr(calc)	ppm	Start	End	Miss	Ions	Peptide
1028.4847	1027.4774	1027.4611	15.9	106 -	114	0	52	R.DFGSVDNFK.A
1060.5854	1059.5781	1059.5924	-13.51	91 -	100	1	3	K.KGTTLQGDLK.A
1140.5769	1139.5697	1139.5393	26.6	22 -	30	0	---	K.QTMEIHHTK.H + Oxidation
1632.7336	1631.7263	1631.7468	-12.52	106 -	119	1	---	R.DFGSVDNFKAEFEK.A
1706.7773	1705.7700	1705.7849	-8.69	188 -	201	0	70	K.EFWNVVNWDEAAAR.F

No match to: 807.3999, 832.4835, 856.5285, 870.5457, 897.4207, 943.5581, 973.5402, 1037.5513, 1051.6991, 1052.6447, 1064.6072, 1066.5148, 1074.5430, 1090.5585, 1107.5630, 1109.5211, 1111.5941, 1150.6550, 1157.6067, 1184.6118, 1193.6299, 1201.6527, 1234.6902, 1262.6852, 1265.6542, 1277.7111, 1307.6798, 1308.6631, 1314.7742, 1320.5936, 1323.6793, 1329.6530, 1338.6701, 1340.6933, 1357.7238, 1365.6565, 1373.6674, 1383.6947, 1390.6914, 1393.7380, 1427.8365, 1434.7666, 1458.7253, 1475.7605, 1487.7463, 1493.7394, 1566.7300, 1586.7234, 1618.7046, 1657.7836, 1688.7715, 1699.8070, 1708.7559, 1722.7759, 1738.7724, 1754.7672, 1770.7611, 1817.9139, 1838.8982, 1851.8839, 1940.8981, 2225.0635, 2286.0353, 2383.9128, 2398.9758, 2705.1300, 2717.0471, 3312.3408

10. gi|16762379 **Mass:** 23093 **Score:** 155 **Expect:** 8.7e-010 **Queries matched:** 5
manganese superoxide dismutase [Salmonella enterica subsp. enterica serovar Typhi str. CT

Observed	Mr(expt)	Mr(calc)	ppm	Start	End	Miss	Ions	Peptide
1028.4847	1027.4774	1027.4611	15.9	106 -	114	0	52	R.DFGSVDNFK.A
1060.5854	1059.5781	1059.5924	-13.51	91 -	100	1	3	K.KGTTLQGDLK.A
1140.5769	1139.5697	1139.5393	26.6	22 -	30	0	---	K.QTMEIHHTK.H + Oxidation
1632.7336	1631.7263	1631.7468	-12.52	106 -	119	1	---	R.DFGSVDNFKAEFEK.A
1706.7773	1705.7700	1705.7849	-8.69	188 -	201	0	70	K.EFWNVVNWDEAAAR.F

No match to: 807.3999, 832.4835, 856.5285, 870.5457, 897.4207, 943.5581, 973.5402, 1037.5513, 1051.6991, 1052.6447, 1064.6072, 1066.5148, 1074.5430, 1090.5585, 1107.5630, 1109.5211, 1111.5941, 1150.6550, 1157.6067, 1184.6118, 1193.6299, 1201.6527, 1234.6902, 1262.6852, 1265.6542, 1277.7111, 1307.6798, 1308.6631, 1314.7742, 1320.5936, 1323.6793,

1329.6530, 1338.6701, 1340.6933, 1357.7238, 1365.6565, 1373.6674, 1383.6947, 1390.6914, 1393.7380, 1427.8365, 1434.7666, 1458.7253, 1475.7605, 1487.7463, 1493.7394, 1566.7300, 1586.7234, 1618.7046, 1657.7836, 1688.7715, 1699.8070, 1708.7559, 1722.7759, 1738.7724, 1754.7672, 1770.7611, 1817.9139, 1838.8982, 1851.8839, 1940.8981, 2225.0635, 2286.0353, 2383.9128, 2398.9758, 2705.1300, 2717.0471, 3312.3408

11. gi|62182516 **Mass:** 23035 **Score:** 155 **Expect:** 8.7e-010 **Queries matched:** 5
superoxide dismutase, manganese [Salmonella enterica subsp. enterica serovar Choleraesuis

Observed	Mr(expt)	Mr(calc)	ppm	Start		End	Miss	Ions	Peptide
1028.4847	1027.4774	1027.4611	15.9	106	-	114	0	52	R.DFGSVDNFK.A
1060.5854	1059.5781	1059.5924	-13.51	91	-	100	1	3	K.KGTTLQGDLK.A
1066.5148	1065.5075	1065.5389	-29.48	22	-	30	0	---	K.QTMAIHHTK.H
1632.7336	1631.7263	1631.7468	-12.52	106	-	119	1	---	R.DFGSVDNFKAEFEK.A
1706.7773	1705.7700	1705.7849	-8.69	188	-	201	0	70	K.EFWNVVNWDEAAAR.F

No match to: 807.3999, 832.4835, 856.5285, 870.5457, 897.4207, 943.5581, 973.5402, 1037.5513, 1051.6991, 1052.6447, 1064.6072, 1074.5430, 1090.5585, 1107.5630, 1109.5211, 1111.5941, 1140.5769, 1150.6550, 1157.6067, 1184.6118, 1193.6299, 1201.6527, 1234.6902, 1262.6852, 1265.6542, 1277.7111, 1307.6798, 1308.6631, 1314.7742, 1320.5936, 1323.6793, 1329.6530, 1338.6701, 1340.6933, 1357.7238, 1365.6565, 1373.6674, 1383.6947, 1390.6914, 1393.7380, 1427.8365, 1434.7666, 1458.7253, 1475.7605, 1487.7463, 1493.7394, 1566.7300, 1586.7234, 1618.7046, 1657.7836, 1688.7715, 1699.8070, 1708.7559, 1722.7759, 1738.7724, 1754.7672, 1770.7611, 1817.9139, 1838.8982, 1851.8839, 1940.8981, 2225.0635, 2286.0353, 2383.9128, 2398.9758, 2705.1300, 2717.0471, 3312.3408

12. gi|82778924 **Mass:** 23079 **Score:** 155 **Expect:** 8.7e-010 **Queries matched:** 5
superoxide dismutase, manganese [Shigella dysenteriae Sd197]

Observed	Mr(expt)	Mr(calc)	ppm	Start		End	Miss	Ions	Peptide
1028.4847	1027.4774	1027.4611	15.9	106	-	114	0	52	R.DFGSVDNFK.A
1060.5854	1059.5781	1059.5924	-13.51	91	-	100	1	3	K.KGTTLQGDLK.A
1140.5769	1139.5697	1139.5393	26.6	22	-	30	0	---	K.QTMEIHHTK.H + Oxidation
1632.7336	1631.7263	1631.7468	-12.52	106	-	119	1	---	R.DFGSVDNFKAEFEK.A
1706.7773	1705.7700	1705.7849	-8.69	188	-	201	0	70	K.EFWNVVNWDEAAAR.F

No match to: 807.3999, 832.4835, 856.5285, 870.5457, 897.4207, 943.5581, 973.5402, 1037.5513, 1051.6991, 1052.6447, 1064.6072, 1066.5148, 1074.5430, 1090.5585, 1107.5630,

1109.5211, 1111.5941, 1150.6550, 1157.6067, 1184.6118, 1193.6299, 1201.6527, 1234.6902, 1262.6852, 1265.6542, 1277.7111, 1307.6798, 1308.6631, 1314.7742, 1320.5936, 1323.6793, 1329.6530, 1338.6701, 1340.6933, 1357.7238, 1365.6565, 1373.6674, 1383.6947, 1390.6914, 1393.7380, 1427.8365, 1434.7666, 1458.7253, 1475.7605, 1487.7463, 1493.7394, 1566.7300, 1586.7234, 1618.7046, 1657.7836, 1688.7715, 1699.8070, 1708.7559, 1722.7759, 1738.7724, 1754.7672, 1770.7611, 1817.9139, 1838.8982, 1851.8839, 1940.8981, 2225.0635, 2286.0353, 2383.9128, 2398.9758, 2705.1300, 2717.0471, 3312.3408

13. gi|49176442 **Mass:** 23083 **Score:** 155 **Expect:** 8.7e-010 **Queries matched:** 5

superoxide dismutase, Mn [Escherichia coli K12]

Observed	Mr(expt)	Mr(calc)	ppm	Start	End	Miss	Ions	Peptide
1028.4847	1027.4774	1027.4611	15.9	106 -	114	0	52	R.DFGSVDNFK.A
1060.5854	1059.5781	1059.5924	-13.51	91 -	100	1	3	K.KGTTLQGDLK.A
1140.5769	1139.5697	1139.5393	26.6	22 -	30	0	---	K.QTMEIHHTK.H + Oxidation
1632.7336	1631.7263	1631.7468	-12.52	106 -	119	1	---	R.DFGSVDNFKAEFEK.A
1706.7773	1705.7700	1705.7849	-8.69	188 -	201	0	70	K.EFWNVVNWDEAAAR.F

No match to: 807.3999, 832.4835, 856.5285, 870.5457, 897.4207, 943.5581, 973.5402, 1037.5513, 1051.6991, 1052.6447, 1064.6072, 1066.5148, 1074.5430, 1090.5585, 1107.5630, 1109.5211, 1111.5941, 1150.6550, 1157.6067, 1184.6118, 1193.6299, 1201.6527, 1234.6902, 1262.6852, 1265.6542, 1277.7111, 1307.6798, 1308.6631, 1314.7742, 1320.5936, 1323.6793, 1329.6530, 1338.6701, 1340.6933, 1357.7238, 1365.6565, 1373.6674, 1383.6947, 1390.6914, 1393.7380, 1427.8365, 1434.7666, 1458.7253, 1475.7605, 1487.7463, 1493.7394, 1566.7300, 1586.7234, 1618.7046, 1657.7836, 1688.7715, 1699.8070, 1708.7559, 1722.7759, 1738.7724, 1754.7672, 1770.7611, 1817.9139, 1838.8982, 1851.8839, 1940.8981, 2225.0635, 2286.0353, 2383.9128, 2398.9758, 2705.1300, 2717.0471, 3312.3408

14. gi|26250673 **Mass:** 23352 **Score:** 155 **Expect:** 8.7e-010 **Queries matched:** 5

Superoxide dismutase [Mn] [Escherichia coli CFT073]

Observed	Mr(expt)	Mr(calc)	ppm	Start	End	Miss	Ions	Peptide
1028.4847	1027.4774	1027.4611	15.9	108 -	116	0	52	R.DFGSVDNFK.A
1060.5854	1059.5781	1059.5924	-13.51	93 -	102	1	3	K.KGTTLQGDLK.A
1140.5769	1139.5697	1139.5393	26.6	24 -	32	0	---	K.QTMEIHHTK.H + Oxidation
1632.7336	1631.7263	1631.7468	-12.52	108 -	121	1	---	R.DFGSVDNFKAEFEK.A
1706.7773	1705.7700	1705.7849	-8.69	190 -	203	0	70	K.EFWNVVNWDEAAAR.F

No match to: 807.3999, 832.4835, 856.5285, 870.5457, 897.4207, 943.5581, 973.5402, 1037.5513, 1051.6991, 1052.6447, 1064.6072, 1066.5148, 1074.5430, 1090.5585, 1107.5630, 1109.5211, 1111.5941, 1150.6550, 1157.6067, 1184.6118, 1193.6299, 1201.6527, 1234.6902, 1262.6852, 1265.6542, 1277.7111, 1307.6798, 1308.6631, 1314.7742, 1320.5936, 1323.6793, 1329.6530, 1338.6701, 1340.6933, 1357.7238, 1365.6565, 1373.6674, 1383.6947, 1390.6914, 1393.7380, 1427.8365, 1434.7666, 1458.7253, 1475.7605, 1487.7463, 1493.7394, 1566.7300, 1586.7234, 1618.7046, 1657.7836, 1688.7715, 1699.8070, 1708.7559, 1722.7759, 1738.7724, 1754.7672, 1770.7611, 1817.9139, 1838.8982, 1851.8839, 1940.8981, 2225.0635, 2286.0353, 2383.9128, 2398.9758, 2705.1300, 2717.0471, 3312.3408

15. gi|38704210 **Mass:** 23310 **Score:** 155 **Expect:** 8.7e-010 **Queries matched:** 5
SodA [Escherichia coli O157:H7 str. Sakai]

Observed	Mr(expt)	Mr(calc)	ppm	Start	End	Miss	Ions	Peptide
1028.4847	1027.4774	1027.4611	15.9	108 -	116	0	52	R.DFGSVDNFK.A
1060.5854	1059.5781	1059.5924	-13.51	93 -	102	1	3	K.KGTTLQGDLK.A
1140.5769	1139.5697	1139.5393	26.6	24 -	32	0	---	K.QTMEIHHTK.H + Oxidation
1632.7336	1631.7263	1631.7468	-12.52	108 -	121	1	---	R.DFGSVDNFKAEFEK.A
1706.7773	1705.7700	1705.7849	-8.69	190 -	203	0	70	K.EFWNVVNWDEAAAR.F

No match to: 807.3999, 832.4835, 856.5285, 870.5457, 897.4207, 943.5581, 973.5402, 1037.5513, 1051.6991, 1052.6447, 1064.6072, 1066.5148, 1074.5430, 1090.5585, 1107.5630, 1109.5211, 1111.5941, 1150.6550, 1157.6067, 1184.6118, 1193.6299, 1201.6527, 1234.6902, 1262.6852, 1265.6542, 1277.7111, 1307.6798, 1308.6631, 1314.7742, 1320.5936, 1323.6793, 1329.6530, 1338.6701, 1340.6933, 1357.7238, 1365.6565, 1373.6674, 1383.6947, 1390.6914, 1393.7380, 1427.8365, 1434.7666, 1458.7253, 1475.7605, 1487.7463, 1493.7394, 1566.7300, 1586.7234, 1618.7046, 1657.7836, 1688.7715, 1699.8070, 1708.7559, 1722.7759, 1738.7724, 1754.7672, 1770.7611, 1817.9139, 1838.8982, 1851.8839, 1940.8981, 2225.0635, 2286.0353, 2383.9128, 2398.9758, 2705.1300, 2717.0471, 3312.3408

16. gi|91213448 **Mass:** 23295 **Score:** 155 **Expect:** 8.7e-010 **Queries matched:** 5
Mn superoxide dismutase [Escherichia coli UTI89]

Observed	Mr(expt)	Mr(calc)	ppm	Start	End	Miss	Ions	Peptide
1028.4847	1027.4774	1027.4611	15.9	108 -	116	0	52	R.DFGSVDNFK.A
1060.5854	1059.5781	1059.5924	-13.51	93 -	102	1	3	K.KGTTLQGDLK.A
1140.5769	1139.5697	1139.5393	26.6	24 -	32	0	---	K.QTMEIHHTK.H + Oxidation

1632.7336	1631.7263	1631.7468	-12.52	108 - 121	1	---	R.DFGSVDNFKAEFEK.A
1706.7773	1705.7700	1705.7849	-8.69	190 - 203	0	70	K.EFWNVVNWDEAAAR.F

No match to: 807.3999, 832.4835, 856.5285, 870.5457, 897.4207, 943.5581, 973.5402, 1037.5513, 1051.6991, 1052.6447, 1064.6072, 1066.5148, 1074.5430, 1090.5585, 1107.5630, 1109.5211, 1111.5941, 1150.6550, 1157.6067, 1184.6118, 1193.6299, 1201.6527, 1234.6902, 1262.6852, 1265.6542, 1277.7111, 1307.6798, 1308.6631, 1314.7742, 1320.5936, 1323.6793, 1329.6530, 1338.6701, 1340.6933, 1357.7238, 1365.6565, 1373.6674, 1383.6947, 1390.6914, 1393.7380, 1427.8365, 1434.7666, 1458.7253, 1475.7605, 1487.7463, 1493.7394, 1566.7300, 1586.7234, 1618.7046, 1657.7836, 1688.7715, 1699.8070, 1708.7559, 1722.7759, 1738.7724, 1754.7672, 1770.7611, 1817.9139, 1838.8982, 1851.8839, 1940.8981, 2225.0635, 2286.0353, 2383.9128, 2398.9758, 2705.1300, 2717.0471, 3312.3408

17. gi|808038 **Mass:** 22952 **Score:** 144 **Expect:** 1.1e-008 **Queries matched:** 4
Mn-superoxide dismutase

Observed	Mr(expt)	Mr(calc)	ppm	Start - End	Miss	Ions	Peptide
1028.4847	1027.4774	1027.4611	15.9	106 - 114	0	52	R.DFGSVDNFK.A
1140.5769	1139.5697	1139.5393	26.6	22 - 30	0	---	K.QTMEIHHTK.H + Oxidation
1632.7336	1631.7263	1631.7468	-12.52	106 - 119	1	---	R.DFGSVDNFKAEFEK.A
1706.7773	1705.7700	1705.7849	-8.69	188 - 201	0	70	K.EFWNVVNWDEAAAR.F

No match to: 807.3999, 832.4835, 856.5285, 870.5457, 897.4207, 943.5581, 973.5402, 1037.5513, 1051.6991, 1052.6447, 1060.5854, 1064.6072, 1066.5148, 1074.5430, 1090.5585, 1107.5630, 1109.5211, 1111.5941, 1150.6550, 1157.6067, 1184.6118, 1193.6299, 1201.6527, 1234.6902, 1262.6852, 1265.6542, 1277.7111, 1307.6798, 1308.6631, 1314.7742, 1320.5936, 1323.6793, 1329.6530, 1338.6701, 1340.6933, 1357.7238, 1365.6565, 1373.6674, 1383.6947, 1390.6914, 1393.7380, 1427.8365, 1434.7666, 1458.7253, 1475.7605, 1487.7463, 1493.7394, 1566.7300, 1586.7234, 1618.7046, 1657.7836, 1688.7715, 1699.8070, 1708.7559, 1722.7759, 1738.7724, 1754.7672, 1770.7611, 1817.9139, 1838.8982, 1851.8839, 1940.8981, 2225.0635, 2286.0353, 2383.9128, 2398.9758, 2705.1300, 2717.0471, 3312.3408

18. gi|157147301 **Mass:** 23608 **Score:** 143 **Expect:** 1.4e-008 **Queries matched:** 4
hypothetical protein CKO_03093 [Citrobacter koseri ATCC BAA-895]

Observed	Mr(expt)	Mr(calc)	ppm	Start - End	Miss	Ions	Peptide
1028.4847	1027.4774	1027.4611	15.9	110 - 118	0	52	R.DFGSVDNFK.A
1140.5769	1139.5697	1139.5393	26.6	26 - 34	0	---	K.QTMEIHHTK.H + Oxidation

1632.7336	1631.7263	1631.7468	-12.52	110 - 123	1	---	R.DFGSVDNFKAEFEK.A
1706.7773	1705.7700	1705.7849	-8.69	192 - 205	0	70	K.EFWNVVNWDEAAAR.F

No match to: 807.3999, 832.4835, 856.5285, 870.5457, 897.4207, 943.5581, 973.5402, 1037.5513, 1051.6991, 1052.6447, 1060.5854, 1064.6072, 1066.5148, 1074.5430, 1090.5585, 1107.5630, 1109.5211, 1111.5941, 1150.6550, 1157.6067, 1184.6118, 1193.6299, 1201.6527, 1234.6902, 1262.6852, 1265.6542, 1277.7111, 1307.6798, 1308.6631, 1314.7742, 1320.5936, 1323.6793, 1329.6530, 1338.6701, 1340.6933, 1357.7238, 1365.6565, 1373.6674, 1383.6947, 1390.6914, 1393.7380, 1427.8365, 1434.7666, 1458.7253, 1475.7605, 1487.7463, 1493.7394, 1566.7300, 1586.7234, 1618.7046, 1657.7836, 1688.7715, 1699.8070, 1708.7559, 1722.7759, 1738.7724, 1754.7672, 1770.7611, 1817.9139, 1838.8982, 1851.8839, 1940.8981, 2225.0635, 2286.0353, 2383.9128, 2398.9758, 2705.1300, 2717.0471, 3312.3408

19. gi|150003719 **Mass:** 153958 **Score:** 92 **Expect:** 0.0017 **Queries matched:** 19

two-component system sensor histidine kinase/response regulator, hybrid [Bacteroides vulg

Observed	Mr(expt)	Mr(calc)	ppm	Start End	Miss	Ions	Peptide
943.5581	942.5508	942.4923	62.0	406 - 413	0	---	K.EGVWISPR.Y
1028.4847	1027.4774	1027.4314	44.8	1244 - 1252	0	13	R.LSGEMNMSK.S + 2 Oxidati
1052.6447	1051.6374	1051.5491	84.0	92 - 99	0	---	R.YVWIGTWK.G
1074.5430	1073.5357	1073.5618	-24.28	439 - 447	0	---	K.QIYNPGNIR.D
1184.6118	1183.6045	1183.6713	-56.45	404 - 413	1	---	K.LKEGVWISPR.Y
1201.6527	1200.6455	1200.5849	50.5	942 - 950	1	---	K.YTPEYKCIK.V
1234.6902	1233.6829	1233.5780	85.0	418 - 427	1	---	R.VMRMTHQGMK.I + Oxidatio
1314.7742	1313.7669	1313.6589	82.2	627 - 638	1	---	R.DRQGGLYVGGHR.G
1320.5936	1319.5864	1319.6908	-79.11	1261 - 1272	0	---	K.AMTGLTPLDFVR.N
1383.6947	1382.6874	1382.6752	8.87	708 - 719	1	---	K.VEMAYKLDGVDK.D + Oxidat
1390.6914	1389.6841	1389.7153	-22.48	406 - 417	1	---	K.EGVWISPRYGAR.V
1427.8365	1426.8292	1426.8184	7.61	111 - 122	1	---	K.IIPFPDTRLLDK.G
1475.7605	1474.7533	1474.7602	-4.73	107 - 118	1	---	K.QTCKIIPFPDTR.L
1632.7336	1631.7263	1631.7647	-23.52	1244 - 1257	1	6	R.LSGEMNMSKSTLYR.K + Oxid
1657.7836	1656.7763	1656.8334	-34.45	455 - 468	0	---	K.GCLWIFSQSGLYVK.R
1706.7773	1705.7700	1705.9250	-90.87	654 - 668	1	13	K.IQFSSVVTDVKIENK.S
1940.8981	1939.8908	1939.8622	14.7	1311 - 1326	0	---	K.EEFGMTPTEYQQSHIK.V + Ox
2717.0471	2716.0398	2716.2802	-88.52	1158 - 1182	1	---	K.NGIDDRIASYDAGADGYIAKPFE

3312.3408 3311.3335 3311.5744 -72.73 594 - 620 1 --- K.VLQYNIYQKVCMNYATSDGNIWV

No match to: 807.3999, 832.4835, 856.5285, 870.5457, 897.4207, 973.5402, 1037.5513, 1051.6991, 1060.5854, 1064.6072, 1066.5148, 1090.5585, 1107.5630, 1109.5211, 1111.5941, 1140.5769, 1150.6550, 1157.6067, 1193.6299, 1262.6852, 1265.6542, 1277.7111, 1307.6798, 1308.6631, 1323.6793, 1329.6530, 1338.6701, 1340.6933, 1357.7238, 1365.6565, 1373.6674, 1393.7380, 1434.7666, 1458.7253, 1487.7463, 1493.7394, 1566.7300, 1586.7234, 1618.7046, 1688.7715, 1699.8070, 1708.7559, 1722.7759, 1738.7724, 1754.7672, 1770.7611, 1817.9139, 1838.8982, 1851.8839, 2225.0635, 2286.0353, 2383.9128, 2398.9758, 2705.1300

20. gi|57867258 **Mass:** 36500 **Score:** 91 **Expect:** 0.0022 **Queries matched:** 12

catabolite control protein A [Staphylococcus epidermidis RP62A]

Observed	Mr(expt)	Mr(calc)	ppm	Start	End	Miss	Ions	Peptide
943.5581	942.5508	942.5498	1.03	35 -	42	1	---	R.NKVNEVIK.R
1037.5513	1036.5441	1036.5553	-10.85	2 -	10	0	---	M.TVTIYDVAR.E
1066.5148	1065.5075	1065.5931	-80.28	201 -	209	0	---	K.NVLSQHQLK.L
1140.5769	1139.5697	1139.5532	14.4	81 -	90	0	---	R.GLEDIATMYK.Y
1184.6118	1183.6045	1183.5907	11.7	1 -	10	0	---	-.MTVTIYDVAR.E + Oxidation
1314.7742	1313.7669	1313.6867	61.1	179 -	190	0	1	K.TFSLIGGEYSIK.A
1329.6530	1328.6458	1328.7425	-72.80	43 -	53	1	---	K.RLNYRPNAVAR.G
1373.6674	1372.6601	1372.6623	-1.60	151 -	162	0	---	K.DDHIASVNIDFK.Q
1393.7380	1392.7307	1392.7361	-3.89	2 -	13	1	---	M.TVTIYDVAREAR.V
1493.7394	1492.7322	1492.7409	-5.85	163 -	175	0	4	K.QAAEEATQYLIEK.G
1618.7046	1617.6974	1617.7271	-18.35	91 -	104	0	---	K.YHSIISNSDNDPEK.E
2286.0353	2285.0280	2285.1910	-71.32	14 -	34	1	---	R.VSMATVSRVVNGNQNVKPETR.N

No match to: 807.3999, 832.4835, 856.5285, 870.5457, 897.4207, 973.5402, 1028.4847, 1051.6991, 1052.6447, 1060.5854, 1064.6072, 1074.5430, 1090.5585, 1107.5630, 1109.5211, 1111.5941, 1150.6550, 1157.6067, 1193.6299, 1201.6527, 1234.6902, 1262.6852, 1265.6542, 1277.7111, 1307.6798, 1308.6631, 1320.5936, 1323.6793, 1338.6701, 1340.6933, 1357.7238, 1365.6565, 1383.6947, 1390.6914, 1427.8365, 1434.7666, 1458.7253, 1475.7605, 1487.7463, 1566.7300, 1586.7234, 1632.7336, 1657.7836, 1688.7715, 1699.8070, 1706.7773, 1708.7559, 1722.7759, 1738.7724, 1754.7672, 1770.7611, 1817.9139, 1838.8982, 1851.8839, 1940.8981, 2225.0635, 2383.9128, 2398.9758, 2705.1300, 2717.0471, 3312.3408

21. gi|156935949 **Mass:** 23785 **Score:** 86 **Expect:** 0.0071 **Queries matched:** 3

hypothetical protein ESA_03843 [Enterobacter sakazakii ATCC BAA-894]

Observed	Mr(expt)	Mr(calc)	ppm	Start		End	Miss	Ions	Peptide
1060.5854	1059.5781	1059.5924	-13.51	96	-	105	1	3	K.KGTTLQGDLK.A
1140.5769	1139.5697	1139.5393	26.6	27	-	35	0	---	K.QTMEIHHTK.H + Oxidation
1706.7773	1705.7700	1705.7849	-8.69	193	-	206	0	70	K.EFWNVVNWDEAAAR.F

No match to: 807.3999, 832.4835, 856.5285, 870.5457, 897.4207, 943.5581, 973.5402, 1028.4847, 1037.5513, 1051.6991, 1052.6447, 1064.6072, 1066.5148, 1074.5430, 1090.5585, 1107.5630, 1109.5211, 1111.5941, 1150.6550, 1157.6067, 1184.6118, 1193.6299, 1201.6527, 1234.6902, 1262.6852, 1265.6542, 1277.7111, 1307.6798, 1308.6631, 1314.7742, 1320.5936, 1323.6793, 1329.6530, 1338.6701, 1340.6933, 1357.7238, 1365.6565, 1373.6674, 1383.6947, 1390.6914, 1393.7380, 1427.8365, 1434.7666, 1458.7253, 1475.7605, 1487.7463, 1493.7394, 1566.7300, 1586.7234, 1618.7046, 1632.7336, 1657.7836, 1688.7715, 1699.8070, 1708.7559, 1722.7759, 1738.7724, 1754.7672, 1770.7611, 1817.9139, 1838.8982, 1851.8839, 1940.8981, 2225.0635, 2286.0353, 2383.9128, 2398.9758, 2705.1300, 2717.0471, 3312.3408

22. gi|160866780 **Mass:** 23685 **Score:** 85 **Expect:** 0.0079 **Queries matched:** 3

hypothetical protein SARI_03588 [Salmonella enterica subsp. arizonae serovar 62:z4,z23:--

Observed	Mr(expt)	Mr(calc)	ppm	Start		End	Miss	Ions	Peptide
1060.5854	1059.5781	1059.5924	-13.51	95	-	104	1	3	K.KGTTLQGDLK.A
1140.5769	1139.5697	1139.5393	26.6	26	-	34	0	---	K.QTMEIHHTK.H + Oxidation
1706.7773	1705.7700	1705.7849	-8.69	192	-	205	0	70	K.EFWNVVNWDEAAAR.F

No match to: 807.3999, 832.4835, 856.5285, 870.5457, 897.4207, 943.5581, 973.5402, 1028.4847, 1037.5513, 1051.6991, 1052.6447, 1064.6072, 1066.5148, 1074.5430, 1090.5585, 1107.5630, 1109.5211, 1111.5941, 1150.6550, 1157.6067, 1184.6118, 1193.6299, 1201.6527, 1234.6902, 1262.6852, 1265.6542, 1277.7111, 1307.6798, 1308.6631, 1314.7742, 1320.5936, 1323.6793, 1329.6530, 1338.6701, 1340.6933, 1357.7238, 1365.6565, 1373.6674, 1383.6947, 1390.6914, 1393.7380, 1427.8365, 1434.7666, 1458.7253, 1475.7605, 1487.7463, 1493.7394, 1566.7300, 1586.7234, 1618.7046, 1632.7336, 1657.7836, 1688.7715, 1699.8070, 1708.7559, 1722.7759, 1738.7724, 1754.7672, 1770.7611, 1817.9139, 1838.8982, 1851.8839, 1940.8981, 2225.0635, 2286.0353, 2383.9128, 2398.9758, 2705.1300, 2717.0471, 3312.3408

23. gi|110833822 **Mass:** 35956 **Score:** 79 **Expect:** 0.035 **Queries matched:** 11

hypothetical protein ABO_0961 [Alcanivorax borkumensis SK2]

Observed	Mr(expt)	Mr(calc)	ppm	Start		End	Miss	Ions	Peptide

1028.4847	1027.4774	1027.4498	26.8	32 -	39	0	8	R.LDFEFDDK.T
1060.5854	1059.5781	1059.6176	-37.23	168 -	176	1	4	K.KLIDETTLK.L
1150.6550	1149.6477	1149.6182	25.7	235 -	243	1	---	R.LFDLKSNWK.G
1184.6118	1183.6045	1183.5509	45.2	31 -	39	1	---	R.RLDFEFDDK.T
1234.6902	1233.6829	1233.7081	-20.45	19 -	30	0	---	K.VPLGATVDIPPR.R
1365.6565	1364.6492	1364.7122	-46.16	228 -	239	1	---	R.MLASDGRLFDLK.S
1390.6914	1389.6841	1389.8092	-90.05	19 -	31	1	---	K.VPLGATVDIPPRR.L
1427.8365	1426.8292	1426.7609	47.9	258 -	268	1	---	K.LTPQYLDFFKR.D
1722.7759	1721.7687	1721.8795	-64.40	2 -	18	1	---	M.TTTASTTIESGQAKAQK.V
1817.9139	1816.9066	1816.9764	-38.40	253 -	267	1	---	K.GLFTKLTPQYLDFFK.R
2705.1300	2704.1227	2704.2560	-49.27	44 -	65	0	---	R.YFYDNDPFLSAFWLSMSTLFPK.G

No match to: 807.3999, 832.4835, 856.5285, 870.5457, 897.4207, 943.5581, 973.5402, 1037.5513, 1051.6991, 1052.6447, 1064.6072, 1066.5148, 1074.5430, 1090.5585, 1107.5630, 1109.5211, 1111.5941, 1140.5769, 1157.6067, 1193.6299, 1201.6527, 1262.6852, 1265.6542, 1277.7111, 1307.6798, 1308.6631, 1314.7742, 1320.5936, 1323.6793, 1329.6530, 1338.6701, 1340.6933, 1357.7238, 1373.6674, 1383.6947, 1393.7380, 1434.7666, 1458.7253, 1475.7605, 1487.7463, 1493.7394, 1566.7300, 1586.7234, 1618.7046, 1632.7336, 1657.7836, 1688.7715, 1699.8070, 1706.7773, 1708.7559, 1738.7724, 1754.7672, 1770.7611, 1838.8982, 1851.8839, 1940.8981, 2225.0635, 2286.0353, 2383.9128, 2398.9758, 2717.0471, 3312.3408

24. gi|116006777 **Mass:** 82342 **Score:** 76 **Expect:** 0.064 **Queries matched:** 15

TraI [Bordetella pertussis]

Observed	Mr(expt)	Mr(calc)	ppm	Start	End	Miss	Ions	Peptide
943.5581	942.5508	942.5498	1.02	598 -	606	0	---	R.ALSELGVVR.I
1074.5430	1073.5357	1073.5730	-34.75	587 -	595	0	---	R.KPPPQSQHR.L
1090.5585	1089.5512	1089.5414	8.94	288 -	297	0	---	R.ADQQSLTTAR.G
1107.5630	1106.5557	1106.5972	-37.50	471 -	480	0	---	R.ITVNGTVEFK.A
1111.5941	1110.5868	1110.6397	-47.63	607 -	617	0	---	R.IAGGSEVLLPR.D
1140.5769	1139.5697	1139.6307	-53.59	1 -	9	1	---	-.MILKHVPMR.S + Oxidation
1234.6902	1233.6829	1233.6638	15.4	140 -	150	1	---	R.TMADIAVKLEK.E + Oxidatio
1265.6542	1264.6469	1264.5466	79.3	433 -	444	1	---	R.AGMSAVRDDGDR.L + Oxidati
1373.6674	1372.6601	1372.6735	-9.78	151 -	162	0	---	K.EFGLQQVNHSSK.R
1390.6914	1389.6841	1389.6459	27.5	93 -	104	0	---	R.VCAALGYSEHQR.I

1393.7380	1392.7307	1392.6245	76.3	129 - 139	0	---	R.NTMHEPFQAYR.T
1632.7336	1631.7263	1631.7647	-23.54	704 - 718	0	---	R.GDEVMVMPIDQATAR.R
1706.7773	1705.7700	1705.9185	-87.04	471 - 485	1	14	R.ITVNGTVEFKAQMIR.A
1722.7759	1721.7687	1721.9134	-84.08	471 - 485	1	---	R.ITVNGTVEFKAQMIR.A + Oxid
3312.3408	3311.3335	3311.5663	-70.28	189 - 215	1	---	R.ECLDEITNAQTWQELHQVMRENGL

No match to: 807.3999, 832.4835, 856.5285, 870.5457, 897.4207, 973.5402, 1028.4847, 1037.5513, 1051.6991, 1052.6447, 1060.5854, 1064.6072, 1066.5148, 1109.5211, 1150.6550, 1157.6067, 1184.6118, 1193.6299, 1201.6527, 1262.6852, 1277.7111, 1307.6798, 1308.6631, 1314.7742, 1320.5936, 1323.6793, 1329.6530, 1338.6701, 1340.6933, 1357.7238, 1365.6565, 1383.6947, 1427.8365, 1434.7666, 1458.7253, 1475.7605, 1487.7463, 1493.7394, 1566.7300, 1586.7234, 1618.7046, 1657.7836, 1688.7715, 1699.8070, 1708.7559, 1738.7724, 1754.7672, 1770.7611, 1817.9139, 1838.8982, 1851.8839, 1940.8981, 2225.0635, 2286.0353, 2383.9128, 2398.9758, 2705.1300, 2717.0471

25. gi|148240893 **Mass:** 44158 **Score:** 75 **Expect:** 0.081 **Queries matched:** 12
Putative cAMP-induced filamentation protein [Bradyrhizobium sp. BTAi1]

Observed	Mr(expt)	Mr(calc)	ppm	Start - End	Miss	Ions	Peptide
1052.6447	1051.6374	1051.6502	-12.13	142 - 150	1	---	K.IRIVPGELR.K
1060.5854	1059.5781	1059.5938	-14.77	189 - 198	0	4	K.IIGVAASHHR.L
1074.5430	1073.5357	1073.5870	-47.74	371 - 380	0	---	K.LGFPTAVVDR.W
1234.6902	1233.6829	1233.6295	43.3	381 - 390	1	---	R.WFPRLYQPAG.-
1277.7111	1276.7038	1276.6048	77.6	334 - 345	0	---	R.GNAPALTGYEER.Q
1323.6793	1322.6720	1322.5812	68.6	301 - 310	0	---	R.MEIWTEEETR.A
1383.6947	1382.6874	1382.7088	-15.50	91 - 101	1	---	R.AHIEVQRMIDR.G + Oxidatio
1458.7253	1457.7180	1457.7613	-29.70	15 - 27	0	---	R.QELEDLATDLLAK.A
1487.7463	1486.7391	1486.6973	28.1	2 - 14	0	---	M.DVSAMEPLLPEDR.Q + Oxidat
1618.7046	1617.6974	1617.7378	-25.01	1 - 14	0	---	-.MDVSAMEPLLPEDR.Q + Oxida
1632.7336	1631.7263	1631.8015	-46.10	334 - 348	1	---	R.GNAPALTGYEERQAR.T
1851.8839	1850.8766	1850.9904	-61.46	199 - 214	1	---	R.LAWIHPFLDGNGRVTR.L

No match to: 807.3999, 832.4835, 856.5285, 870.5457, 897.4207, 943.5581, 973.5402, 1028.4847, 1037.5513, 1051.6991, 1064.6072, 1066.5148, 1090.5585, 1107.5630, 1109.5211, 1111.5941, 1140.5769, 1150.6550, 1157.6067, 1184.6118, 1193.6299, 1201.6527, 1262.6852, 1265.6542, 1307.6798, 1308.6631, 1314.7742, 1320.5936, 1329.6530, 1338.6701, 1340.6933,

1357.7238, 1365.6565, 1373.6674, 1390.6914, 1393.7380, 1427.8365, 1434.7666, 1475.7605, 1493.7394, 1566.7300, 1586.7234, 1657.7836, 1688.7715, 1699.8070, 1706.7773, 1708.7559, 1722.7759, 1738.7724, 1754.7672, 1770.7611, 1817.9139, 1838.8982, 1940.8981, 2225.0635, 2286.0353, 2383.9128, 2398.9758, 2705.1300, 2717.0471, 3312.3408

26. gi|58039077 **Mass:** 37927 **Score:** 75 **Expect:** 0.093 **Queries matched:** 12
D-alanyl-D-alanine carboxypeptidase [Gluconobacter oxydans 621H]

Observed	Mr(expt)	Mr(calc)	ppm	Start	End	Miss	Ions	Peptide
1111.5941	1110.5868	1110.5492	33.9	146 -	155	0	---	R.ALGMTNTTFR.N
1157.6067	1156.5994	1156.6314	-27.67	60 -	68	0	---	K.LMTLYLTFR.A
1184.6118	1183.6045	1183.5464	49.1	255 -	264	0	---	R.SMEMVSLLDK.G + 2 Oxidati
1193.6299	1192.6226	1192.6023	17.1	136 -	145	0	---	R.FAQLMTQQAR.A
1234.6902	1233.6829	1233.6764	5.24	332 -	342	1	---	R.MVSARHVVAHK.K
1308.6631	1307.6558	1307.6577	-1.44	254 -	264	1	---	R.RSMEMVSLLDK.G
1323.6793	1322.6720	1322.6956	-17.82	320 -	331	1	---	R.YGRISHHGAAVR.M
1340.6933	1339.6860	1339.6476	28.7	254 -	264	1	---	R.RSMEMVSLLDK.G + 2 Oxidat
1357.7238	1356.7166	1356.7184	-1.32	241 -	253	0	---	R.LIGVVMGAPNNTR.R + Oxidat
1657.7836	1656.7763	1656.8037	-16.55	8 -	23	0	---	K.AFLLCAMTAMGLGSAK.A + Oxi
1817.9139	1816.9066	1816.9068	-0.09	220 -	236	0	---	K.TGYTDLAGHNLITSAQR.G
2286.0353	2285.0280	2285.1409	-49.39	136 -	155	1	---	R.FAQLMTQQARALGMTNTTFR.N

No match to: 807.3999, 832.4835, 856.5285, 870.5457, 897.4207, 943.5581, 973.5402, 1028.4847, 1037.5513, 1051.6991, 1052.6447, 1060.5854, 1064.6072, 1066.5148, 1074.5430, 1090.5585, 1107.5630, 1109.5211, 1140.5769, 1150.6550, 1201.6527, 1262.6852, 1265.6542, 1277.7111, 1307.6798, 1314.7742, 1320.5936, 1329.6530, 1338.6701, 1365.6565, 1373.6674, 1383.6947, 1390.6914, 1393.7380, 1427.8365, 1434.7666, 1458.7253, 1475.7605, 1487.7463, 1493.7394, 1566.7300, 1586.7234, 1618.7046, 1632.7336, 1688.7715, 1699.8070, 1706.7773, 1708.7559, 1722.7759, 1738.7724, 1754.7672, 1770.7611, 1838.8982, 1851.8839, 1940.8981, 2225.0635, 2383.9128, 2398.9758, 2705.1300, 2717.0471, 3312.3408

27. gi|109649126 **Mass:** 93680 **Score:** 72 **Expect:** 0.17 **Queries matched:** 19
ATP dependent DNA ligase [Desulfitobacterium hafniense DCB-2]

Observed	Mr(expt)	Mr(calc)	ppm	Start	End	Miss	Ions	Peptide
807.3999	806.3927	806.4286	-44.61	418 -	424	0	---	R.ELVYAGR.A

856.5285	855.5212	855.5654	-51.61	579 - 585	1	---	R.RILSIVR.C
973.5402	972.5329	972.5392	-6.50	377 - 384	1	---	R.NGDWIKLK.C
1037.5513	1036.5441	1036.4938	48.5	63 - 71	1	---	K.GPSYNTRDK.R
1201.6527	1200.6455	1200.5961	41.1	589 - 598	1	---	K.GVAQTCFYKK.H
1262.6852	1261.6780	1261.7156	-29.84	33 - 42	1	---	R.FVVQHHLARR.D
1320.5936	1319.5864	1319.6721	-64.99	196 - 207	0	---	R.NPFSSTEVQLAK.L
1340.6933	1339.6860	1339.6772	6.57	283 - 293	0	---	K.TDFQALQNYLK.N
1393.7380	1392.7307	1392.6674	45.5	476 - 486	0	---	K.FADWTEENLLR.Q
1434.7666	1433.7593	1433.7006	40.9	354 - 367	1	---	R.AACEAGMEGIIGKK.A
1458.7253	1457.7180	1457.6796	26.4	586 - 597	1	---	R.CPKGVAQTCFYK.K
1618.7046	1617.6974	1617.7861	-54.86	49 - 62	0	---	R.LEWDGAMLSWAVPK.G + Oxida
1688.7715	1687.7642	1687.9257	-95.68	196 - 210	1	---	R.NPFSSTEVQLAKLVR.H
1706.7773	1705.7700	1705.7903	-11.85	267 - 282	0	2	R.AMILDGEMAITDPEGK.T + Oxi
1722.7759	1721.7687	1721.7852	-9.59	267 - 282	0	---	R.AMILDGEMAITDPEGK.T + 2 O
1738.7724	1737.7652	1737.7727	-4.32	178 - 192	1	---	R.TGRTMTEIEEGEEEK.F
1754.7672	1753.7599	1753.7676	-4.39	178 - 192	1	---	R.TGRTMTEIEEGEEEK.F + Oxid
2225.0635	2224.0562	2224.0589	-1.22	211 - 228	1	---	R.HLPEGEGWLYELKYDGYR.I
2286.0353	2285.0280	2285.1176	-39.18	158 - 177	1	---	K.EKDDYVQTADGISQFITSIR.T

No match to: 832.4835, 870.5457, 897.4207, 943.5581, 1028.4847, 1051.6991, 1052.6447, 1060.5854, 1064.6072, 1066.5148, 1074.5430, 1090.5585, 1107.5630, 1109.5211, 1111.5941, 1140.5769, 1150.6550, 1157.6067, 1184.6118, 1193.6299, 1234.6902, 1265.6542, 1277.7111, 1307.6798, 1308.6631, 1314.7742, 1323.6793, 1329.6530, 1338.6701, 1357.7238, 1365.6565, 1373.6674, 1383.6947, 1390.6914, 1427.8365, 1475.7605, 1487.7463, 1493.7394, 1566.7300, 1586.7234, 1632.7336, 1657.7836, 1699.8070, 1708.7559, 1770.7611, 1817.9139, 1838.8982, 1851.8839, 1940.8981, 2383.9128, 2398.9758, 2705.1300, 2717.0471, 3312.3408

28. gi|89076064 **Mass:** 84568 **Score:** 68 **Expect:** 0.44 **Queries matched:** 14
hypothetical protein SKA34_16590 [Photobacterium sp. SKA34]

Observed	Mr(expt)	Mr(calc)	ppm	Start - End	Miss	Ions	Peptide
1111.5941	1110.5868	1110.5855	1.13	316 - 326	0	---	K.DLLMAAPAGPR.V
1201.6527	1200.6455	1200.6536	-6.81	703 - 712	1	---	K.VKVMEVDLPR.K + Oxidation
1234.6902	1233.6829	1233.6969	-11.32	394 - 404	1	---	R.ETDAFVAKLLK.D
1265.6542	1264.6469	1264.6299	13.4	635 - 646	0	---	K.TATFAEGVNEVK.D

1277.7111	1276.7038	1276.6748	22.7	733 - 743	1	---	R.QPRDNAPRPAR.Q
1373.6674	1372.6601	1372.7310	-51.67	185 - 197	1	---	K.GQAELTSRLVDGK.E
1427.8365	1426.8292	1426.8144	10.4	327 - 340	1	---	R.VTLALDPGLRTGSK.I
1586.7234	1585.7161	1585.8563	-88.39	82 - 96	1	---	K.LTPALEAEIKTADSK.T
1657.7836	1656.7763	1656.8795	-62.24	2 - 16	0	8	M.SNSINQLIASELNVR.N
1706.7773	1705.7700	1705.8201	-29.35	286 - 299	0	6	K.ILMHMETELMSALR.E + 2 Oxi
1722.7759	1721.7687	1721.8150	-26.92	286 - 299	0	---	K.ILMHMETELMSALR.E + 3 Oxi
1754.7672	1753.7599	1753.7842	-13.86	752 - 770	0	5	R.DNGNAGMGGAFAAAFANAK.K
1770.7611	1769.7538	1769.7791	-14.31	752 - 770	0	(2)	R.DNGNAGMGGAFAAAFANAK.K +
1851.8839	1850.8766	1850.9448	-36.81	163 - 178	1	---	R.AILMERFAEDATLLDK.I + Oxi

No match to: 807.3999, 832.4835, 856.5285, 870.5457, 897.4207, 943.5581, 973.5402, 1028.4847, 1037.5513, 1051.6991, 1052.6447, 1060.5854, 1064.6072, 1066.5148, 1074.5430, 1090.5585, 1107.5630, 1109.5211, 1140.5769, 1150.6550, 1157.6067, 1184.6118, 1193.6299, 1262.6852, 1307.6798, 1308.6631, 1314.7742, 1320.5936, 1323.6793, 1329.6530, 1338.6701, 1340.6933, 1357.7238, 1365.6565, 1383.6947, 1390.6914, 1393.7380, 1434.7666, 1458.7253, 1475.7605, 1487.7463, 1493.7394, 1566.7300, 1618.7046, 1632.7336, 1688.7715, 1699.8070, 1708.7559, 1738.7724, 1817.9139, 1838.8982, 1940.8981, 2225.0635, 2286.0353, 2383.9128, 2398.9758, 2705.1300, 2717.0471, 3312.3408

29. gi|91778672 **Mass:** 48306 **Score:** 66 **Expect:** 0.66 **Queries matched:** 13
Electron-transferring-flavoprotein dehydrogenase [Burkholderia xenovorans LB400]

Observed	Mr(expt)	Mr(calc)	ppm	Start - End	Miss	Ions	Peptide
1052.6447	1051.6374	1051.6026	33.2	329 - 338	0	---	R.LAAETVIHAK.A
1107.5630	1106.5557	1106.5642	-7.66	395 - 404	1	---	R.TMLTVDGKDK.K
1109.5211	1108.5138	1108.5587	-40.43	354 - 363	0	---	K.AALDQSFVMK.D
1111.5941	1110.5868	1110.5379	44.0	420 - 430	0	---	R.SLAGMVGDAYK.L
1308.6631	1307.6558	1307.7159	-45.92	254 - 264	1	---	R.TSPYVLLEKMK.Q
1338.6701	1337.6628	1337.6761	-9.95	418 - 430	1	---	K.ARSLAGMVGDAYK.L
1393.7380	1392.7307	1392.6959	25.0	190 - 200	0	---	K.EILFMPEETIR.Q + Oxidatio
1475.7605	1474.7533	1474.7780	-16.75	32 - 44	1	---	K.VLQIERGEYPGSK.N
1487.7463	1486.7391	1486.7490	-6.66	351 - 363	1	---	K.AYKAALDQSFVMK.D + Oxidat
1493.7394	1492.7322	1492.8072	-50.24	420 - 433	1	---	R.SLAGMVGDAYKLLR.A
1618.7046	1617.6974	1617.7709	-45.44	219 - 233	0	---	R.ITDGMTGTGFLYTNK.E

1754.7672	1753.7599	1753.8743	-65.22	234 - 249	1	---	K.ESLTIGVGCMLGDFKK.N
1770.7611	1769.7538	1769.8692	-65.21	234 - 249	1	---	K.ESLTIGVGCMLGDFKK.N + Oxi

No match to: 807.3999, 832.4835, 856.5285, 870.5457, 897.4207, 943.5581, 973.5402, 1028.4847, 1037.5513, 1051.6991, 1060.5854, 1064.6072, 1066.5148, 1074.5430, 1090.5585, 1140.5769, 1150.6550, 1157.6067, 1184.6118, 1193.6299, 1201.6527, 1234.6902, 1262.6852, 1265.6542, 1277.7111, 1307.6798, 1314.7742, 1320.5936, 1323.6793, 1329.6530, 1340.6933, 1357.7238, 1365.6565, 1373.6674, 1383.6947, 1390.6914, 1427.8365, 1434.7666, 1458.7253, 1566.7300, 1586.7234, 1632.7336, 1657.7836, 1688.7715, 1699.8070, 1706.7773, 1708.7559, 1722.7759, 1738.7724, 1817.9139, 1838.8982, 1851.8839, 1940.8981, 2225.0635, 2286.0353, 2383.9128, 2398.9758, 2705.1300, 2717.0471, 3312.3408

30. gi|146282034 **Mass:** 37412 **Score:** 66 **Expect:** 0.71 **Queries matched:** 10

TIM-barrel protein, yjbN family [Pseudomonas stutzeri A1501]

Observed	Mr(expt)	Mr(calc)	ppm	Start End	Miss	Ions	Peptide
807.3999	806.3927	806.3592	41.4	221 - 226	0	---	K.TLEECR.E
1052.6447	1051.6374	1051.6026	33.1	308 - 316	0	---	R.QLLSVDIHK.T
1308.6631	1307.6558	1307.7099	-41.32	292 - 304	0	---	R.HVLGLGQGFPGAR.R
1390.6914	1389.6841	1389.6572	19.4	167 - 178	1	---	R.EAGCRSFTVHAR.I
1487.7463	1486.7391	1486.6221	78.7	18 - 29	0	---	R.FSVAPMMDWTDR.H + 2 Oxida
1699.8070	1698.7998	1698.9305	-76.92	206 - 220	1	---	K.RDFPDLEIILNGGIK.T
1838.8982	1837.8909	1838.0414	-81.86	172 - 188	1	---	R.SFTVHARIAILEGLSPK.Q
1940.8981	1939.8908	1939.8128	40.2	18 - 32	1	---	R.FSVAPMMDWTDRHCR.F + 2 Ox
2286.0353	2285.0280	2285.1474	-52.25	41 - 61	0	---	K.HALLYTEMVTTGALLHGDTAR.F
2705.1300	2704.1227	2704.3881	-98.13	279 - 304	1	---	R.HLAEGGAMHHVTRHVLGLGQGFPG

No match to: 832.4835, 856.5285, 870.5457, 897.4207, 943.5581, 973.5402, 1028.4847, 1037.5513, 1051.6991, 1060.5854, 1064.6072, 1066.5148, 1074.5430, 1090.5585, 1107.5630, 1109.5211, 1111.5941, 1140.5769, 1150.6550, 1157.6067, 1184.6118, 1193.6299, 1201.6527, 1234.6902, 1262.6852, 1265.6542, 1277.7111, 1307.6798, 1314.7742, 1320.5936, 1323.6793, 1329.6530, 1338.6701, 1340.6933, 1357.7238, 1365.6565, 1373.6674, 1383.6947, 1393.7380, 1427.8365, 1434.7666, 1458.7253, 1475.7605, 1493.7394, 1566.7300, 1586.7234, 1618.7046, 1632.7336, 1657.7836, 1688.7715, 1706.7773, 1708.7559, 1722.7759, 1738.7724, 1754.7672, 1770.7611, 1817.9139, 1851.8839, 2225.0635, 2383.9128, 2398.9758, 2717.0471, 3312.3408

31. gi|75675707 **Mass:** 76662 **Score:** 65 **Expect:** 0.81 **Queries matched:** 12

DEAD/DEAH box helicase [Nitrobacter winogradskyi Nb-255]

Observed	Mr(expt)	Mr(calc)	ppm	Start		End	Miss	Ions	Peptide
870.5457	869.5384	869.4970	47.6	339	-	346	0	---	K.TIAPLAER.A
973.5402	972.5329	972.5240	9.16	296	-	305	0	---	R.LLQGDVGSGK.T
1060.5854	1059.5781	1059.5019	72.0	597	-	606	0	---	R.EPLGEMSAAR.L
1184.6118	1183.6045	1183.6462	-35.24	513	-	523	1	---	R.FGDRVGLVHGK.M
1193.6299	1192.6226	1192.6200	2.16	609	-	618	1	---	R.VIRETTDGFR.I
1314.7742	1313.7669	1313.6688	74.7	455	-	466	1	---	R.QPIDTRAVSDGR.L
1323.6793	1322.6720	1322.7306	-44.32	648	-	659	0	---	R.SDVHAQLITLAR.D
1329.6530	1328.6458	1328.6870	-31.05	597	-	608	1	---	R.EPLGEMSAARLR.V
1373.6674	1372.6601	1372.7497	-65.24	293	-	305	1	---	R.MLRLLQGDVGSGK.T
1493.7394	1492.7322	1492.7382	-4.07	668	-	681	0	4	K.QNPNLTGSHGEALR.C
1657.7836	1656.7763	1656.8795	-62.27	461	-	476	1	14	R.AVSDGRLTEVIDAVGR.A
2225.0635	2224.0562	2224.1164	-27.10	87	-	106	0	---	R.APHLVYASDETGSVVLTYFR.A

No match to: 807.3999, 832.4835, 856.5285, 897.4207, 943.5581, 1028.4847, 1037.5513, 1051.6991, 1052.6447, 1064.6072, 1066.5148, 1074.5430, 1090.5585, 1107.5630, 1109.5211, 1111.5941, 1140.5769, 1150.6550, 1157.6067, 1201.6527, 1234.6902, 1262.6852, 1265.6542, 1277.7111, 1307.6798, 1308.6631, 1320.5936, 1338.6701, 1340.6933, 1357.7238, 1365.6565, 1383.6947, 1390.6914, 1393.7380, 1427.8365, 1434.7666, 1458.7253, 1475.7605, 1487.7463, 1566.7300, 1586.7234, 1618.7046, 1632.7336, 1688.7715, 1699.8070, 1706.7773, 1708.7559, 1722.7759, 1738.7724, 1754.7672, 1770.7611, 1817.9139, 1838.8982, 1851.8839, 1940.8981, 2286.0353, 2383.9128, 2398.9758, 2705.1300, 2717.0471, 3312.3408

32. gi|149279903 **Mass:** 89251 **Score:** 65 **Expect:** 0.91 **Queries matched:** 11

TonB-linked outer membrane receptor [Pedobacter sp. BAL39]

Observed	Mr(expt)	Mr(calc)	ppm	Start		End	Miss	Ions	Peptide
870.5457	869.5384	869.4759	71.8	784	-	790	1	---	R.SFSVKFR.Y
1037.5513	1036.5441	1036.4825	59.4	157	-	166	0	---	R.EQGGLGSDFK.F
1193.6299	1192.6226	1192.6088	11.6	252	-	262	0	9	R.SALTGQFVDQK.T
1234.6902	1233.6829	1233.6394	35.3	614	-	623	0	---	K.DFIYPVPDLR.S
1265.6542	1264.6469	1264.6299	13.4	123	-	134	0	---	K.ESGFAVNAIETK.K
1277.7111	1276.7038	1276.7139	-7.93	167	-	178	1	---	K.FSINGLSGKQVK.F
1320.5936	1319.5864	1319.6681	-61.95	82	-	94	0	13	K.SSQSVTLSAGQQK.T

1393.7380	1392.7307	1392.7249	4.19	123 - 135	1	---	K.ESGFAVNAIETKK.F
1657.7836	1656.7763	1656.8319	-33.53	103 - 117	1	---	K.DPQQLDNVSIDGKTK.N
1754.7672	1753.7599	1753.8635	-59.07	380 - 395	0	1	K.NDIFTEGLNLSAFASR.T
1940.8981	1939.8908	1939.9640	-37.72	157 - 175	1	---	R.EQGGLGSDFKFSINGLSGK.Q

No match to: 807.3999, 832.4835, 856.5285, 897.4207, 943.5581, 973.5402, 1028.4847, 1051.6991, 1052.6447, 1060.5854, 1064.6072, 1066.5148, 1074.5430, 1090.5585, 1107.5630, 1109.5211, 1111.5941, 1140.5769, 1150.6550, 1157.6067, 1184.6118, 1201.6527, 1262.6852, 1307.6798, 1308.6631, 1314.7742, 1323.6793, 1329.6530, 1338.6701, 1340.6933, 1357.7238, 1365.6565, 1373.6674, 1383.6947, 1390.6914, 1427.8365, 1434.7666, 1458.7253, 1475.7605, 1487.7463, 1493.7394, 1566.7300, 1586.7234, 1618.7046, 1632.7336, 1688.7715, 1699.8070, 1706.7773, 1708.7559, 1722.7759, 1738.7724, 1770.7611, 1817.9139, 1838.8982, 1851.8839, 2225.0635, 2286.0353, 2383.9128, 2398.9758, 2705.1300, 2717.0471, 3312.3408

33. gi|124010167 **Mass:** 17818 **Score:** 64 **Expect:** 1.1 **Queries matched:** 8

hypothetical protein M23134_01768 [Microscilla marina ATCC 23134]

Observed	Mr(expt)	Mr(calc)	ppm	Start End	Miss	Ions	Peptide
1064.6072	1063.5999	1063.5550	42.2	23 - 31	0	---	K.EIDAQTFIK.I
1234.6902	1233.6829	1233.6645	14.9	143 - 152	0	---	K.LPIEVLYQYP.-
1323.6793	1322.6720	1322.7095	-28.34	107 - 117	1	---	K.DKNGVYLHIHK.V
1390.6914	1389.6841	1389.7656	-58.67	82 - 93	1	---	R.VTIYKLGWADPK.T
1393.7380	1392.7307	1392.6132	84.4	54 - 63	1	---	K.NVEYFYCKDR.R
1487.7463	1486.7391	1486.7456	-4.40	94 - 106	0	---	K.TFTTNNALFPYAK.D
1738.7724	1737.7652	1737.9050	-80.44	64 - 77	1	---	R.RGVYLIEEVFTQER.F
1838.8982	1837.8909	1837.8570	18.5	1 - 15	1	---	-.MFSKNNQVFYYNAGR.H

No match to: 807.3999, 832.4835, 856.5285, 870.5457, 897.4207, 943.5581, 973.5402, 1028.4847, 1037.5513, 1051.6991, 1052.6447, 1060.5854, 1066.5148, 1074.5430, 1090.5585, 1107.5630, 1109.5211, 1111.5941, 1140.5769, 1150.6550, 1157.6067, 1184.6118, 1193.6299, 1201.6527, 1262.6852, 1265.6542, 1277.7111, 1307.6798, 1308.6631, 1314.7742, 1320.5936, 1329.6530, 1338.6701, 1340.6933, 1357.7238, 1365.6565, 1373.6674, 1383.6947, 1427.8365, 1434.7666, 1458.7253, 1475.7605, 1493.7394, 1566.7300, 1586.7234, 1618.7046, 1632.7336, 1657.7836, 1688.7715, 1699.8070, 1706.7773, 1708.7559, 1722.7759, 1754.7672, 1770.7611, 1817.9139, 1851.8839, 1940.8981, 2225.0635, 2286.0353, 2383.9128, 2398.9758, 2705.1300, 2717.0471, 3312.3408

34. gi|30263944 **Mass:** 104494 **Score:** 64 **Expect:** 1.1 **Queries matched:** 16
prophage LambdaBa02, tape measure protein, putative [Bacillus anthracis str. Ames]

Observed	Mr(expt)	Mr(calc)	ppm	Start		End	Miss	Ions	Peptide
832.4835	831.4763	831.4450	37.5	11	-	17	0	---	R.VTLDTQR.F
1028.4847	1027.4774	1027.4280	48.0	225	-	232	0	---	K.ESMNFEQK.M + Oxidation (
1060.5854	1059.5781	1059.5812	-2.90	543	-	552	0	---	K.VTALEVAETK.M
1066.5148	1065.5075	1065.4583	46.2	1	-	10	0	---	-.MAGDMEIGAR.V + Oxidation
1107.5630	1106.5557	1106.5641	-7.63	490	-	499	0	---	K.ISGLLNESMK.D + Oxidation
1277.7111	1276.7038	1276.6333	55.2	249	-	260	0	---	K.QIGDLAVTMGEK.T + Oxidati
1307.6798	1306.6725	1306.6737	-0.90	390	-	400	1	---	K.TMLMRLNPSTK.E + Oxidatio
1308.6631	1307.6558	1307.6213	26.4	694	-	704	1	---	K.DKAINNMMDLK.L + Oxidatio
1323.6793	1322.6720	1322.6686	2.56	390	-	400	1	---	K.TMLMRLNPSTK.E + 2 Oxidat
1390.6914	1389.6841	1389.6810	2.25	833	-	846	0	---	K.EIEAGGMLSLDAGK.K
1393.7380	1392.7307	1392.7222	6.13	175	-	185	1	---	R.ELAEQQNRLHR.T
1458.7253	1457.7180	1457.8089	-62.38	558	-	571	1	---	K.GKIEQLSGAVDTLK.K
1487.7463	1486.7391	1486.7124	17.9	171	-	182	1	---	R.ETNRELAEQQNR.L
1657.7836	1656.7763	1656.8029	-16.01	678	-	693	1	5	R.VSEATSKAAGAYMDLK.D + Oxi
1688.7715	1687.7642	1687.8239	-35.40	348	-	365	0	---	K.YGLSASSAVAAGAGMTFK.D
1851.8839	1850.8766	1850.9625	-46.40	261	-	277	1	---	K.TKYSSVEAGQGIEELIK.A

No match to: 807.3999, 856.5285, 870.5457, 897.4207, 943.5581, 973.5402, 1037.5513, 1051.6991, 1052.6447, 1064.6072, 1074.5430, 1090.5585, 1109.5211, 1111.5941, 1140.5769, 1150.6550, 1157.6067, 1184.6118, 1193.6299, 1201.6527, 1234.6902, 1262.6852, 1265.6542, 1314.7742, 1320.5936, 1329.6530, 1338.6701, 1340.6933, 1357.7238, 1365.6565, 1373.6674, 1383.6947, 1427.8365, 1434.7666, 1475.7605, 1493.7394, 1566.7300, 1586.7234, 1618.7046, 1632.7336, 1699.8070, 1706.7773, 1708.7559, 1722.7759, 1738.7724, 1754.7672, 1770.7611, 1817.9139, 1838.8982, 1940.8981, 2225.0635, 2286.0353, 2383.9128, 2398.9758, 2705.1300, 2717.0471, 3312.3408

35. gi|148244760 **Mass:** 13648 **Score:** 64 **Expect:** 1.1 **Queries matched:** 7
hypothetical protein COSY_0617 [Candidatus Vesicomyosocius okutanii HA]

Observed	Mr(expt)	Mr(calc)	ppm	Start		End	Miss	Ions	Peptide
1051.6991	1050.6918	1050.5961	91.1	14	-	22	0	---	K.TLLTITFDK.I
1234.6902	1233.6829	1233.6102	58.9	109	-	119	0	---	K.DIGHTHSQLNI.-

1262.6852	1261.6780	1261.6336	35.1	1 -	11	0	---	-.MQTPTNISLNK.E + Oxidatio
1308.6631	1307.6558	1307.7337	-59.51	12 -	22	1	---	K.EKTLLTITFDK.I
1338.6701	1337.6628	1337.6979	-26.21	23 -	33	0	---	K.INYPLSAEYLR.V
1475.7605	1474.7533	1474.7892	-24.37	107 -	119	1	---	K.LKDIGHTHSQLNI.-
1566.7300	1565.7228	1565.7375	-9.43	79 -	91	0	---	K.HDSGIYSWSHLHK.L

No match to: 807.3999, 832.4835, 856.5285, 870.5457, 897.4207, 943.5581, 973.5402, 1028.4847, 1037.5513, 1052.6447, 1060.5854, 1064.6072, 1066.5148, 1074.5430, 1090.5585, 1107.5630, 1109.5211, 1111.5941, 1140.5769, 1150.6550, 1157.6067, 1184.6118, 1193.6299, 1201.6527, 1265.6542, 1277.7111, 1307.6798, 1314.7742, 1320.5936, 1323.6793, 1329.6530, 1340.6933, 1357.7238, 1365.6565, 1373.6674, 1383.6947, 1390.6914, 1393.7380, 1427.8365, 1434.7666, 1458.7253, 1487.7463, 1493.7394, 1586.7234, 1618.7046, 1632.7336, 1657.7836, 1688.7715, 1699.8070, 1706.7773, 1708.7559, 1722.7759, 1738.7724, 1754.7672, 1770.7611, 1817.9139, 1838.8982, 1851.8839, 1940.8981, 2225.0635, 2286.0353, 2383.9128, 2398.9758, 2705.1300, 2717.0471, 3312.3408

36. gi|146279657 **Mass:** 56213 **Score:** 64 **Expect:** 1.2 **Queries matched:** 10

hypothetical protein Rsph17025_3642 [Rhodobacter sphaeroides ATCC 17025]

Observed	Mr(expt)	Mr(calc)	ppm	Start	End	Miss	Ions	Peptide
1060.5854	1059.5781	1059.5383	37.6	39 -	49	0	---	R.IVVCGPSGSGK.S
1150.6550	1149.6477	1149.5747	63.6	50 -	58	1	---	K.STMIRCINR.L
1157.6067	1156.5994	1156.6815	-70.99	323 -	335	0	---	R.SIGALAGALSAVK.G
1262.6852	1261.6780	1261.6700	6.30	297 -	308	1	---	R.VAGTSSIMPQKK.N + Oxidati
1277.7111	1276.7038	1276.6193	66.2	486 -	497	1	---	R.MEARLSADASAR.E
1308.6631	1307.6558	1307.6946	-29.62	203 -	213	1	---	R.EHERLAAVLDR.A
1329.6530	1328.6458	1328.6659	-15.17	458 -	468	1	---	R.WMDPVEAVRAR.T
1357.7238	1356.7166	1356.7473	-22.66	501 -	514	1	15	R.TAALAEAASRLAGR.V
1708.7559	1707.7487	1707.8475	-57.85	55 -	68	1	---	R.CINRLEEHQSGPIR.V
1838.8982	1837.8909	1837.8992	-4.52	385 -	401	1	---	R.CKANFSTATDLADGLVR.D

No match to: 807.3999, 832.4835, 856.5285, 870.5457, 897.4207, 943.5581, 973.5402, 1028.4847, 1037.5513, 1051.6991, 1052.6447, 1064.6072, 1066.5148, 1074.5430, 1090.5585, 1107.5630, 1109.5211, 1111.5941, 1140.5769, 1184.6118, 1193.6299, 1201.6527, 1234.6902, 1265.6542, 1307.6798, 1314.7742, 1320.5936, 1323.6793, 1338.6701, 1340.6933, 1365.6565, 1373.6674, 1383.6947, 1390.6914, 1393.7380, 1427.8365, 1434.7666, 1458.7253, 1475.7605, 1487.7463, 1493.7394, 1566.7300, 1586.7234, 1618.7046, 1632.7336, 1657.7836, 1688.7715,

1699.8070, 1706.7773, 1722.7759, 1738.7724, 1754.7672, 1770.7611, 1817.9139, 1851.8839, 1940.8981, 2225.0635, 2286.0353, 2383.9128, 2398.9758, 2705.1300, 2717.0471, 3312.3408

37. gi|15602483 **Mass:** 99875 **Score:** 63 **Expect:** 1.3 **Queries matched:** 15
PepN [Pasteurella multocida subsp. multocida str. Pm70]

Observed	Mr(expt)	Mr(calc)	ppm	Start		End	Miss	Ions	Peptide
1028.4847	1027.4774	1027.4835	-5.97	832	-	839	1	---	R.FSRYDGQR.Q
1037.5513	1036.5441	1036.5440	0.03	859	-	866	1	---	R.DLYEKIEK.A
1066.5148	1065.5075	1065.4549	49.4	234	-	241	1	---	R.SMKWDEDR.F
1107.5630	1106.5557	1106.5720	-14.76	429	-	438	0	---	R.ASGIDLTQFR.H
1109.5211	1108.5138	1108.5877	-66.61	32	-	41	0	---	K.TVVTATSQFR.R
1193.6299	1192.6226	1192.5625	50.4	795	-	805	0	---	K.AFHAIDGSGYR.F
1265.6542	1264.6469	1264.6888	-33.13	32	-	42	1	---	K.TVVTATSQFRR.L
1340.6933	1339.6860	1339.7572	-53.11	671	-	682	1	---	R.VNAEDIALRALR.N
1365.6565	1364.6492	1364.6725	-17.05	52	-	63	0	---	R.LDGHSFQFSSLK.L
1383.6947	1382.6874	1382.6103	55.8	328	-	338	0	---	R.DQEFTSDLWSR.S
1566.7300	1565.7228	1565.8024	-50.88	309	-	320	1	---	R.ITCRDWFQLSLK.E
1618.7046	1617.6974	1617.7933	-59.30	219	-	232	1	---	R.GNLDRAEWAMQSLK.R
1706.7773	1705.7700	1705.8821	-65.71	128	-	141	0	1	R.QITYMLDRPDVLAR.Y + Oxida
1838.8982	1837.8909	1837.9727	-44.47	313	-	327	1	---	R.DWFQLSLKEGLTVFR.D
2705.1300	2704.1227	2704.1857	-23.30	104	-	127	0	---	K.NTSLQGLYQSGEGICTQCEAEGFR

No match to: 807.3999, 832.4835, 856.5285, 870.5457, 897.4207, 943.5581, 973.5402, 1051.6991, 1052.6447, 1060.5854, 1064.6072, 1074.5430, 1090.5585, 1111.5941, 1140.5769, 1150.6550, 1157.6067, 1184.6118, 1201.6527, 1234.6902, 1262.6852, 1277.7111, 1307.6798, 1308.6631, 1314.7742, 1320.5936, 1323.6793, 1329.6530, 1338.6701, 1357.7238, 1373.6674, 1390.6914, 1393.7380, 1427.8365, 1434.7666, 1458.7253, 1475.7605, 1487.7463, 1493.7394, 1586.7234, 1632.7336, 1657.7836, 1688.7715, 1699.8070, 1708.7559, 1722.7759, 1738.7724, 1754.7672, 1770.7611, 1817.9139, 1851.8839, 1940.8981, 2225.0635, 2286.0353, 2383.9128, 2398.9758, 2717.0471, 3312.3408

38. gi|82749916 **Mass:** 67077 **Score:** 63 **Expect:** 1.3 **Queries matched:** 12
RGD-containing lipoprotein [Staphylococcus aureus RF122]

Observed	Mr(expt)	Mr(calc)	ppm	Start	End	Miss	Ions	Peptide

943.5581	942.5508	942.4883	66.3	178 - 186	0	---	K.NSVGLPNSR.A
1064.6072	1063.5999	1063.6100	-9.49	522 - 530	0	---	K.SVFLMLNIK.K
1066.5148	1065.5075	1065.5091	-1.44	257 - 265	1	---	R.DYAVNKDNK.A
1234.6902	1233.6829	1233.5990	68.0	60 - 70	0	---	K.DQLENAGFNVK.M
1320.5936	1319.5864	1319.7085	-92.55	167 - 177	1	---	K.NLVYDNKVLDK.N
1323.6793	1322.6720	1322.6718	0.19	343 - 353	1	---	K.DDNSIYDKLIK.A
1357.7238	1356.7166	1356.7249	-6.11	327 - 337	1	---	K.ILKDEDIDQLR.K
1365.6565	1364.6492	1364.6725	-17.03	404 - 414	0	---	K.FVNQVNQEYPK.G
1390.6914	1389.6841	1389.7616	-55.78	367 - 378	1	---	K.VNNKDGIYQIVK.I
1434.7666	1433.7593	1433.6357	86.2	309 - 320	0	---	K.SVPNNNTYNMHK.H + Oxidati
1493.7394	1492.7322	1492.7674	-23.61	403 - 414	1	18	K.KFVNQVNQEYPK.G
1618.7046	1617.6974	1617.8362	-85.83	388 - 402	0	---	R.EVNYLTHSSAGILSK.K

No match to: 807.3999, 832.4835, 856.5285, 870.5457, 897.4207, 973.5402, 1028.4847, 1037.5513, 1051.6991, 1052.6447, 1060.5854, 1074.5430, 1090.5585, 1107.5630, 1109.5211, 1111.5941, 1140.5769, 1150.6550, 1157.6067, 1184.6118, 1193.6299, 1201.6527, 1262.6852, 1265.6542, 1277.7111, 1307.6798, 1308.6631, 1314.7742, 1329.6530, 1338.6701, 1340.6933, 1373.6674, 1383.6947, 1393.7380, 1427.8365, 1458.7253, 1475.7605, 1487.7463, 1566.7300, 1586.7234, 1632.7336, 1657.7836, 1688.7715, 1699.8070, 1706.7773, 1708.7559, 1722.7759, 1738.7724, 1754.7672, 1770.7611, 1817.9139, 1838.8982, 1851.8839, 1940.8981, 2225.0635, 2286.0353, 2383.9128, 2398.9758, 2705.1300, 2717.0471, 3312.3408

39. gi|89893362 **Mass:** 93668 **Score:** 63 **Expect:** 1.5 **Queries matched:** 18
hypothetical protein DSY0616 [Desulfitobacterium hafniense Y51]

Observed	Mr(expt)	Mr(calc)	ppm	Start End	Miss	Ions	Peptide
807.3999	806.3927	806.4286	-44.61	418 - 424	0	---	R.ELVYAGR.A
856.5285	855.5212	855.5654	-51.61	579 - 585	1	---	R.RILSIVR.C
973.5402	972.5329	972.5392	-6.50	377 - 384	1	---	R.NGDWIKLK.C
1037.5513	1036.5441	1036.5488	-4.53	572 - 579	1	---	R.MLPYVSRR.I + Oxidation (
1201.6527	1200.6455	1200.5961	41.1	589 - 598	1	---	K.GVAQTCFYKK.H
1262.6852	1261.6780	1261.7156	-29.84	33 - 42	1	---	R.FVVQHHLARR.D
1320.5936	1319.5864	1319.6721	-64.99	196 - 207	0	---	R.NPFSSTEVQLAK.L
1340.6933	1339.6860	1339.6772	6.57	283 - 293	0	---	K.TDFQALQNYLK.N
1393.7380	1392.7307	1392.6674	45.5	476 - 486	0	---	K.FADWTEENLLR.Q

1434.7666	1433.7593	1433.8129	-37.38	682 - 694	0	---	K.AILAELSLNSYLK.T
1458.7253	1457.7180	1457.6796	26.4	586 - 597	1	---	R.CPKGVAQTCFYK.K
1618.7046	1617.6974	1617.7861	-54.86	49 - 62	0	---	R.LEWDGAMLSWAVPK.G + Oxida
1688.7715	1687.7642	1687.9257	-95.68	196 - 210	1	---	R.NPFSSTEVQLAKLVR.H
1706.7773	1705.7700	1705.7903	-11.85	267 - 282	0	2	R.AMILDGEMAITDPEGK.T + Oxi
1722.7759	1721.7687	1721.7852	-9.59	267 - 282	0	---	R.AMILDGEMAITDPEGK.T + 2 O
1738.7724	1737.7652	1737.7727	-4.32	178 - 192	1	---	R.TGRTMTEIEEGEEEK.F
1754.7672	1753.7599	1753.7676	-4.39	178 - 192	1	---	R.TGRTMTEIEEGEEEK.F + Oxid
2225.0635	2224.0562	2224.0589	-1.22	211 - 228	1	---	R.HLPEGEGWLYELKYDGYR.I

No match to: 832.4835, 870.5457, 897.4207, 943.5581, 1028.4847, 1051.6991, 1052.6447, 1060.5854, 1064.6072, 1066.5148, 1074.5430, 1090.5585, 1107.5630, 1109.5211, 1111.5941, 1140.5769, 1150.6550, 1157.6067, 1184.6118, 1193.6299, 1234.6902, 1265.6542, 1277.7111, 1307.6798, 1308.6631, 1314.7742, 1323.6793, 1329.6530, 1338.6701, 1357.7238, 1365.6565, 1373.6674, 1383.6947, 1390.6914, 1427.8365, 1475.7605, 1487.7463, 1493.7394, 1566.7300, 1586.7234, 1632.7336, 1657.7836, 1699.8070, 1708.7559, 1770.7611, 1817.9139, 1838.8982, 1851.8839, 1940.8981, 2286.0353, 2383.9128, 2398.9758, 2705.1300, 2717.0471, 3312.3408

40. gi|109648252 **Mass:** 24507 **Score:** 62 **Expect:** 1.7 **Queries matched:** 8
conserved hypothetical protein [Desulfitobacterium hafniense DCB-2]

Observed	Mr(expt)	Mr(calc)	ppm	Start - End	Miss	Ions	Peptide
832.4835	831.4763	831.5582	-98.52	102 - 108	1	---	R.IFAKILK.A
943.5581	942.5508	942.5134	39.6	211 - 218	1	---	R.RLLQEGDL.-
1037.5513	1036.5441	1036.5263	17.2	73 - 81	0	---	R.VEEIMAFAK.R
1193.6299	1192.6226	1192.6274	-4.01	73 - 82	1	---	R.VEEIMAFAKR.I
1338.6701	1337.6628	1337.7013	-28.74	109 - 120	1	---	K.AKGLESYGIICK.V
1493.7394	1492.7322	1492.7378	-3.76	88 - 101	0	14	K.IGIATCVGLMSEAR.I + Oxida
1586.7234	1585.7161	1585.7624	-29.18	55 - 69	0	---	K.LSNAAAEIEGTYYGK.L
1708.7559	1707.7487	1707.8866	-80.74	111 - 126	1	---	K.GLESYGIICKVGAVDK.T

No match to: 807.3999, 856.5285, 870.5457, 897.4207, 973.5402, 1028.4847, 1051.6991, 1052.6447, 1060.5854, 1064.6072, 1066.5148, 1074.5430, 1090.5585, 1107.5630, 1109.5211, 1111.5941, 1140.5769, 1150.6550, 1157.6067, 1184.6118, 1201.6527, 1234.6902, 1262.6852, 1265.6542, 1277.7111, 1307.6798, 1308.6631, 1314.7742, 1320.5936, 1323.6793, 1329.6530, 1340.6933, 1357.7238, 1365.6565, 1373.6674, 1383.6947, 1390.6914, 1393.7380, 1427.8365,

1434.7666, 1458.7253, 1475.7605, 1487.7463, 1566.7300, 1618.7046, 1632.7336, 1657.7836, 1688.7715, 1699.8070, 1706.7773, 1722.7759, 1738.7724, 1754.7672, 1770.7611, 1817.9139, 1838.8982, 1851.8839, 1940.8981, 2225.0635, 2286.0353, 2383.9128, 2398.9758, 2705.1300, 2717.0471, 3312.3408

41. gi|49482442 **Mass:** 67044 **Score:** 62 **Expect:** 1.7 **Queries matched:** 12
RGD-containing lipoprotein [Staphylococcus aureus subsp. aureus MRSA252]

Observed	Mr(expt)	Mr(calc)	ppm	Start	End	Miss	Ions	Peptide
943.5581	942.5508	942.4883	66.3	178 -	186	0	---	K.NSVGLPNSR.A
1064.6072	1063.5999	1063.6100	-9.49	522 -	530	0	---	K.SVFLMLNIK.K
1066.5148	1065.5075	1065.5091	-1.44	257 -	265	1	---	R.DYAVNKDNK.A
1234.6902	1233.6829	1233.5990	68.0	60 -	70	0	---	K.DQLENAGFNVK.M
1320.5936	1319.5864	1319.7085	-92.55	167 -	177	1	---	K.NLVYDNKVLDK.N
1323.6793	1322.6720	1322.6718	0.19	343 -	353	1	---	K.DDNSIYDKLIK.A
1357.7238	1356.7166	1356.7249	-6.11	327 -	337	1	---	K.ILKDEDIDQLR.K
1365.6565	1364.6492	1364.6725	-17.03	404 -	414	0	---	K.FVNQVNQEYPK.G
1390.6914	1389.6841	1389.7616	-55.78	367 -	378	1	---	K.VNNKDGIYQIVK.I
1434.7666	1433.7593	1433.6357	86.2	309 -	320	0	---	K.SVPNNNTYNMHK.H + Oxidati
1493.7394	1492.7322	1492.7674	-23.61	403 -	414	1	18	K.KFVNQVNQEYPK.G
1618.7046	1617.6974	1617.8362	-85.83	388 -	402	0	---	R.EVNYLTHSSAGILSK.K

No match to: 807.3999, 832.4835, 856.5285, 870.5457, 897.4207, 973.5402, 1028.4847, 1037.5513, 1051.6991, 1052.6447, 1060.5854, 1074.5430, 1090.5585, 1107.5630, 1109.5211, 1111.5941, 1140.5769, 1150.6550, 1157.6067, 1184.6118, 1193.6299, 1201.6527, 1262.6852, 1265.6542, 1277.7111, 1307.6798, 1308.6631, 1314.7742, 1329.6530, 1338.6701, 1340.6933, 1373.6674, 1383.6947, 1393.7380, 1427.8365, 1458.7253, 1475.7605, 1487.7463, 1566.7300, 1586.7234, 1632.7336, 1657.7836, 1688.7715, 1699.8070, 1706.7773, 1708.7559, 1722.7759, 1738.7724, 1754.7672, 1770.7611, 1817.9139, 1838.8982, 1851.8839, 1940.8981, 2225.0635, 2286.0353, 2383.9128, 2398.9758, 2705.1300, 2717.0471, 3312.3408

42. gi|153167459 **Mass:** 70979 **Score:** 62 **Expect:** 1.8 **Queries matched:** 11
hypothetical protein LMHG_01079 [Listeria monocytogenes FSL N1-017]

Observed	Mr(expt)	Mr(calc)	ppm	Start	End	Miss	Ions	Peptide
832.4835	831.4763	831.4702	7.34	429 -	435	1	---	K.LKSDLEK.I

1037.5513	1036.5441	1036.5553	-10.82	409 - 416	1	---	K.IETYRDIK.Q
1066.5148	1065.5075	1065.4397	63.7	547 - 555	1	---	K.KQDGDDNMK.E + Oxidation
1201.6527	1200.6455	1200.6714	-21.59	70 - 80	0	---	K.VELIIQSTNGK.Y
1320.5936	1319.5864	1319.6721	-65.00	589 - 599	0	---	K.QVNQIVFTEDK.N
1323.6793	1322.6720	1322.6210	38.6	460 - 470	0	---	K.EMSELLDSMLR.N
1390.6914	1389.6841	1389.6300	38.9	97 - 108	0	---	K.ITSSGEYVDYEK.Y
1493.7394	1492.7322	1492.7377	-3.74	417 - 428	1	14	K.QQLISMEKMQNK.L + Oxidati
1706.7773	1705.7700	1705.8635	-54.80	161 - 176	0	8	R.VNTSASVLFPGETTQR.T
1817.9139	1816.9066	1816.9215	-8.21	1 - 14	1	---	-.MIMQNYYIERVTIK.G + Oxida
2705.1300	2704.1227	2704.3344	-78.26	208 - 230	1	---	R.RNSVINYINSSLSELYENYEVAK.

No match to: 807.3999, 856.5285, 870.5457, 897.4207, 943.5581, 973.5402, 1028.4847, 1051.6991, 1052.6447, 1060.5854, 1064.6072, 1074.5430, 1090.5585, 1107.5630, 1109.5211, 1111.5941, 1140.5769, 1150.6550, 1157.6067, 1184.6118, 1193.6299, 1234.6902, 1262.6852, 1265.6542, 1277.7111, 1307.6798, 1308.6631, 1314.7742, 1329.6530, 1338.6701, 1340.6933, 1357.7238, 1365.6565, 1373.6674, 1383.6947, 1393.7380, 1427.8365, 1434.7666, 1458.7253, 1475.7605, 1487.7463, 1566.7300, 1586.7234, 1618.7046, 1632.7336, 1657.7836, 1688.7715, 1699.8070, 1708.7559, 1722.7759, 1738.7724, 1754.7672, 1770.7611, 1838.8982, 1851.8839, 1940.8981, 2225.0635, 2286.0353, 2383.9128, 2398.9758, 2717.0471, 3312.3408

43. gi|150015592 **Mass:** 16889 **Score:** 62 **Expect:** 1.8 **Queries matched:** 8
transcriptional regulator, MarR family [Clostridium beijerinckii NCIMB 8052]

Observed	Mr(expt)	Mr(calc)	ppm	Start	End	Miss	Ions	Peptide
856.5285	855.5212	855.5178	4.05	23 -	29	0	---	K.LIDLNIR.S
1184.6118	1183.6045	1183.6125	-6.73	49 -	58	0	---	K.ELSEFLYVGK.S
1201.6527	1200.6455	1200.6601	-12.22	120 -	129	0	---	K.EEEQTVIILK.R
1329.6530	1328.6458	1328.7551	-82.28	119 -	129	1	---	K.KEEEQTVIILK.R
1357.7238	1356.7166	1356.7612	-32.93	120 -	130	1	---	K.EEEQTVIILKR.I
1393.7380	1392.7307	1392.7038	19.3	30 -	41	0	---	R.SGQHDFLYVISK.N
1566.7300	1565.7228	1565.8705	-94.34	101 -	114	0	---	K.IDATFLELVAIFSK.S
1940.8981	1939.8908	1939.9891	-50.67	42 -	58	1	---	K.NEGISQKELSEFLYVGK.S

No match to: 807.3999, 832.4835, 870.5457, 897.4207, 943.5581, 973.5402, 1028.4847, 1037.5513, 1051.6991, 1052.6447, 1060.5854, 1064.6072, 1066.5148, 1074.5430, 1090.5585, 1107.5630, 1109.5211, 1111.5941, 1140.5769, 1150.6550, 1157.6067, 1193.6299, 1234.6902,

1262.6852, 1265.6542, 1277.7111, 1307.6798, 1308.6631, 1314.7742, 1320.5936, 1323.6793, 1338.6701, 1340.6933, 1365.6565, 1373.6674, 1383.6947, 1390.6914, 1427.8365, 1434.7666, 1458.7253, 1475.7605, 1487.7463, 1493.7394, 1586.7234, 1618.7046, 1632.7336, 1657.7836, 1688.7715, 1699.8070, 1706.7773, 1708.7559, 1722.7759, 1738.7724, 1754.7672, 1770.7611, 1817.9139, 1838.8982, 1851.8839, 2225.0635, 2286.0353, 2383.9128, 2398.9758, 2705.1300, 2717.0471, 3312.3408

44. gi|154249526 **Mass:** 42276 **Score:** 61 **Expect:** 2 **Queries matched:** 8

hypothetical protein Fnod_0841 [Fervidobacterium nodosum Rt17-B1]

Observed	Mr(expt)	Mr(calc)	ppm	Start	End	Miss	Ions	Peptide
1060.5854	1059.5781	1059.6189	-38.48	358 -	365	1	---	R.FLILRNER.V
1066.5148	1065.5075	1065.5495	-39.39	34 -	42	0	---	K.LTFFPQEGK.L
1150.6550	1149.6477	1149.6730	-22.01	324 -	333	1	---	K.AHLLRSALNR.M
1201.6527	1200.6455	1200.6397	4.80	101 -	109	1	---	K.CQNEILRLR.V
1338.6701	1337.6628	1337.6649	-1.54	110 -	120	0	---	R.VNYIENMAIQK.V + Oxidatio
1340.6933	1339.6860	1339.6329	39.6	1 -	12	0	---	-.MGSFTIEIDAEK.L
1365.6565	1364.6492	1364.6711	-16.04	210 -	220	0	---	K.TFDLEEIEEIK.I
1493.7394	1492.7322	1492.8290	-64.85	88 -	100	0	23	R.VNLLFQDGIVTFK.C

No match to: 807.3999, 832.4835, 856.5285, 870.5457, 897.4207, 943.5581, 973.5402, 1028.4847, 1037.5513, 1051.6991, 1052.6447, 1064.6072, 1074.5430, 1090.5585, 1107.5630, 1109.5211, 1111.5941, 1140.5769, 1157.6067, 1184.6118, 1193.6299, 1234.6902, 1262.6852, 1265.6542, 1277.7111, 1307.6798, 1308.6631, 1314.7742, 1320.5936, 1323.6793, 1329.6530, 1357.7238, 1373.6674, 1383.6947, 1390.6914, 1393.7380, 1427.8365, 1434.7666, 1458.7253, 1475.7605, 1487.7463, 1566.7300, 1586.7234, 1618.7046, 1632.7336, 1657.7836, 1688.7715, 1699.8070, 1706.7773, 1708.7559, 1722.7759, 1738.7724, 1754.7672, 1770.7611, 1817.9139, 1838.8982, 1851.8839, 1940.8981, 2225.0635, 2286.0353, 2383.9128, 2398.9758, 2705.1300, 2717.0471, 3312.3408

45. gi|21672982 **Mass:** 92806 **Score:** 61 **Expect:** 2 **Queries matched:** 16

DNA gyrase subunit A [Chlorobium tepidum TLS]

Observed	Mr(expt)	Mr(calc)	ppm	Start	End	Miss	Ions	Peptide
832.4835	831.4763	831.4524	28.7	127 -	134	0	---	K.AIAGEMLK.D
870.5457	869.5384	869.5698	-36.13	301 -	307	1	---	R.LVIELKR.D
1028.4847	1027.4774	1027.5774	-97.33	789 -	797	1	---	R.LIRLDAGDR.I

1037.5513	1036.5441	1036.5158	27.3	677 - 686	1	---	R.SMGRTAMGVK.G
1066.5148	1065.5075	1065.5091	-1.44	436 - 443	1	---	R.QKIDDEYR.E
1107.5630	1106.5557	1106.5827	-24.45	125 - 134	1	---	R.MKAIAGEMLK.D + Oxidation
1111.5941	1110.5868	1110.5526	30.8	696 - 704	1	---	R.CISMVTTKR.N + Oxidation
1157.6067	1156.5994	1156.5473	45.1	397 - 407	0	---	R.QSPDTPAAQSR.L
1308.6631	1307.6558	1307.6469	6.80	364 - 374	1	---	R.TQYDLNAAEKR.A
1323.6793	1322.6720	1322.7194	-35.83	651 - 662	0	---	R.LTDGDHQIILAK.S
1329.6530	1328.6458	1328.7009	-41.52	6 - 16	0	---	K.ILPISIEEEMR.D
1458.7253	1457.7180	1457.6544	43.7	347 - 356	1	---	K.EMMQYYIRHR.N + 2 Oxidati
1586.7234	1585.7161	1585.8385	-77.18	4 - 16	1	7	R.EKILPISIEEEMR.D
1632.7336	1631.7263	1631.8770	-92.35	255 - 268	0	---	R.ESIIVTELPYQVNK.V
1688.7715	1687.7642	1687.7948	-18.11	397 - 411	1	---	R.QSPDTPAAQSRLMDR.F + Oxid
2286.0353	2285.0280	2285.1362	-47.32	17 - 36	1	---	R.DSYLDYSMSVIVSRALPDVR.D

No match to: 807.3999, 856.5285, 897.4207, 943.5581, 973.5402, 1051.6991, 1052.6447, 1060.5854, 1064.6072, 1074.5430, 1090.5585, 1109.5211, 1140.5769, 1150.6550, 1184.6118, 1193.6299, 1201.6527, 1234.6902, 1262.6852, 1265.6542, 1277.7111, 1307.6798, 1314.7742, 1320.5936, 1338.6701, 1340.6933, 1357.7238, 1365.6565, 1373.6674, 1383.6947, 1390.6914, 1393.7380, 1427.8365, 1434.7666, 1475.7605, 1487.7463, 1493.7394, 1566.7300, 1618.7046, 1657.7836, 1699.8070, 1706.7773, 1708.7559, 1722.7759, 1738.7724, 1754.7672, 1770.7611, 1817.9139, 1838.8982, 1851.8839, 1940.8981, 2225.0635, 2383.9128, 2398.9758, 2705.1300, 2717.0471, 3312.3408

46. gi|121527739 **Mass:** 35386 **Score:** 61 **Expect:** 2 **Queries matched:** 9

putative integrase/recombinase protein [Ralstonia pickettii 12J]

Observed	Mr(expt)	Mr(calc)	ppm	Start	End	Miss	Ions	Peptide
1064.6072	1063.5999	1063.5662	31.7	222 -	231	0	---	R.TADALFVTAR.G
1201.6527	1200.6455	1200.6575	-10.00	211 -	221	1	---	R.QTGRTALLGER.T
1365.6565	1364.6492	1364.7538	-76.62	303 -	313	1	---	R.LRTLHAQHHPR.G
1487.7463	1486.7391	1486.7568	-11.95	46 -	57	0	---	R.DLTLYAHWLAER.N
1586.7234	1585.7161	1585.8365	-75.93	192 -	205	1	---	R.LVPFGAEAGDWLRR.Y
1657.7836	1656.7763	1656.7963	-12.07	148 -	161	1	---	R.DRAMIELMYASGLR.V + 2 Oxi
1699.8070	1698.7998	1698.8552	-32.61	232 -	245	1	---	R.GEGMTRQAFWYLIK.R
2398.9758	2397.9686	2398.1376	-70.47	25 -	44	1	---	R.FCDALWLEDGLARNTLDAYR.R

3312.3408	3311.3335	3311.5881	-76.86	8 -	37	1	---	K.DAEAPSLPPQSADAIQRFCDALWL

No match to: 807.3999, 832.4835, 856.5285, 870.5457, 897.4207, 943.5581, 973.5402, 1028.4847, 1037.5513, 1051.6991, 1052.6447, 1060.5854, 1066.5148, 1074.5430, 1090.5585, 1107.5630, 1109.5211, 1111.5941, 1140.5769, 1150.6550, 1157.6067, 1184.6118, 1193.6299, 1234.6902, 1262.6852, 1265.6542, 1277.7111, 1307.6798, 1308.6631, 1314.7742, 1320.5936, 1323.6793, 1329.6530, 1338.6701, 1340.6933, 1357.7238, 1373.6674, 1383.6947, 1390.6914, 1393.7380, 1427.8365, 1434.7666, 1458.7253, 1475.7605, 1493.7394, 1566.7300, 1618.7046, 1632.7336, 1688.7715, 1706.7773, 1708.7559, 1722.7759, 1738.7724, 1754.7672, 1770.7611, 1817.9139, 1838.8982, 1851.8839, 1940.8981, 2225.0635, 2286.0353, 2383.9128, 2705.1300, 2717.0471

47. gi|26554375 **Mass:** 87671 **Score:** 61 **Expect:** 2.1 **Queries matched:** 14
phenylalanyl-tRNA synthetase beta subunit [Mycoplasma penetrans HF-2]

Observed	Mr(expt)	Mr(calc)	ppm	Start	End	Miss	Ions	Peptide
832.4835	831.4763	831.4967	-24.53	417 -	423	1	---	K.VKAPVYR.E
943.5581	942.5508	942.5134	39.6	706 -	713	0	---	K.NNLNLLDK.F
1028.4847	1027.4774	1027.5161	-37.66	407 -	415	0	---	K.LMGFNFVGK.K + Oxidation
1037.5513	1036.5441	1036.5301	13.4	29 -	37	1	---	K.VEKHPNADK.L
1111.5941	1110.5868	1110.5961	-8.38	714 -	722	0	---	K.FEIESVFIK.D
1140.5769	1139.5697	1139.6161	-40.79	407 -	416	1	---	K.LMGFNFVGKK.V
1308.6631	1307.6558	1307.7085	-40.28	369 -	380	1	---	K.VSSVNYVGDKLK.D
1329.6530	1328.6458	1328.7493	-77.88	472 -	482	1	---	K.YFINKGFTLVK.T
1338.6701	1337.6628	1337.7303	-50.40	256 -	267	1	---	K.EIAVSLNAHKEK.F
1383.6947	1382.6874	1382.7380	-36.61	404 -	415	1	---	K.NLKLMGFNFVGK.K + Oxidati
1390.6914	1389.6841	1389.7286	-32.01	503 -	514	1	---	K.SIKIMNPISSER.E + Oxidati
1427.8365	1426.8292	1426.8031	18.3	2 -	14	0	---	M.LNSIGVEVENIIK.F
1657.7836	1656.7763	1656.7930	-10.03	506 -	518	1	---	K.IMNPISSEREYFR.N + Oxidat
1699.8070	1698.7998	1698.9152	-67.92	743 -	756	1	---	K.ETNEINEIVERLIK.D

No match to: 807.3999, 856.5285, 870.5457, 897.4207, 973.5402, 1051.6991, 1052.6447, 1060.5854, 1064.6072, 1066.5148, 1074.5430, 1090.5585, 1107.5630, 1109.5211, 1150.6550, 1157.6067, 1184.6118, 1193.6299, 1201.6527, 1234.6902, 1262.6852, 1265.6542, 1277.7111, 1307.6798, 1314.7742, 1320.5936, 1323.6793, 1340.6933, 1357.7238, 1365.6565, 1373.6674, 1393.7380, 1434.7666, 1458.7253, 1475.7605, 1487.7463, 1493.7394, 1566.7300, 1586.7234, 1618.7046, 1632.7336, 1688.7715, 1706.7773, 1708.7559, 1722.7759, 1738.7724, 1754.7672,

1770.7611, 1817.9139, 1838.8982, 1851.8839, 1940.8981, 2225.0635, 2286.0353, 2383.9128, 2398.9758, 2705.1300, 2717.0471, 3312.3408

48. gi|138895630 **Mass:** 9671 **Score:** 61 **Expect:** 2.2 **Queries matched:** 8
Molybdopterin biosynthesis MoeB-like protein [Geobacillus thermodenitrificans NG80-2]

Observed	Mr(expt)	Mr(calc)	ppm	Start		End	Miss	Ions	Peptide
897.4207	896.4134	896.4174	-4.51	2	-	9	0	---	M.VMNFSGSR.R
1028.4847	1027.4774	1027.4579	19.0	1	-	9	0	---	-.MVMNFSGSR.R
1074.5430	1073.5357	1073.5175	16.9	66	-	75	0	---	K.ASGHTVIDMK.E + Oxidation
1184.6118	1183.6045	1183.5590	38.4	1	-	10	1	---	-.MVMNFSGSRR.S
1314.7742	1313.7669	1313.6948	54.9	24	-	35	1	---	K.GLACPMPVVRAK.K + Oxidati
1393.7380	1392.7307	1392.7105	14.5	11	-	23	1	---	R.SSMMNVAKVLDAK.G
1493.7394	1492.7322	1492.7384	-4.19	76	-	87	0	---	K.EENGVLMFWIQK.G
1566.7300	1565.7228	1565.7548	-20.47	76	-	88	1	---	K.EENGVLMFWIQKG.- + Oxidat

No match to: 807.3999, 832.4835, 856.5285, 870.5457, 943.5581, 973.5402, 1037.5513, 1051.6991, 1052.6447, 1060.5854, 1064.6072, 1066.5148, 1090.5585, 1107.5630, 1109.5211, 1111.5941, 1140.5769, 1150.6550, 1157.6067, 1193.6299, 1201.6527, 1234.6902, 1262.6852, 1265.6542, 1277.7111, 1307.6798, 1308.6631, 1320.5936, 1323.6793, 1329.6530, 1338.6701, 1340.6933, 1357.7238, 1365.6565, 1373.6674, 1383.6947, 1390.6914, 1427.8365, 1434.7666, 1458.7253, 1475.7605, 1487.7463, 1586.7234, 1618.7046, 1632.7336, 1657.7836, 1688.7715, 1699.8070, 1706.7773, 1708.7559, 1722.7759, 1738.7724, 1754.7672, 1770.7611, 1817.9139, 1838.8982, 1851.8839, 1940.8981, 2225.0635, 2286.0353, 2383.9128, 2398.9758, 2705.1300, 2717.0471, 3312.3408

49. gi|118045195 **Mass:** 52397 **Score:** 61 **Expect:** 2.3 **Queries matched:** 10
histidyl-tRNA synthetase [Chloroflexus aggregans DSM 9485]

Observed	Mr(expt)	Mr(calc)	ppm	Start		End	Miss	Ions	Peptide
1037.5513	1036.5441	1036.4978	44.7	453	-	460	0	---	R.EGEYVVWR.D
1074.5430	1073.5357	1073.5903	-50.86	229	-	238	0	---	R.VLAAMGIDLR.T + Oxidation
1193.6299	1192.6226	1192.6057	14.2	1	-	10	1	---	-.MTVDLVRGMR.D + Oxidation
1314.7742	1313.7669	1313.7052	47.0	239	-	250	1	---	R.TGTRSPAEVIQR.L
1329.6530	1328.6458	1328.7565	-83.30	177	-	187	1	---	R.TRGLLVWSLER.L
1357.7238	1356.7166	1356.7184	-1.33	415	-	426	1	---	R.MSGHTVVLDVRK.R + Oxidati

1434.7666	1433.7593	1433.8466	-60.89	158 - 170	1	---	R.IGHIGLARELLSR.F
1493.7394	1492.7322	1492.7674	-23.62	364 - 377	0	9	R.QSVPAVGFAYGLER.V
1657.7836	1656.7763	1656.7600	9.88	8 - 21	1	---	R.GMRDVMPAEYLTSR.H + 2 Oxi
1838.8982	1837.8909	1837.9587	-36.88	99 - 113	1	---	R.LSYAGPVFRYERPQR.H

No match to: 807.3999, 832.4835, 856.5285, 870.5457, 897.4207, 943.5581, 973.5402, 1028.4847, 1051.6991, 1052.6447, 1060.5854, 1064.6072, 1066.5148, 1090.5585, 1107.5630, 1109.5211, 1111.5941, 1140.5769, 1150.6550, 1157.6067, 1184.6118, 1201.6527, 1234.6902, 1262.6852, 1265.6542, 1277.7111, 1307.6798, 1308.6631, 1320.5936, 1323.6793, 1338.6701, 1340.6933, 1365.6565, 1373.6674, 1383.6947, 1390.6914, 1393.7380, 1427.8365, 1458.7253, 1475.7605, 1487.7463, 1566.7300, 1586.7234, 1618.7046, 1632.7336, 1688.7715, 1699.8070, 1706.7773, 1708.7559, 1722.7759, 1738.7724, 1754.7672, 1770.7611, 1817.9139, 1851.8839, 1940.8981, 2225.0635, 2286.0353, 2383.9128, 2398.9758, 2705.1300, 2717.0471, 3312.3408

50. gi|88797880 **Mass:** 40810 **Score:** 61 **Expect:** 2.3 **Queries matched:** 11

hypothetical protein MED297_12030 [Reinekea sp. MED297]

Observed	Mr(expt)	Mr(calc)	ppm	Start - End	Miss	Ions	Peptide
832.4835	831.4763	831.4450	37.6	220 - 226	0	---	R.QTISVER.K
1074.5430	1073.5357	1073.5328	2.71	296 - 304	0	---	K.MNQPVWATK.Y
1090.5585	1089.5512	1089.5277	21.5	296 - 304	0	---	K.MNQPVWATK.Y + Oxidation
1201.6527	1200.6455	1200.6350	8.71	316 - 327	0	---	K.SPGVTLSANDLK.Q
1314.7742	1313.7669	1313.6688	74.7	101 - 114	1	---	K.SRGAPAAVEQGGSK.G
1393.7380	1392.7307	1392.6972	24.0	296 - 306	1	---	K.MNQPVWATKYR.L
1434.7666	1433.7593	1433.6946	45.2	154 - 165	1	---	R.ACINNHIGHDRK.R
1618.7046	1617.6974	1617.6844	8.03	72 - 84	0	---	K.DEPTGDFFMVMWK.S + Oxidat
1706.7773	1705.7700	1705.8159	-26.86	85 - 100	1	6	K.SDEDANGKLYAAPIDK.S
1708.7559	1707.7487	1707.8376	-52.07	151 - 164	1	---	K.FVRACINNHIGHDR.K
2225.0635	2224.0562	2224.0484	3.52	2 - 20	1	---	M.AVNGHASYFEVPECGLYRR.N

No match to: 807.3999, 856.5285, 870.5457, 897.4207, 943.5581, 973.5402, 1028.4847, 1037.5513, 1051.6991, 1052.6447, 1060.5854, 1064.6072, 1066.5148, 1107.5630, 1109.5211, 1111.5941, 1140.5769, 1150.6550, 1157.6067, 1184.6118, 1193.6299, 1234.6902, 1262.6852, 1265.6542, 1277.7111, 1307.6798, 1308.6631, 1320.5936, 1323.6793, 1329.6530, 1338.6701, 1340.6933, 1357.7238, 1365.6565, 1373.6674, 1383.6947, 1390.6914, 1427.8365, 1458.7253, 1475.7605, 1487.7463, 1493.7394, 1566.7300, 1586.7234, 1632.7336, 1657.7836, 1688.7715, 1699.8070, 1722.7759, 1738.7724, 1754.7672, 1770.7611, 1817.9139, 1838.8982, 1851.8839,

1940.8981, 2286.0353, 2383.9128, 2398.9758, 2705.1300, 2717.0471, 3312.3408

Search Parameters

```
Type of search         : MS/MS Ion Search
Enzyme                 : Trypsin
Fixed modifications    : Carbamidomethyl (C)
Variable modifications : Oxidation (M)
Mass values            : Monoisotopic
Protein Mass           : Unrestricted
Peptide Mass Tolerance : ± 100 ppm
Fragment Mass Tolerance: ± 0.8 Da
Max Missed Cleavages   : 1
Instrument type        : MALDI-TOF-TOF
Query1 (807.3999,1+)   : <no title>
Query2 (832.4835,1+)   : <no title>
Query3 (856.5285,1+)   : <no title>
Query4 (870.5457,1+)   : <no title>
Query5 (897.4207,1+)   : <no title>
Query6 (943.5581,1+)   : <no title>
Query7 (973.5402,1+)   : <no title>
Query8 (1028.4847,1+)  : <no title>
Query9 (1037.5513,1+)  : <no title>
Query10 (1051.6991,1+) : <no title>
Query11 (1052.6447,1+) : <no title>
Query12 (1060.5854,1+) : <no title>
Query13 (1064.6072,1+) : <no title>
Query14 (1066.5148,1+) : <no title>
Query15 (1074.5430,1+) : <no title>
Query16 (1090.5585,1+) : <no title>
Query17 (1107.5630,1+) : <no title>
Query18 (1109.5211,1+) : <no title>
Query19 (1111.5941,1+) : <no title>
Query20 (1140.5769,1+) : <no title>
Query21 (1150.6550,1+) : <no title>
Query22 (1157.6067,1+) : <no title>
```

```
Query23 (1184.6118,1+) : <no title>
Query24 (1193.6299,1+) : <no title>
Query25 (1201.6527,1+) : <no title>
Query26 (1234.6902,1+) : <no title>
Query27 (1262.6852,1+) : <no title>
Query28 (1265.6542,1+) : <no title>
Query29 (1277.7111,1+) : <no title>
Query30 (1307.6798,1+) : <no title>
Query31 (1308.6631,1+) : <no title>
Query32 (1314.7742,1+) : <no title>
Query33 (1320.5936,1+) : <no title>
Query34 (1323.6793,1+) : <no title>
Query35 (1329.6530,1+) : <no title>
Query36 (1338.6701,1+) : <no title>
Query37 (1340.6933,1+) : <no title>
Query38 (1357.7238,1+) : <no title>
Query39 (1365.6565,1+) : <no title>
Query40 (1373.6674,1+) : <no title>
Query41 (1383.6947,1+) : <no title>
Query42 (1390.6914,1+) : <no title>
Query43 (1393.7380,1+) : <no title>
Query44 (1427.8365,1+) : <no title>
Query45 (1434.7666,1+) : <no title>
Query46 (1458.7253,1+) : <no title>
Query47 (1475.7605,1+) : <no title>
Query48 (1487.7463,1+) : <no title>
Query49 (1493.7394,1+) : <no title>
Query50 (1566.7300,1+) : <no title>
Query51 (1586.7234,1+) : <no title>
Query52 (1618.7046,1+) : <no title>
Query53 (1632.7336,1+) : <no title>
Query54 (1657.7836,1+) : <no title>
Query55 (1688.7715,1+) : <no title>
Query56 (1699.8070,1+) : <no title>
Query57 (1706.7773,1+) : <no title>
Query58 (1708.7559,1+) : <no title>
Query59 (1722.7759,1+) : <no title>
```

```
Query60 (1738.7724,1+) : <no title>
Query61 (1754.7672,1+) : <no title>
Query62 (1770.7611,1+) : <no title>
Query63 (1817.9139,1+) : <no title>
Query64 (1838.8982,1+) : <no title>
Query65 (1851.8839,1+) : <no title>
Query66 (1940.8981,1+) : <no title>
Query67 (2225.0635,1+) : <no title>
Query68 (2286.0353,1+) : <no title>
Query69 (2383.9128,1+) : <no title>
Query70 (2398.9758,1+) : <no title>
Query71 (2705.1300,1+) : <no title>
Query72 (2717.0471,1+) : <no title>
Query73 (3312.3408,1+) : <no title>
```

Mascot: http://www.matrixscience.com/
