# Supplementary material for: Proteomic analysis of Salmonella enterica serovar Enteritidis following propionate adaptation
Source: BMC Microbiol. 2010 Sep 28;10:249. doi: 10.1186/1471-2180-10-249 (PMC2957393; doi:10.1186/1471-2180-10-249)
Supplement: Additional file 4 — Protein Report D. Mass spectrometry report for CpxR and Dps [file 1471-2180-10-249-S4.PDF]

Protein Identification Report

Order 11779

Nicole Calhown

University of Arkansas

Date: November 06, 2008

Overview

Mass spectrometric peptide mapping and sequencing analysis

Customer samples:

Sample name	Protein found in database	GI-number	MW	Score	Seq. cov.	Note
PA2	DNA-binding transcriptional regulator CpxR [Shigella flexneri 5 str. 8401]	gi|110807397	23714	185	27%	
PA2	2,3-bisphosphoglycerate-dependent phosphoglycerate mutase [Shigella dysenteriae 1012]	gi|194435028	28539	74	13%	
PA6	DNA starvation/stationary phase protection protein Dps [Salmonella enterica subsp. enterica serovar Typhi str. CT18]	gi|16759749	18706	482	52%	

Quality control standards included in the analysis

Standard	Protein found in database	GI-number	MW	Score	Seq. cov.	Note
1 pmol BSA	albumin [Bos taurus]	gi|30794280	69278	936	36%	
62 fmol Transferrin	transferrin [Homo sapiens]	gi|115394517	76910	128	12%	

Alphalyse Inc. ■ 200 Page Mill Road, Suite 100 ■ Palo Alto, CA 94306 ■ USA
Tel: (650) 543-3193 ■ e-mail mail@pick-n-post.com
www.pick-n-post.com

Experimental

Samples received

The following samples were received at Alphalyse for protein analysis.

PA2
PA6

Protein identification by MS peptide mapping and sequencing analysis

The protein samples were reduced and alkylated with iodoacetamide, i.e. carbamidomethylated, and digested with trypsin that cleaves after lysine and arginine residues. The resulting peptides were concentrated on a ZipTip micropurification column and eluted onto an anchorchip target for analysis on a Bruker Autoflex III MALDI TOF/TOF instrument. The peptide mixture was analyzed in positive reflector mode for accurate peptide mass determination and 5-10 of the peptides selected for analysis by MS/MS fragmentation for partial peptide sequencing. The MS and MS/MS spectra were combined and used for a database search in an in-house protein database using the Mascot software.

Matching proteins are found in the database based on the number of matching peptide masses and the peptide fragment masses. The protein identification is based on a probability-scoring algorithm (www.matrixscience.com) and the significant best matching protein is shown in the result report. Homologous proteins with a lower score are not included in the report, but if the protein from the correct organism is not present in the database, then a significant matching homologous protein from another organism may be reported. If a sample contains several proteins and they are found with a significant score, they are all reported.

The identified database protein sequences are shown in the Results together with the obtained mass spectrometric peptide maps. The peptides used for the identification are highlighted in the sequence and the peptide masses listed for comparison of the determined and calculated values. Peptide identities confirmed by MS/MS sequencing are shown in bold.

Bioinformatics analysis

Alphalyse has collected a range of Bioinformatics tools for further analysis of the proteins. Selected tools are hyperlinked for each identified protein in the identification report. The database entry for the identified protein can be looked up at the NCBI website (National Center for Biotechnology Information) by selecting the NCBI Entry hyperlink. A Blast homology search against the NCBI database is found by selecting Blink NCBI. Known functional domains in the protein can be found in the Conserved Domain Database (Conserved Domains in NCBI).

The Bioinformatics Guide at (www.pick-n-post.com) explains and guides you through a range of important bioinformatics tools to let you investigate the function and properties of the protein. The guide contains hyperlinks to bioinformatics search forms and case examples how the tools are used.

Alphalyse Inc. ■ 200 Page Mill Road, Suite 100 ■ Palo Alto, CA 94306 ■ USA
Tel: (650) 543-3193 ■ e-mail mail@pick-n-post.com
www.pick-n-post.com

Protein Identification Report

Order 11779

Results

Sample name: PA2

Protein Information

Protein name:	DNA-binding transcriptional regulator CpxR [Shigella flexneri 5 str. 8401]
Alphalyse number:	ALPHA12734
GI-number:	gi|110807397
MW:	23714
pI:	5,8
Mascot score:	185
Sequence coverage:	27%
Bioinformatic tools:	1: NCBI Entry 2: Blink (Blast) NCBI 3: Conserved Domains in NCBI

Analysis Information

- MS analysis method: MALDI-TOF peptide mass fingerprint and MALDI-TOF/TOF peptide sequencing
- Enzyme: Trypsin
- Variable modifications: Carbamidomethyl (C),Oxidation (M)
- Database search program: Mascot version 2.2.03
- Peptide Tolerance: 60 ppm
- Allowed up to 1 miscleavage
- Database: NRDB1 (7155275 protein sequences)

Protein sequence

Matched peptides shown in bold underline

```
  1 MEGFNVIVAH DGEQALDLLD DSIDLLLLDV MMPKKNGIDT LKALRQTHQT
 51  PVIMLTARGS ELDRVLGLEL GADDYLPKPF NDRELVARIR  AILRRSHWSE
101 QQQNNDNGSP TLEVDALVLN PGRQEASFDG QTLELTGTEF TLLYLLAQHL
151 GQVVSREHLS QEVLGKRLTP FDRAIDMHIS NLRRKLPDRK DGHPWFKTLR
201 GRGYLMVSAS
```

Peptides used for identification

Peptides shown in bold have been analysed by MS/MS sequencing

Start - End	Observed	Mr(expt)	Mr(calc)	Delta	Miss	Sequence
46 - 58	1495.75	1494.74	1494.80	-36.00	0	R.QTHQTPVIMLTAR.G
**46 - 58**	**1511.75**	**1510.75**	**1510.79**	**-30.00**	**0**	**R.QTHQTPVIMLTAR.G Oxidation (M) (Ions score 33)**
84 - 90	856.50	855.49	855.53	-44.00	1	R.ELVARIR.A
157 - 166	1139.56	1138.55	1138.60	-43.00	0	R.EHLSQEVLGK.R
**157 - 167**	**1295.68**	**1294.67**	**1294.70**	**-24.00**	**1**	**R.EHLSQEVLGKR.L (Ions score 33)**
**167 - 173**	**904.47**	**903.46**	**903.49**	**-32.00**	**1**	**K.RLTPFDR.A (Ions score 11)**
174 - 183	1169.57	1168.56	1168.60	-36.00	0	R.AIDMHISNLR.R
**174 - 183**	**1185.57**	**1184.56**	**1184.60**	**-31.00**	**0**	**R.AIDMHISNLR.R Oxidation (M) (Ions score 39)**
174 - 184	1341.65	1340.64	1340.70	-41.00	1	R.AIDMHISNLRR.K Oxidation (M)

Alphalyse Inc. ■ 200 Page Mill Road, Suite 100 ■ Palo Alto, CA 94306 ■ USA
Tel: (650) 543-3193 ■ e-mail mail@pick-n-post.com
www.pick-n-post.com

201 - 210	1040.49	1039.49	1039.51	-24.00	1	R.GRGYLMVSAS.-
201 - 210	1056.47	1055.46	1055.51	-40.00	1	R.GRGYLMVSAS.- Oxidation (M)

Alphalyse Inc. ■ 200 Page Mill Road, Suite 100 ■ Palo Alto, CA 94306 ■ USA
Tel: (650) 543-3193 ■ e-mail mail@pick-n-post.com
www.pick-n-post.com

Protein Identification Report

Order 11779

Sample name: PA2

Protein Information

**Protein name:**	**2,3-bisphosphoglycerate-dependent phosphoglycerate mutase [Shigella dysenteriae 1012]**
Alphalyse number:	ALPHA12734
GI-number:	gi|194435028
MW:	28539
pI:	5,85
Mascot score:	74
Sequence coverage:	13%
Bioinformatic tools:	1: NCBI Entry 2: Blink (Blast) NCBI 3: Conserved Domains in NCBI

Analysis Information

- MS analysis method: MALDI-TOF peptide mass fingerprint and MALDI-TOF/TOF peptide sequencing
- Enzyme: Trypsin
- Variable modifications: Carbamidomethyl (C),Oxidation (M)
- Database search program: Mascot version 2.2.03
- Peptide Tolerance: 60 ppm
- Allowed up to 1 miscleavage
- Database: NRDB1 (7155275 protein sequences)

Protein sequence

Matched peptides shown in bold underline

```
  1 MAVTKLVLVR HGESQWNKEN RFTGWYDVDL SEKGVSEAKA AGKLLKEEGY
 51 SFDFAYTSVL KRAIHTLWNV LDELDQAWLP VEKSWKLNER HYGALQGLNK
101 AETAEKYGDE QVKQWRRGFA VTPPELTKDD ERYPGHDPRY AKLSEKELPL
151 TESLALTIDR VIPYWNETIL PRMKSGERVL IAAHGNSLRA LVKYLDNMSE
201 EEILELNIPT GVPLVYEFDE NFKPLKRYYL GNADEIAAKA AAVANQGKAK
251
```

Peptides used for identification

Peptides shown in bold have been analysed by MS/MS sequencing

Start - End	Observed	Mr(expt)	Mr(calc)	Delta	Miss	Sequence
91 - 100	1100.55	1099.54	1099.58	-32.00	0	R.HYGALQGLNK.A
**179 - 189**	**1150.64**	**1149.63**	**1149.66**	**-26.00**	**0**	**R.VLIAAHGNSLR.A (Ions score 60)**
228 - 239	1327.65	1326.64	1326.65	-1.00	0	R.YYLGNADEIAAK.A

Alphalyse Inc. ■ 200 Page Mill Road, Suite 100 ■ Palo Alto, CA 94306 ■ USA
Tel: (650) 543-3193 ■ e-mail mail@pick-n-post.com
www.pick-n-post.com

Protein Identification Report

Order 11779

Sample name: PA6

Protein Information

Protein name:	DNA starvation/stationary phase protection protein Dps [Salmonella enterica subsp. enterica serovar Typhi str. CT18]
Alphalyse number:	ALPHA12735
GI-number:	gi|16759749
MW:	18706
pI:	5,73
Mascot score:	482
Sequence coverage:	52%
Bioinformatic tools:	1: NCBI Entry 2: Blink (Blast) NCBI 3: Conserved Domains in NCBI

Analysis Information

- MS analysis method: MALDI-TOF peptide mass fingerprint and MALDI-TOF/TOF peptide sequencing
- Enzyme: Trypsin
- Variable modifications: Carbamidomethyl (C),Oxidation (M)
- Database search program: Mascot version 2.2.03
- Peptide Tolerance: 60 ppm
- Allowed up to 1 miscleavage
- Database: NRDB1 (7155275 protein sequences)

Protein sequence

Matched peptides shown in bold underline

```
  1 MSTAKLVKTK ASNLLYTRND VSESDKKATV ELLNRQVIQF IDLSLITKQA
 51 HWNMRGANFI AVHEMLDGFR TALTDHLDTM AERAVQLGGV ALGTTQVINS
101 KTPLKSYPLD IHNVQDHLKE LADRYAVVAN DVRKAIGEAK DEDTADIFTA
151 ASRDLDKFLW FIESNIE
```

Peptides used for identification

Peptides shown in bold have been analysed by MS/MS sequencing

Start - End	Observed	Mr(expt)	Mr(calc)	Delta	Miss	Sequence
**9 - 18**	**1166.62**	**1165.61**	**1165.65**	**-30.00**	**1**	**K.TKASNLLYTR.N (Ions score 38)**
11 - 18	937.48	936.47	936.50	-31.00	0	K.ASNLLYTR.N
**28 - 35**	**915.50**	**914.49**	**914.52**	**-31.00**	**0**	**K.ATVELLNR.Q (Ions score 30)**
49 - 55	958.39	957.38	957.42	-46.00	0	K.QAHWNMR.G Oxidation (M)
56 - 70	1692.77	1691.76	1691.81	-29.00	0	R.GANFIAVHEMLDGFR.T Oxidation (M)
71 - 83	1473.66	1472.66	1472.69	-25.00	0	R.TALTDHLDTMAER.A
**71 - 83**	**1489.64**	**1488.63**	**1488.69**	**-39.00**	**0**	**R.TALTDHLDTMAER.A Oxidation (M) (Ions score 57)**
120 - 133	1590.77	1589.76	1589.82	-33.00	1	K.ELADRYAVVANDVR.K
**125 - 133**	**1006.50**	**1005.49**	**1005.52**	**-35.00**	**0**	**R.YAVVANDVR.K (Ions score 28)**
**125 - 134**	**1134.59**	**1133.58**	**1133.62**	**-34.00**	**1**	**R.YAVVANDVRK.A (Ions score 38)**
**135 - 153**	**1980.90**	**1979.90**	**1979.94**	**-24.00**	**1**	**K.AIGEAKDEDTADIFTAASR.D (Ions score 148)**

Alphalyse Inc. ■ 200 Page Mill Road, Suite 100 ■ Palo Alto, CA 94306 ■ USA
Tel: (650) 543-3193 ■ e-mail mail@pick-n-post.com
www.pick-n-post.com

141 - 153	1411.59	1410.58	1410.63	-32.00	0	K.DEDTADIFTAASR.D

Alphalyse Inc. ■ 200 Page Mill Road, Suite 100 ■ Palo Alto, CA 94306 ■ USA
Tel: (650) 543-3193 ■ e-mail mail@pick-n-post.com
www.pick-n-post.com

Quality control standards

Sample name: 1 pmol BSA

Protein Information

**Protein name:**	**albumin [Bos taurus]**
Alphalyse number:	Standard1
GI-number:	gi|30794280
MW:	69278
pI:	5,82
Mascot score:	936
Sequence coverage:	36%
Bioinformatic tools:	1: NCBI Entry 2: Blink (Blast) NCBI 3: Conserved Domains in NCBI

Analysis Information

- MS analysis method: MALDI-TOF peptide mass fingerprint and MALDI-TOF/TOF peptide sequencing
- Enzyme: Trypsin
- Variable modifications: Carbamidomethyl (C),Oxidation (M)
- Database search program: Mascot version 2.2.03
- Peptide Tolerance: 60 ppm
- Allowed up to 1 miscleavage
- Database: NRDB1 (7155275 protein sequences)

Protein sequence

Matched peptides shown in bold underline

1 MKWVTFISLL LLFSSAYSRG VFRRDTHKSE IAHR**FKDLGE EHFK**GLVLIA
51 FSQYLQQCPF DEHVK**LVNEL TEFAK**TCVAD ESHAGCEK**SL HTLFGDELCK**
101 **VASLRETYGD MADCCEKQEP ERNECFLSHK DDSPDLPK**LK PDPNTLCDEF**
151 **KADEK**KFWGK **YLYEIAR**RHP YFYAPELLYY ANKYNGVFQE CCQAEDKGAC
201 LLPKIETMRE KVLTSSARQR LRCASIQKFG ERALKAWSVA RLSQKFPKAE
251 FVEVTKLVTD LTKVHK**ECCH GDLLECADDR ADLAK**YICDN QDTISSK**LKE**
301 **CCDKPLLEK**S HCIAEVEKDA IPENLPPLTA DFAEDKDVCK NYQEAK**DAFL**
351 **GSFLYEYSRR HPEYAVSVLL R**LAKEYEATL EECCAK**DDPH ACYSTVFDKL**
401 **KHLVDEPQNL IK**QNCDQFEK **LGEYGFQNAL IVR**YTR**KVPQ VSTPTLVEVS**
451 **R**SLGKVGTRC CTKPESER**MP CTEDYLSLIL NR**LCVLHEKT PVSEKVTKCC
501 TESLVNR**RPC FSALTPDETY VPK**AFDEKLF TFHADICTLP DTEKQIK**KQT**
551 **ALVELLK**HKP KATEEQLKTV MENFVAFVDK **CCAADDKEAC FAVEGPK**LVV
601 STQTALA

Peptides used for identification

Peptides shown in bold have been analysed by MS/MS sequencing

Start - End	Observed	Mr(expt)	Mr(calc)	Delta	Miss	Sequence
**35 - 44**	**1249.57**	**1248.56**	**1248.61**	**-**	**1**	**R.FKDLGEEHFK.G (Ions score 70)**

Alphalyse Inc. ■ 200 Page Mill Road, Suite 100 ■ Palo Alto, CA 94306 ■ USA
Tel: (650) 543-3193 ■ e-mail mail@pick-n-post.com
www.pick-n-post.com

				**40.00**		
66 - 75	1163.57	1162.57	1162.62	-48.00	0	K.LVNELTEFAK.T
**89 - 100**	**1419.64**	**1418.63**	**1418.69**	**-39.00**	**0**	**K.SLHTLFGDELCK.V Carbamidomethyl (C) (Ions score 70)**
139 - 155	2019.93	2018.93	2018.96	-17.00	1	K.LKPDPNTLCDEFKADEK.K Carbamidomethyl (C)
**161 - 167**	**927.46**	**926.46**	**926.49**	**-32.00**	**0**	**K.YLYEIAR.R (Ions score 46)**
267 - 285	2247.92	2246.91	2246.94	-11.00	1	K.ECCHGDLLECADDRADLAK.Y 3 Carbamidomethyl (C)
298 - 309	1532.73	1531.72	1531.77	-34.00	1	K.LKECCDKPLLEK.S 2 Carbamidomethyl (C)
**347 - 359**	**1567.70**	**1566.70**	**1566.74**	**-25.00**	**0**	**K.DAFLGSFLYEYSR.R (Ions score 113)**
**360 - 371**	**1439.77**	**1438.76**	**1438.80**	**-32.00**	**1**	**R.RHPEYAVSVLLR.L (Ions score 64)**
361 - 371	1283.66	1282.66	1282.70	-37.00	0	R.HPEYAVSVLLR.L
387 - 401	1795.78	1794.77	1794.82	-29.00	1	K.DDPHACYSTVFDKLK.H Carbamidomethyl (C)
**402 - 412**	**1305.66**	**1304.65**	**1304.71**	**-42.00**	**0**	**K.HLVDEPQNLIK.Q (Ions score 94)**
**421 - 433**	**1479.75**	**1478.75**	**1478.79**	**-28.00**	**0**	**K.LGEYGFQNALIVR.Y (Ions score 125)**
**437 - 451**	**1639.90**	**1638.89**	**1638.93**	**-23.00**	**1**	**R.KVPQVSTPTLVEVSR.S (Ions score 94)**
438 - 451	1511.79	1510.78	1510.84	-34.00	0	K.VPQVSTPTLVEVSR.S
469 - 482	1740.77	1739.76	1739.82	-33.00	0	R.MPCTEDYLSLILNR.L Carbamidomethyl (C); Oxidation (M)
**508 - 523**	**1880.89**	**1879.89**	**1879.91**	**-15.00**	**0**	**R.RPCFSALTPDETYVPK.A Carbamidomethyl (C) (Ions score 102)**
548 - 557	1142.66	1141.65	1141.71	-49.00	1	K.KQTALVELLK.H
581 - 597	1927.77	1926.76	1926.79	-15.00	1	K.CCAADDKEACFAVEGPK.L 3 Carbamidomethyl (C)

Alphalyse Inc. ■ 200 Page Mill Road, Suite 100 ■ Palo Alto, CA 94306 ■ USA
Tel: (650) 543-3193 ■ e-mail mail@pick-n-post.com
www.pick-n-post.com

Protein Identification Report

Order 11779

Sample name: 62 fmol Transferrin

Protein Information

**Protein name:**	**transferrin [Homo sapiens]**
Alphalyse number:	Standard2
GI-number:	gi|115394517
MW:	76910
pI:	6,97
Mascot score:	128
Sequence coverage:	12%
Bioinformatic tools:	1: NCBI Entry 2: Blink (Blast) NCBI 3: Conserved Domains in NCBI

Analysis Information

- MS analysis method: MALDI-TOF peptide mass fingerprint and MALDI-TOF/TOF peptide sequencing
- Enzyme: Trypsin
- Variable modifications: Carbamidomethyl (C),Oxidation (M)
- Database search program: Mascot version 2.2.03
- Peptide Tolerance: 60 ppm
- Allowed up to 1 miscleavage
- Database: NRDB1 (7155275 protein sequences)

Protein sequence

Matched peptides shown in bold underline

```
  1 MRLAVGALLV CAVLGLCLAV PDKTVRWCAV SEHEATKCQS FRDHMKSVIP
 51 SDGPSVACVK KASYLDCIRA IAANEADAVT LDAGLVYDAY LAPNNLKPVV
101 AEFYGSKEDP QTFYYAVAVV KKDSGFQMNQ LRGKKSCHTG LGRSAGWNIP
151 IGLLYCDLPE PRKPLEKAVA NFFSGSCAPC ADGTDFPQLC QLCPGCGCST
201 LNQYFGYSGA FKCLKDGAGD VAFVKHSTIF ENLANKADRD QYELLCLDNT
251 RKPVDEYKDC HLAQVPSHTV VARSIGGKED LIWELLNQAQ EHFGKDKSKE
301 FQLFSSPHGK DLLFKDSAHG FLKVPPRMDA KMYLGYEYVT AIRNLREGTC
351 PEAPTDECKP VKWCALSHHE RLKCDEWSVN SVGKIECVSA ETTEDCIAKI
401 MNGEADAMSL DGGFVYIAGK CGLVPVLAEN YNKSDNCEDT PGAGYFAVAV
451 VKKSASDLTW DNLKGKKSCH TAVGRTAGWN IPMGLLYNKI NHCRFDEFFS
501 EGCAPGSKKD SSLCKLCMGS GLNLCEPNNK EGYYGYTGAF RCLVEKGDVA
551 FVKHQTVPQN TGGKNPDPWA KNLNEKDYEL LCLDGTRKPV EEYANCHLAR
601 APNHAVVTRK DKEACVHKIL RQQQHLFGSN VTDCSGNFCL FRSETKDLLF
651 RDDTVCLAKL HDRNTYEKYL GEEYVKAVGN LRKCSTSSLL EACTFRRP
```

Peptides used for identification

Peptides shown in bold have been analysed by MS/MS sequencing

Start - End	Observed	Mr(expt)	Mr(calc)	Delta	Miss	Sequence
**61 - 69**	**1125.57**	**1124.56**	**1124.56**	**-1.00**	**1**	**K.KASYLDCIR.A Carbamidomethyl (C) (Ions score 38)**
**62 - 69**	**997.48**	**996.47**	**996.47**	**-1.00**	**0**	**K.ASYLDCIR.A Carbamidomethyl (C) (Ions score 12)**
123 - 132	1211.53	1210.52	1210.54	-16.00	0	K.DSGFQMNQLR.G Oxidation (M)
332 - 343	1494.67	1493.66	1493.72	-40.00	0	K.MYLGYEYVTAIR.N Oxidation (M)

Alphalyse Inc. ■ 200 Page Mill Road, Suite 100 ■ Palo Alto, CA 94306 ■ USA
Tel: (650) 543-3193 ■ e-mail mail@pick-n-post.com
www.pick-n-post.com

363 - 371	1195.54	1194.53	1194.54	0.00	0	K.WCALSHHER.L Carbamidomethyl (C)
**531 - 541**	**1283.55**	**1282.55**	**1282.56**	**-11.00**	**0**	**K.EGYYGYTGAFR.C (Ions score 38)**
588 - 600	1586.73	1585.72	1585.77	-29.00	0	R.KPVEEYANCHLAR.A Carbamidomethyl (C)
669 - 676	1000.48	999.47	999.49	-18.00	0	K.YLGEEYVK.A
684 - 696	1531.66	1530.65	1530.68	-19.00	0	K.CSTSSLLEACTFR.R 2 Carbamidomethyl (C)

Alphalyse Inc. ■ 200 Page Mill Road, Suite 100 ■ Palo Alto, CA 94306 ■ USA
Tel: (650) 543-3193 ■ e-mail mail@pick-n-post.com
www.pick-n-post.com
